# Fitness costs of mobilised colistin resistance gene 3 (*mcr-3*): systematic review, epidemiological study, and functional analysis

**DOI:** 10.1016/j.ebiom.2025.105923

**Published:** 2025-09-12

**Authors:** Lujie Liang, Yaxin Li, Lin Wang, Wenli Wang, Yihao Zhang, Hui Zhao, Yaxuan Wang, Lingxuan Lyu, Jiachen Li, Dianrong Zhou, Zhe Hu, Lizhen Luo, Guanxiu Wang, Jia Wan, Lin Xu, Meisong Li, Min Dai, Meiting Yang, Shun Xiong, Lan-Lan Zhong, Fang Bai, Siyuan Feng, Guo-Bao Tian

**Affiliations:** a Department of Immunology, School of Medicine, Shenzhen Campus of Sun Yat-sen University, Shenzhen, 518107, Guangdong, China; b Advanced Medical Technology Centre, The First Affiliated Hospital, Zhongshan School of Medicine, Sun Yat-sen University, Guangzhou, 510080, China; c Programme in Pathobiology, The Fifth Affiliated Hospital, Zhongshan School of Medicine, Sun Yat-Sen University, Guangdong, 510080, China; d Key Laboratory of Tropical Diseases Control (Sun Yat-sen University), Ministry of Education, Guangzhou, 510080, China; e State Key Laboratory of Oncology in South China, Sun Yat-sen University Cancer Centre, Guangzhou, 510060, China; f Shanghai Institute for Advanced Immunochemical Studies and School of Life Science and Technology, Shanghai Tech University, Shanghai, 201210, China; g Division of Molecular Oncology, Graduate School of Medicine, Nagoya University, Aichi, Japan; h Laboratory Medicine, Guangdong Provincial People's Hospital, Guangdong Academy of Medical Sciences, Guangzhou, Guangdong, 510080, China; i College of Life Sciences, South China Agricultural University, Guangzhou, Guangdong, 510642, China; j Department of Clinical Laboratory Medicine, Third Affiliated Hospital of Guangzhou Medical University, Guangzhou, 510150, China; k School of Laboratory Medicine, Chengdu Medical College, Chengdu, China

**Keywords:** *mcr-1*, *mcr-3*, Prevalence, Colistin, Fitness, MCR inhibitors

## Abstract

**Background:**

The rapid evolution and dissemination of mobilised colistin resistance gene (*mcr*) family has revealed as a severe threat to the global public health. Nevertheless, dramatic reduction in the prevalence of *mcr-1*, the major member of *mcr* family, was observed after the withdrawal of colistin in animal fodder in China since 2017, demonstrating that colistin acts as a selective stress to promote the dissemination of *mcr-1*. As the second largest lineage, *mcr-3* was firstly discovered in 2017 and has been identified from numerous sources. However, whether the spreading of *mcr-3* is driven by colistin remains unknown.

**Methods:**

To this end, we investigated the global prevalence of *mcr-3* from 2005 to 2022 by an up-to-date systematic review, along with a nation-wide epidemiological study to establish the change of *mcr-3* prevalence in China before and after 2017. To investigate the fitness cost imposed by MCR-3 upon bacterial host, *in vitro* and *in vivo* competitive assays were employed, along with morphological study and fluorescent observation. Moreover, by replacing non-optimal codons with optimal codons, synonymous mutations were introduced into the 5′-coding regions of *mcr-3* to study mechanisms accounting for the distinct fitness cost conferred by MCR-1 and MCR-3. Furthermore, by combining AlphaFold and molecular dynamics (MD) simulation, we provided a complete characterisation on the putative lipid A binding pocket localised at the linker domain of MCR-3. Crucially, inhibitors targeting at the putative binding pocket of MCR-1 or MCR-3 were identified from small molecules library using the pipeline of virtual screening.

**Findings:**

The global prevalence of *mcr-3* increased continuously from 2005 to 2022. The average prevalence was 0.18% during 2005–2014 and rapidly increased to 3.41% during 2020–2022. The prevalence of *mcr-3* in China increased from 0.79% in 2016 to 5.87% in 2019. We found that the fitness of *mcr-3*-bearing *Escherichia coli* and empty plasmid control was comparable but higher than that of *mcr-1*-positive strain. Although the putative lipid A binding pocket of MCR-3 was similar to that of in MCR-1, *mcr-3* occupies remarkable codon bias at the 5′-end of coding region that disrupted the stability of mRNA, further reduced its protein expression in *E. coli*, resulting in the low fitness burden of bacterial host. Moreover, the 5′-end codon usage frequency appeared as a critical factor related with the evolution of *mcr* family. Furthermore, based on the similar lipid A binding pocket among MCR family protein, we identified three MCR inhibitors targeting at such pocket by screening from small-molecule library, which effectively restored the colistin susceptibility of *mcr*-bearing *E. coli*.

**Interpretation:**

We found that the prevalence of *mcr-3* increased continuously during 2016–2019 in China, demonstrating that the withdrawal of colistin in husbandry failed to prevent the dissemination of *mcr-3*. Our study evidenced that the 5′-end codon bias appeared as a crucial regulator upon the fitness cost conferred by horizontally transferred genes. Most importantly, the putative lipid A binding pocket verified from current study was a promising target site for designing inhibitors against *mcr*-positive strains.

**Funding:**

10.13039/501100001809Natural Science Foundation of China, National Key Research and Development Programme of China.

**Research in context**

**Evidence before this study**

We systematically searched relevant literatures from PubMed using the following terms: “*mcr-3*” AND “colistin” AND “resistance” AND “fitness”. There was no limitation in publication date and language. Until August 9th 2024, 4 studies have been identified. Two of them focused on the prevalence of *mcr*-bearing/colistin resistant within a small number of regions and sample sources. However, none of these studies provided comprehensive view upon the prevalence of *mcr-3* before and after the withdrawal of colistin in animal husbandry. Although the remaining articles investigated the impact of compensatory mutations/mobile elements upon the fitness of *mcr-1* or *mcr-3*-bearing strains, no convinced conclusion was given regarding how *mcr-1* or *mcr-3* gene itself impacts the fitness of bacterial host.

**Added value of this study**

Our study evidenced that the prevalence of *mcr-3* increased continuously during 2016–2019 in China. Previous studies have demonstrated that the reduced prevalence of *mcr-1* after the withdrawal of colistin in China was induced by the MCR-1-mediated fitness cost due to impaired membrane integrity. However, unlike *mcr-1*, the expression of MCR-3 does not compromise significant membrane integrity, which accounted for the low fitness burden of bacterial host. With further investigation, we found that this phenomenon was closely related to mRNA stability and protein expression of MCR-1/MCR-3. Crucially, the codon optimality at 5′-end of mRNA was a critical regulator for the mRNA stability of *mcr-1* or *mcr-3*. Comparing to *mcr-1*, more non-optimal codons clustered at the 5′-coding region of *mcr-3*, which reduced the mRNA stability and guaranteed the low fitness burden upon bacterial host. Collectively, our study comprehensively illustrated the mechanisms accounting for the distinct prevalence of *mcr-1* or *mcr-3*. Additionally, the MCR inhibitors identified in our study appeared to be effective compounds to restore the efficacy of colistin against *mcr-3*-bearing strain.

**Implications of all the available evidence**

Our current study evidenced that the withdrawal of colistin in husbandry failed to prevent the dissemination of *mcr-3*. Given the *mcr* family as an example, we found that the codon optimality of mRNA 5′-end appeared as a crucial regulator upon the fitness cost conferred by horizontally transferred genes. Furthermore, the MCR inhibitors were promising compounds that restore the colistin susceptibility of *mcr*-bearing *Escherichia coli*. Our study provides strategy against the potential outbreak of *mcr-3*.

## Introduction

Infection caused by multidrug-resistant gram-negative bacteria, particularly Enterobacteriaceae, is considered one of the greatest threats to global public health worldwide.^1^ Currently, at least 700,000 people die per year from antibiotic resistant pathogens,^2^ and the number may increase to 10 million by 2050.^3^ Colistin, a critically important antimicrobial for human health defined by the WHO in 2018, is the “last resort” antibiotic for the treatment of multidrug-resistant Enterobacteriaceae.^1^ However, the emergence and rapid dissemination of mobile colistin resistance (*mcr*) genes jeopardise and compromise the clinical treatment outcome of colistin.^4^, ^5^, ^6^ The International Network for Optimal Resistance Monitoring (INFORM) global surveillance Programme revealed that the worldwide prevalence of *mcr* from 2014 to 2016 was 3.2% among clinically isolated pathogens.^7^ To prevent the continuous dissemination of *mcr* genes, the Chinese Ministry of Agriculture issued the banning of colistin as an additive in livestock fodders in 2017. This implementation positively contributed to the reduced production of colistin and the nationwide prevalence of *mcr-1.**^8^* Our previous studies demonstrated that MCR-1 employs a putative lipid A binding pocket at the linker region that disrupts lipid A distribution, resulting in outer membrane defects and fitness loss in *E. coli*.^9^^,^^10^ Moreover, we have previously shown that the reduced prevalence of *mcr-1* after the withdrawal of colistin in animal husbandry is closely associated with the MCR-1-mediated fitness burden on the bacterial host.^11^

To date, the *mcr* family, comprised of *mcr-1* to *mcr-10*, has spread widely within different regions and various bacterial species.^12^^,^^13^ So far, totally 115 *mcr* family members have been identified. Among them, *mcr-1* and *mcr-3* are the largest lineages, which contribute 32.2% and 36.5% of the *mcr* family, respectively (Figure S1). Like MCR-1, MCR-3 consists of a C-terminal catalytic domain exposed in the periplasm, which is responsible for the interaction with the phosphatidylethanolamine (PE) substrate and is connected with a predicted five-transmembrane (TM) region anchored in the lipid bilayer of the inner membrane at the N-terminus through a flexible linker.^14^ By transferring the phosphoethanolamine (PEA) group from the PE donor to the 1′- or 4′-phosphate head of lipid A, MCR-3 thereby protects the bacterial host from the damage caused by colistin.^15^^,^^16^ Recently, the linker of a functional variant of MCR-3 was shown to facilitate colistin resistance in *Aeromonas* species.^14^ Although the *mcr-3* gene shares 45% nucleotide sequence identity with the *mcr-1* gene,^17^^,^^18^ it seems to exhibit a highly different transmission trajectory when compared with *mcr-1*. For example, the South Korean government issued the same banning policy in 2008, and the prevalence of *mcr-3* in food-producing pigs was nearly 0% in 2005 but increased to 6.7% in 2015 and 17.8% in 2018.^19^ Interestingly, several groups have shown that the expression of MCR-3 has no effect on the bacterial host.^20^ For example, the *mcr-3*-bearing plasmid stably persists in bacterial hosts even without the stress of colistin. These studies suggest that increased attention should be given to potential outbreaks of *mcr-3* even after the withdrawal of colistin in animal husbandry. However, studies focused on the fitness cost of *mcr-3* are extremely limited. Interestingly, a recent study has reported that the under expression of MCR-1 induced by promoter mutations leads to increased fitness in *mcr-1*-bearing bacteria,^21^ suggesting that protein expression level might impact the fitness of MCR-positive bacteria. Additionally, our previous study has established that the putative lipid A binding pocket of MCR-1 regulates the fitness cost in bacterial hosts.^9^ Although it seems likely that the protein structure of MCR-3 is similar to that of MCR-1, the mechanisms determining the varied fitness costs mediated by these MCR members remain unknown.

To close this knowledge gap and present a comprehensive view of the spread and fitness of *mcr-3*-positive bacteria, we investigated the dissemination of *mcr-3* from 2005 to 2022 through a systematic review and the influence of colistin on the prevalence of *mcr-3* in China. We also explored the mechanism by which MCR-3 affects bacterial fitness. Finally, we screened MCR-specific inhibitors from a small-molecule library.

## Methods

### Strains, antibiotics, and culture conditions

The standard bacterial strains applied in this research were the derivatives of *E. coli* K-12, including MG1655, BW25113, C600 and DH5-α. *E. coli* MG1655, BW25113 and C600 were utilised to assay the influence of MCR-1 or MCR-3 on the bacterial colistin sensitivity, fitness and membrane permeability, while DH5-α was cloning vesicle for plasmid construction. Meanwhile, to analyse the influence of MCR-3 expression upon the fitness of *E. coli* in the background of clinical isolated plasmids, plasmid conjugal transfer experiments were carried out as previously mentioned.^22^ *mcr-1-* or *mcr-3*-bearig strains were isolated from human/animal faecal samples. Streptomycin-resistant *E. coli* C600 was used as recipient and the *mcr-1*- or *mcr-3*-positive strains as donor. The conjugants were identified by PCR and used for competitive assays. All the experiments were performed in Luria Bertani broth (LB) at 37 °C. When necessary, antibiotic for maintaining target plasmid and inducer of arabinose promoter were supplemented as follows: 30 μg/mL chloramphenicol (CHL, Meilunbio,cat#MB2014-1,China), 100 μg/mL spectinomycin (SPM, Meilunbio,cat#MB1497,China), 100 μg/mL ampicillin (AMP, Meilunbio,cat#MB1378,China), 500 μg/mL rifampicin (RIF, Meilunbio,cat#MB1769,China), and 0.2% arabinose (Macklin,cat#L800478,China). All the strains used in this study were listed in Table S10.

### Epidemiological study

To investigate the prevalence of *mcr-3* in China, 1494 non-duplicated samples were collected from eight provinces in China (namely Guangdong, Shandong, Shanxi, Anhui, Yunnan, Liaoning, Sichuan and Guangxi), from 2016 to 2019. All of them were faecal samples from swine and farmers of swine farm, which were collected by sterile rectal swab and sent to the lab immediately. Previous studies have found that *mcr-3* was identified from both plasmid (e.g. *E. coli*) and chromosome (e.g. *Aeromonas veronii*).^18^^,^^23^ As previously described,^8^^,^^24^ to investigate the prevalence of plasmid-mediated *mcr-3*, all the faecal samples were cultured by adding 3 mL E*. coli* enrichment broth (MacConkey Broth, Sigma, cat#1.46382, USA) without antibiotics selection and cultivated at 37 °C overnight. Negative control was performed in every cultivation. Subsequently, total DNA was extracted by water-boiling method and screened for the presence of *mcr-3* using PCR. The positive amplicons were sent to BGI Genomics Co. (Guangzhou, China) for Sanger sequencing. Meanwhile, to confirm the phenotype of colistin resistance, the selected *mcr-3*^+^ strains were recultured on selective agar containing 2 μg/mL colistin (Meilunbio, cat#MB1188, China) for 18–24 h at 37 °C.

### Systematic review and meta-analysis

The systematic review and meta-analysis were conducted in accordance with the PRISMA (Preferred Reporting Items for Systematic Reviews and Meta-Analyses) guidelines, which collected literatures published by April 1st, 2023, from PubMed, EMBASE, Cochrane library and CNKI. This study is not registered in PROSPERO prospectively. No registration number is available. Following terms were searched: “*mcr-3*”, “prevalence”, “occurrence”, “incidence”, “epidemiology”. There is no specific limitation for language or regions. Two co-authors (LJ Liang and YH Zhang) performed literature screening independently. Inclusion criteria included: epidemiological studies reporting positive detection number of *mcr-3* and the total sample size from different sources of sampling: environment, human, animals. Exclusion criteria included: (1) no full text available; (2) conference abstract, posters, letters, case reports, reviews; (3) studies did not report positive detection number and/or full sample size. From an initial number of 222 articles, based on the title and abstract, 110 (92 repeated and 18 irrelevant articles) were removed. The remaining 112 articles were assessed for eligibility, and 1 article was excluded because of the lack of full text. For the 111 included publications, 33 (11 case reports, 8 reviews, 2 mechanisms studies, 3 methodological studies and 9 articles without describing the prevalence of *mcr-3*) were further excluded (Figure S2A). We finally included 78 publications for synthesis of the systematic review. Next, data were collected by two co-authors (LJ Liang and YH Zhang) independently. The following content were collected: author, publication year, sampling region, sampling time, sample source, sample type, positive sample count, sample size.

In general, data can be extracted from nearly all the included studies. Some missing data may exist for certain time point or region. The missing values are removed from analysis. No imputation is performed. Considering the high heterogeneity of included studies, random-effect model (DerSimonian and Laird inverse variance) was used because all the included studies were retrospective. Raw positive rate was first calculated as event dividing by sample size. Because most studies reported very small proportion of events, Freeman-Tukey double arcsine transformation was performed. Estimated pooled effect size was later back transformed to rate (%) for easier interpretation. To understand the contribution of confounding factors to the estimated pooled positive rate, Bayesian hierarchical analysis was further performed as a gold standard when data are heterogenous.^25^ We evaluated three major confounding factors: sampling location, sampling time and sampling source. In Bayesian hierarchical linear mixed model, we first adjusted raw positive rate to fit into the model. Before log transformation, a small value of 10^−5^ was added. Due to skewed data distribution, Gamma distribution was used for modelling. The model was iterated for 5000 times with 4 chains. In addition, subgroup analyses for different time periods, sampling regions and sampling sources were performed with DerSimonian and Laird random-effect model. We labelled sampling time on Funnel plot to indicate publication and sampling bias. Meta-analyses were performed via R package meta and rstanarm. Visualisation was performed using ggplot2, bayesplot and lessR.

### Plasmid construction

To express MCR-1, MCR-3 and its relative variants in *E. coli* K-12 strains, target DNA fragments were cloned into the plasmid backbone of pACYCDuet-1 as previously described.^9^ And the expression of target proteins was under the regulation of arabinose promoter or the native promoter of *mcr-1* or *mcr-3* amplified from *mcr-1*- or *mcr-3*-positive isolates from human samples (Figure S27). To introduce point-mutations into *mcr-3*, the DNA fragments encoding target mutants were amplified through overlapped PCR with appropriated primers as listed in Table S11. Meanwhile, to generate RraA overexpressing strain, the DNA fragment encoding RraA was amplified with the primers pBAD24-RraA-F/RraA-pBAD24-R, carrying 15–20 bp overlapping arms that were homologous to the plasmid backbone of pBAD24. And the plasmid backbone of pBAD24 was amplified using the primers pBAD24-F/pBAD24-R. The purified plasmid and target fragments were mixed in a molar ratio of 1:3, followed by adding 2× ClonExpress® Mix (ClonExpress Ultra One Step Cloning Kit, Vazyme, cat#C115-02, China) into the reaction and incubating in a thermocycler at 50 °C for 15 min. The well-reacted products were transformed into *E. coli* DH5-α competent cells for enrichment. All the constructs were sent to BGI Genomics Co. (Guangzhou, China) for Sanger sequencing. All the plasmids used in this study were listed as Table S12.

### DNA sequence alignment among native promoters regulating *mcr-3*

To determine the conservation upon the nucleotide sequence of *mcr-3* native promoter, the DNA sequences of *mcr-3*-bearing plasmids identified from our previous study were downloaded from NCBI database,^24^ namely GDZJ002 (MH043623), GDZJ003 (MH043625) and GDZJ004 (MH043626). The indicated plasmids were all isolated from human/animal faecal samples. Sequences alignment were subjected to MEGA (version 11) and visualised using ESPript (version 3.0).^26^ The DNA sequence encoding *mcr-3* promoter region among indicated plasmids were highly conserved (Figure S28).

### Agar dilution MICs assay

To evaluate the colistin and SDS–EDTA susceptibility of MCR-1-positve *E. coli*, MCR-3-positive *E. coli* and its related mutants in the background of BW25113, a MICs assay was performed with agar dilution method similar as previously mentioned.^9^ In brief, colistin or SDS–EDTA were added into Mueller–Hinton agar (MHA) containing 30 μg/mL CHL and 0.2% arabinose. Fresh single colonies of indicated strains were inoculated into fresh Mueller–Hinton broth (MHB) containing 30 μg/mL CHL and 0.2% arabinose, and cultivated at 37 °C. Exponential phase cultures were collected and then adjusted to an OD_600_ ranged from 0.5 to 0.6 by using a spectrophotometer (NP80, IMPLEN). The well-adjusted cultures were diluted in six gradients, ranging from 10^−1^ to 10^−6^, with saline. For each dilution, 3 μl culture was spotted onto the MHA plates containing colistin in different concentrations and incubated at 37 °C overnight. Strain carrying empty plasmid was set as control. Three replicates were carried out for each strain. The MIC values were defined as the concentration at which bacterial growth was absolutely inhibited. And the concentrations of tested agents were as follows: 0.125, 0.25, 0.5, 1, 2, 4, 8, 16 and 32 μg/mL for colistin; 0.001%, 0.01%, 0.1%, 1% and 2% for SDS (along with 100 μM EDTA).

### Growth curves measurement

Fresh single colonies of *E. coli* BW25113 carrying *mcr-1*, *mcr-3* or pACYCDuet-1 were inoculated into fresh Luria Bertani broth (LB) containing 30 μg/mL CHL and 0.2% arabinose, and cultivated at 37 °C. Exponential phase cultures were collected and then adjusted to an OD_600_ ranged from 0.5 to 0.6 by utilising a spectrophotometer (NP80, IMPLEN), following by dilution with saline in the ratio of 1:10. For each strain, 20 μl diluted culture was inoculated into 180 μl LB broth containing 0.2% arabinose and 30 μg/mL CHL in a 96-well plate. Three replicates were performed. A spectrophotometer plate reader (PowerWave 340, BioTek) was utilised to measure the optical density at the wavelength of 600 (OD_600_) of each well per hour. And the growth curve was plotted with Prism 9 software.

### *In vitro* competitive assay

To compare the fitness between *mcr-1*-positive and *mcr-3*-positive *E. coli* in the laboratory cultural condition, an *in vitro* competitive assay was carried out as previously described. Briefly, fresh single colonies of indicated strains and control strain carrying *gfp* were inoculated into fresh LB broth and cultivated overnight at 37 °C. Next, for each strain, the overnight culture was then adjusted to a same OD_600_ value by using a spectrophotometer (NP80, IMPLEN). The well-adjusted cultures of *mcr-1*- or *mcr-3*-positive strains were mixed with the same volume of control strain, respectively. The mixture was then sub-cultured into 1 mL LB broth and cultivated at 37 °C. For strains expressing MCR-1 or MCR-3 under the regulation of arabinose promoter, 30 μg/mL CHL and 0.2% arabinose were also supplemented in the media for induction. 100 μl culture of each sample was collected at 0, 24 and 48 h after initiation, and the percentage of GFP-negative population, regarding as the MCR positive proportion, was verified by a flow-cytometer (Gallios10, Beckman). And the sample without GFP was set as control for gating.

Meanwhile, to compare the fitness between *mcr-1*-positve cells and *mcr-3*-positive cells under the stress of colistin, same volume of well-adjusted cultures of *mcr-3*-positive strain and *mcr-1*-positve strain labelled with GFP were mixed thoroughly, following by sub-cultured into LB broth containing 2 or 4 μg/mL colistin. Similar procedures were performed for analysis. And the percentage of GFP-negative population was regarded as the fraction of *mcr-3*-positive strain, and GFP-positive population as *mcr-1*-positve strain. Three replicates were performed for each sample. The data was analysed with the FlowJo v.10 software and visualised with Prism 9 software.

### *In vivo* competitive assay

*In vivo* competition assay was performed to determine the fitness of *mcr-1*- or *mcr-3*-positive *E. coli* C600 in the environment of murine intestinal tract. Briefly, female specific pathogen free (SPF) BALB/c mice between 6 and 8 weeks of age were obtained from the Animal Supply Centre of Sun Yat-sen University, which were taken to minimise host-intrinsic variability and to strictly control for the potential confounding effects of host sex on bacterial fitness. All the animals received standard laboratory chow diet and double-deionised water, maintaining under specific pathogen-free conditions at the animal facility of the Sun Yat-sen University.

Fresh single colonies of *mcr-1*- or *mcr-3*-positive *E. coli* C600 and control strain carrying *kan*^*R*^ were inoculated into fresh LB broth and cultivated at 37 °C. Next, for each strain, exponential phase culture was collected and washed with saline for twice. After resuspension into saline, all the cultures were then adjusted to a same OD_600_ value by using a spectrophotometer (NP80, IMPLEN). The well-adjusted cultures of *mcr-1*- or *mcr-3*-positive strains were then mixed with the same volume of control strain, respectively, containing approximately 10^9^ colony forming units (CFUs) per millilitre. Mice were separated into two groups, and gently restrained and oral gavage with 100 μl mixture (10^8^ CFUs) containing *mcr-1*- or *mcr-3*-positive strains, respectively. Faecal sample of each mouse was collected at 8, 24 and 48 h after gavage. After resuspension into saline, pellet was collected by centrifugation (376 g for 3 min) and the supernatant was kept for verifying CFUs. After dilution in six gradients with saline (ranging from 10^−1^ to 10^−6^), 3 μl culture was spotted onto the LB plates containing 4 μg/mL colistin or 25 μg/mL kanamycin. CFUs was counted after incubation at 37 °C overnight. And the percentage of MCR positive strain was calculated as follows:

Percentage of MCR positive strain = CFUs^CT^/(CFUs^Kan^ + CFUs^CT^).

where CFUs^CT^ represented the CFUs value identified from LB plates containing 4 μg/mL colistin, and CFUs^Kan^ represented the CFUs value identified from LB plates containing 25 μg/mL kanamycin. Three replicates were carried out for each strain. The results were plotted with Prism 9 software.

### Time killing assay

To identify the viability of strains expressing MCR-1 or MCR-3 under variable stress, time killing assays were performed to determine the resistance of indicated strains towards the stresses of colistin, acid, and disinfectants. Fresh single colonies of *E. coli* BW25113 carrying *mcr-1*, *mcr-3* or pACYCDuet-1 were inoculated into fresh LB broth containing 30 μg/mL CHL and 0.2% arabinose, and cultivated at 37 °C. Exponential phase cultures were diluted with LB broth to the density of ∼10^6^ CFUs/mL and aliquoted for treatment with chemical reagents as follows: sodium hypochlorite (0.05% and 0.5%), hydrogen peroxide (0.03% and 0.3%), SDS-EDTA (0.01% and 0.1% SDS, with 100 μM EDTA), phenol (0.02% and 0.2%), iodophor (0.04 and 0.4 mM) and ethanol (0.5% and 5%). The samples without stress treatment were set as control. For each treatment, 100 μl culture was sampled at time intervals 0 h (T0) and 4 h (Tn), and diluted in six gradients, ranged from 10^−1^ to 10^−6^, with saline. Then, 3 μl of each diluent was spotted onto LB agar plates. Each treatment was repeated in triplicate. After incubation at 37 °C for 18 h, CFUs were counted to evaluate viability of target strains.

### Growth inhibition assay

Since *mcr-1*- or *mcr-3*-positive *E. coli* mainly colonise in the intestinal tract of host, the bacteria are challenged with various stresses, especially nutrient limitation and hypoxia. To test the growth rate of target strains under such environment, we mimicked the stresses inside the host intestinal tract by cultivating indicated strains under nutrition or oxygen-limited conditions.

To mimic the nutrition-limited condition, fresh single colonies of *E. coli* BW25113 harbouring *mcr-1*, *mcr-3* or pACYCDuet-1 were inoculated into fresh LB broth containing 30 μg/mL CHL and cultivated overnight at 37 °C. The overnight cultures were then adjusted to an OD_600_ ranged from 0.5 to 0.6 by using a spectrophotometer (NP80, IMPLEN). The well-adjusted culture was sub-cultured into 1 mL M9 minimal medium broth containing 30 μg/mL CHL and 0.2% arabinose and cultivated at 37 °C.

Meanwhile, to mimic the oxygen-limited condition, similarly, the well-adjusted overnight culture was sub-cultured into 1 mL LB broth containing 30 μg/mL CHL and 0.2% arabinose and cultivated at 37 °C in an anaerobic incubator.

All the above samples were collected at 0, 12, 24 and 48 h after treatment, and diluted in six gradients, ranging from 10^−1^ to 10^−6^, with saline. Then, 3 μl of each diluent was spotted onto LB agar plates. Each treatment was repeated in triplicate. After incubation at 37 °C for 18 h, CFUs were counted to evaluate viability.

### Fluorescent imaging

Based on previous research,^27^ the membrane shrinkage of *E. coli* was accompanied with the formation of foci at the two poles of the bacillus. To verify the membrane shrinkage of indicated strains caused by the expression of MCR-1 or MCR-3, a two-fluorescent reporter system was constructed by labelling the periplasm with enhanced GFP (EGFP) and cytoplasm with mCherry in the background of *E. coli* BW25113. For each strain, culture was collected after induction with 0.2% arabinose for 48 h. After the supplement with 1% glucose at 48 h, all the cultures were collected at 51 h and 60 h. Next, for each sample, 5 μl of bacterial culture was placed on a gel pad containing 1% agarose and covered with a coverslip. The GFP and mCherry signals were recorded with a fluorescent microscope (Olympus BX63) on phase-contrast equipped with 100× oil immersion objective and a xenon lamp. And the image merged with the two fluorescent signals were processed with Fuji software (version 2.14.0).

### Morphological analysis by SEM

The surface morphology of *E. coli* BW25113 expressing MCR-1 or MCR-3 were observed by scanning electron microscopy (SEM). Fresh single colonies of indicated strains and empty plasmid control were inoculated into fresh LB broth containing 30 μg/mL CHL and cultivated overnight at 37 °C. Next, the exponential phase cultures were then adjusted to an OD_600_ ranged from 0.5 to 0.6 by using a spectrophotometer (NP80, IMPLEN). The well-adjusted culture was sub-cultured into 1 mL LB broth containing 30 μg/mL CHL and 0.2% arabinose and cultivated at 37 °C, following by collecting samples at 12 h after induction. All the samples were harvested by centrifugation (1503 g for 3 min) and fixed with 2% glutaraldehyde (Servicebio, cat#G1102, China) for at least 4 h at room temperature, following by dehydration with graded ethanol series and air dry. The dried powder was loaded on a rotating stage, and sputtered with gold using a vacuum coater (Leica EM ACE200) and coating a 0.1 mm gold layer. Microscopy was performed with a desktop field emission scanning electron microscope (Phenom Pharos G2, Thermo Fisher Scientific). Images were collected using the secondary electron detector, and the acceleration voltage was adjusted to 10 kV. SEM images were recorded at magnifications ranging from 20,000× to 100,000×.

### 1-*N*-phenylnaphthylamine (NPN) uptake assay

To determine the integrity of bacterial outer membrane, fresh single colonies of *E. coli* BW25113 carrying *mcr-1*, *mcr-3* or pACYCDuet-1 were inoculated into fresh LB broth containing 30 μg/mL CHL and 0.2% arabinose, and cultivated at 37 °C. Exponential phase cultures were collected and harvested by centrifugation (1503 g for 3 min). After washing with assay buffer (5 mM HEPES, 5 mM glucose, pH = 7.2) for twice, cultures were resuspended in assay buffer to a final OD_600_ = 1. Next, 100 μl of washed cultures and 100 μl of assay buffer containing 20 μM NPN (MedChemExpress, cat#HY-W009756, USA) were mixed together and added to a 96-well half area black opaque plate (Greiner Bio), where assay buffer was set as control to remove background signal. The fluorescence for each well was then immediately monitored with a spectrophotometer microplate reader (PowerWave 340, BioTek) at an excitation wavelength of 350 nm and emission wavelength of 420 nm. And the NPN uptake was calculated as follows:

NPN uptake = *F*_*obs*_ *− F*_*ctrl*_

where *F*_*obs*_ represented the NPN uptake of different strains, and *F*_*ctrl*_ represented the background signal without addition of bacterial culture. Three replicates were carried out for each strain. The results were plotted with Prism 9 software.

### PI (propidium iodide) staining assay

To evaluate the inner membrane permeability of *E. coli* BW25113 carrying *mcr-1*, *mcr-3* or pACYCDuet-1, fresh single colonies of indicated strains and empty plasmid control were inoculated into fresh LB broth containing 30 μg/mL CHL and cultivated overnight at 37 °C. The overnight cultures were then adjusted to an OD_600_ ranged from 0.5 to 0.6 by using a spectrophotometer (NP80, IMPLEN). The well-adjusted culture was sub-cultured into 2 mL LB broth containing 30 μg/mL CHL and 0.2% arabinose and cultivated at 37 °C, following by collecting samples at 12 h and 24 h after induction. The collected cultures were then harvested by centrifugation (1503 g for 3 min), and washed twice with saline and resuspended with 95 μl buffer A (PI staining kit, Sangon Biotech, cat#E607306, China). After addition of 5 μl PI dye into each resuspended culture, samples were incubated in dark for 15 min. The percentage of PI-positive population of each sample was verified by a flow-cytometer (Gallios10, Beckman). And the sample without staining with PI was set as control for gating. The data were analysed with the FlowJo v.10 software and visualised with Prism 9 software.

### Transcriptomics analysis

The bacterial transcriptomic responses caused by the expression of MCR-1 or MCR-3 were analysed by transcriptomics analysis as previously described.^28^ In brief, fresh single colonies of *E. coli* BW25113 carrying *mcr-1*, *mcr-3* or pACYCDuet-1 were inoculated into fresh Luria Bertani broth (LB) containing 30 μg/mL CHL and 0.2% arabinose, and three replicates were carried out for each strain. After induction for 12 h, cultures were harvested by centrifugation (1503 g for 3 min) and cell pellets were collected. The samples were sent for Magigene Co. (Guangzhou, China) for RNA sample preparation and transcriptomic analysis. Briefly, the cell pellets were firstly resuspended in RNAprotect (Qiagen), followed by centrifugation at 13523*g*, 4 °C for 10 min. Next, RNA was extracted from the cell pellet using a RNeasy Mini Kit (Qiagen, cat#74904, China), according to the manufacturer's instructions. The quality and quantity of the purified RNA were checked using Nanodrop (Thermo Fisher Scientific). RNase-free DNase (Qiagen, cat#79254, China) was added to remove DNA contamination. All samples were subjected to RNA-Seq using the Illumina HiSeq System (Illumina Inc). Data were employed to assemble the transcriptome using Trinity RNA-Seq software, and RNA-Seq reads were aligned according to the genome sequences of *E. coli* K-12 (NC_010473.1) using the Subread aligner. A volcano map was constructed with R studio (version 3.6.1) to show the comprehensive differences between *mcr-1*-positve cells and *mcr-3*-positive cells The differential transcription genes were mapped to KEGG database (http://geneontology.org/) and gene ontology (GO) terms (with Blast2GO software) for enrichment analysis, and the results were plotted by R studio (version 3.6.1). Also, proteins encoded by studied genes were performed for protein–protein interaction (PPI) analysis through STRING (http://string-db.org/), and the result was plotted by Cytoscape software.

### Western blot analysis

The expression levels of PbgA-HA and LPS of *E. coli* BW25113 carrying *mcr-1*, *mcr-3* or pACYCDuet-1 were verified by western blotting. Similar as previous research,^9^ fresh single colonies of target strains were inoculated into 5 mL fresh Luria Bertani broth (LB) containing 30 μg/mL CHL and 0.2% arabinose. Exponential phase cultures were collected and harvested by centrifugation (376 g for 3 min). 1 mL RIPA Lysis Buffer (Beyotime, cat#P0013, China) was added to resuspended the cell pellet, following by further breaking down the bacillus with an ultrasonic processor (SONICS, VCX 130). After centrifugation (9391 g for 1 min), the protein concentration of each sample was quantified by using the Bradford Protein Assay Kit (Beyotime, cat#P0012, China), according to manufacturers’ protocol. Samples were standardised based on protein concentration and mixed with SDS-PAGE Sample Loading Buffer (Beyotime, cat#P0286, China), followed by electrophoresis on a 12% Bis-Tris SDS-polyacrylamide gel (Epizyme, cat#PG113, China). Next, proteins and LPS were transferred to a polyvinylidene difluoride (PVDF) membrane (Thermo fisher) by using a Trans-Blot Turbo Transfer System (BIO RAD). Primary antibodies detecting RpoB (BioLegend, cat#663006, RRID: AB_2565555, USA), LPS core (Hycult Biotech, cat#HM6011, RRID: AB_2750644, Netherlands) and HA-tag (Cell Signalling, cat#3724, RRID: AB_1549585, USA) were used with a dilution of 1:100,000, 1:1000 and 1:10,000, respectively. Goat anti-rabbit horseradish peroxidase (HRP) conjugate (Zen Bioscience, cat#550010, China) and rabbit anti-mouse HRP conjugate (Dingguo Biology, cat#IH-0011, China) secondary antibodies were each used at a 1:10,000 dilution. After processing of chemiluminescent detection with a ChemiDoc Touch Imaging System (BIO RAD), the protein levels were quantified with Fuji software, where the expression level of RpoB was regarded as reference. Three replicates were performed for each sample.

### Quantitative real-time PCR

To verify the change of transcriptional level of genes related with glutamate-dependent acid stress response pathway, the mRNA of *E. coli* BW25113 harbouring *mcr-1*, *mcr-3* or pACYCDuet-1 were extracted for quantitative real-time PCR (q-PCR). Overnight cultures of target strains were sub-cultured into 1 mL fresh LB broth containing 30 μg/mL CHL and 0.2% arabinose. Exponential phase cultures were collected and harvested by centrifugation (376 g for 3 min). After removal of the supernatant, the cell pellet was resuspended in RNA-easy Isolation Reagent (Vazyme, cat#R403, China). Total RNA was precipitated by the supplement of isopropanol and collected by centrifugation (4 °C, 13523 g for 10 min), the supernatant was discarded, and the mRNA pellet was washed with 75% ethanol. The mRNA pellet was dehydrated through air dry and dissolved in RNase-free H_2_O. The contaminating genomic DNA was digested with gDNA wiper Mix (Vazyme, cat#R423, China). The cDNA was prepared from purified mRNA with HiScriptII qRT SuperMix II (Vazyme, cat#R423, China) through reverse transcription. The cDNA levels of target genes were then quantified by quantitative real-time PCR (qRT-PCR) on a CFX96 cycler (BIO RAD) by using AceQ Universal SYBR qPCR Master Mix (Vazyme, cat#Q713, China), according to manufacturers’ protocol. All qPCR primers were determined to be >95% efficient, and the cDNA masses tested were experimentally validated to be within the linear dynamic range of the assay. Signals were normalised to those of the transcript of housekeeping gene *rpoB* and quantified with the ΔΔCT analysis. Error bars are 95% confidence intervals of the three technical replicates.

### Phylogenetic analysis of *mcr* family

The DNA sequence of *mcr* family genes were downloaded from NCBI database, which includes *mc-1* (NG_050417.1), *mcr-2* (NG_051171.1), *mcr-3* (NG_055505.1), *mcr-4* (NG_057470.1), *mcr-5* (NG_055658.1) and *mcr-8* (NG_061399.1). Draft DNA sequence of *mcr* family genes were aligned and then applied to phylogenetic tree construction and visualisation using MEGA (version 11) with neighbour-joining (NJ) method.

### Codon adaptation index (CAI) calculation

CAI values of each gene of *mcr* family were calculated using the equation as described followed:

$$\text{CAI}={\left(\prod_{i=1}^{L}w_{i}\right)}^{1/L}$$

where “L” is the length of the gene in the number of codons. For a codon “i”, *w*_*i*_ represents its codon weightage which is calculated based on the observed frequency of that codon relative to the frequencies of all its synonymous codons from a reference set of highly expressed genes.

$$w_{i}=\frac{f_{i}}{\max\left(f_{i}\right)}$$

where *f*_*i*_ is the observed frequency of the codon and max (*f*_*i*_) is the maximum observed frequency among all its synonymous codons in the reference set.

The clustered analysis and correlation analysis were performed by using SPSS (version 29.0.1.0) with Hierarchical cluster method.

### Molecular dynamics simulation and free energy analysis

The complex structure of MCR-3 and lipid A was modelled using the advanced homology modelling module^29^ in the Schrödinger software package, based on the binding mode of MCR-1 and lipid A reported in our previous work, and the modelled complex structure was globally optimised using the Prime module to obtain a more reliable initial structure for molecular dynamics simulation. Three independent simulations for per protein with length of 3000 ns were performed by using Desmond^30^ of Schrödinger2022-3 under the Charmm36 m^31^ force field. The POPE membrane was assembled to proteins by referring to the membrane positions of MCR-1. The complexed structures were explicitly solvated with TIP3P water molecules under cubic periodic boundary conditions for 15 Å buffer region. The overlapping water molecules are deleted and 0.15 M NaCl are added, and the systems were neutralised by adding Na^+^ as counter ions. Brownian motion simulation was used to relax these systems into local energy minimum states separately. An ensemble (NPT) was then applied to maintain the constant temperature (310 K) and pressure (1. 01325 bar) of the systems, and the simulations start with two random initial velocity. The simulations described above were performed using the AutoMD (https://github.com/Wang-Lin-boop/AutoMD) script to handle the system and control the simulation process. This script utilised Vippar to parameterise the force field for the system and generated input files for the Desmond simulation.

The generated trajectories were analysed using the AutoTRJ (https://github.com/Wang-Lin-boop/AutoMD/blob/main/AutoTRJ) script, which merged different replicas of the same system, treated periodic boundary conditions, and performed various analyses. In this study, we performed principal component analysis on the trajectories and analysed the free energy landscape using the sham^32^, ^33^, ^34^ Programme in the GROMACS software package. We extracted a conformation of lipid A from RCSB (https://www.rcsb.org/) (PDB code: 5IJD) for molecular docking simulation. For each system, we used an affinity propagation^35^ method to cluster the trajectories, obtaining the representative conformation of MCR-3-lipid A.

### Virtual screening of MCR inhibitors

Based on the MD trajectories and the MCR-1-lipid A complex structure reported in our previous work, we obtained 60 representative conformations from six MD trajectories using affinity propagation clustering^35^ and hierarchical clustering.^36^ Subsequently, we used cross-docking to dock the probe molecules of Glide into the putative lipid A binding pocket of MCR-1, and inspected visually for the three optimal ligand binding conformations.

The cross-docking was performed using XDock (https://github.com/Wang-Lin-boop/Schrodinger-Script/blob/main/XDock) script with default settings. We then generated docking grids using Glide^37^ module of Schrödinger2021-1 with a centre box size of 12 Å and accepted aromatic hydrogens and halogens as hydrogen bond donors. The van der Waals radius of the receptor was set to 0.9, and the hydroxyl group of the T112 residue was set as a rotatable group. We applied the advance mode of GVSrun (https://github.com/Wang-Lin-boop/Schrodinger-Script/blob/main/GVSrun) script to screen the Specs (https://www.specs.net/) compound database. In this mode, all ligands in the database were first filtered by a fast docking with HTVS precision, and the top 5% of ligands were retained for further docking with standard precision (SP). During docking, the ligand strain energy was added for scoring correction, and intramolecular hydrogen bonds were rewarded. The top 4000 compounds were selected for 2-fold enhanced sampling and the number of ligand conformations generated was increased to 10,000. A re-docking was performed with SP to obtain more accurate binding conformations, which were re-ranked. All compounds were inspected visually to identify potential active molecules. We selected a total of 200 compounds for Molecular mechanics with generalised Born and surface area solvation (MM/GBSA) binding free energy calculations using Prime module of Schrödinger2022-3. Finally, these potential active molecules were docked into the putative lipid A binding pocket of MCR-3, and compounds that exhibited desirable binding modes for both MCR-1 and MCR-3 were selected.

### *In vitro* inhibition assay of colistin and MCR inhibitors in combination

To evaluate the antibacterial effect of MCR inhibitors in combination with colistin, AlamarBlue reduction assays were performed. Overnight cultures of the mcr-1-positive and mcr-3-positive strains were subcultured in fresh LB broth at a 1:100 ratio and induced with 0.2% arabinose to express MCR-1 and MCR-3, respectively. Exponential-phase cultures (∼1 × 10^8^ CFU/mL) were collected, diluted to approximately 1 × 10^6^ CFU/mL, and dispensed into 96-well microplates to a final volume of 100 μL. Bacteria were exposed to colistin (concentration gradient: 0.25–64 μg/mL), either alone or in combination with three compounds (100 μM), for 16 h. AlamarBlue solution was added to quantify bacterial proliferation, and fluorescence was measured at λ_ex_ = 545 nm and λ_em_ = 595 nm. Calculations:

$$\text{AlamarBlue}\;\text{reduction}\;\text{rate}\;\left(\%\right)=\left[\left(F_{obs}-F_{neg-ctrl}\right)/\left(F_{pos-ctrl}-F_{neg-ctrl}\right)\right]\;\times \;100\text{;}$$

Inhibition rate (%) = 100%- AlamarBlue reduction rate (%); All experiments were performed in triplicate. Data were analysed and plotted using *Prism 9* software.

### Time killing assays of colistin and MCR inhibitors in combination

To assess the bactericidal activity of MCR inhibitors combined with colistin, time-kill assays were conducted. Fresh single colonies of *E. coli* BW25113 carrying *mcr-1* or *mcr-3* were inoculated into LB broth containing 30 μg/mL chloramphenicol and 0.2% arabinose, followed by incubation at 37 °C. Exponential-phase cultures (∼1 × 10^8^ CFU/mL) were aliquoted and treated with colistin (1∗MIC, 16 μg/mL), colistin (1∗MIC, 16 μg/mL) combined with each MCR inhibitor (compound #1–3, 100 μM). DMSO-treated samples served as controls. For each treatment, 100 μl of culture was sampled at 0 h, 4 h, and 8 h, serially diluted in six 10-fold gradients, using saline, and 5 μl of each dilution was spotted onto LB agar plates. After incubation at 37 °C for 18 h, viable colonies, CFUs were counted. All experiments were performed in triplicate. Results were analysed using Prism 9 software.

### Ethics

With the approval from Ethics Committee of Zhongshan School of Medicine on Laboratory Animal Care (reference number: SYSU-IACUC-2022-B0031), Sun Yat-sen University, all the animal experiments were conducted based on the standard of the National Institutes of Health Guide for the Care and Use of Laboratory Animals. This study is reported in accordance with the ARRIVE guidelines 2.0 (https://arriveguidelines.org).

### Statistics

Statistical analysis was performed using Prism (version 9, GraphPad Software). Data were analysed using unpaired *t*-tests for two-group comparisons and one-way ANOVA for three or more groups. A *P* value of 0.05 or less was considered statistically significant. The phylogenetic tree of *mcr* family was generated by using MEGA (version 11). The clustered analysis and correlation analysis were performed by using SPSS (version 29.0.1.0).

### Role of funders

The funders had no role in study design, data collection, data analyses, interpretation, writing, or submission of this manuscript.

## Results

### The global prevalence of *mcr-3* increased continuously from 2005 to 2022

As *mcr-1* and *mcr-3* are the two largest lineages of the *mcr* family, monitoring their dissemination trends is critical for understanding the evolving threat of plasmid-mediated colistin resistance. While our previous study has found that the withdrawal of colistin led to a substantial decline in the prevalence of *mcr-1*-positive bacteria in China,^8^ the epidemiological trajectory of *mcr-3* remains poorly characterised. To investigate the global dynamic change in *mcr-3* prevalence, a systematic review and meta-analysis of epidemiological studies reporting the positive detection of *mcr-3* between 2005 and 2022 were performed. Overall, 112 relevant studies were assessed for eligibility. After screening based on our inclusion and exclusion criteria, 78 studies involving 76436 samples were eligible for quantitative meta-analysis (Figure S2A).

**Fig. 1:**
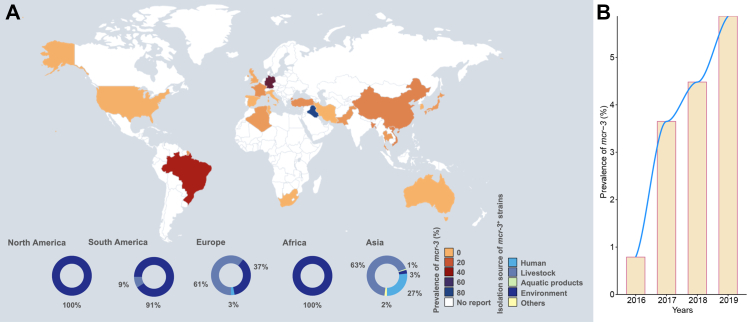
**Global prevalence of *mcr-3***. **(A)** Countries in yellow to blue represent the prevalence of *mcr-3* in these countries from 2005– to 2022, whereas countries in white lack studies. The pie charts represent the sources of *mcr-3*-bearing isolates for each continent. The numbers shown represent the percentages. **(B)** The histogram represents the prevalence of *mcr-3* from 2016 to 2019 on the basis of 1494 samples collected from 8 provinces (Guangdong, Shandong, Shanxi, Anhui, Yunnan, Liaoning, Sichuan and Guangxi) across China.

The global average prevalence of *mcr-3* from 2005 to 2022 was 1.99% (95% CI: 0.77–3.61%, Figure S3). The global distribution of the *mcr-3* gene, in relation to its various sources, revealed significant geographic segregation (Fig. 1A). To date, *mcr-3* has spread widely in 5 out of 7 continents. Eighteen of 23 countries have reported the positive detection of *mcr-3*. The prevalence of *mcr-3* in more than half of these countries (11 of 18) has exceeded 2% (Figure S4). The sampling source was also evaluated. The prevalence of *mcr-3* was highest in the environment (7.21%), followed by other animal sources (3.4%), humans (2.77%), marine products (2.41%) and livestock (1.1%, Figure S5). In our analysis, we found that most cases were detected in Asia. Moreover, sources for isolating *mcr-3*-positive bacteria are highly diverse in Asia (Fig. 1A). A total of 27% of the *mcr-3*-bearing bacteria were isolated from humans, 68% from livestock, 3% from the environment, 2% from other sources and 1% from aquatic products. It was followed by Europe (61% from livestock, 37% from the environment and 3% from humans), South America (91% from the environment and 9% from livestock), North America (100% from the environment) and Africa (100% from the environment). These results indicated that the dissemination of *mcr-3* was dependent on geography and source.

Next, we examined whether the prevalence of *mcr-3* changed over time. Our previous studies discovered a decrease in another major member of the *mcr* family, *mcr-1*, between 2015 and 2019.^8^^,^^38^ Therefore, we examined the prevalence trends before, during, and after this period (2015–2019) by dividing the data into three subgroups. The global prevalence of *mcr-3* has continuously increased since 2015 (Figure S2B and Figure S6). The prevalence of *mcr-3* between 2005 and 2014 was 0.18% (95% CI: 0–0.73%), accounting for 51.51% and 48.48% of the cases from livestock and humans, respectively. The prevalence increased to 2.63% (95% CI: 0.81%–5.16%) between 2015 and 2019, accounting for 68.75%, 24.54%, 4.17%, 1.97%, and 0.58% of the cases from livestock, human, environment, others and aquatic products, respectively. From 2020 to 2022, the prevalence was even higher (3.41%, 95% CI: 0.32%–8.59%; 51.72% from the environment, 27.59% from livestock, 10.34% from humans and 10.34% from aquatic products). Moreover, we statistically measured the potential impact of confounding factors including time, region and source of sampling on the prevalence of *mcr-*3 by fitting these regressors into Bayesian hierarchical model. As shown in Figure S7, most of the sampling regions and all the sampling sources do not have significant impact on *mcr-3* prevalence. Sampling time was found to be significantly related to the increase of *mcr-3* prevalence. Taken together, an unexpected significant increase of *mcr-3* dissemination was discovered across the world along last two decades.

Furthermore, since the withdraw of colistin in China resulted in reduced prevalence of *mcr-1*, we conducted a nationwide prospective epidemiological study to investigate the influence of colistin banning policy upon the *mcr-3* prevalence in China. In total, 1494 nonduplicated samples were collected from eight provinces (Guangdong, Shandong, Shanxi, Anhui, Yunnan, Liaoning, Sichuan and Guangxi) from 2016 to 2019. Among them, 4.28% (64/1494) were *mcr-3* positive when verified by PCR and Sanger sequencing. Notably, the incidence of *mcr-3* increased continuously during these four years (0.79% in 2016, 3.65% in 2017, 4.48% in 2018 and 5.87% in 2019; Fig. 1B). Unlike *mcr-1*, the banning of colistin in husbandry had no impact on the spread of *mcr-3* in China.

In summary, unlike the decreased prevalence of *mcr-1*,^8^^,^^38^, ^39^, ^40^ the global prevalence of *mcr-3* has increased continuously over the past decade. Crucially, the banning policy of colistin in husbandry has failed to prevent the continuous dissemination of *mcr-3* in China.

### *mcr-3*-positive *E. coli* displayed greater fitness than the *mcr-1*-positive strain both *in vitro* and *in vivo*

**Fig. 2:**
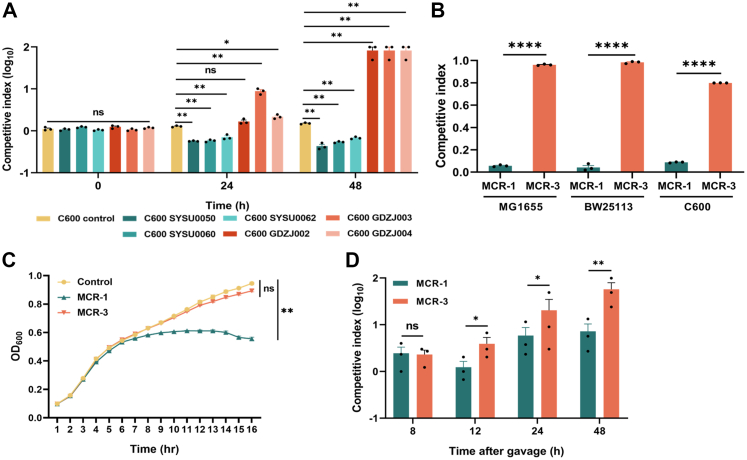
**Fitness comparison between *mcr-1*- and *mcr-3*-bearing *E. coli***. **(A)** Fitness evaluation of *E. coli* C600 conjugants carrying *mcr-1-* or *mcr-3*-bearing plasmids through an *in vitro* competitive assay. The indicated plasmids were isolated from human/animal faecal samples. C600 SYSU0050, C600 SYSU0060 and C600 SYSU0062 were *mcr-1*-bearing strains, whereas C600 GDZJ002, C600 GDZJ003 and C600 GDZJ004 were *mcr-3*-positive strains. The cultures of the indicated strains and the GFP-positive control strain were mixed at a ratio of 1:1. Samples were collected at 0 h, 24 h and 48 h after initiation. The percentage of target strains was determined by flow cytometry and analysed by FlowJo (version 10) software. The y-axis represents the log_10_ value of the percentage of the GFP-negative population/percentage of the GFP-positive population, and the x-axis represents the period of growth (h). **(B)** Fitness evaluation of *E. coli* K-12 strains (MG1655, BW25113 and C600) carrying *mcr-1* or *mcr-3* through an *in vitro* competitive assay. The cultures of the indicated strains and the GFP-positive control strain were mixed at a ratio of 1:1. Samples were collected at 0 h (T0) and 24 h (Tn) after initiation. The percentage of target strains was determined by flow cytometry and analysed by FlowJo (version 10) software. The y-axis represents the percentage of the GFP-negative population at Tn/percentage of the GFP-negative population at T0. **(C)** Measurement of growth curves of *E. coli* BW25113 carrying empty vector, *mcr-1* or *mcr-3*. The y-axis shows the optical density at a wavelength of 600 nm (OD_600_) of the broth cultures, and the x-axis shows the period of growth (h). **(D)** Evaluation of the fitness of *E. coli* C600 bearing *mcr-1* or *mcr-3* through an *in vivo* competitive assay. The cultures of the indicated strains and the GFP-positive control strain were mixed at a ratio of 1:1. The mice were orally gavaged with a 100 μl mixture (10^8^ CFUs). Samples were collected at 0 h, 8 h, 24 h and 48 h after initiation. The percentage of target strains was then determined through a CFU assay. The y-axis represents the log_10_ value of the percentage of the GFP-negative population/percentage of the GFP-positive population, and the x-axis represents the period of growth (h). All the experiments described above were performed three times with similar results. The error bars indicate the standard errors of the means (SEMs) for three biological replicates. A two-tailed unpaired *t* test was performed to determine the statistical significance of the data. ns, no significant difference; ∗, *P* < 0.1; ∗∗, *P* < 0.01; ∗∗∗∗, *P* < 0.0001.

The MCR-1-mediated fitness cost has resulted in a dramatically reduced prevalence of *mcr-1* after the withdrawal of colistin from animal fodder in 2017.^11^^,^^41^ Unlike *mcr-1*, the results of an epidemiological study revealed that the prevalence of *mcr-3* had the opposite pattern to that of *mcr-1*, which suggested that the expression of *mcr-3* might confer no biological burden upon the bacterial host. To test this hypothesis, through an *in vitro* competitive assay, we monitored the fitness of conjugants harbouring *mcr-1*- or *mcr-3*-positive plasmids in the background of *E. coli*, which is one of the main host strains of *mcr-1* and *mcr-3*.^13^^,^^17^^,^^18^^,^^24^^,^^42^ The cultures of the bacteria with the empty control plasmid harbouring *gfp* and the target strains were initially mixed at a ratio of 1:1. All the *mcr-1*-positive strains presented a competitive index <1 at 24 h and 48 h after initiation, whereas the values for the *mcr-3*-positive strains were always >1 at the same time points (Fig. 2A). This result indicated that the expression of MCR-3 placed no biological burden on the bacterial host, which was confirmed by the use of *E. coli* K-12 laboratory-adapted strains (Fig. 2B). Both *mcr-1* and *mcr-3* were cloned, inserted into the medium-copy plasmid pACYCDuet-1 and regulated by an arabinose-inducible promoter.^11^ Moreover, since the colistin minimum inhibitory concentrations (MICs) of *mcr-1*- or *mcr-3*-positive *E. coli* BW25113 were comparable (Figure S8A), the fitness of the indicated strains challenged with colistin at sublethal concentrations was estimated (Figure S8B). The cultures of the indicated strains were initially mixed at a ratio of 1:1, and the percentage of *mcr-3*-bearing cells increased to >99% after 24 h and 48 h. As the quality strains, the MICs of *E. coli* ATCC 25922 and BW25113 also be determined (Table S1). Moreover, by measuring the growth curves, significant growth arrest was observed for *mcr-1*-expressing *E. coli* but not for *mcr-3*-positive strain when compared to control strain (Fig. 2C). Therefore, these results confirmed that the expression of *mcr-3* imposed a lower fitness cost than *mcr-1* did, even under the stress of colistin.

To further verify whether the low fitness burden of *mcr-3*-positive *E. coli* was exclusively an *in vitro* phenomenon, we carried out a competitive assay on a mouse model of intestinal tract colonisation. The mice were inoculated through oral gavage with a mixture of 1 × 10^8^ CFUs, which contained control (carrying *kan*^*R*^) and *mcr-1*- or *mcr-3*-positive strains at a ratio of 1:1. Intriguingly, compared with *mcr-1*-positive cells, more *mcr-3*-positive cells were isolated from the faecal sample (Fig. 2D). The *mcr-3*-positive *E. coli* had average *in vivo* competitive indices of 2.4, 20.3 and 57.3 at 8, 24 and 48 h after gavage, respectively, whereas the values were 2.3, 5.8 and 10.7 for the *mcr-1*-expressing strain, respectively. Thus, these results suggested that *mcr-3*-positive cells might display greater fitness than *mcr-1*-positive cells in the native environment of the animal intestinal tract.

Taken together, these results confirmed that *mcr-3*-bearing *E. coli* exhibited greater fitness than *mcr-1*-bearing *E. coli* both *in vitro* and *in vivo*.

### MCR-3-positive *E. coli* exhibited intact membrane integrity

**Fig. 3:**
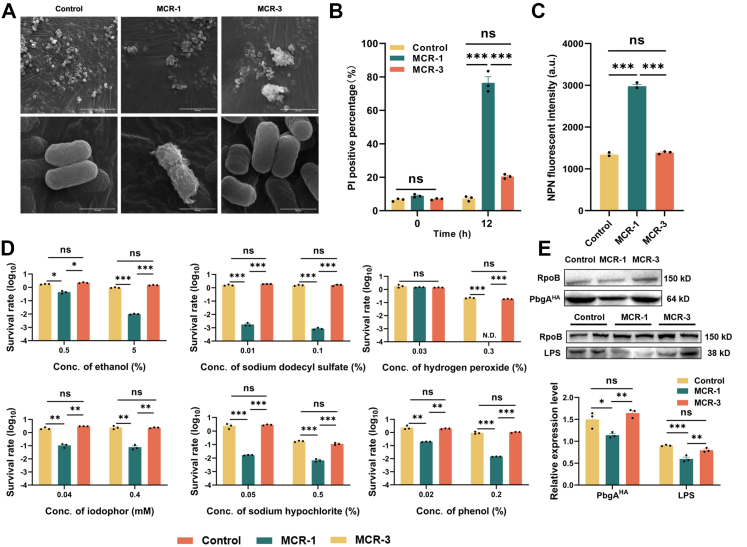
**The expression of MCR-3 had no effect on membrane homoeostasis**. **(A)** SEM micrographs of *E. coli* BW25113 carrying the empty vector, *mcr-1* or *mcr-3*. Overnight cultures of the above strains were subcultured into fresh LB broth at a ratio of 1:100 and induced with 0.2% arabinose. The logarithmic phase cultures were collected for sample preparation. **(B)** Determination of inner membrane integrity. The inner membrane permeability of *E. coli* carrying *mcr-1* or *mcr-3* was evaluated by a PI staining assay. Overnight cultures were subcultured into fresh LB broth at a ratio of 1:100 and induced with 0.2% arabinose to express MCR-1 or MCR-3. Samples were collected at 0 h and 12 h after initiation, followed by staining with PI dye for 15 min. **(C)** Determination of outer membrane integrity. The outer membrane permeability of *mcr-1-* or *mcr-3*-bearing strains was evaluated by measuring the NPN uptake of logarithmic phase cultures. **(D)** Susceptibility of *mcr-1* or *mcr-3*-harbouring strains to disinfectants. The viability of MCR-1/MCR-3-expressing strains stressed with ethanol (0.5% and 5%), sodium dodecyl sulphate (0.01% and 0.1%), hydrogen peroxide (0.03% and 0.3%), sodium hypochlorite (0.05% and 0.5%), iodophor (0.04 mM and 0.4 mM) and phenol (0.02% and 0.2%). A CFU assay was carried out to determine the survival rate at 4 h after initiation (Tn). The y-axis represents the log_10_ value of the viability at Tn/viability at T0, and the x-axis represents the concentration of the reagents. **(E)** Determination of the intracellular expression levels of PbgA^HA^ and LPS in *E. coli* BW25113 carrying the empty vector, *mcr-1* or *mcr-*3 by western blotting. All the experiments described above were performed three times with similar results. The error bars indicate the standard errors of the means (SEMs) for three biological replicates. A two-tailed unpaired *t* test and one-way ANOVA analysis were performed to determine the statistical significance of the data. ns, no significant difference; ∗, *P* < 0.1; ∗∗, *P* < 0.01; ∗∗∗, *P* < 0.001.

Our previous research demonstrated that the expression of MCR-1 significantly perturbs the homoeostasis of bacterial membranes and causes peptidoglycan (PG) layer remodelling, which subsequently increases membrane permeability and facilitates cell death.^9^^,^^10^ We thus wondered whether the expression of MCR-3 had any influence on bacterial membrane construction. To test this hypothesis, we observed the morphological features of the indicated strains by scanning electron microscopy (SEM). As expected, wrinkles and breakages were observed on the surface of the *mcr-1*-positive cells, whereas the membranes of the *mcr-3*-positive cells and the control were intact and smooth (Fig. 3A). Although our previous study revealed that the expression of MCR-1 resulted in severe cytoplasmic shrinkage,^27^ we found that no shrinkage was observed in the stationary culture of *mcr-3*-positive *E. coli* (Figure S9). Taken together, these results suggested that the expression of MCR-3 had no significant influence on the morphology of membrane surface.

To characterise the membrane integrity of *mcr-3*-positive cells fully, we monitored the growth of the indicated strains in the presence of the anionic detergent SDS and EDTA (Figure S10). In particular, the susceptibilities of *mcr-3*-positive cells and control cells to SDS-EDTA were comparable, whereas *mcr-1*-expressing *E. coli* were sensitive to this reagent. We next assessed the effect of MCR-3 on the integrity of the bacterial inner membrane with propidium iodide (PI) dye, which has to the cross compromised membranes to bind to DNA and RNA in damaged cells. As expected, the expression of MCR-1 resulted in a strong PI signal for *E. coli* (78% PI-positive population), whereas the percentage of the PI-positive population (21%) in *mcr-3*-positive cells was significantly lower (Fig. 3B). We also performed a 1-N-phenylnaphthylamine (NPN) staining assay to investigate the permeability of the outer membrane. NPN is a type of probe that eliminates strong fluorescent signals upon contact with phospholipids exposed by damage to the LPS monolayer.^16^ The expression of MCR-1 caused permeabilisation of the outer membrane, but the NPN signal of the *mcr-3*-positive cells was comparable to that of the control (Fig. 3C). Overall, the expression of MCR-3 caused no significant damage to the inner or outer membrane of the bacterial host. Since the membrane structure of the *mcr-3*-positive strain was more intact than that of the strain expressing MCR-1, the *mcr-3*-positive strain displayed greater viability than did the *mcr-1*-positive *E. coli* strain under the treatment of disinfectants commonly used in the clinic (Fig. 3D).

We next performed whole-transcriptome analysis to further profile the transcriptomic changes in the *mcr-1*- or *mcr-3*-bearing strains. In total, 4206 genes were identified, representing 89.5% coverage of the *E. coli* BW25113 genome. Unexpectedly, among the top enriched processes revealed by KEGG enrichment analysis (Table S2), the mRNAs of *gad* family genes, which control the pathway of the glutamate-GABA-dependent acid stress response,^43^^,^^44^ were markedly upregulated in the *mcr-3*-positive strain, whereas the capsule synthesis-related genes (*wca* family) presented increased transcriptional levels in *mcr-1*-bearing *E. coli* (Figure S11A, Table S3).^45^^,^^46^ However, there was no significant difference in the transcriptional level of these two pathways between *mcr-3*-bearing *E. coli* and the control (Figure S11B). These findings indicated that the transcriptional level of the glutamate-GABA acid stress response pathway was repressed in the *mcr-1*-expressing strain, but the capsule formation pathway was activated. This result was further validated by q‒PCR (Figure S11C). Consistent with the results of the transcriptomics analysis, both the control and *mcr-3*-expressing strains presented greater viability than did the *mcr-1*-positive strain when challenged with LB medium at pH = 4.0 (Figure S11D). Moreover, scanning electron microscopy (SEM) revealed that extracellular matrix-encapsulated cells were present in the *mcr-1*-bearing strain but not in the *mcr-3*-positive *E. coli* strain (Figure S11E and F). Increased transcriptional levels of RscA were observed in *mcr-1*-bearing *E. coli*. RcsA is an activator of the colanic acid capsular polysaccharide synthesis pathway^47^ and a repressor of the GABA-glutamate-dependent acid resistance system.^48^ A previous study revealed that a reduction in the LPS level of the outer membrane significantly increased the expression of RcsA.^49^^,^^50^ Indeed, the expression levels of LPS and PbgA, an LPS sensor protein anchored in the inner membrane,^51^ in *mcr-3*-positive *E. coli* were comparable to those in the control, which were both greater than those in *mcr-1*-positive cells (Fig. 3E). In summary, the expression of MCR-3 had no effect on the LPS level of the bacterial host, whereas the reduced LPS level in *mcr-1*-positive *E. coli* activated RcsA and weakened adaptive ability in acidic environments, such as the human digestive tract.

Overall, our results revealed that the expression of MCR-3 had no effect on the homoeostasis of the membrane LPS level or damage the integrity of the bacterial membrane.

### 5′-end codons with a low usage frequency guaranteed a low fitness cost for *mcr-3*-bearing *E. coli*

In our previous study, we identified a putative lipid A binding pocket localised at the transmembrane region of MCR-1, which governs the colistin resistance, membrane integrity and fitness of the bacterial host.^9^ Although it has been reported that the catalytic domain of MCR-3 is highly similar with that of MCR-1,^24^ whether such putative lipid A binding pocket also plays crucial role in the enzymatic activity of MCR-3 remains unknown. To this end, we compared the differences between MCR-1 and MCR-3 in terms of the lipid A binding site, binding pose, and interaction energy with lipid A. By exploring the free energy landscape of MCR-1, we identified the stable global minimal state and high-energy local minimal state of the protein (Figure S12A). However, unlike MCR-1, MCR-3 exhibited multiple local minimal states, among which a global minimal state was generated, which suggested the inherent high flexibility and instability of the MCR-3 protein (Figure S12B). We selected the global minimal state of MCR-1 and MCR-3 for further analysis, similar to the lipid A binding mode of MCR-1, a putative lipid A binding pocket was verified at the linker domain of MCR-3 (Figure S12C). The head of lipid A formed stable hydrogen bond interactions with R180, R187, and K200 of MCR-3, which was similar to the lipid A binding mode of MCR-1. In particular, by analysing the frequency of interface interactions between MCR-1/MCR-3 and lipid A (Figure S12D), we found that MCR-3 generated a hydrogen bond network across the interaction interface on the transmembrane domain, which might account for the enhanced lipid A affinity of MCR-3 compared with that of MCR-1 (Figure S12E). Moreover, to validate the computational predictions and analyses described above, we introduced a series of mutations into the lipid A binding pocket of MCR-3 (Figure S12F). Among them, mutations such as R180A, R187A and N184A were expected to disrupt the polar interactions between the heads of lipid A and MCR-3, increasing the susceptibility of the bacterial host to colistin. In summary, the putative lipid A binding pockets of MCR-1 and MCR-3 were highly comparable, indicating strong structural similarity between the two proteins. Hence, the distinct fitness costs associated with MCR-1 and MCR-3 are unlikely to be attributed to unique structural features of MCR-3.

**Fig. 4:**
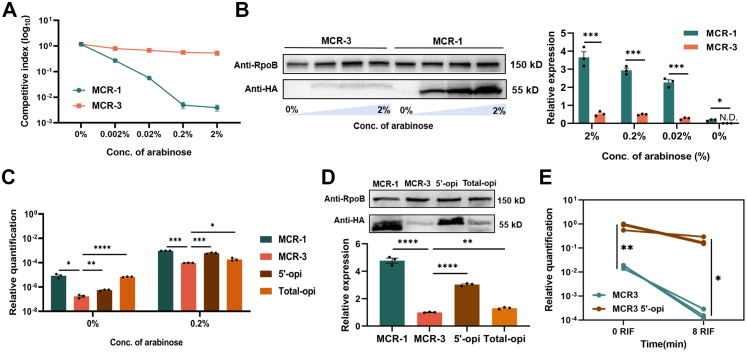
**The codon optimality of the 5′-coding region determined the fitness cost conferred by MCR-1 and MCR-3**. **(A)** Evaluation of fitness of *E. coli* BW25113 carrying *mcr-1* or *mcr-3* through an *in vitro* competitive assay. The expression of MCR-1 or MCR-3 was induced by arabinose at a series of concentrations. Cultures of the indicated strains and the GFP-positive control strain were mixed at a ratio of 1:1. Samples were collected at 0 h (T0) and 24 h (Tn) after initiation. The percentage of target strains was determined by flow cytometry and analysed by FlowJo (version 10) software. The y-axis represents the log_10_ value of the percentage of the GFP-negative population at Tn/percentage of the GFP-negative population at T0, and the x-axis represents the concentration of arabinose. **(B)** Determination of the protein expression levels of MCR-1 or MCR-3 in *E. coli* BW25113 through western blotting. An HA tag was added at the C-terminus of the indicated proteins. The expression of MCR-1 or MCR-3 was induced by arabinose at a series of concentrations (0%, 0.02%, 0.2% and 2%). **(C)** The mRNA transcript levels of *mcr-1*, *mcr-3* and their variants in *E. coli* BW25113 were determined by q‒PCR, which was normalised to the transcript level of the housekeeping gene *rpoB* and quantified by ΔΔCT analysis. **(D)** Determination of the protein expression levels of MCR-1, MCR-3 and their variants in *E. coli* BW25113 through western blotting. An HA tag was added at the C-terminus of the indicated proteins. **(E)** Evaluation of mRNA stability of *mcr-3* and its variants in *E. coli* BW25113. Overnight cultures were subcultured into fresh LB broth at a ratio of 1:100 and induced with 0.2% arabinose to express the indicated genes. Next, when the OD_600_ of the cultures reached 0.6, the activity of RNase E in the indicated strains was repressed by treatment with 500 μg/mL rifampicin for 8 min. Samples before and after rifampicin treatment were collected, and the mRNA levels of the indicated genes were determined by q‒PCR, which was normalised to the transcript level of the housekeeping gene *rpoB* and quantified by ΔΔCT analysis. All the experiments described above were performed three times with similar results. The error bars indicate the standard errors of the means (SEMs) for three biological replicates. A two-tailed unpaired *t* test and one-way ANOVA analysis were performed to determine the statistical significance of the data. ∗, *P* < 0.1; ∗∗, *P* < 0.01; ∗∗∗, *P* < 0.001; ∗∗∗∗, *P* < 0.0001. N.D., not detected. 5′-opi represents the *mcr-3* variant with codon optimisation at the first 30 bp fragment of the gene, and total-opi represents the *mcr-3* variant with codon optimisation at the remaining fragment of the gene.

Previous studies have shown that excessive expression of intramembrane proteins always has deleterious effects on fitness.^52^^,^^53^ Hence, we assumed that the varied fitness observed in *mcr-1*- and *mcr-3*-bearing *E. coli* might be related to the expression of the indicated proteins. To test this hypothesis, we estimated the fitness of *mcr-1*- or *mcr-3*-bearing *E. coli* and the cellular expression of MCR-1/MCR-3 by induction with arabinose at different concentrations (Fig. 4A and B). The results revealed that the fitness of the *mcr-1*-positive strain decreased with increasing arabinose concentration, which was accompanied by an increase in protein expression. Unexpectedly, the fitness of the *mcr-3*-positive strain was significantly higher than that of the *mcr-1*-bearing strain, while the protein expression level of MCR-3 was lower than that of MCR-1. Through immunofluorescence staining, we found that, compared with that of MCR-1, the Cy3 fluorescent signal of MCR-3 was weaker in *E. coli* (Figure S13). This phenotype was further confirmed by regulating the expression of MCR-1/MCR-3 with their native promoters (Figure S14A). These results established that the low fitness cost of *mcr-3*-bearing *E. coli* was significantly correlated with low protein expression levels.

In addition to the protein expression level, the mRNA transcription level of *mcr-3* was significantly lower than that of *mcr-1* in *E. coli* (Figure S14B). The amount of cellular mRNA is governed by the balance between mRNA production and mRNA decay,^54^ which determines subsequent protein expression.^55^^,^^56^ Transcriptional initiation is mainly determined by the activation of promoters. Indeed, the expression of both *mcr-1* and *mcr-3* was regulated by the arabinose promoter in this study. Hence, it is reasonable to believe that the transcriptional levels of *mcr-1* and *mcr-3* are impacted mainly by mRNA degradation. Several factors influence mRNA stability, including mRNA secondary structure and codon adaptation index. When predicting the mRNA secondary structures of *mcr-1* and *mcr-3* using RNAFold, we found that *mcr-1* forms a more stable mRNA secondary structure than *mcr*-3 (Figure S15 and Table S4). However, since stable mRNA secondary structures can reduce translational efficiency, these results suggest that mRNA secondary structure is not the determining factor for the differential expression levels between MCR-1 and MCR-3. Previous studies have demonstrated that codon usage bias is a major determinant of mRNA stability,^57^ and the introduction of rare codons results in mRNA destabilisation.^58^ By comparing the codon adaptation indices (CAIs) of *mcr-1* and *mcr-3*, we found that these two genes revealed similar codon usage biases in *E. coli* (Figure S16A). Since the nature of the mRNA 5′-end greatly impacts transcript stability in bacteria,^59^ we further calculated the CAI of the first 30 bp fragment of *mcr-1* and *mcr-3*. The 5′-end of *mcr-3* revealed more significant codon bias than did that of *mcr-1* (Figure S16A), which was rich in codons with a low usage frequency (Figure S16B). We therefore assumed that codons with a low usage frequency clustered at the 5′-end of *mcr-3* might be responsible for the reduced mRNA stability. To test this hypothesis, we generated two *mcr-3* variants with codon optimisation at different segments: the first 30 bp fragment (5′-opi) and the rest of the gene (total-opi, Figure S16C). After induction with 0.2% arabinose, the transcriptional level of 5′-opi was 4.6-fold greater than that of WT *mcr-3*, which was accompanied by a 6.3-fold increase in the protein expression level (Fig. 4C and D). However, the mRNA and protein expression levels of total-opi were comparable to those of WT *mcr-3*, demonstrating that the codon usage frequency of the 5′-end of the mRNA significantly effects cellular mRNA and protein levels. Furthermore, with increasing protein expression levels of 5′-opi, the colistin MIC for 5′-opi-expressing *E. coli* (32 μg/mL) was twice that of the *mcr*-3-bearing strain (16 μg/mL, Table S5). These findings indicated that the protein expression level of MCR-3 is a crucial determinant of colistin resistance in host bacteria.

Next, to further estimate the impact of 5′-end codon bias on mRNA stability, the transcript levels of *mcr-3* and 5′-opi were measured after treatment with 500 μg/mL rifampicin. Rifampicin is an inhibitor of bacterial RNA polymerase. By inhibiting the production of mRNA, rifampicin treatment enables the evaluation of the mRNA degradation rate.^60^ In line with our hypothesis, the 5′-end codons optimisation increased the mRNA stability of *mcr-3* (Fig. 4E). The mRNA decay pathway is controlled mainly by the RNase E-dependent ribonuclease in *E. coli*.^61^ The overexpression of RraA, an inhibitor of RNase E,^62^^,^^63^ significantly reduced the difference of mRNA level and protein expression level between *mcr-1* and *mcr-3* (Figure S17). Moreover, both the growth rate and fitness of *E. coli* harbouring 5′-opi were significantly lower than those of the *mcr-3*-bearing strain (Figure S18A and B). The increased expression of MCR-3 enhanced the membrane permeabilisation of the bacterial host (Figure S18C), which indicated that increasing the protein expression level of MCR-3 had a deleterious effect on bacterial fitness by damaging membrane integrity.

In summary, codons with a low usage frequency clustered at the 5′-end of *mcr-3* reduced mRNA stability, which further resulted in a reduced level of protein expression. This feature appeared as a critical factor that guarantees the low fitness cost of *mcr-3*-bearing *E. coli*.

### The codon usage bias at the mRNA 5′-end was closely linked to the evolution of the *mcr* family

**Fig. 5:**
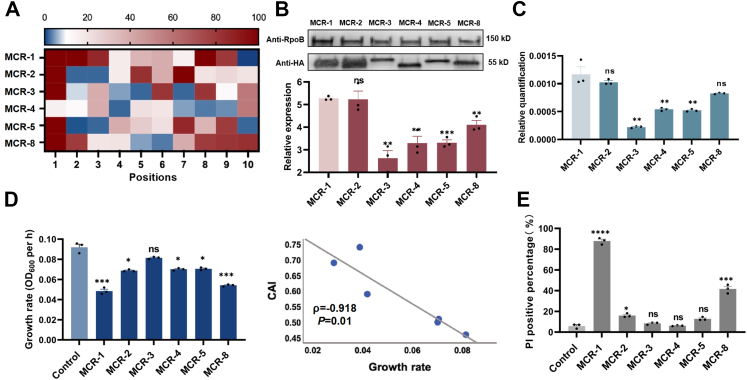
**Codon optimality of the 5′-coding region determined the fitness of *mcr*-positive *E. coli***. **(A)** Codon usage frequency of the first 30 bp fragment of the *mcr* family genes. **(B)** Protein expression levels of the *mcr* genes. The protein expression level was determined by western blotting. An HA tag was added at the C-terminus of the indicated proteins. **(C)** mRNA transcript levels of the *mcr* genes. The mRNA transcript level was determined by q‒PCR, which was normalised to the transcript level of the housekeeping gene *rpoB* and quantified by ΔΔCT analysis. **(D)** Growth rate of *E. coli* BW25113 bearing *mcr* genes during the logarithmic phase (left) and correlation with the 5′-end codon adaptation index (right) of the indicated genes. ρ is Spearman's rank correlation coefficient. The related *P* values and regression lines are shown. **(E)** Determination of inner membrane integrity. The inner membrane permeability of *E. coli* carrying the *mcr* genes were evaluated by a PI staining assay. Overnight cultures were subcultured into fresh LB broth at a ratio of 1:100 and induced with 0.2% arabinose to express the MCR proteins. Samples were collected at 0 h and 12 h after initiation, followed by staining with PI dye for 15 min. The proportion of PI-positive cells was determined by flow cytometry and analysed by FlowJo (version 10) software. All the experiments described above were performed three times with similar results. The error bars indicate the standard errors of the means (SEMs) for three biological replicates. A two-tailed unpaired *t* test was performed to determine the statistical significance of the data. ns, no significant difference; ∗, *P* < 0.1; ∗∗, *P* < 0.01; ∗∗∗, *P* < 0.001; ∗∗∗∗, *P* < 0.0001.

Since 5′-end codon usage frequency affected the fitness cost of *mcr-1*- and *mcr-3*-bearing strains, we wondered whether this feature could also impact the fitness cost induced by MCR family members. To this end, we analysed the codon usage frequency and CAI of the first 30 bp fragment among *mcr-1*, *mcr-2*, *mcr-3*, *mcr-4*, *mcr-5* and *mcr-8*, while *mcr-6*, *mcr-7*, *mcr-9* and *mcr-10* were excluded, since *E. coli* is not the main host mediating the spread of these genes.^13^ Compared with those of *mcr-1* and *mcr-2*, more codons with a low usage frequency were enriched at the 5′-ends of *mcr-3*, *mcr-4*, *mcr-5* and *mcr-8* (Fig. 5A). The CAIs of *mcr-2* and *mcr-1* were similar and were both higher than those of the remaining *mcr* family members. Moreover, in line with the feature of the 5′-end codon bias, the protein and mRNA expression levels of *mcr-2* and *mcr-1* were similar, whereas those of *mcr-3*, *mcr-4*, *mcr-5* and *mcr-8* were significantly reduced (Fig. 5B and C). Notably, the 5′-end CAI values of the *mcr* family members were highly correlated with their mRNA level (ρ = 0.962, *P* = 0.002) and protein expression level (ρ = 0.985, *P* < 0.001; Figure S19) in *E. coli*. These results illustrated that the 5′-end codon bias of *mcr* genes was a crucial determinant of their mRNA transcription and protein translation in *E. coli*.

Next, the growth rates of *E. coli* strains bearing different *mcr* genes varied, which negatively correlated with the 5′-end CAI values of the indicated genes (ρ = −0.918, *P* = 0.01; Fig. 5D). Moreover, by measuring the uptake of PI dye and sensitivity to SDS, the *E. coli* strains harbouring *mcr* genes with high 5′-end CAI values (*mcr-1*) revealed higher membrane permeability than those expressing the *mcr* gene, revealing codon bias at the transcriptional initiation region (Fig. 5E and Figure S20). This result indicated that the 5′-end codon bias appeared to be a critical regulator of the fitness of *mcr*-positive *E. coli* by impacting membrane permeability.

A previous study revealed that the prevalence of rare codons in the first ∼40 codons in yeast is due to the relatively recent evolution of these coding sequences.^64^ To gain more insight into the impact of 5′-end codon bias on the evolution of *mcr* family members, the neighbour-joining (NJ) method was utilised to construct a phylogenetic tree among the indicated *mcr* genes on the basis of their nucleotide sequences (Figure S21A). The NJ method identified *mcr-1* as the sister gene of *mcr-2*; *mcr-5* was grouped in the same evolutionary clade as *mcr-1* and *mcr-2*, whereas *mcr-3*, *mcr-4* and *mcr-8* were grouped in another evolutionary clade, revealing their close phylogenetic relationship. Additionally, the CAI values of the first 30 bp fragment of the indicated *mcr* genes were used in hierarchical clustering, which yielded two major clusters (Figure S21B). The dendrogram of the cluster analysis had a similar pattern to that of the phylogenetic tree, where Cluster I was composed of *mcr-1* and *mcr-2*, whereas *mcr-3*, *mcr-4* and *mcr-8* belonged to the second cluster. However, *mcr-5* was included in Cluster II but not in Cluster I. Compared with *mcr-8*, *mcr-5* was more closely related to *mcr-3* and *mcr-4*. Overall, the topological structure of the phylogenetic tree and the clustering results based on the CAI values of the *mcr* family genes were similar, suggesting that 5′-end codon optimality might impact the evolution of the *mcr* family.

Our results demonstrated that the codon usage frequency of the mRNA 5′-end was a crucial determinant regulating both the mRNA level and protein expression of the *mcr* family members in *E. coli* that impacted the fitness of the bacterial host by altering membrane integrity. Moreover, *mcr* genes with similar 5′-end CAI values presented similar phylogenetic trajectories, which indicated that the 5′-end CAI appeared to be a potential factor impacting the evolution of the *mcr* family.

### Inhibitors targeting the putative lipid A binding pocket exhibited striking efficacy against *mcr*-positive *E. coli*

**Fig. 6:**
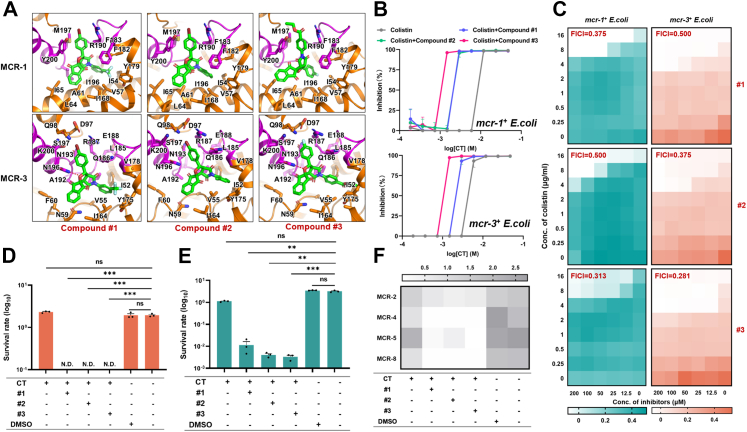
**Identification of inhibitors inactivating MCR proteins**. **(A)** The protein structure of the putative lipid A binding pocket of MCR-1 or MCR-3 in complex with the indicated inhibitor (bottom). The catalytic domain, linker domain and transmembrane domain are in cyan, magenta and orange, respectively. **(B)** AlamarBlue reduction assays of the indicated MCR inhibitors (compounds #1, #2 and #3) and colistin were performed to characterise the inhibition ability to the *E. coli* BW25113 strains harbouring *mcr-1* or *mcr-3*. The overnight culture of *mcr-1-*positive strain and *mcr-3-*positive strain were subcultured in fresh LB broth at a ratio of 1:100 and induced with 0.2% arabinose to express MCR-1 and MCR-3 respectively. Exponential phase cultures (∼1 × 10^8^ CFU/mL) were then collected and diluted into about 1 × 10^6^ CFU/mL in the 96-well microplates to a final volume of 100 μL. Bacteria were exposed to colistin (gradient: 0.25–64 μg/mL), alone or with the three compounds (100 μM), for 16 h. AlamarBlue solution was added to quantify bacterial proliferation, and the AlamarBlue reduction was quantified with λ_ex_ = 545 nm and λ_em_ = 595 nm. **(C)***mcr-1*^+^*E. coli* and *mcr-3*^+^*E. coli* were also used to test the efficacy of a drug combination consisting of the indicated compounds (#1, #2 and #3) and colistin through a checkerboard assay. A total of 1 × 10^3^ CFUs of the indicated strains were inoculated at initiation (T0), followed by treatment with drug combinations at various concentrations at 37 °C for 16 h (Tn). The OD_600_ was measured before and after the treatment. The graphs show the value of OD_600_ T0 minus the OD_600_ Tn of each well for both strains. A colour gradient heatmap displays white to aquamarine colours that indicate low to high values, respectively. The synergetic effect was further determined by calculating the FICI. **(D)** Viability of *mcr-3*-bearing *E. coli* challenged with colistin with/without the indicated compounds (#1, #2 and #3). An overnight culture of the *mcr-3*-positive strain was subcultured in fresh LB broth at a ratio of 1:100 and induced with 0.2% arabinose to express MCR-3. Exponential phase cultures were then collected and challenged with the indicated compounds. After treatment for 4 h, bacterial viability was determined by CFU assays. Colistin was used at a sub-MIC (8 μg/mL), and the concentration of each inhibitor was 100 μM. **(E)** The same assay was also performed to evaluate the antimicrobial effectiveness of the indicated drug combination against *E. coli* harbouring *mcr-1*, and the concentration of each inhibitor was 100 μM or 200 μM. For the *E. coli* strains carrying other MCR family members (MCR-2, MCR-4, MCR-5 and MCR-8), a colour gradient heatmap, with white to blue colours, indicates the low to high values of the survival rate, respectively, after treatment with the same indicated drug combinations. The concentration of each inhibitor was 100 μM **(F)**. All the experiments described above were performed three times with similar results. The error bars indicate the standard errors of the means (SEMs) for three biological replicates. A two-tailed unpaired *t* test was performed to determine the statistical significance of the data. ns, no significant difference; ∗∗, *P* < 0.01; ∗∗∗, *P* < 0.001. N.D., not detected.

As 5′-end codon bias is a critical determinant for the fitness of *mcr*-positive strains, which guarantee the continuously increased prevalence of *mcr-3*, it's necessary to explore efficient strategy to diminish the threat of *mcr-3*, and even other *mcr*-family members. Given that MCR-1 and MCR-3 are the predominant clinically relevant MCR family members, we conducted preliminary screening using MCR-1 and subsequently redocked the selected molecules onto MCR-3 to identify candidates that could inhibit both proteins. We extracted representative conformations of MCR-1 from the previous MD trajectories and used a cross-docking strategy to identify suitable ligand binding conformations. By designing a virtual screening pipeline, 14 candidates that exhibited promising binding modes for both MCR-1 and MCR-3 were identified (Figure S22). Among these compounds, three inhibitors significantly reduced the MICs of colistin of *mcr-1-* or *mcr-3*-bearing *E. coli* (compounds #1, #2 and #3; Table S6), which were assumed to interact with the putative lipid A binding pocket of MCR-1 and MCR-3 (Fig. 6A and Figure S23). However, the compounds that were expected to bind to the catalytic domain of MCR-1 and MCR-3 had no effect on the colistin susceptibility of the indicated strains (Table S6), suggesting that the putative lipid A binding pocket was a more promising target site than the catalytic domain for designing MCR inhibitors. To evaluate the specificity of the selected inhibitors, we collected several colistin-resistant but *mcr*-negative *E. coli* strains from clinical settings and tested the MICs of colistin in the presence of the inhibitors. As expected, the inhibitors failed to reduce the colistin MICs of these isolates (Table S7), confirming that the compounds specifically target MCR-1 or MCR-3 proteins. Consistently, these three MCR inhibitors enhanced colistin efficacy against *mcr-1-* or *mcr-3*-positive *E. coli in vitro* (Fig. 6B), with MICs of colistin in combination with inhibitors decreasing from 16 μg/mL to 4 μg/mL (Table S8), whereas treatment with the inhibitor alone conferred no antimicrobial activity on the indicated strains (Table S9). Meanwhile, all the tested inhibitors enhanced the antimicrobial efficacy of colistin. Measurement of the fractional inhibitory concentration index (FICI) revealed that compounds #1, #2 and #3 all exhibited synergistic activity with colistin (CT) against both *mcr-1*- and *mcr-3*-positive strains, with FICI ≤0.5 (Fig. 6C). When treated with 100 μM inhibitors, no viable *mcr-3*-bearing *E. coli* cells were observed after exposure to sub-MIC colistin (Fig. 6D), whereas 200 μM inhibitors significantly reduced the viability of *mcr-1*-positive strains in the presence of colistin (Fig. 6E), suggesting that the stronger affinity between MCR-3 and lipid A may explain the enhanced inhibitor efficacy against *mcr-3*-bearing strains. The time-kill assay demonstrated that compound #3 exhibited the strongest synergistic effects with colistin against *mcr-1*- and *mcr-3*-positive strains (Figure S24). Importantly, the inhibitors are predicted to target the conserved lipid A-binding pocket across other MCR family members (MCR-2, -4, -5, and -8; Figure S25), effectively restoring colistin sensitivity in *E. coli* expressing these proteins (Fig. 6F).

In summary, our results revealed that inhibitors targeting the putative lipid A binding pocket were able to restore the colistin sensitivity of *mcr-*positive *E. coli*, especially *mcr-3*-positive strains.

## Discussion

In this research, we defined the epidemiological features of *mcr-3*. The global prevalence of *mcr-3* increased continuously from 2005 to 2022. Unlike that of *mcr-1*, the withdrawal of colistin from animal husbandry failed to stop the dissemination of *mcr-3* in China. Moreover, our study comprehensively established the mechanism accounting for the varied fitness of *mcr-1-* or *mcr-3*-bearing *E. coli*, which emphasised that more attention is needed to prevent the continuous spread of *mcr-3*. In particular, the putative lipid A binding pocket of the MCR protein was used as a target site for designing effective inhibitors against *mcr*-positive *E. coli*.

Our data demonstrated that the prevalence of *mcr-3*, the second largest lineage of the *mcr* family, increased continuously from 2016 to 2019 in China even after the banning of colistin in animal husbandry. On the basis of our systematic review, *mcr-3* has been widely identified from numerous regions and sources over the past decade. Moreover, the cooccurrences of *mcr-3* and other antibiotic resistance genes are frequently reported in clinical isolates. For example, *mcr-3* was detected in extended-spectrum beta-lactamase-producing (ESBL) *E. coli* isolated from the bloodstream of an infected patient in Denmark.^65^ Additionally, a *Salmonella enterica* serotype Choleraesuis with the cooccurrence of *mcr-3* and *bla*_*CTX-M-55*_ was isolated from a bacteriaemic patient in Thailand.^66^ A case of cooccurrence of *mcr-3* and *fosA3* in *E. coli* was reported from a clinical sample in China.^67^ Since multidrug-resistant (MDR) bacteria are associated with nosocomial infections, the isolation of *mcr-3* from MDR clinical isolates emphasises the risk of *mcr-3* dissemination in colonised patients. By performing *in vitro* competitive assays using both clinically isolated strains and laboratory-adapted strains, we demonstrated the crucial result that the expression of MCR-3 imposed a lower fitness burden on the bacterial host than the expression of MCR-1. Moreover, it is interesting to note that the fitness benefit of *mcr-3*-bearing *E. coli* did not disappear even under the stress of colistin treatment or the murine intestinal environment. In summary, our results suggested that the fitness cost conferred by MCR-1 and MCR-3 determined the prevalence of the indicated strains after the withdrawal of colistin in animal fodder.

Expression level is a major regulator upon the fitness effect of genes.^68^ Although previous study has established that the under expression of MCR-1 induced by promoter mutations leads to increased fitness in *mcr-1*-bearing bacteria, the most notable finding in our current study was that codons with low usage frequency clustered at the translational initiation region of *mcr-1* or *mcr-3*, which had crucial effects on mRNA stability, protein expression, membrane permeability and bacterial fitness (Figure S26).^21^ Many studies have demonstrated that, in both eukaryotic and prokaryotic cells, rare codons clustered at the 5′-end of the coding region slow the speed of cotranslational folding governed by ribosomes, which improves the efficiency of cotranslational protein folding and gene expression by minimising ribosome collision.^69^^,^^70^ However, the results from our study were the opposite of this conventional theory. The protein expression of *mcr* genes with codons with high usage frequency clustered at the 5′-end was significantly greater than that of those harbouring nonoptimal codons in the same region. In line with observations in yeast,^64^ our results demonstrated that increasing the percentage of optimal codons at the 5′-end of the coding region promoted the expression of the target protein. By performing genome-wide mRNA decay analysis in yeast, Vladimir et al. reported that nonoptimal codons reduce the stability of mRNAs.^57^ Hence, it is reasonable to believe that nonoptimal codons clustered at the first 30 bp fragment reduced the stability of the mRNA 5′-end. Michael et al. provided convincing evidence that, for horizontally transferred genes in *E. coli*, 5′-end mRNA stability and codon optimality impact host fitness.^71^ The 5′-end overstabilisation of mRNAs enables “escape” from degradation and results in the accumulation of transcripts. Two lines of evidence in our study corroborated this theory: i) codon optimisation at the 5′-end of *mcr-3* (5′-opi) increased the mRNA half-life, and ii) the overexpression of RraA increased the cellular mRNA level of *mcr-3* and reduced the growth rate of the bacterial host. RraA is an inhibitor of RNase E, an essential ribonuclease in *E. coli*.^62^^,^^63^ It is possible that the nonoptimal codons clustered at the 5′-end of the *mcr-*3 mRNA diminished the stability of the transcripts by increasing the efficiency of RNase E-based degradation.

Currently, many studies have focused on investigating MCR inhibitors,^13^ which reverse the colistin sensitivity of *mcr*-expressing strains. Nevertheless, most of these inhibitors do not specifically target MCR proteins, and the drug combinations had additive effects rather than synergistic effects. For example, Sophie et al. reported that the addition of carbonyl cyanide *m*-chlorophenyl hydrazine (CCCP), a chemical inhibitor of oxidative phosphorylation, was able to reverse the susceptibility of *mcr-1*-positive bacteria to colistin.^72^ However, this effect was specific to not only a *mcr-1*-positive strain but also colistin-resistant strains induced by chromosomal mutations. Moreover, inhibitor that specifically target MCR-3 is quite limited. Current available MCR-1 inhibitors are not structure-based, and the antimicrobial mechanism remains unclear. Recently, Zhang et al. demonstrated that silver targeting the catalytic domain of MCR-1 allowed re-sensitisation of the *mcr-1*-bearing strain to colistin.^73^ However, such a strategy failed to reduce the MICs of colistin in bacteria carrying *mcr-3*. Indeed, the amino acid sequence of the MCR-3 catalytic domain has a low similarity with that of MCR-1 and might result in a distinct protein conformation. Notably, the putative lipid A binding pocket, which is highly conserved among the MCR family members,^9^ appears to be a promising target site for designing effective inhibitors against *mcr*-positive bacteria. Moreover, our previous research suggested that MCR-1-mediated lipid A modification might be guaranteed by a two-step process in “loading-transferring” mode, where the initial process involving the interaction between the lipid A binding pocket and the lipid A substrate was highly important for the colistin resistance of the bacterial host. Upon screening from a small-molecule library, the three selected inhibitors that significantly increased the susceptibility of *mcr-1*- or *mcr-3*-harbouring *E. coli* to colistin were all compounds that targeted the putative lipid A binding pocket, whereas the inhibitors targeting the catalytic domain failed to diminish the colistin MICs of the indicated strains. Further study is needed to investigate whether the indicated compounds inactivate the enzymatic activity of MCR proteins by abolishing the interaction between the lipid A binding pocket and the lipid A substrate. In addition to *mcr-1*- or *mcr-3*-positive strains, drug combinations containing MCR inhibitors and colistin enabled the resensitisation of *E. coli* carrying other *mcr* members toward colistin. Hence, it is reasonable to believe that the MCR inhibitors verified in our study could represent an effective strategy to combat *mcr*-positive strains, especially *mcr-3*-bearing bacteria.

There were several limitations in our study: 1) while our study establishes that 5′-end codon optimality significantly impacts mRNA stability of *mcr-1* and *mcr-3* in *E. coli*, codon optimisation of the *mcr-3* 5′-end failed to fully restore its mRNA level to that of *mcr-1*. This suggests that mRNA stability in the MCR family (particularly MCR-1 and MCR-3) is not solely regulated by 5′-coding region codon optimality; 2) by demonstrating the correlation between 5′-end codon bias and the fitness of *mcr*-positive *E. coli*, our study found that *mcr-3* mediated the lowest biological burden among the *mcr* family, which is matched with the continuously increased prevalence of *mcr-3* and highly suggested that a potential threat challenging the clinical treatment outcome of colistin might be mediated by *mcr-3* rather than other *mcr* members. However, the actual prevalence of other *mcr* members has not been determined yet; 3) although binding free energy calculations indicate stronger lipid A affinity for MCR-3 than MCR-1, *mcr-3*-positive cells exhibited greater membrane integrity than *mcr-1*-positive cells. Previous work demonstrated that lipid A binding affinity to MCR-1's lipid A-binding pocket critically affects bacterial host fitness. However, whether divergent lipid A affinities between MCR-1 and MCR-3 regulate strain-specific fitness differences remains unresolved; 4) despite comparable 5′-end CAI values between *mcr-2* and *mcr-1*, *mcr-2* showed lower mRNA/protein expression levels yet conferred faster growth and enhanced membrane integrity compared to *mcr-1*. These findings imply that MCR-mediated fitness costs are multifactorial, extending beyond 5′-end codon optimality; 5) Codon optimisation within the first 30 bp of *mcr-3* increased mRNA stability, whereas optimisation of downstream regions had no effect. Clustering of nonoptimal codons at the 5′-end may promote RNase E-mediated mRNA decay, as RraA overexpression elevated *mcr-*3 mRNA levels. Intriguingly, 5′-end optimisation also increased host membrane permeability, but the mechanistic links between 5′-end codon optimality, RraA-dependent mRNA degradation, and membrane integrity remain unclear; 6) although the native *mcr-3* promoter is highly conserved, its impact on MCR-3 protein expression and host fitness was not systematically characterised; 7) while identified MCR inhibitors effectively restored colistin sensitivity in *mcr*-positive *E. coli*, their moderate-to-low potency (requiring 100–200 μM concentrations to reduce colistin MICs) highlights the need for structural optimisation to improve drug-like properties.

In conclusion, our current study emphasised that increased attention should be given to potential outbreaks of *mcr-3* even after the withdrawal of colistin in animal husbandry, and the MCR-specific inhibitors we identified may represent an alternative strategy that effectively restores the efficacy of colistin against *mcr*-positive bacteria.

## Contributors

LLJ, WWL, LYX and WL contributed equally to this study. HZ, ZLL, TGB, FSY, and BF conceived the idea, designed and supervised the study. LLJ, WWL, LYX, WL, ZH, WYX, ZDR, LLX, LLZ, WGX, WJ and LMS conducted the experiments and produced the tables and figures. LLJ, LYX, WL, XS, YMT and ZYH searched the literature. DM, LLJ, LYX, WL, ZYH and LJC contributed to data analysis and interpretation. ZLL, BF, FSY, and TGB assessed and verified the underlying data. LLJ, WL, FSY, BF and TGB wrote the manuscript. All authors reviewed, revised, and approved the final report.

## Data sharing statement

All data collected for the study that underpins the conclusions are presented in the main paper and the Supplementary Materials. Data can be shared by the corresponding author upon reasonable request and can be contacted at tiangb@mail.sysu.edu.cn. Raw data of RNA-sequencing can be accessed through GEO Dataset repository under the accession number: GSE300593.

## Declaration of interests

The authors declare no conflicts of interest.

## Appendix A Supplementary data

Reagent and Antibody Validation Statement

Supplementary Table S3

Western blot
