# Supplementary material for: Fitness costs of mobilised colistin resistance gene 3 (mcr-3): systematic review, epidemiological study, and functional analysis
Source: eBioMedicine. 2025 Sep 12;120:105923. doi: 10.1016/j.ebiom.2025.105923 (PMC12571581; doi:10.1016/j.ebiom.2025.105923)
Supplement: Reagent and Antibody Validation Statement [file mmc1.docx]

**Reagent Validation Statement**

All **reagents** used in this study are commercially sourced. Information about the antibodies, including catalog numbers, vendors, and Research Resource Identifier (RRID), is provided in the Methods section. Relevant references for the applications of these **antibodies** are as follows:

**RpoB**

This antibody is a monoclonal type produced in mice and verified to react with E. coli. It was generated using a purified protein as the immunogen and purified via affinity chromatography. The antibody is in a phosphate-buffered solution, pH 7.2, at 1.0 mg/ml. Handle it aseptically as it lacks preservatives like Sodium Azide. Store undiluted between 2°C and 8°C; check the vial label or CoA for details.

For Western blotting (WB), quality-tested and recommended, use 0.00625 - 1.0 µg/ml. Dilute in 5% nonfat dry milk/TBS-T. Incubate for 2 hours at room temperature or overnight at 4°C with agitation. After primary incubation, wash three times for 5 minutes each with TBS-Tween-20. Incubate with HRP-conjugated secondary antibody (1:1000 - 1:5000 in 5% milk/TBS-T) for 30 minutes at room temperature. Wash four times for 10 minutes each with TBS-Tween-20 and once for 2 minutes with PBS.

For detection, incubate the membrane with ECL substrate (10ml, 1-2 minutes, 0.125ml/cm²). Drain excess, wrap the blots, and expose to X-ray film (5 seconds to 60 minutes).

**LPS core**

This antibody is a monoclonal antibody available in various amounts. Quantities >500 µg are provided without preservatives and carrier-free, while quantities ≤500 µg include preservatives and a carrier. The immunogen is 10E8 heat-killed bacteria, and the isotype is Mouse IgG2a. Store at 4°C for at least one year. Dilute in blocking buffer as suggested, or try dilutions from 1:50 to 1:3000 if not specified. Incubation can use blocking buffer or TBST, with time ranging from a few hours to overnight. Prolonged incubation should use a more dilute antibody. Incubate at room temperature or 4°C, with agitation recommended.

**HA-tag**

This monoclonal antibody, produced by immunising animals with a synthetic peptide containing the influenza hemagglutinin epitope (YPYDVPDYA), is specific for detecting exogenously expressed HA-tagged proteins. It may cross-react with an unknown protein of ~100 kDa. The antibody is supplied in a buffer containing 10 mM sodium HEPES (pH 7.5), 150 mM NaCl, 100 µg/ml BSA, 50% glycerol, and less than 0.02% sodium azide. For storage, keep at -20°C and avoid aliquoting.In Western blotting, dilute the antibody 1:1000 and incubate the membrane with the primary antibody in 10 ml of primary antibody dilution buffer with gentle agitation overnight at 4°C.

**Goat anti-rabbit horseradish peroxidase (HRP) conjugate**

This antibody, a rabbit-derived polyclonal antibody, is validated for multiple applications. In Western Blot (WB), it can be diluted between 1:5000 and 1:20000. For Immunohistochemistry (IHC), the recommended dilution range is 1:500 to 1:1000. It also shows efficacy in ELISA at a dilution of 1:5000 to 1:20000. The antibody is reactive to mouse, with immunogen being mouse IgG, making it particularly valuable in immunology research. It is supplied in liquid form, in a storage buffer containing 0.01M Phosphate Buffered Saline (PBS), pH 7.2, with 1% BSA, 50% glycerol, and 0.05% Sodium Azide. For short-term storage, it should be kept at 4°C. For long-term storage, it is recommended to aliquot and store at -20°C, avoiding freeze/thaw cycles.

**Rabbit anti-mouse HRP conjugate**

This product is a horseradish peroxidase (HRP)-conjugated goat anti-rabbit IgG (H+L) antibody, catalogued as IH-0011. It is available as a lyophilised powder for import or as a liquid when imported and repackaged, in which case it is dissolved in distilled water. The product volume is 0.1ml, supplemented with an additional 0.1ml of glycerol, and the antibody concentration is 0.4mg/ml. For application-specific dilution ranges, in immunohistochemical staining of tissue sections, it is recommended to dilute between 1:250 and 1:2,500. In ELISA and Western Blots using enzyme substrate colorimetry, a dilution of 1:2,500 to 1:50,000 is advised. For Western Blots using chemiluminescence, the suggested dilution range is 1:5,000 to 1:100,000. The antibody is formulated in a buffer containing 0.01M sodium phosphate and 0.25M NaCl, with a pH of 7.6. It includes 15mg/ml of bovine serum albumin (BSA), which is IgG and protease-free, serving as a stabiliser. Storage at -20℃ is recommended for a period of up to one year. The antibody shows specificity for the heavy chains of rabbit IgG and the light chains of most rabbit immunoglobulins. It does not react with non-immunoglobulin serum proteins. However, cross-reactivity with immunoglobulins from other species cannot be ruled out.
