# Supplementary material for: Fitness costs of mobilised colistin resistance gene 3 (mcr-3): systematic review, epidemiological study, and functional analysis
Source: eBioMedicine. 2025 Sep 12;120:105923. doi: 10.1016/j.ebiom.2025.105923 (PMC12571581; doi:10.1016/j.ebiom.2025.105923)
Supplement: Supplementary Figures and Tables [file mmc2.docx]

**Supplementary figures**

**
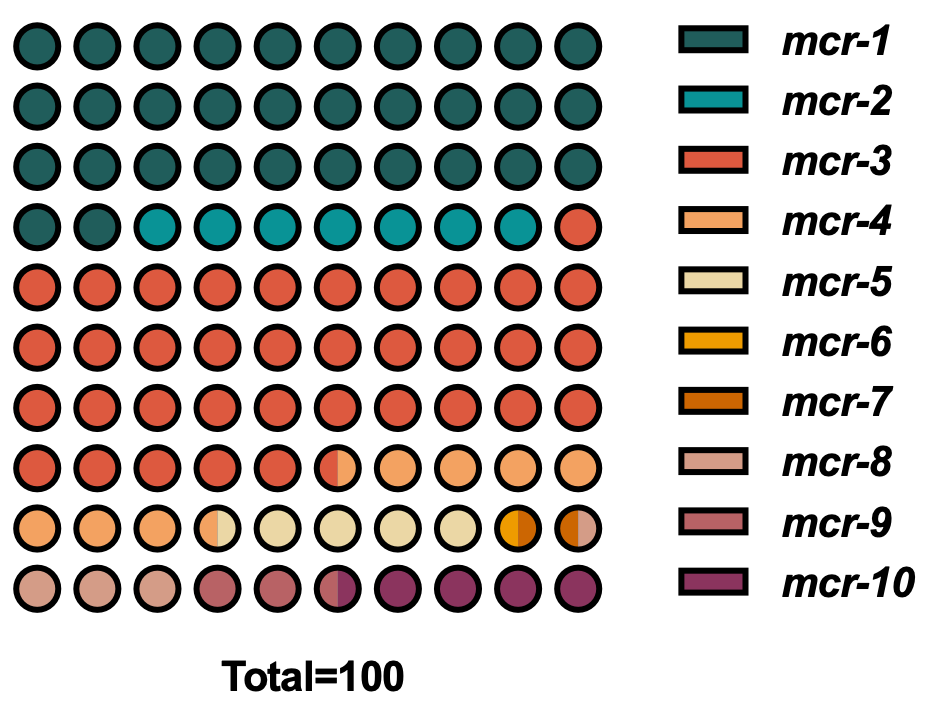
**

**Figure S1. Proportion of *mcr* members among the *mcr* family.**

To date, the *mcr* family totally contains 115 members, with 37 belonging to *mcr-1* (32.2%), 8 to *mcr-2* (7.0%), 42 to *mcr-3* (36.5%), 9 to *mcr-4* (7.8%), 5 to *mcr-5* (4.3%), 1 to *mcr-6* (0.9%), 1 to *mcr-7* (0.9%), 4 to *mcr-8* (3.5%), 3 to *mcr-9* (2.6%), and 5 to *mcr-10* (4.3%). All the *mcr* genes used here were sampled from the *Bacterial Antimicrobial Resistance Reference Gene Database* of the NCBI website.


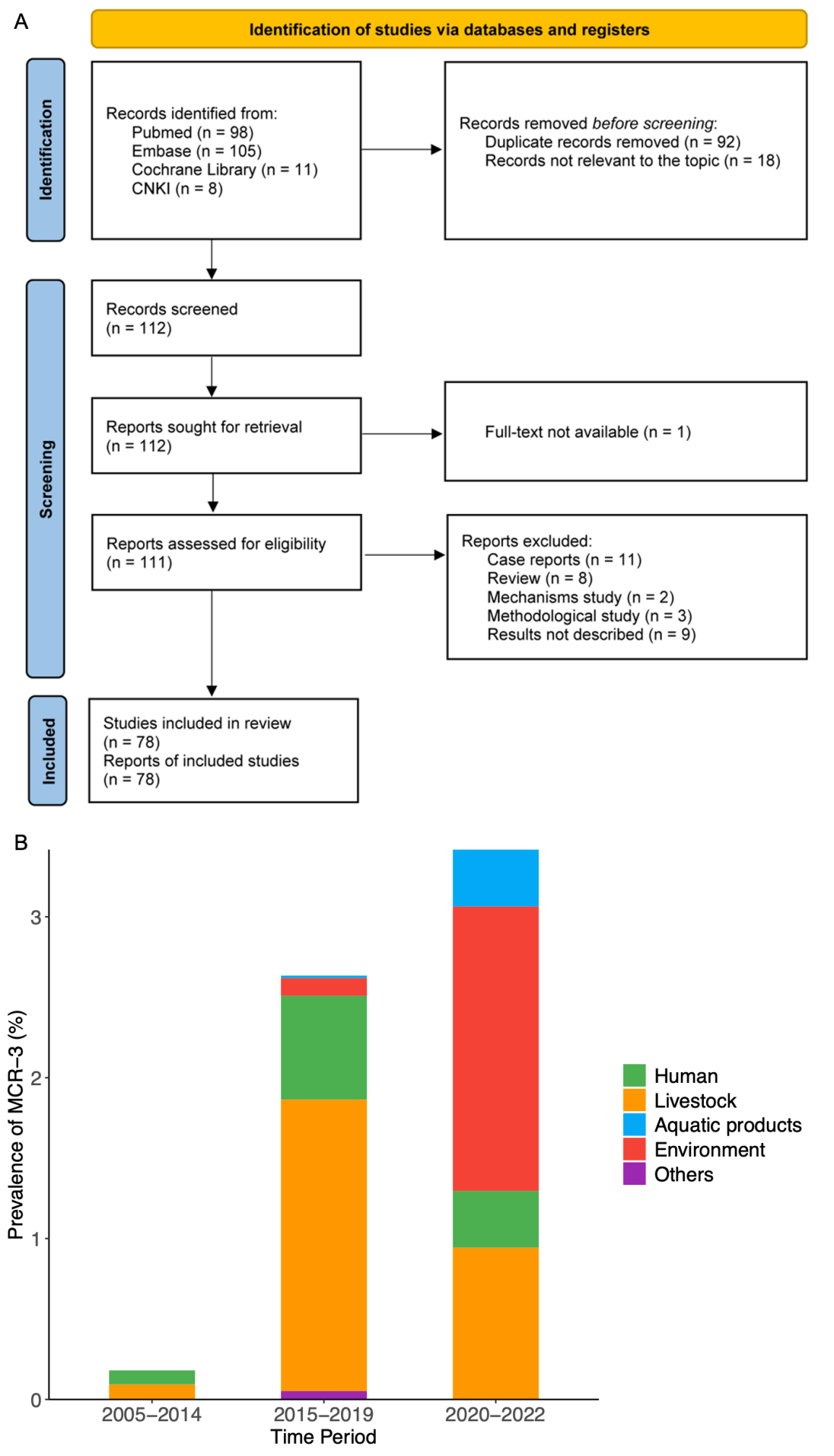


**Figure S2. Global prevalence of *mcr-3* analysed by meta-analysis.**

**(A)** The PRISMA flow diagram shows the review process involving the selection and inclusion of studies for the systematic reviews and meta-analyses.

**(B)** The histogram represents the global prevalence of *mcr-3* during the periods of 2005-2014, 2015-2019 and 2020-2022, respectively. Each stacking bar illustrates the percentage of different sources for *mcr-3*-bearing isolates during each period.

**
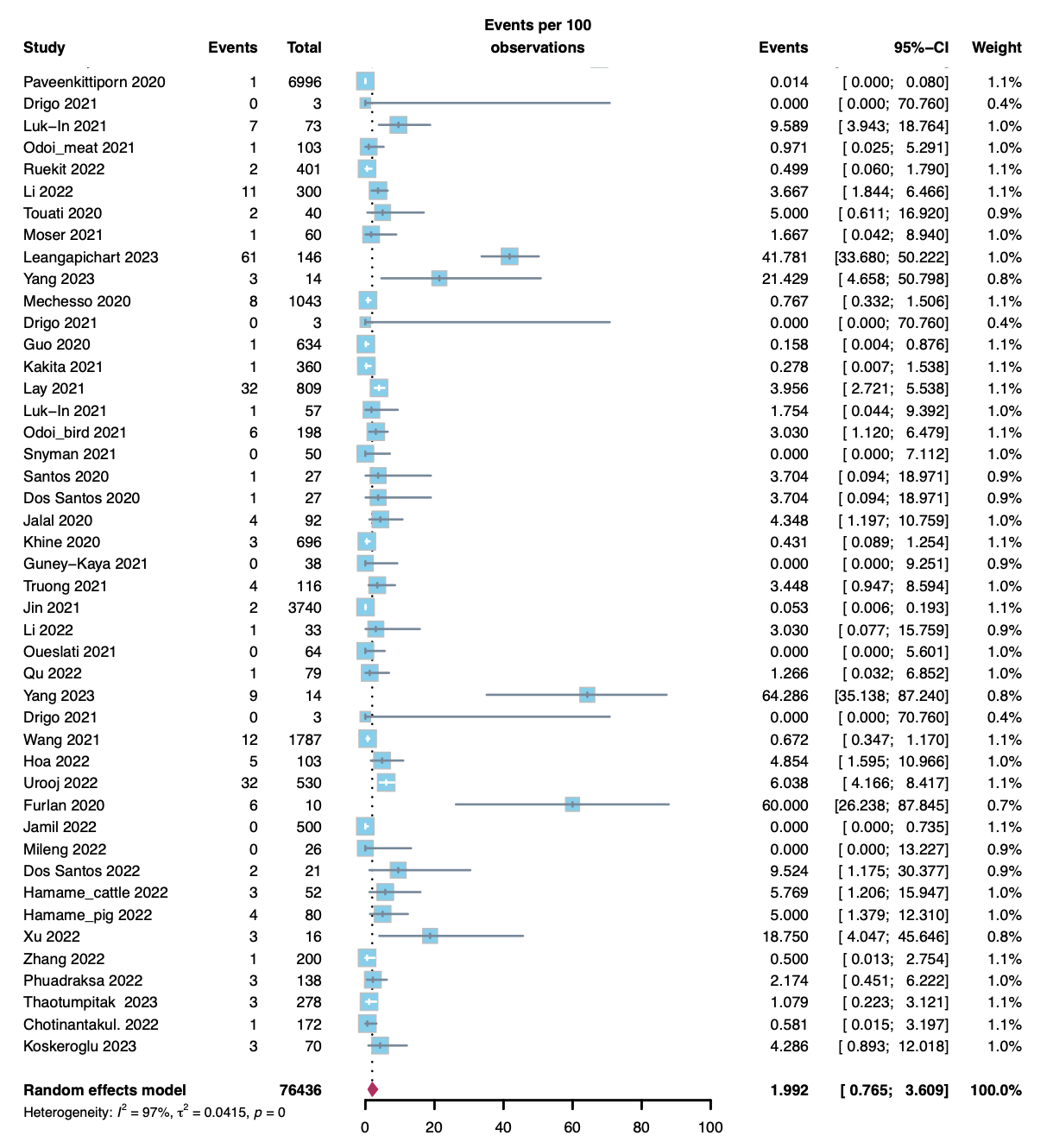
**

**Figure S3. Forest plot of included studies.**

Estimated pooled positive rate of *mcr-3* is 1.99% (95% CI: 0.77-3.61%).


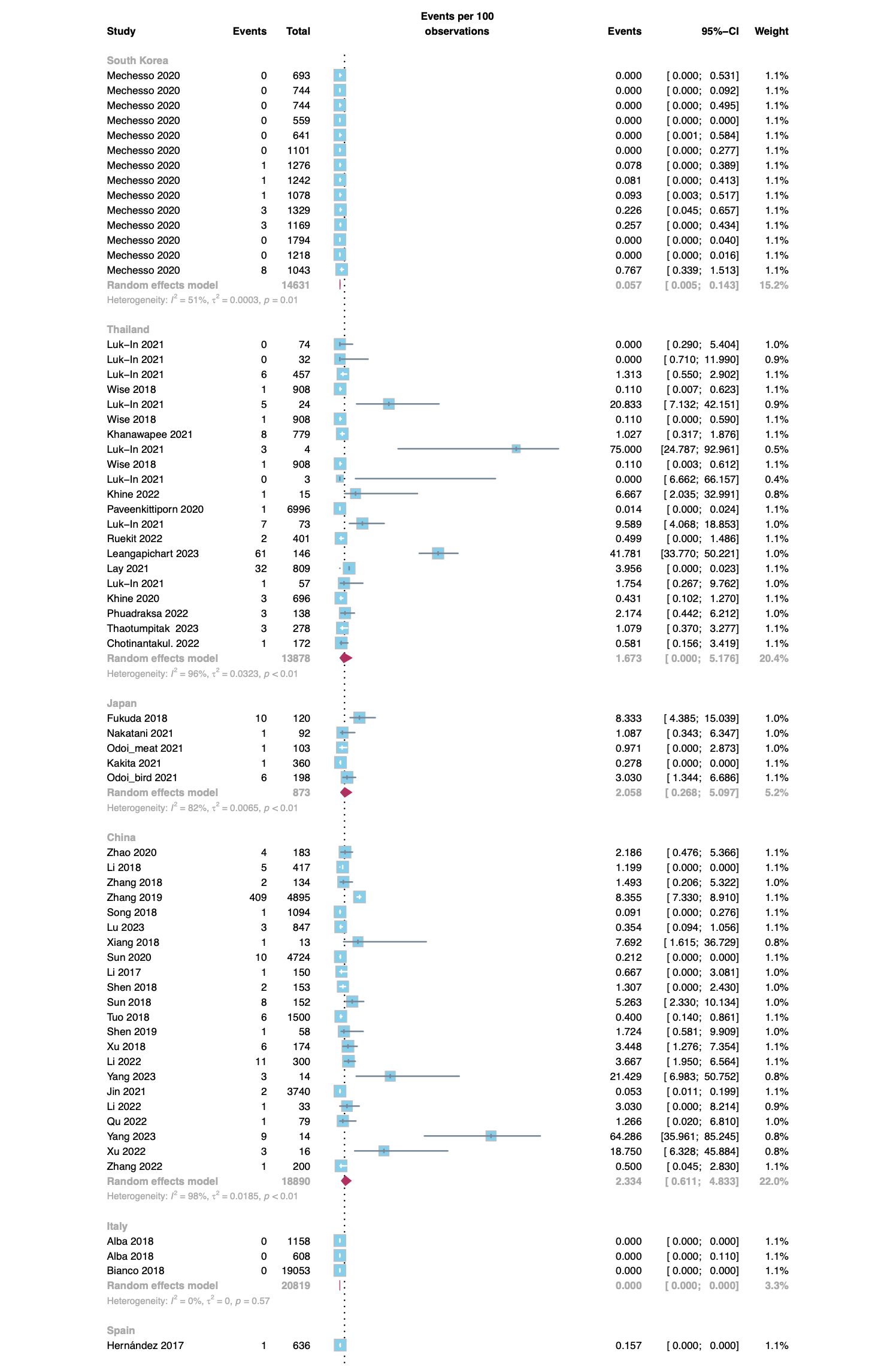


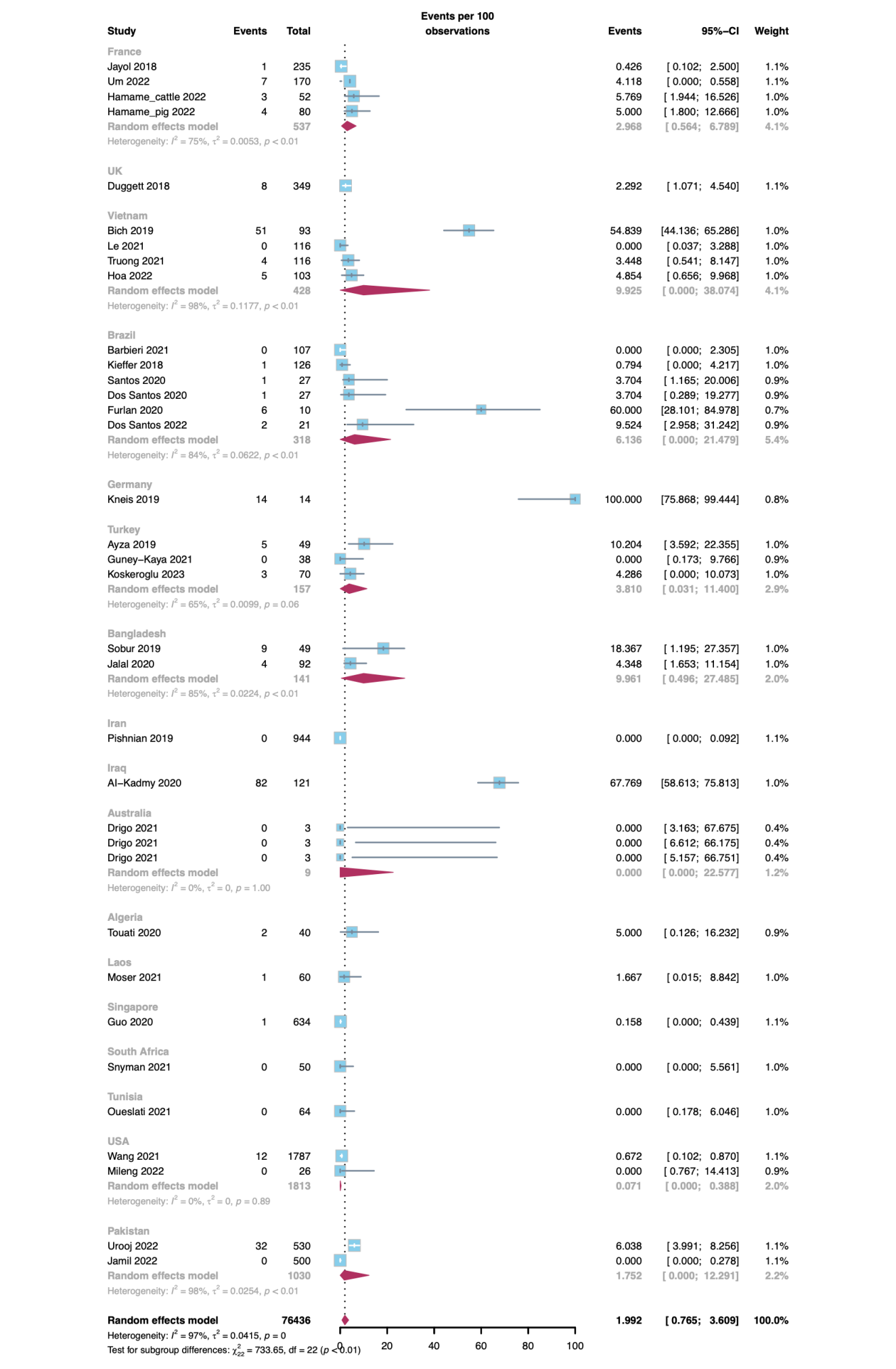


**Figure S4. Forest plot of subgroup analysis for different sampling regions.**


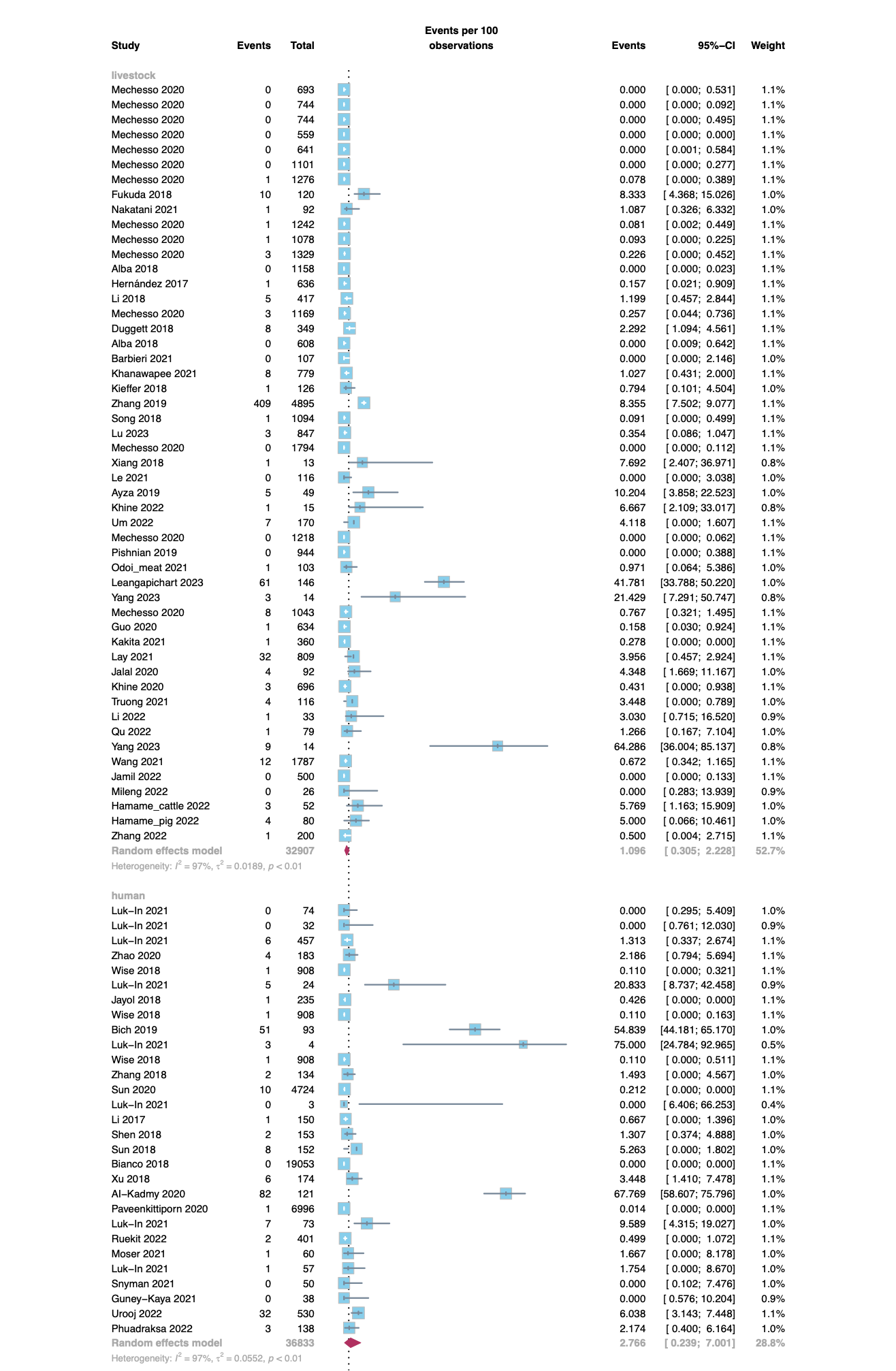


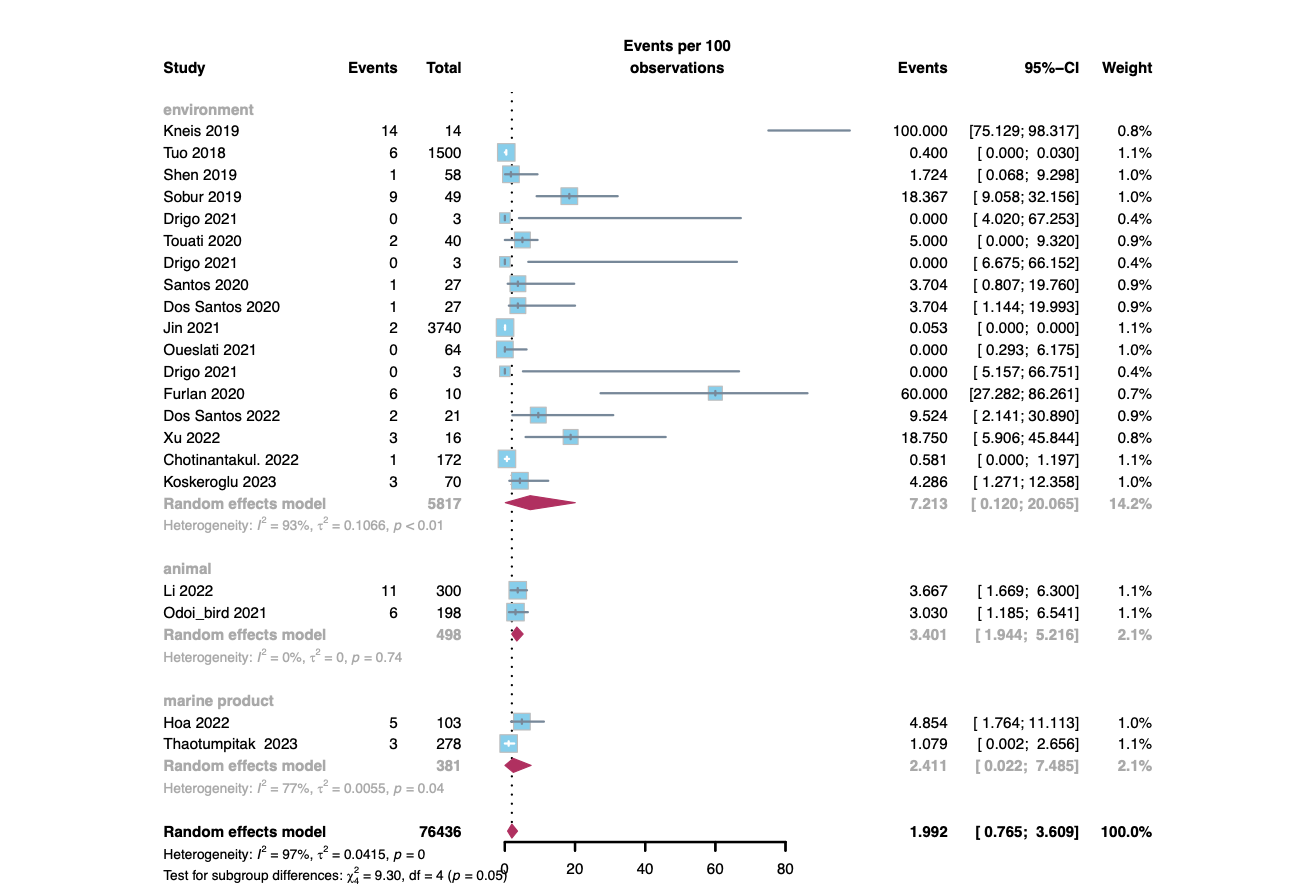


**Figure S5. Forest plot of subgroup analysis for different sampling source.**


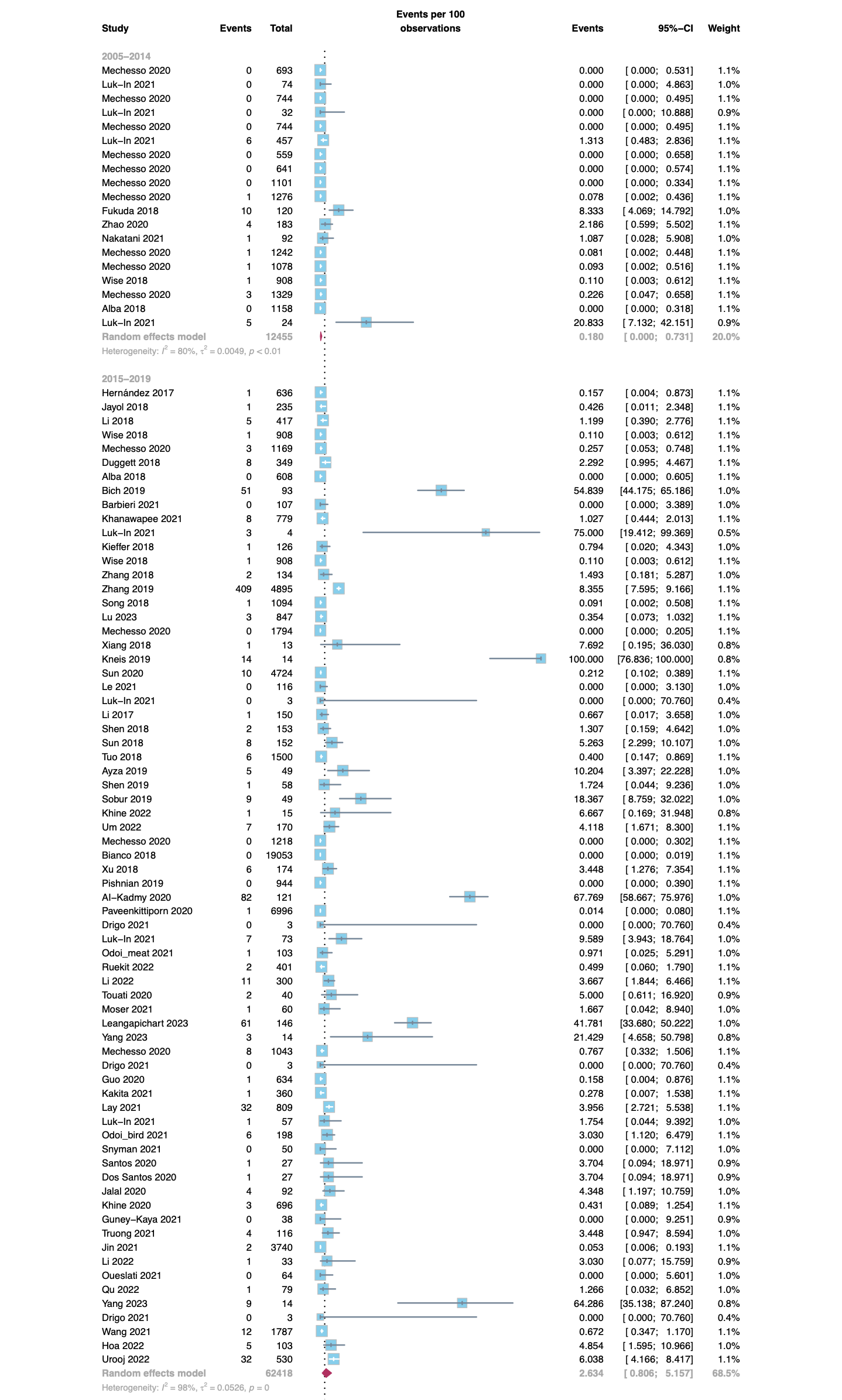


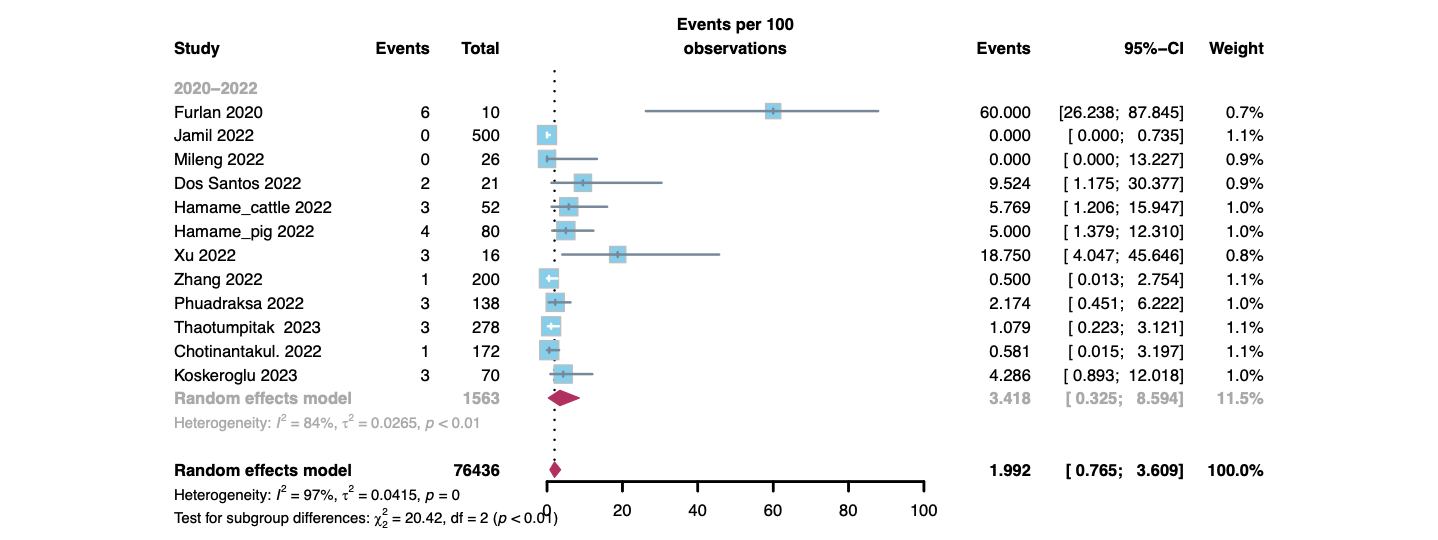


**Figure S6. Forest plot of subgroup analysis for different sampling time periods.**

**Figure S7. Bayesian hierarchical analysis revealed fixed effect of confounding factors.**

By comparing different sampling regions, sampling time and sampling sources, most confounding factors did not contribute to significant change of effect size. Importantly, no significant impact on effect size was identified along different sampling time points.


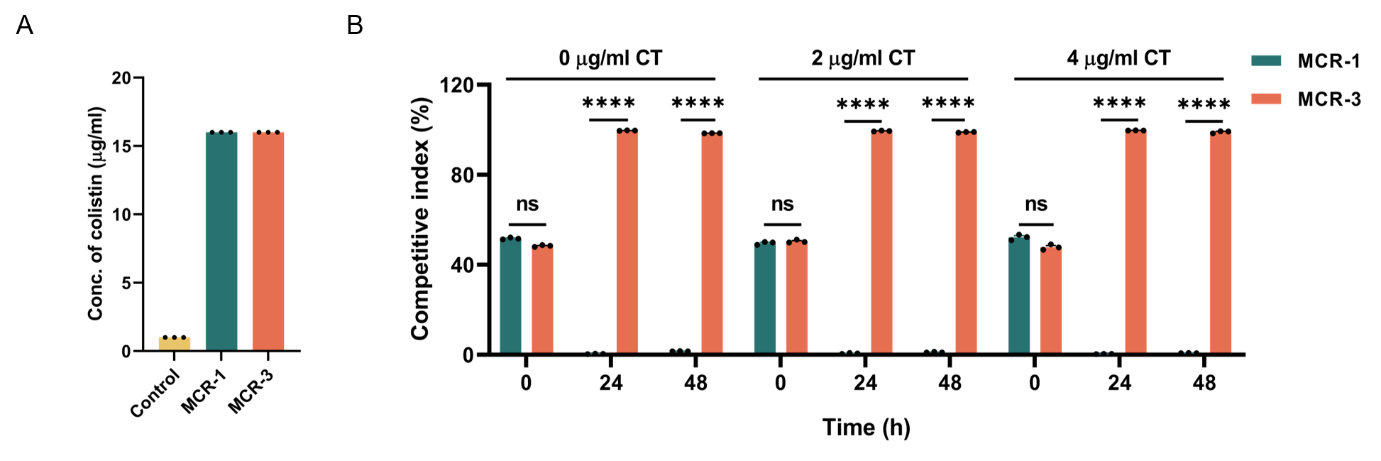


**Figure S8. Fitness comparison upon *mcr-1* or *mcr-3*-bearing *E. coli* under colistin stress.**

**(A)** Assessment of the susceptibility of MCR-1 or MCR-3-expressing *E. coli* to colistin. The strain carrying the empty plasmid was used as a control.

**(B)** Fitness evaluation of *E. coli* BW25113 harbouring *mcr-1* or *mcr-3* stressed with colistin. The cultures of GFP^-^ *mcr-3*-bearing *E. coli* and GFP^+^ *mcr-1*-bearing strain were mixed in the ratio of 1:1 at the initiation, and colistin was added to a final concentration of 4 μg/mL. Samples were collected at 0 h, 24h and 48h after initiation. And the percentage of target strains were determined by flow cytometry and analysed by FlowJo (version 10) software. The y-axis shows the percentage of target strain, and the x-axis shows period of growth (h).

The experiments were performed thrice with similar results. Error bars indicate standard errors of the means (SEMs) for three biological replicates. A two-tailed unpaired t test was performed to determine the statistical significance of the data. ns, no significant difference; ****, *P*< 0.0001.


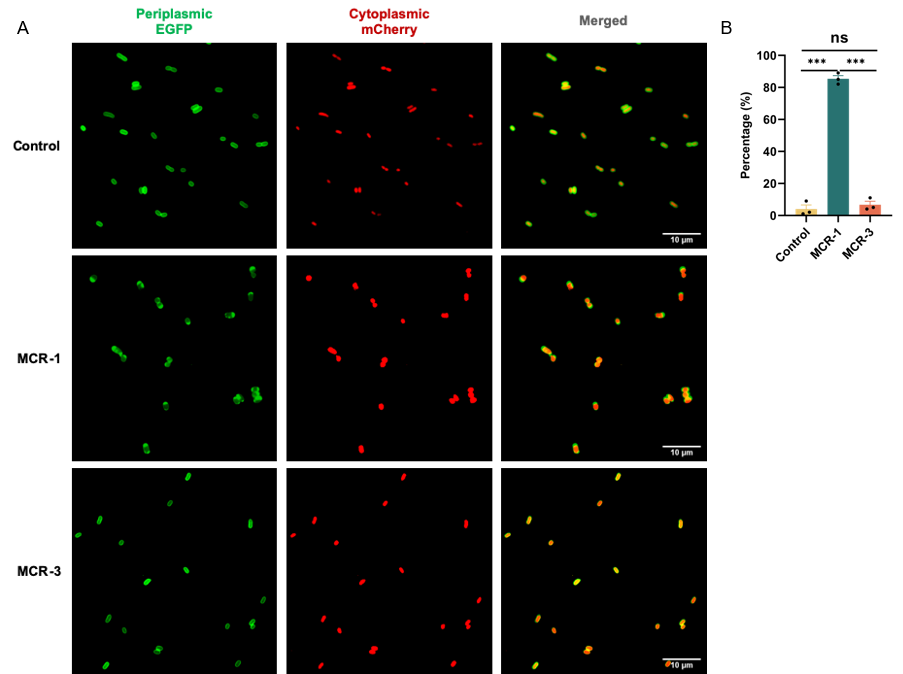


**Figure S9. Observation of periplasm shrinkage of *mcr-1-* or *mcr-3*-bearing *E. coli*.**

**(A)** Representative periplasmic GFP and cytoplasmic mCherry images of *E. coli* BW25113 expressing MCR-1 or MCR-3. Overnight cultures were sub-cultured into fresh LB broth in the ratio of 1:100 and induced with 0.2% arabinose to express target proteins, and logarithmic phase cultures were sampled for microscope observation and analysis. Shrinkage of the cytoplasm was evident by the bright periplasmic GFP signal localized at bacterial pole(s). The fluorescent images were processed and analysed by Fiji (version 2.1.0) software.

**(B)** Calculation of percentage of cells exhibiting shrinkage at bacterial pole(s).

The experiments were performed thrice with similar results. Error bars indicate standard errors of the means (SEMs) for three biological replicates. One-way ANOVA analysis was performed to determine the statistical significance of the data. ns, no significant difference; ***, *P*< 0.001.


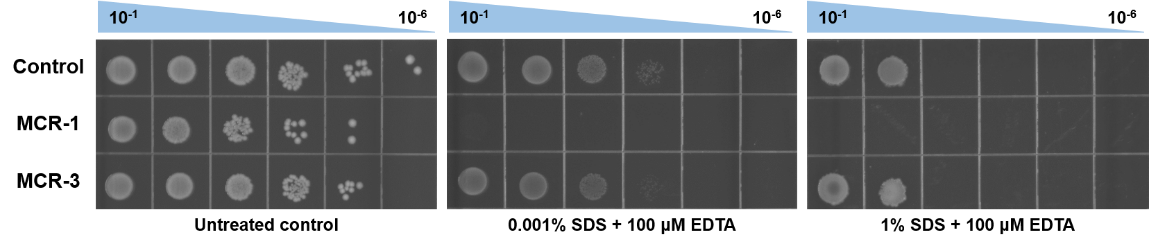


**Figure S10.** **SDS-EDTA sensitivity of *mcr-1* or *mcr-3*-bearing *E. coli* BW25113.**

Fresh cultures of *E. coli* BW25113 bearing empty vector, *mcr-1* or *mcr-3* were spotted on LB agar plates containing 0.001% SDS and 100 μM EDTA. Ten-fold serial-dilution of indicated cultures were inoculated onto the agar plates.


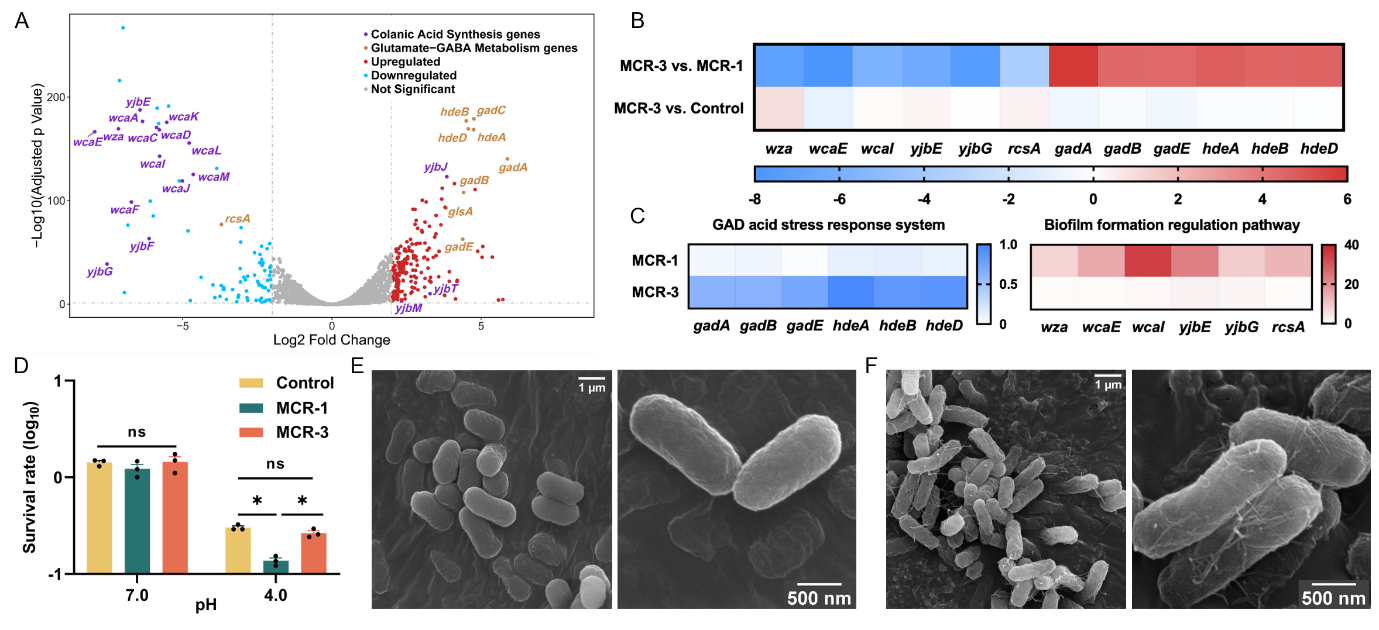


**Figure S11. Transcriptomic analysis of *E. coli* BW25113 expressing MCR-1 or MCR-3.**

**(A)** Volcano diagram of the differentially expressed protein between *E. coli* BW25113 carrying *mcr-3* and *mcr-1* determined by transcriptomic analysis. The differential expression threshold was set as log_2_ fold change >1 and p value <0.05. Red dots represent significantly upregulated genes, while blue dots represent significantly downregulated genes. The genes related to the colanic acid (CA) synthesis and glutamate-GABA metabolism are annotated and coloured in orange and purple, respectively.

**(B)** A colour gradient heat map represents the differentially transcriptional level of genes related to colanic acid (CA) synthesis and glutamate-GABA metabolism between MCR-3-expressing *E. coli* and control or *mcr-1*-bearing strain based on the transcriptomic analysis, with hot (red) to cold (blue) colours indicating high to low values.

**(C)** The transcriptional level of differentially transcribed genes between *mcr-1* and *mcr-3*-bearing strains were determined by q-PCR, which was normalized to the transcript level of the housekeeping gene *rpoB* and quantified with ΔΔCT analysis. The heat map represents the fold change in target genes transcriptional levels for MCR-1 or MCR-3-expressing *E. coli* compared with those of the empty plasmid control, with hot (red) to cold (blue) colours indicating high to low values.

**(D)** The viability of MCR-1 or MCR-3-expressing strains stressed with acid. Overnight cultures of the indicated strains were sub-cultured into fresh LB broth (pH=4.0) in the ratio of 1:100 and induced with 0.2% arabinose. CFUs assay was carried out to determine the survival rate at 4 h after initiation (Tn). The y-axis represents the value of log10(viability at Tn/ viability at T0), and the x-axis represents the pH value of LB broth.

Observation of biofilm formation of *mcr-3*-bearing strain **(e)** and *mcr-1*-expressing *E. coli* **(f)** by using SEM micrographs. Overnight culture of target strain was subcultured into fresh LB broth in the ratio of 1:100 and induced with 0.2% arabinose. The stationary phase cultures were collected for sample preparation.

All the above-described experiments were performed thrice with similar results. Error bars indicate standard errors of the means (SEMs) for three biological replicates. One-way ANOVA analysis was performed to determine the statistical significance of the data. ns, no significant difference; *, *P*< 0.1.


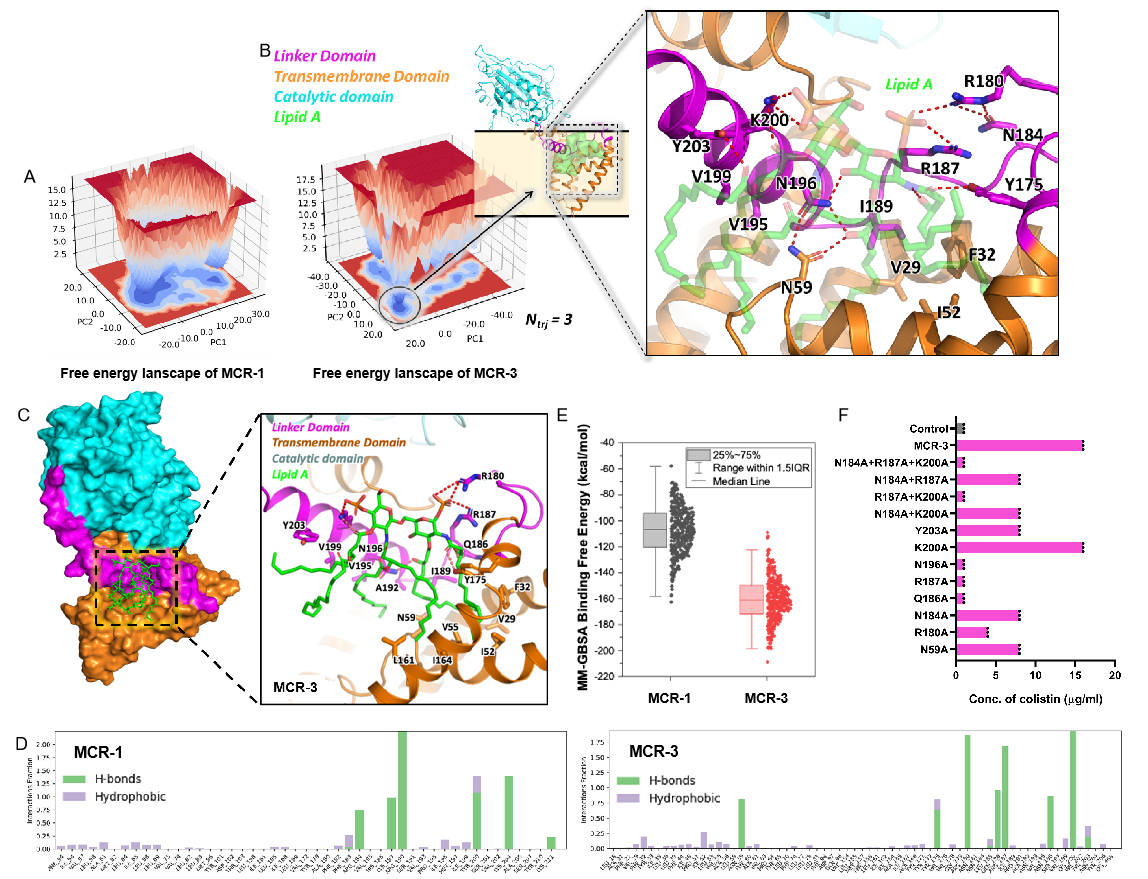


**Figure S12. Identification of putative lipid A binding pocket in MCR-3.**

**(A-B)** The free energy landscapes upon MCR-1/MCR-3 proteins and the complex structure of MCR-3-lipid A.

**(C)** Protein structure of MCR-3 and close-up view of the lipid A binding cavity at the linker domain. The catalytic domain, linker domain and transmembrane domain are in cyan, magenta and orange, respectively, with lipid A is shown as green sticks and salt bridges for interaction as red dashes.

**(D)** The frequency histograms and MM-GBSA binding free energy of the interaction between MCR-1 and MCR-3 with lipid A during MD trajectories.

**(E)** Estimations of the binding free energies of lipid A against MCR-1 and MCR-3 were calculated. The calculation was performed over the last 200 ns trajectory after reaching equilibrium by using MM-GBSA.

**(F)** Functional evaluation of the key residues interacting with lipid A in the lipid A binding cavity of MCR-3. To verify the influence of target mutations on colistin resistance activity, the colistin MICs of the indicated mutants were determined by agar dilution MIC tests. The experiments were performed thrice with same results.


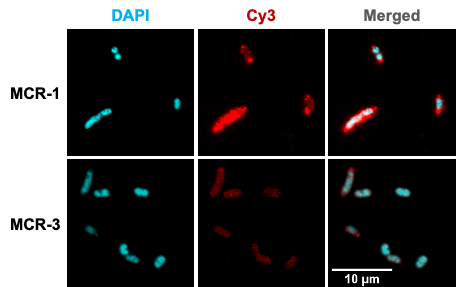


**Figure S13. Immunofluorescence staining of MCR-1/MCR-3 on *E. coli* BW25113.**

A HA-tag was added at the C-terminal of MCR-1/MCR-3. Cy3-coniugated anti-mouse IgG antibody was served as secondary antibody to label MCR-1/MCR-3 on *E. coli* BW25113. DAPI was used to stain the cytoplasmic chromosome of indicated strains.


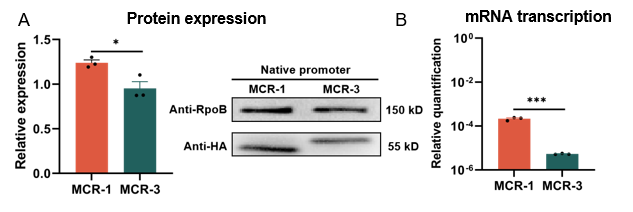


**Figure S14. Protein/mRNA level of *mcr-1*/*mcr-3* under the regulation of native promoters.**

**(A)** Determination of protein expression levels of NP-MCR-1/MCR-3 in *E. coli* BW25113 through western blot. HA-tag was added at the C-terminal of indicated proteins.

**(B)** The mRNA transcriptional level of *mcr-1* and *mcr-3* regulated by native promoters in *E. coli* BW25113 were determined by q‒PCR, which was normalized to the transcript level of the housekeeping gene *rpoB* and quantified with ΔΔCT analysis.

All the above-described experiments were performed thrice with similar results. Error bars indicate standard errors of the means (SEMs) for three biological replicates. A two-tailed unpaired *t* test was performed to determine the statistical significance of the data. ns, no significant difference; *, *P*< 0.1; ***, *P*< 0.001. NP, native promoter.


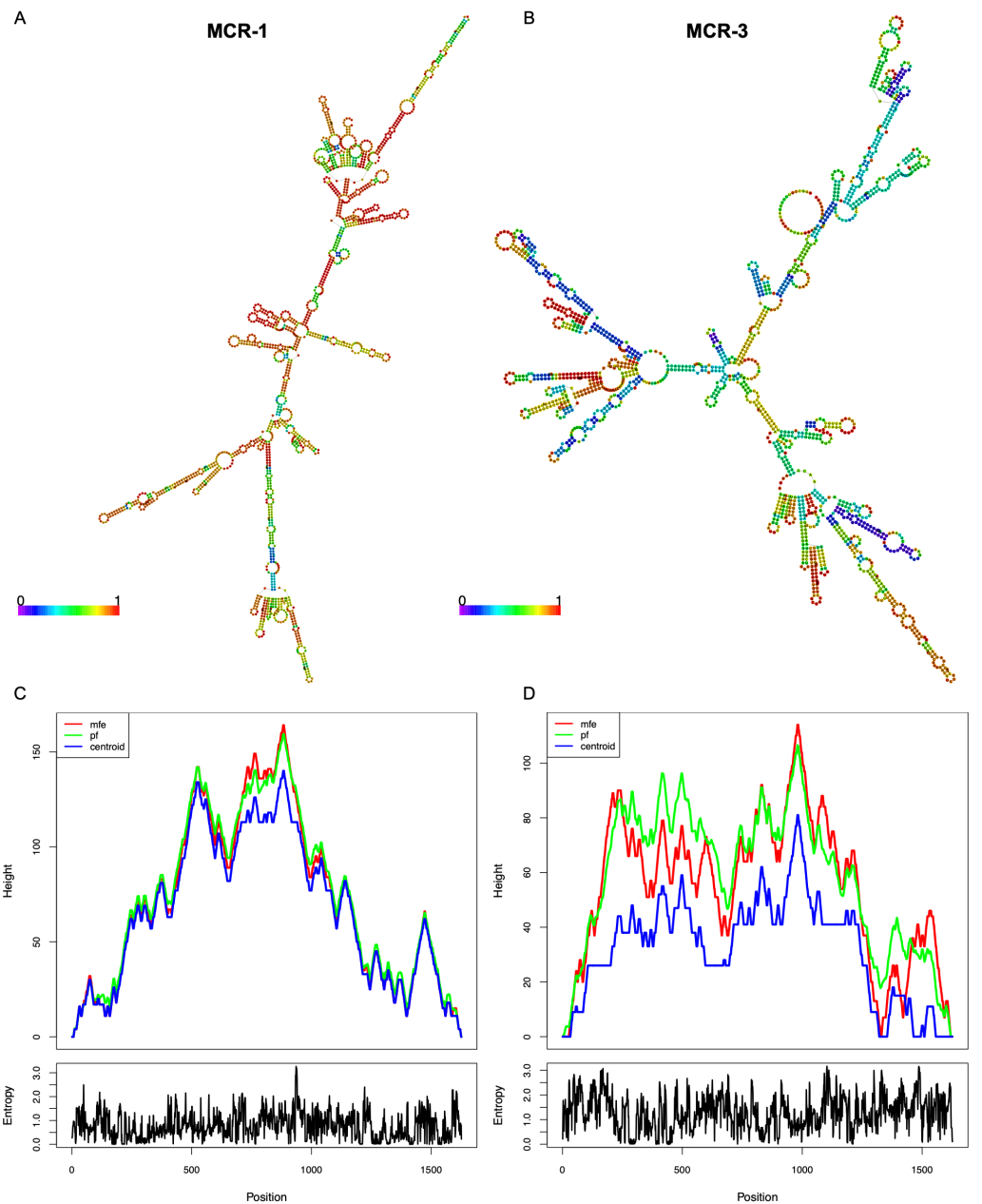


**Figure S15. The predicted mRNA secondary structure of *mcr-1* and *mcr-3*.**

**(A-B)** The mRNA secondary structure of *mcr-1* and *mcr-3* in the term of minimum free energy (MFE);

**(C-D)** The mountain plot representation of the MFE structure, the thermodynamic ensemble of RNA structures, and the centroid structure.

The above results were analysed and plotted using RNAFold. Base pair probability ranged from 0 to 1. Larger probability indicates higher quality of secondary structure prediction.


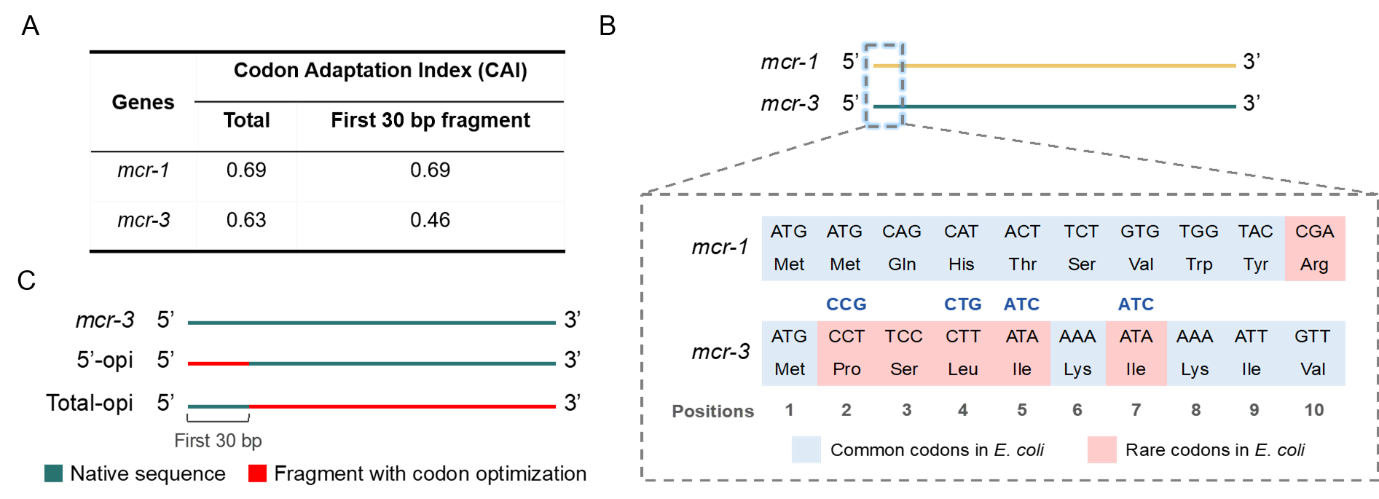


**Figure S16. Codon optimisation upon *mcr-3* through Synonymous mutations.**

**(A)** Codon adaptation index of *mcr-1* and *mcr-3.*

**(B)** Localization of codons in low usage frequency at the first 30 bp fragment of *mcr-1*/*mcr-3*. Codons coloured in blue represents synonymous mutations for codon optimisation at the 5’-end of *mcr-3*.

**(C)** Construction of *mcr-3* variants with codon optimisation at different segments of the gene. 5’-opi represents the *mcr-3* variant with codon optimisation at the first 30 bp fragment of the gene, and total-opi represents the *mcr-3* variant with codon optimisation at the remaining fragment of the gene.


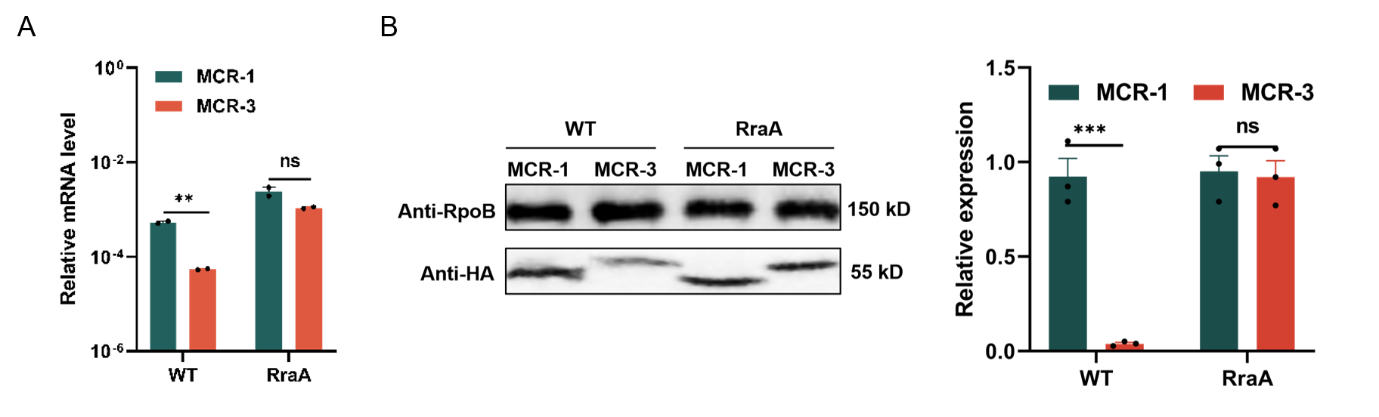


**Figure S17. Impact of RraA upon *mcr-1*/*mcr-3*-positive strains.**

**(A)** The mRNA transcript levels of *mcr-1* and *mcr-3* in *E. coli* BW25113 overexpressing RraA were determined by q‒PCR, normalized to the transcript level of the housekeeping gene *rpoB* and quantified by ΔΔCT analysis.

**(B)** Determination of protein expression levels of MCR-1 or MCR-3 in *E. coli* BW25113 with overexpression of RraA through western blot. HA-tag was added at the C-terminal of indicated proteins.

All the above-described experiments were performed thrice with similar results. Error bars indicate standard errors of the means (SEMs) for three biological replicates. A two-tailed unpaired *t* test was performed to determine the statistical significance of the data. ns, no significant difference; **, *P*< 0.01; ***, *P*< 0.001.


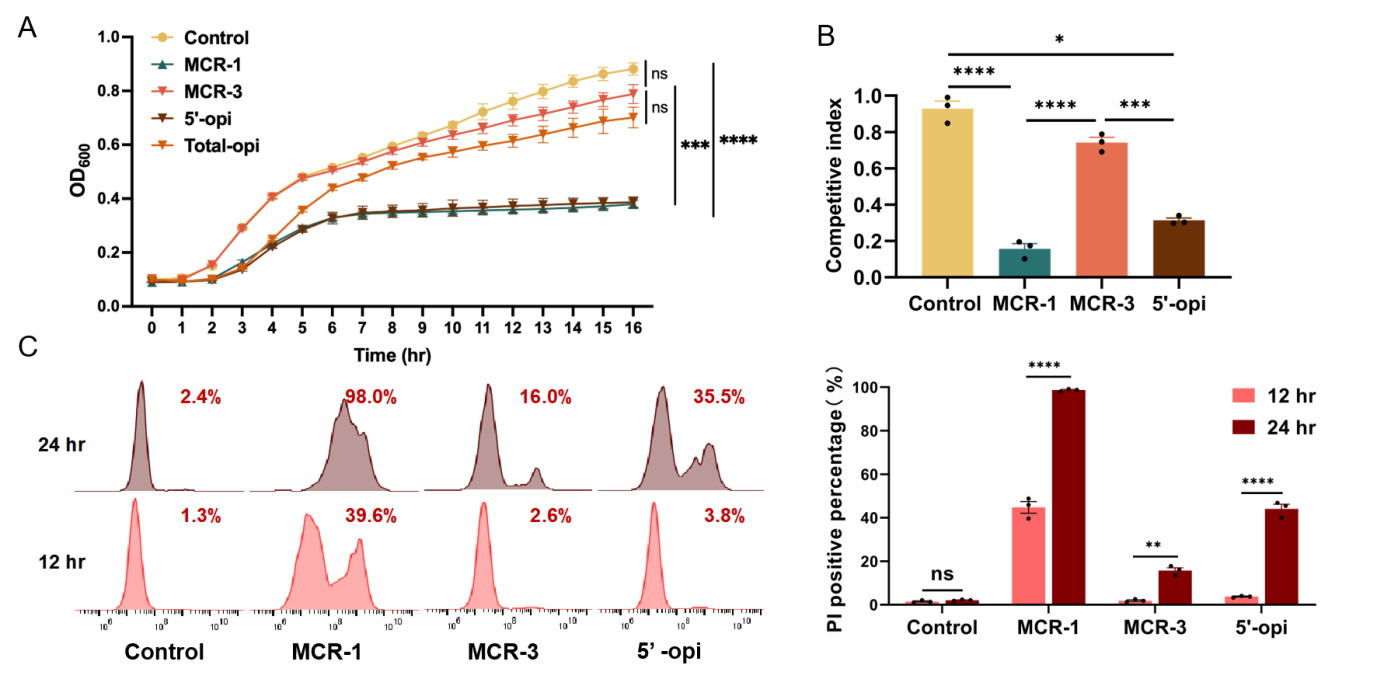


**Figure S18. Impact of 5’-end codon optimality upon membrane permeability and fitness of bacterial host.**

**(A)** Measurement of growth curves of *E. coli* BW25113 carrying an empty vector, *mcr-1*, *mcr-3* or its variants. The y-axis shows the optical density at a wavelength of 600 nm (OD_600_) of the broth cultures, and the x-axis shows the period of growth (h).

**(B)** Fitness evaluation of *E. coli* BW25113 carrying *mcr-1*, *mcr-3* or its variants through *in vitro* competitive assay. The cultures of indicated strains and GFP-positive control strain were mixed in the ratio of 1:1. Samples were collected at 0 h and 24 h after initiation. And the percentage of target strains were determined by flow cytometry and analysed by FlowJo (version 10) software. The y-axis represents the value of log_10_(percentage of GFP negative population/ percentage of GFP positive population), and the x-axis represents period of growth (h). The representative result is shown as **(B)**. The GFP-negative population represents the indicated strains for analysis, while the GFP-positive population represents the control strains.

**(C)** Determination of membrane integrity. The membrane permeability of *E. coli* carrying *mcr-1*, *mcr-3* or its variants were evaluated by PI staining assay. Overnight cultures were sub-cultured into fresh LB broth in the ratio of 1:100 and induced with 0.2% arabinose to express MCR-1 or MCR-3. Samples were collected at 12 h and 24 h after initiation, followed by staining with PI dye for 15 min. The PI-positive proportion was determined by flow cytometry and analysed by FlowJo (version 10) software.

All the above-described experiments were performed thrice with similar results. Error bars indicate standard errors of the means (SEMs) for three biological replicates. A two-tailed unpaired *t* test and one-way ANOVA analysis were performed to determine the statistical significance of the data. ns, no significant difference; *, *P*< 0.1; **, *P*< 0.01; ***, *P*< 0.001; ****, *P*< 0.0001.

**
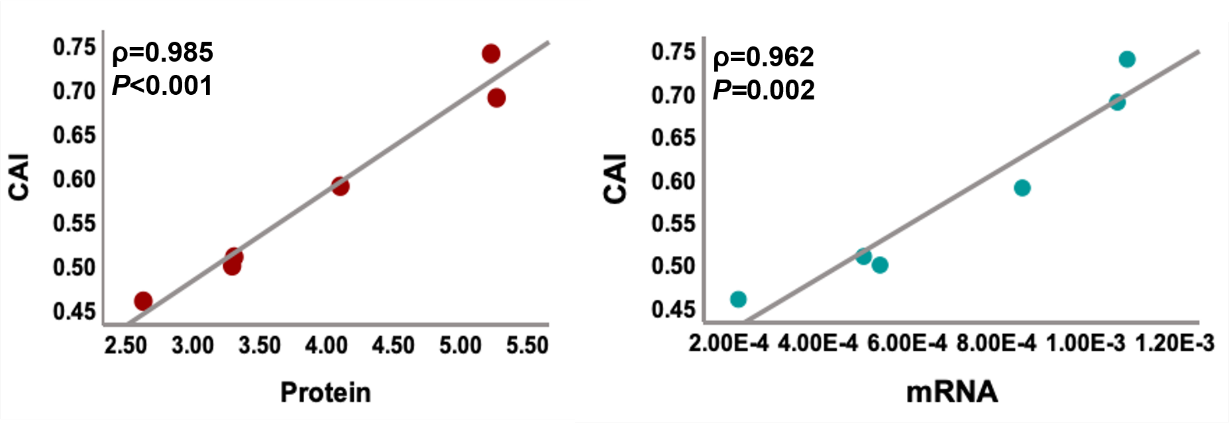
**

**Figure S19. Correlation analysis between the CAI and the mRNA/protein level in *mcr-1-* or *mcr-3*-positive *E. coli* BW25113.**

Evaluating the correlation between the 5’-end codon adaptation index and the mRNA/protein level in *mcr-1-* or *mcr-3*-positive *E. coli* BW25113. ρ is Spearman’s rank correlation coefficient. The related *P* values and regression lines are shown.

**
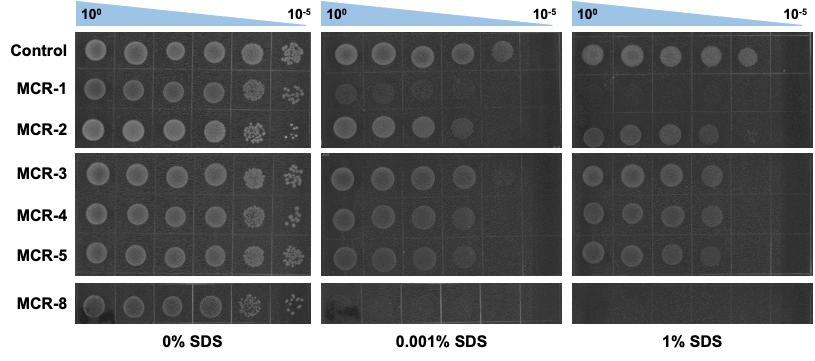
**

**Figure S20. SDS-EDTA sensitivity of *mcr*-positive *E. coli* BW25113.**

Fresh cultures of *E. coli* BW25113 bearing empty vector, *mcr-1*, *mcr-2*, *mcr-3*, *mcr-4*, *mcr-5* or *mcr-8* were spotted on LB agar plates containing 0.001% SDS and 1 mM EDTA or % SDS and 1 mM EDTA. Ten-fold serial-dilution of indicated cultures were inoculated onto the agar plates.


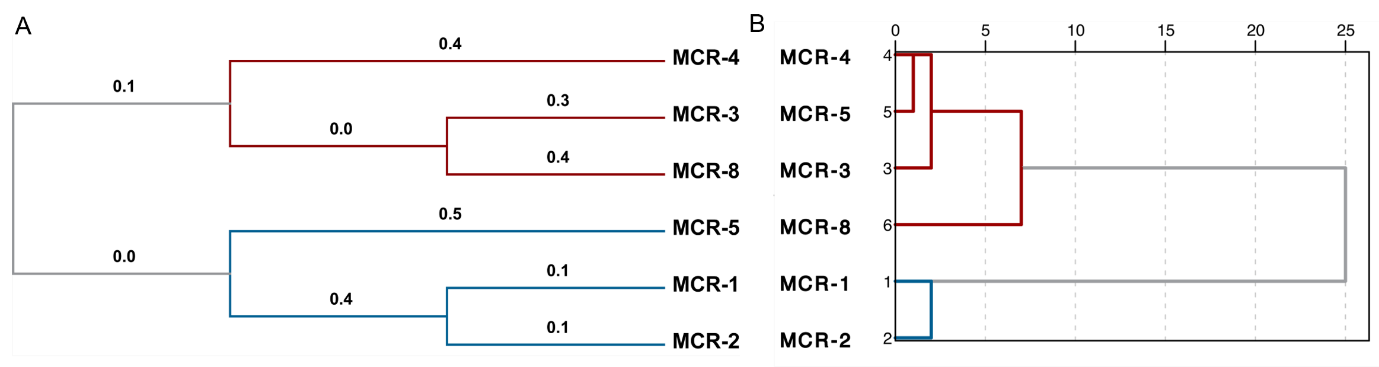


**Figure S21. Phylogenetic tree and cluster analysis of *mcr* family genes.**

**(A)** Phylogenetic tree derived for *mcr* family genes by the neighbour‒joining method. All the *mcr* genes used here were sampled from the gene database of the NCBI website and subjected to MEGA (version 11) for analysis.

**(B)** Cluster analysis of *mcr* family genes based on the 5’-end codon adaptation index, which represents the relationship between *mcr* genes and *mcr* clusters on the basis of the 5’-end codon usage frequency.

A phylogenetic tree of the *mcr* family genes was constructed with MEGA (version 11). Correlation analysis and cluster analysis were performed with SPSS (version 29.0.1.0).


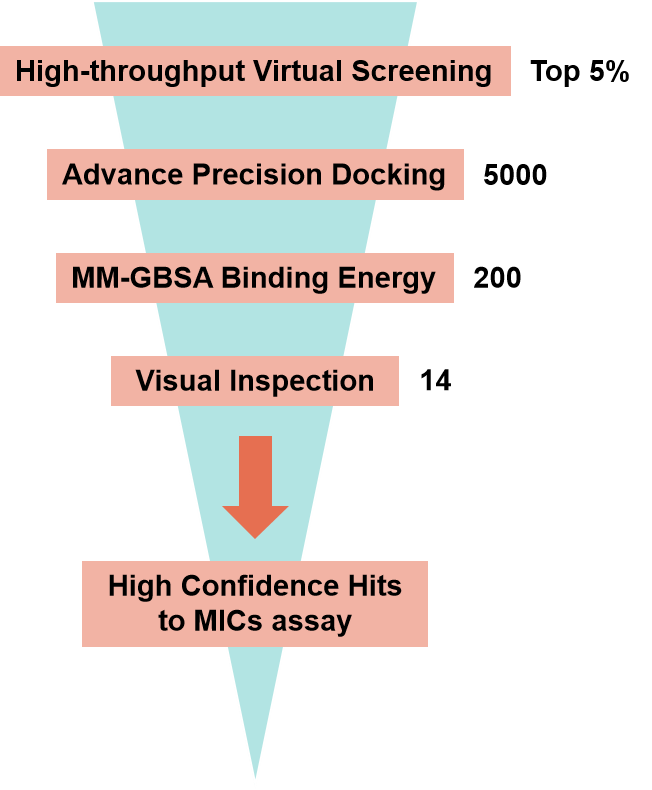


**Figure S22. Flow chart of screening MCR inhibitors.**

Flow chart illustrating the process of screening MCR-1- and MCR-3-specific inhibitors. The values represent the numbers of inhibitors.


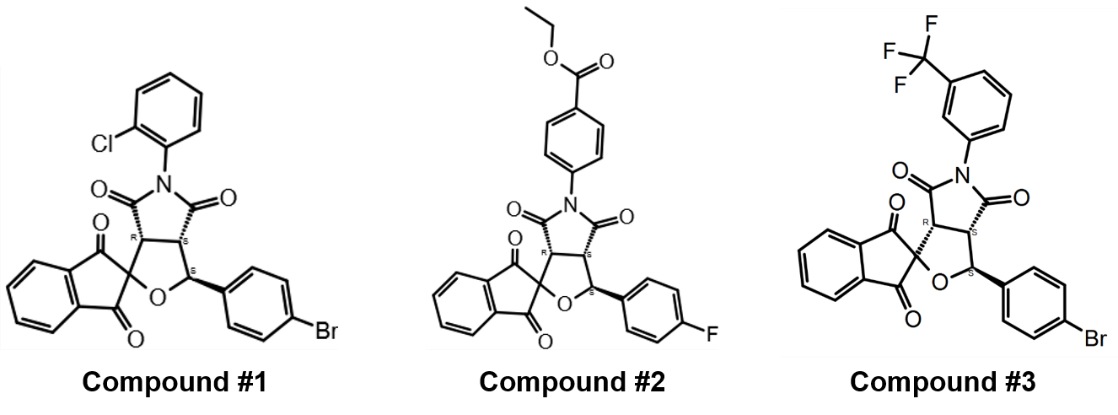


**Figure S23. Molecular structure of MCR inhibitors.**

The molecular structure of compounds #1, #2 and #3 that targeted at the putative lipid A binding pocket of MCR-1 and MCR-3.


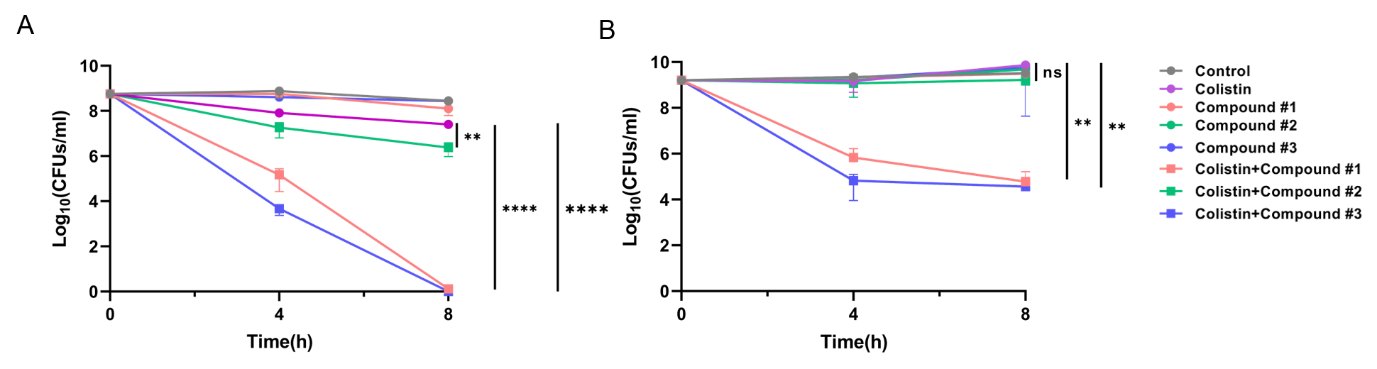


**Figure S24. Time killing curve of MCR inhibitors combined with colistin against *mcr-1*- or *mcr-3* positive *E. coli* BW25113**

**(A)** Viability of *mcr-3*-bearing *E. coli* challenged with colistin with/without the indicated compounds (#1, #2 and #3). An overnight culture of the *mcr-3*-positive strain was subcultured in fresh LB broth at a ratio of 1:100 and induced with 0.2% arabinose to express MCR-3. Exponential phase cultures were then collected and challenged with the indicated compounds. After treatment for 4 h and 8 h, bacterial viability was determined by CFU assays. Colistin was used at a 1*MIC (16 μg/mL), and the concentration of each inhibitor was 100 μM.

**(B)**The same assay was also performed to evaluate the combined bactericidal effect against *E. coli* harbouring *mcr-1*.

All the experiments described above were performed three times with similar results. The error bars indicate the standard errors of the means (SEMs) for three biological replicates. A two-tailed unpaired t test was performed to determine the statistical significance of the data. ns, no significant difference; **, P< 0.01; ****, P< 0.0001.


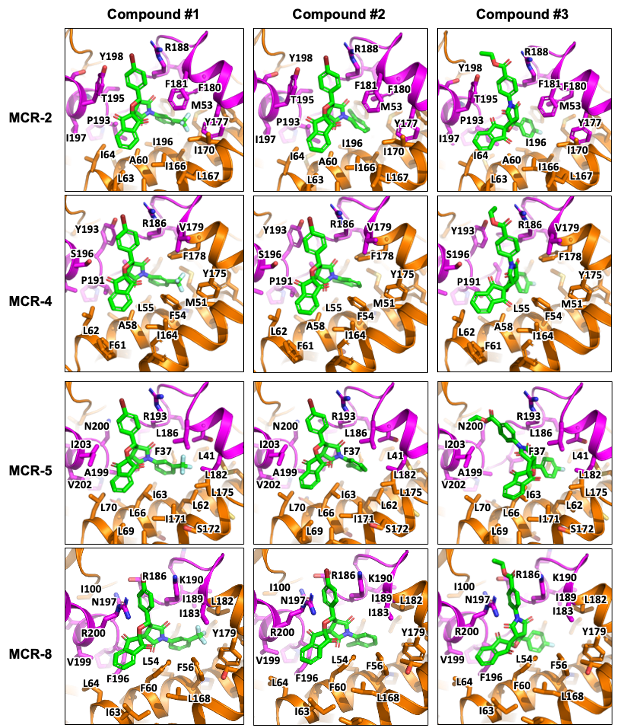


**Figure S25. Interaction between MCR inhibitors and MCR proteins.**

The protein structure of putative lipid A binding pocket of MCR in complex with indicated inhibitors (compounds #1, #2 and #3). The catalytic domain, linker domain and transmembrane domain of the MCR proteins are in cyan, magenta and orange, respectively.

**Figure S26. Schematic graph establishing the mechanism regulating MCR-3 fitness effect.**

The 5’-end codon optimality appeared as a crucial regulator that impact the fitness effect of MCR-1 and MCR-3 by controlling protein expression level. Comparing with *mcr-1*, more codons with low adaptation index cluster at the 5’-end mRNA coding region of *mcr-3*. Such feature results in low translational level of MCR-3, while the protein expression level of MCR-1 is higher, which is related with the increased membrane permeability of bacterial host.

PI represents propidium Iodide. The enhanced blocking against PI entrance in *mcr-3*-bearing *E. coli* is highlighted in red.

**
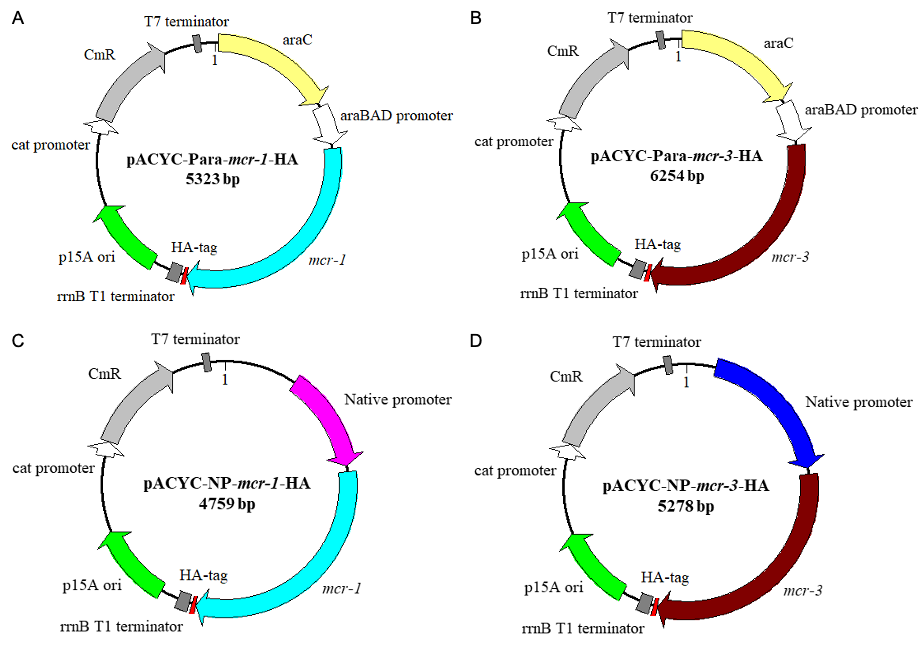
**

**Figure S27. Plasmid maps of pACYC-Para/NP-MCR-1-HA and pACYC-Para/NP-MCR-3-HA.**

The plasmids for expressing MCR-1 or MCR-3 under the regulation of arabinose promoter were shown as **(A)** and **(B)**, and the plasmids for expressing target proteins under the regulation of MCR-1/MCR-3 native promoter was shown as **(C)** and **(D)**. The elements in each plasmid were as follows: CmR, chloramphenicol acetyltransferase; cat promoter, promoter regulating the expression of chloramphenicol acetyltransferase; p15A ori, the medium-copy-number p15A origin of replication; T7 terminator, transcription terminator for bacteriophage T7 RNA polymerase; araC, gene encoding L-arabinose regulatory protein; araBAD promoter, promoter of the L-arabinose operon; native promoter, the native promoters of *mcr-1* or *mcr-3* genes amplified from clinically collected strain.


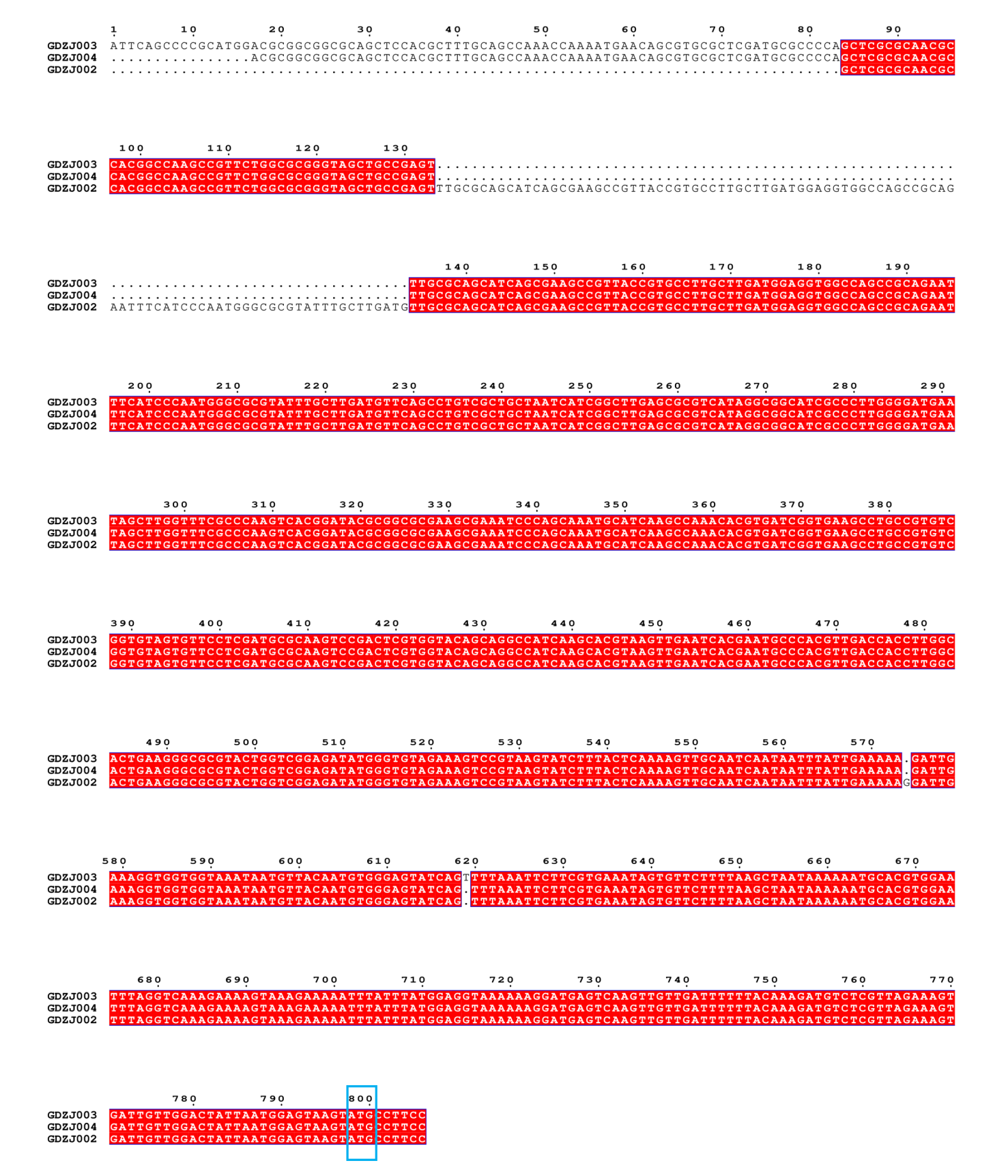


**Figure S28. Sequence alignment of *mcr-3* promoter region.**

Sequence alignment of three DNA sequences encoding the promoter region of *mcr-3*, namely GDZJ002, GDZJ003 and GDZJ004. The conserved segments are highlighted in red, and the position of start codon are indicted (blue rectangle).

**Supplementary tables**

| ***E. coli* strains** | **Colistin MICs (μg/mL)** |
| --- | --- |
| ATCC 25922 | 0.5 |
| BW25113 | 0.5 |
| BW25113-Vector | 0.5 |

**Table S1. Colistin MICs of quality control strain *E. coli* ATCC25922 and BW25113.**


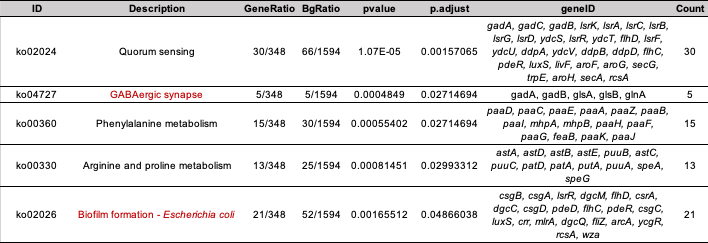


**Table S2. KEGG enrichment of transcriptomic analysis.**

The KEGG enrichment in the term of biological processes between MCR-3-positive *E. coli* and MCR-1-expressing *E. coli*.

| **Index** | ***mcr-1*** | ***mcr-3*** |
| --- | --- | --- |
| Free energy of the thermodynamic ensemble | -549.70 kcal/mol | -456.39 kcal/mol |
| Frequency of the MFE structure | 0.00 % | 0.00 % |
| Ensemble diversity | 296.77 | 504.39 |

**Table S4. mRNA secondary structure of *mcr-1* and *mcr-3*.**

Prediction of mRNA secondary structure upon *mcr-1* and *mcr-3* using RNAfold.

| ***E. coli* strains** | **Colistin MICs (μg/mL)** |
| --- | --- |
| Control | 0.5 |
| MCR-1 | 16 |
| MCR-3 | 16 |
| MCR-3 5’-opi | 32 |

**Table S5. Colistin MICs of *E. coli* expressing *mcr-3* 5’-opi^+^.**

Colistin MICs was determined for *E. coli* BW25113 expressing MCR-3 with 5’-end codons optimisation.


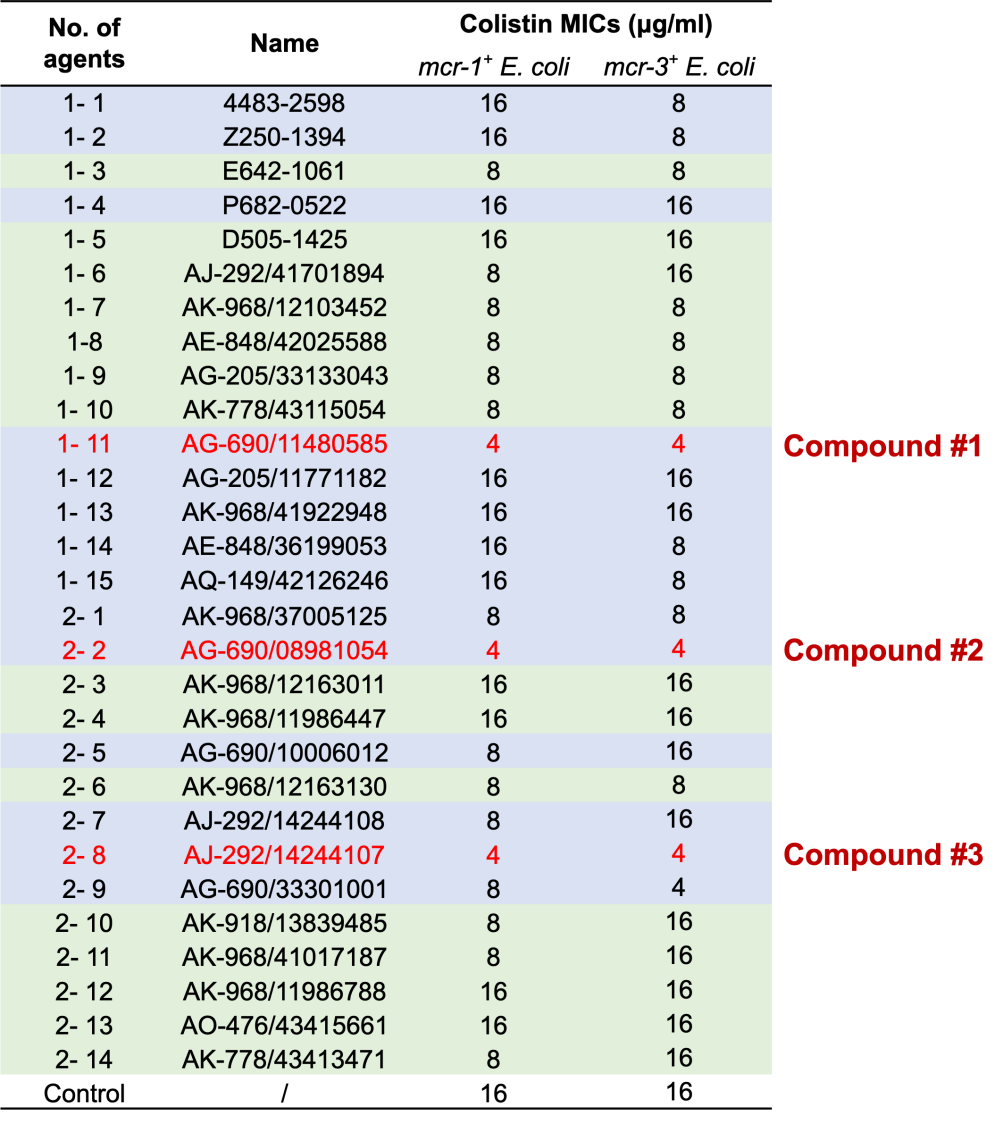


**Table S6. Screening of effective inhibitor targeting at MCR-1 and MCR-3 from small molecule library.**

The compounds target at putative lipid A pocket of MCR-1 or MCR-3 are highlighted in blue, while green for those targeting at catalytic domain. The compounds selected for study are in red, namely compounds #1, #2 and #3. The concentration of each compound for MICs assay was 100 μM.

| Inhibitors | MICs of colistin (μg/mL) | | | |
| --- | --- | --- | --- | --- |
|  | CT^R^ clinical isolate 1 | CT^R^ clinical isolate 2 | CT^R^ clinical isolate 3 | CT^R^ clinical isolate 4 |
| #1 | 128 | 32 | 32 | 128 |
| #2 | 128 | 32 | 32 | 128 |
| #3 | 128 | 32 | 32 | 128 |
| Control | 128 | 32 | 32 | 128 |

**Table S7. Colistin MICs of *mcr* negative CT^R^ *E. coli* collected from clinic with the supplement of target inhibitors.**

The concentrations of the selected MCR inhibitors (compounds #1, #2 and #3) were 100 μM. Treatment without the addition of inhibitors was set as control.

| Inhibitors | MICs of colistin (μg/mL) | | |
| --- | --- | --- | --- |
|  | *mcr-1*^+^ *E. coli* BW25113 | *mcr-3*^+^ *E. coli* BW25113 | Empty control |
| 1-11 | 4 | 4 | 0.5 |
| 2-2 | 4 | 4 | 0.5 |
| 2-8 | 4 | 4 | 0.5 |
| Control | 16 | 16 | 0.5 |

**Table S8. Colistin MICs of *mcr-1-* or *mcr-3*-positive *E. coli* with the supplement of MCR inhibitors.**

The concentrations of the selected MCR inhibitors were 100 μM. Empty control represents *E. coli* BW25113 carrying pACYCDuet-1 empty plasmid. Treatment without the addition of inhibitors was set as control.

| Inhibitors | MICs of MCR inhibitors (μM) | | |
| --- | --- | --- | --- |
|  | *mcr-1*^+^ *E. coli* BW25113 | *mcr-3*^+^ *E. coli* BW25113 | Empty control |
| #1 | >400 | >400 | >400 |
| #2 | >400 | >400 | >400 |
| #3 | >400 | >400 | >400 |

**Table S9. Inhibitors MICs of *mcr-1-* or *mcr-3*-positive *E. coli*.**

Empty control represents *E. coli* BW25113 carrying pACYCDuet-1 empty plasmid.

**Table S3. Differentially transcription analysis (MCR-3 vs. MCR-1).**

**Table S10. Bacterial strains used in this study.**

**Table S11.** **Primers used in this study.**

**Table S12. Plasmids used in this study.**
