# Supplementary material for: Fitness costs of mobilised colistin resistance gene 3 (mcr-3): systematic review, epidemiological study, and functional analysis
Source: eBioMedicine. 2025 Sep 12;120:105923. doi: 10.1016/j.ebiom.2025.105923 (PMC12571581; doi:10.1016/j.ebiom.2025.105923)
Supplement: Supplementary Table S10 [file mmc3.pdf]

Table S10. Bacterial strains used in this study.

Bacterial strains	Description	Reference/source
E.coli DH5α	Type strain	Our lab
E.coli BW25113	Type strain	Our lab
E.coli MG1655	Type strain	Our lab
E.coli C600	Type strain	Our lab
E.coli ATCC25922	Type strain	Our lab
E.coli C600 SYSU0050	C600 conjugant harbouring *mcr-1* -positive plasmid isolated from	Our lab
E.coli C600 SYSU0060	C600 conjugant harbouring *mcr-1* -positive plasmid isolated from	Our lab
E.coli C600 SYSU0062	C600 conjugant harbouring *mcr-1* -positive plasmid isolated from	Our lab
E.coli C600 GDZJ002	C600 conjugant harbouring *mcr-3* -positive plasmid isolated from	Our lab
E.coli C600 GDZJ003	C600 conjugant harbouring *mcr-3* -positive plasmid isolated from	Our lab
E.coli C600 GDZJ004	C600 conjugant harbouring *mcr-3* -positive plasmid isolated from	Our lab
CT^R clinical isolate 1	*mcr* -negative colistin-resistant clinical isolate	Our lab
CT^R clinical isolate 2	*mcr* -negative colistin-resistant clinical isolate	Our lab
CT^R clinical isolate 3	*mcr* -negative colistin-resistant clinical isolate	Our lab
CT^R clinical isolate 4	*mcr* -negative colistin-resistant clinical isolate	Our lab
