# Supplementary material for: Fitness costs of mobilised colistin resistance gene 3 (mcr-3): systematic review, epidemiological study, and functional analysis
Source: eBioMedicine. 2025 Sep 12;120:105923. doi: 10.1016/j.ebiom.2025.105923 (PMC12571581; doi:10.1016/j.ebiom.2025.105923)
Supplement: Supplementary Table S11 [file mmc4.pdf]

**Table S11. Primers used in this study.**

| Primers | Description | Reference/source |
|---|---|---|
| MCR3-F | TTGGCACTGTATTTTGCATTT | Our lab |
| MCR3-R | TTAACGAAATTGGCTGGAACA | Our lab |
| ParaBAD-*mcr-3*-R | GAATTCCTCCTGCTAGCCCAA | This study |
| ParaBAD-*mcr-3*-F | TTGGGCTAGCAGGAGGAATTCATGCCTTCCCTTATAAAAATAAAAATT | This study |
| pBAD24-RraA-F | TGGGCTAGCAGGAGGAATTCATGAAATACGATACTTCCGAGCTTT | This study |
| pBAD24-R | GAATTCCTCCTGCTAGCCCAA | This study |
| RraA-pBAD24-R | CCAAAACAGCCAAGCTTTCATCATTCAATATCCAGCGGATCTT | This study |
| pBAD24-F | TGAAAGCTTGGCTGTTTTGGC | This study |
| pACYC-*mcr-3* opi-F | GTTTTTTTGAGTAGTTTCTCATGCCGAGCCTGATTAAAATTAA | This study |
| pACYC-R | GAGAAACTACTCAAAAAAACGGGTATG | This study |
| *mcr-3* opi-pACYC-R | TTGACTGGCCGTCGTTTTACTTACGCATAATCCGGCACATC | This study |
| pACYC-F | GTAAAACGACGGCCAGTCAAA | This study |
| *mcr-3* opi qpcr-F | GTGTATAACCGCTATCTGGC | This study |
| *mcr-3* opi qpcr-R | ACATACATGGAACGCTCACCGCA | This study |
| *mcr-3*-qpcr-F | ACCTCCAGCGTGAGATTGTTCCA | This study |
| *mcr-3*-qpcr-R | GGATTGGTGTCTTTCTCATAGCC | This study |
| *mcr-1*-qpcr-F | ATATGCTGATCATGCTGCACC | This study |
| *mcr-1*-qpcr-R | ATTGCTGTGCGTCTGTAGCCA | This study |
| MCR-3 NP-F | ATTGTCTGATTCGTTACCAACCTTATTCAGCCCCGCATG | This study |
| MCR-3 NP-R | ATTTTTATAAGGGAAGGCATACTTACTCCATTAATAGTCCAACAATCAC | This study |
| pACYC-MCR-3-F | ATGCCTTCCCTTATAAAAATAAAAATT | This study |
| pACYC-MCR-3-R | TTGGTAACGAATCAGACAATTGACG | This study |
| *mcr-3* 5'-opi-F | TGGGCTAGCAGGAGGAATTCATGCCATCACTTATTAAAATTAAAATTG | This study |
| *mcr-3* 5'-opi-R | CAACGTGGGCTTACTTTGATTAGTATCCCGTTTTGCATCATCA | This study |
| *mcr-3*-R | ATCAAAGTAAGCCCACGTTGATG | This study |
| pACYC plasmid-F | GAATTCCTCCTGCTAGCCCAA | This study |
| *mcr-3* R187A-F | CTCCAGGCCGAGATTGTTCCAGCCAATTTCGTTAATAGTACC | This study |
| *mcr-3* R187A-R | GAAATTGGCTGGAACAATCTCGGCCTGGAGGTTTGAATTGTT | This study |
| MCR-3 R180A F | AGTGGGGGCCAACAATTCAAACCTCCAGCGTGAGATTGTTCCAGCCA | This study |
| MCR-3 R180A R | TTTGAATTGTTGGCCCCCACTGACACATAATCTTGATAGTATAGT | This study |
| MCR-3 N184A F | ACAATTCAGCCCTCCAGCGTGAGATTGTTCCAGCCAATTTCGTTA | This study |
| MCR-3 N184A R | CACGCTGGAGGGCTGAATTGTTGCGCCCCACTGACACATAATCTT | This study |
| MCR-3 Q186A F | AACCTCGCCCGTGAGATTGTTCCAGCCAATTTCGTTAATAGTACC | This study |
| MCR-3 Q186A R | GAACAATCTCACGGGCGAGGTTTGAATTGTTGCGCCCCACTGACA | This study |
| MCR-3 N196A F | TTTCGTTGCCAGTACCGTTAAATACGTTTACAATCGTTATCTCGC | This study |
| MCR-3 N196A R | ATTTAACGGTACTGGCAACGAAATTGGCTGGAACAATCTCACGCT | This study |
| MCR-3 K200A F | GTACCGTTGCCTACGTTTACAATCGTTATCTCGCTGAACCAATCC | This study |
| MCR-3 K200A R | CGATTGTAAACGTAGGCAACGGTACTATTAACGAAATTGGCTGGA | This study |
| *mcr-3* N59A-F | CAGCGCTTGCATTTGTATTTGTTCCATTTTCGATACG | This study |
| *mcr-3* N59A-R | TACAAATGCAAGCGCTGCAACAAGCAATATTG | This study |
| *mcr-3* Y203A-F | CGTTGCAAATCGTTATCTTGCTGAACCAATCC | This study |
| *mcr-3* Y203A-R | GATAACGATTTGCAACGTATTTAACGGTACTATTAACGAAAT | This study |
