# Supplementary material for: Fitness costs of mobilised colistin resistance gene 3 (mcr-3): systematic review, epidemiological study, and functional analysis
Source: eBioMedicine. 2025 Sep 12;120:105923. doi: 10.1016/j.ebiom.2025.105923 (PMC12571581; doi:10.1016/j.ebiom.2025.105923)
Supplement: Supplementary Table S12 [file mmc5.pdf]

Table S12. Plasmids used in this study.

Plasmids	Description	Reference/source
pACYCDuet-1	Vector carrying the P15A replicon, *lacI* gene and chloramphenicol resistance	Addgene #71147
pBAD24	A tightly controlled expression vectors regulated by the arabinose operon, carrying pBR322 replicon and ampicillin gene.	Our lab
pACYC-EGFP	pACYCDuet-1 derivative harbouring EGFP under the regulation of JW25113 promoter.	Our lab
pACYC-Para-*mcr-1* -HA	pACYCDuet-1 derivative replacing T7 promoter with arabinose promoter and carrying wide type *mcr-1* gene. Sequence encoding HA-tag is added at 3'-end.	Our lab
pACYC-Para-*mcr-3* -HA	pACYCDuet-1 derivative replacing T7 promoter with arabinose promoter and carrying wide type *mcr-3* gene. Sequence encoding HA-tag is added at 3'-end.	This study
pACYC-Para-*mcr-2* -HA	pACYCDuet-1 derivative replacing T7 promoter with arabinose promoter and carrying wide type *mcr-2* gene. Sequence encoding HA-tag is added at 3'-end.	This study
pACYC-Para-*mcr-4* -HA	pACYCDuet-1 derivative replacing T7 promoter with arabinose promoter and carrying wide type *mcr-4* gene. Sequence encoding HA-tag is added at 3'-end.	This study
pACYC-Para-*mcr-5* -HA	pACYCDuet-1 derivative replacing T7 promoter with arabinose promoter and carrying wide type *mcr-5* gene. Sequence encoding HA-tag is added at 3'-end.	This study
pACYC-Para-*mcr-8* -HA	pACYCDuet-1 derivative replacing T7 promoter with arabinose promoter and carrying wide type *mcr-8* gene. Sequence encoding HA-tag is added at 3'-end.	This study
pACYC-Para-*mcr-3* -HA 5'-opi	pACYCDuet-1 derivative replacing T7 promoter with arabinose promoter and carrying wide type *mcr-3* gene with codon optimization at 5'-end.	This study
pACYC-Para-*mcr-3* -HA opi	pACYCDuet-1 derivative replacing T7 promoter with arabinose promoter and carrying wide type *mcr-3* gene with codon optimization.	This study
pACYC-Para-*mcr-3* N69A	pACYCDuet-1 derivative replacing T7 promoter with arabinose promoter and carrying *mcr-3* gene with mutation N69A.	This study
pACYC-Para-*mcr-3* R180A	pACYCDuet-1 derivative replacing T7 promoter with arabinose promoter and carrying *mcr-3* gene with mutation R180A.	This study
pACYC-Para-*mcr-3* N184A	pACYCDuet-1 derivative replacing T7 promoter with arabinose promoter and carrying *mcr-3* gene with mutation N184A.	This study
pACYC-Para-*mcr-3* Q186A	pACYCDuet-1 derivative replacing T7 promoter with arabinose promoter and carrying *mcr-3* gene with mutation Q186A.	This study
pACYC-Para-*mcr-3* R187A	pACYCDuet-1 derivative replacing T7 promoter with arabinose promoter and carrying *mcr-3* gene with mutation R187A.	This study
pACYC-Para-*mcr-3* N196A	pACYCDuet-1 derivative replacing T7 promoter with arabinose promoter and carrying *mcr-3* gene with mutation N196A.	This study
pACYC-Para-*mcr-3* K200A	pACYCDuet-1 derivative replacing T7 promoter with arabinose promoter and carrying *mcr-3* gene with mutation K200A.	This study
pACYC-Para-*mcr-3* Y203A	pACYCDuet-1 derivative replacing T7 promoter with arabinose promoter and carrying *mcr-3* gene with mutation Y203A.	This study
pACYC-Para-*mcr-3* N184A+K200A	pACYCDuet-1 derivative replacing T7 promoter with arabinose promoter and carrying *mcr-3* gene with mutation N184A+K200A.	This study
pACYC-Para-*mcr-3* R187A+K200A	pACYCDuet-1 derivative replacing T7 promoter with arabinose promoter and carrying *mcr-3* gene with mutation R187A+K200A.	This study
pACYC-Para-*mcr-3* N184A+R187A	pACYCDuet-1 derivative replacing T7 promoter with arabinose promoter and carrying *mcr-3* gene with mutation N184A+R187A.	This study
pACYC-Para-*mcr-3* N184A+R187A+K200A	pACYCDuet-1 derivative replacing T7 promoter with arabinose promoter and carrying *mcr-3* gene with mutation N184A+R187A+K200A.	This study
pACYC-NP-*mcr-1* -HA	pACYCDuet-1 derivative replacing T7 promoter with the native promoter of *mcr-1* and carrying wide type *mcr-1* gene. Sequence encoding HA-tag is added at 3'-	Our lab
pACYC-NP-*mcr-3* -HA	pACYCDuet-1 derivative replacing T7 promoter with the native promoter of *mcr-3* and carrying wide type *mcr-3* gene. Sequence encoding HA-tag is added at 3'-	This study
pBAD24-RraA	pBAD24 derivative carrying *rraA* .	This study
