# Supplementary material for: Fitness costs of mobilised colistin resistance gene 3 (mcr-3): systematic review, epidemiological study, and functional analysis
Source: eBioMedicine. 2025 Sep 12;120:105923. doi: 10.1016/j.ebiom.2025.105923 (PMC12571581; doi:10.1016/j.ebiom.2025.105923)
Supplement: Supplementary Table S3 [file mmc6.pdf]

Table S3. Differentially transcription analysis (MCR-3 vs. MCR-1)

Gene_ID	log2FC	PValue	Gene_Name
gene-b2055	-7.948151361	8.30E-170	*wcaE*
gene-b4028	-7.536722365	3.94E-41	*yjbG*
gene-b2062	-7.154653056	8.89E-173	*wza*
gene-b2052	-7.117738227	2.95E-220	*fcl*
gene-b2053	-6.998598999	1.73E-271	*gmd*
gene-b3263	-6.954047648	6.91E-13	*yhdU*
gene-b2061	-6.837001846	3.97E-79	*wzb*
gene-b2054	-6.717725354	1.19E-101	*wcaF*
gene-b4026	-6.430074508	2.19E-191	*yjbE*
gene-b2059	-6.34579572	3.82E-180	*wcaA*
gene-b4027	-6.12553687	4.64E-66	*yjbF*
gene-b2051	-6.086430519	1.77E-102	*gmm*
gene-b2058	-5.987269905	4.97E-88	*wcaB*
gene-b3517	5.880877717	1.74E-143	*gadA*
gene-b2057	-5.875313122	3.86E-174	*wcaC*
gene-b2060	-5.856421535	3.03E-193	*wzc*
gene-b2048	-5.80618554	4.43E-178	*cpsG*
gene-b2056	-5.783492713	9.70E-172	*wcaD*
gene-b2050	-5.772450227	4.11E-146	*wcaI*
gene-b2851	5.729500683	8.14E-06	*ygeG*
gene-b4184	5.586637353	2.82E-05	*yjfL*
gene-b2045	-5.533977278	3.63E-179	*wcaK*
gene-b2049	-5.473385369	2.73E-195	*cpsB*
gene-b1166	5.376729241	6.03E-48	*ariR*
gene-b2046	-5.111001219	3.46E-122	*wzxC*
gene-b0331	5.058940887	2.32E-58	*prpB*
gene-b0333	5.035895023	7.90E-48	*prpC*
gene-b2047	-5.011163323	4.17E-122	*wcaJ*
gene-b0334	4.888127489	1.12E-53	*prpD*
gene-b1115	-4.820665895	1.56E-73	*ycfT*
gene-b1259	4.799687216	1.06E-113	*yciG*
gene-b2044	-4.781189978	7.26E-159	*wcaL*
gene-b1492	4.760243996	1.02E-182	*gadC*
gene-b3510	4.752196844	6.52E-172	*hdeA*
gene-b4707	-4.743550146	8.97E-05	*esrE*
gene-b2043	-4.645125913	1.94E-128	*wcaM*
gene-b3511	4.567725545	1.05E-172	*hdeD*
gene-b3509	4.505360783	1.08E-180	*hdeB*
gene-b1493	4.41248785	8.85E-111	*gadB*
gene-b3512	4.388823609	1.98E-65	*gadE*
gene-b1474	-4.381124795	6.55E-28	*fdnG*
gene-b1041	4.202927234	4.18E-25	*csgB*
gene-b1257	4.189099042	8.47E-24	*yciE*
gene-b2252	4.147177872	2.06E-06	*ais*
gene-b3491	4.107201957	1.63E-119	*yhiM*
gene-b1475	-3.986130622	1.57E-20	*fdnH*
gene-b4199	3.935540285	1.64E-33	*yjfY*
gene-b1258	3.935504398	4.97E-24	*yciF*
gene-b3563	-3.920719298	8.04E-08	*yiaB*
gene-b2028	-3.856482868	3.32E-134	*ugd*
gene-b4045	3.854392451	3.23E-126	*yjbJ*
gene-b0485	3.823234731	6.95E-96	*glsA*
gene-b3513	3.791186985	1.40E-96	*mdtE*
gene-b1165	3.778878097	6.49E-10	*ymgA*
gene-b3446	3.774611555	2.86E-10	*yrhB*
gene-b1375	-3.749110108	4.61E-09	*ynaE*
gene-b1224	-3.710358571	7.60E-20	*narG*
gene-b1951	-3.699384008	8.00E-80	*rcsA*
gene-b0486	3.699379035	4.63E-115	*ybaT*
gene-b3514	3.682137017	1.75E-104	*mdtF*
gene-b4187	3.655394462	2.13E-79	*aidB*
gene-b1225	-3.639688253	6.20E-16	*narH*
gene-b4813	-3.629151108	5.98E-18	*narS*
gene-b1226	-3.616801189	4.96E-16	*narJ*

gene-b1037	3.593968213	1.47E-53	*csgG*
gene-b0335	3.583874487	1.38E-28	*prpE*
gene-b2110	-3.537111814	7.87E-06	*yehC*
gene-b3555	3.493291012	1.50E-88	*yiaG*
gene-b1042	3.491552891	2.07E-49	*csgA*
gene-b1906	3.486463925	1.23E-70	*yecH*
gene-b3239	3.446287098	1.64E-67	*yhcO*
gene-b4067	3.40101154	1.09E-47	*actP*
gene-b1227	-3.325990718	7.06E-16	*narI*
gene-b2241	3.322130441	2.22E-23	*glpA*
gene-b1038	3.307040249	5.78E-28	*csgF*
gene-b4620	3.290935982	1.10E-11	*yjbT*
gene-b1428	3.270286479	5.64E-78	*ydcK*
gene-b3451	3.270233436	1.78E-24	*ugpE*
gene-b4831	3.260495691	8.97E-57	*allZ*
gene-b4611	3.2235749	5.11E-37	*sibE*
gene-b0964	3.210703119	1.39E-45	*yccT*
gene-b4416	3.200730396	1.40E-54	*rybA*
gene-b4516	-3.184115403	0.002133893	*insA4*
gene-b1953	3.158811613	1.06E-101	*yodD*
gene-b2072	-3.144463767	3.43E-08	*pphC*
gene-b4705	3.127376405	1.48E-08	*mntS*
gene-b1223	-3.127130996	2.88E-10	*narK*
gene-b1494	3.122619824	7.46E-27	*pqqL*
gene-b3426	3.12113687	4.03E-82	*glpD*
gene-b0329	3.120136741	9.99E-62	*yahO*
gene-b2242	3.109225757	3.65E-18	*glpB*
gene-b3508	3.106745954	7.12E-48	*yhiD*
gene-b1810	3.081322573	2.64E-47	*yoaC*
gene-b3556	-3.073176127	1.08E-36	*cspA*
gene-b3646	-3.06106655	2.15E-23	*yicG*
gene-b0379	-3.057262413	1.12E-62	*yaiY*
gene-b3939	-3.041744619	1.01E-76	*metB*
gene-b2135	3.031134448	2.23E-103	*yohC*
gene-b1747	3.031050576	2.64E-72	*astA*
gene-b0435	3.014097112	6.28E-74	*bolA*
gene-b4035	2.999536078	9.34E-33	*malK*
gene-b3494	2.955502329	1.99E-61	*uspB*
gene-b2765	-2.948423691	4.22E-28	*queD*
gene-b4050	-2.900984102	1.62E-25	*pspG*
gene-b0698	2.88463896	1.38E-15	*kdpA*
gene-b3506	2.875307532	4.93E-76	*slp*
gene-b4209	-2.865498732	1.90E-14	*ytfE*
gene-b1952	2.862129405	6.96E-40	*dsrB*
gene-b1060	2.859441466	2.85E-61	*bssS*
gene-b3515	2.845004907	7.37E-45	*gadW*
gene-b1544	-2.841339265	1.04E-07	*ydfK*
gene-b3927	2.8267912	8.13E-84	*glpF*
gene-b4068	2.826583988	1.77E-27	*yjcH*
gene-b3828	-2.821911959	3.75E-29	*metR*
gene-b3257	-2.819103257	9.09E-05	*yhdT*
gene-b3049	2.817360301	1.98E-48	*glgS*
gene-b1746	2.815942843	9.94E-61	*astD*
gene-b1182	2.804981717	7.86E-35	*hlyE*
gene-b0972	2.804465295	1.38E-22	*hyaA*
gene-b1195	2.788263103	6.15E-80	*ymgE*
gene-b1511	2.787317771	2.19E-94	*lsrK*
gene-b1513	2.781905828	3.53E-49	*lsrA*
gene-b2243	2.776122533	2.80E-17	*glpC*
gene-b1039	2.767916059	4.37E-29	*csgE*
gene-b2660	2.738085958	8.01E-47	*lhgD*
gene-b1745	2.736875683	1.15E-49	*astB*
gene-b4189	2.71754924	1.27E-94	*bsmA*
gene-b2659	2.714437072	4.97E-35	*glaH*
gene-b0973	2.690725225	4.02E-18	*hyaB*
gene-b1000	2.689974381	1.78E-53	*cbpA*

gene-b1205	2.687825486	1.07E-60	*ychH*
gene-b1391	2.683793307	1.56E-13	*paaD*
gene-b2663	2.655573189	6.24E-41	*gabP*
gene-b4446	2.654371532	5.21E-24	*sibC*
gene-b1675	2.65335928	8.41E-29	*fumD*
gene-b4725	2.652768513	2.49E-12	*rseD*
gene-b1744	2.636717212	2.70E-65	*astE*
gene-b0979	2.618096705	2.56E-54	*appB*
gene-b2080	2.592699175	1.40E-54	*yegP*
gene-b1305	-2.590865898	3.61E-30	*pspB*
gene-b1304	-2.588462584	2.47E-52	*pspA*
gene-b1514	2.579884621	7.93E-58	*lsrC*
gene-b0999	2.543149162	2.58E-46	*cbpM*
gene-b1643	-2.541996852	5.62E-05	*ydhI*
gene-b1516	2.540580477	2.32E-48	*lsrB*
gene-b3103	2.514112053	6.23E-63	*yhaH*
gene-b1684	2.51262899	8.07E-42	*sufA*
gene-b2664	2.511730218	1.30E-46	*glaR*
gene-b1301	2.506155653	1.07E-32	*puuB*
gene-b0384	2.487773034	1.45E-53	*psiF*
gene-b1307	-2.487407664	5.72E-24	*pspD*
gene-b1748	2.478250958	5.68E-33	*astC*
gene-b1302	2.4731235	1.03E-31	*puuE*
gene-b0389	2.469385287	2.73E-43	*yaiA*
gene-b2662	2.464210859	2.83E-42	*gabT*
gene-b4418	2.454544743	2.27E-06	*sraB*
gene-b3941	-2.453005122	6.73E-22	*metF*
gene-b4567	2.452691095	1.89E-10	*yjjZ*
gene-b0978	2.448564541	6.43E-56	*appC*
gene-b4447	2.445664558	2.59E-42	*sibD*
gene-b3686	-2.441342145	2.65E-20	*ibpB*
gene-b1518	2.436134665	1.42E-45	*lsrG*
gene-b1957	2.429388282	2.93E-54	*yodC*
gene-b3482	-2.419197287	2.15E-42	*rhsB*
gene-b0873	-2.417216468	3.51E-05	*hcp*
gene-b0199	-2.409771029	1.48E-50	*metN*
gene-b2377	2.404649376	3.59E-36	*yfdY*
gene-b0124	2.4007565	2.12E-40	*gcd*
gene-b1515	2.396698927	1.17E-38	*lsrD*
gene-b1390	2.389966706	1.37E-24	*paaC*
gene-b0803	2.389800992	4.69E-25	*ybiI*
gene-b3362	2.388730807	8.30E-33	*yhfG*
gene-b2552	-2.366941916	8.55E-20	*hmp*
gene-b4345	-2.359179167	2.99E-07	*mcrC*
gene-b3518	2.357352346	3.44E-43	*ccp*
gene-b3552	-2.356541704	1.35E-58	*yiaD*
gene-b2112	2.354303075	4.80E-14	*yehE*
gene-b2390	-2.354043081	1.09E-45	*ypeC*
gene-b2724	-2.348040371	0.008408183	*hycB*
gene-b4048	2.339346073	0.000711856	*yjbM*
gene-b2466	-2.336757061	7.87E-40	*ypfG*
gene-b1490	2.33587329	1.59E-37	*dosC*
gene-b4094	2.334176879	1.69E-05	*phnN*
gene-b2661	2.333456392	5.59E-36	*gabD*
gene-b3926	2.310400882	1.64E-58	*glpK*
gene-b3481	-2.307817538	3.08E-18	*nikR*
gene-b1306	-2.306381437	1.79E-42	*pspC*
gene-b1188	2.30205442	1.05E-34	*ycgB*
gene-b4518	2.29936641	2.68E-37	*ymdF*
gene-b0325	2.290949964	1.20E-39	*yahK*
gene-b1440	2.283845865	3.05E-32	*ydcS*
gene-b0974	2.278345196	9.06E-14	*hyaC*
gene-b1480	2.277503645	1.32E-40	*sra*
gene-b3516	2.263345763	1.21E-36	*gadX*
gene-b4217	2.255655937	1.99E-51	*ytfK*
gene-b2809	2.253858389	8.58E-30	*ygdI*

gene-b2147	2.250497265	3.84E-24	*preA*
gene-b1795	2.249699016	1.01E-42	*yeaQ*
gene-b2333	-2.248489371	0.001058391	*yfcP*
gene-b2239	2.247750043	7.98E-39	*glpQ*
gene-b0260	-2.247164204	1.06E-34	*mmuP*
gene-b3361	2.240551704	9.38E-42	*fic*
gene-b1392	2.233867004	5.06E-22	*paaE*
gene-b0005	-2.228961043	1.30E-17	*yaaX*
gene-b1512	2.228917788	2.58E-56	*lsrR*
gene-b0304	2.221577224	8.37E-43	*rclA*
gene-b1683	2.220999902	5.04E-32	*sufB*
gene-b1441	2.220108655	2.97E-31	*ydcT*
gene-b1341	2.219755113	6.33E-48	*dgcM*
gene-b2146	2.217877853	7.66E-26	*preT*
gene-b2141	2.217116834	6.24E-17	*yohJ*
gene-b2142	2.216358192	3.59E-47	*yohK*
gene-b0530	2.216313829	2.18E-19	*sfmA*
gene-b1388	2.208909847	6.61E-15	*paaA*
gene-b2371	2.208627672	1.52E-10	*yfdE*
gene-b4060	2.206843187	2.92E-08	*yjcB*
gene-b0137	2.203465008	1.42E-10	*yadL*
gene-b1892	2.201351107	1.22E-05	*flhD*
gene-b4366	2.19502814	6.26E-15	*bglJ*
gene-b4411	2.194656066	1.12E-30	*ecnB*
gene-b2534	-2.188150274	6.43E-05	*yfhR*
gene-b0812	2.186051594	1.40E-46	*dps*
gene-b1020	2.175465673	2.40E-21	*phoH*
gene-b3055	-2.172354849	3.63E-56	*ygiM*
gene-b0564	2.169379958	5.13E-12	*appY*
gene-b0382	2.168123895	1.76E-24	*iraP*
gene-b2696	2.164331435	3.06E-53	*csrA*
gene-b4547	2.163285256	1.87E-31	*ypfN*
gene-b3519	2.162507138	3.03E-28	*treF*
gene-b4062	-2.158145183	5.65E-25	*soxS*
gene-b1517	2.151025327	9.69E-56	*lsrF*
gene-b0975	2.149185227	9.87E-19	*hyaD*
gene-b3606	-2.121007509	6.61E-15	*trmL*
gene-b2476	2.11763958	2.73E-35	*purC*
gene-b1478	2.114464332	1.10E-39	*adhP*
gene-b2386	2.111720576	5.64E-09	*fryC*
gene-b1784	2.107639786	2.27E-34	*yeaH*
gene-b3507	2.104550753	2.50E-07	*dctR*
gene-b4036	2.104354866	9.47E-26	*lamB*
gene-b1001	2.100053424	8.29E-13	*yccE*
gene-b4756	2.096307713	2.13E-06	*yqiD*
gene-b1495	2.094733272	2.73E-14	*yddB*
gene-b2557	2.094516094	3.07E-33	*purL*
gene-b0872	-2.094428204	1.57E-05	*hcr*
gene-b1783	2.092689065	1.78E-31	*yeaG*
gene-b1110	-2.089867729	6.41E-30	*ycfJ*
gene-b0624	2.084457595	9.14E-33	*crcB*
gene-b0434	-2.082497551	3.81E-61	*yajG*
gene-b3742	-2.076319883	1.40E-37	*mioC*
gene-b1668	2.061689393	7.67E-38	*ydhS*
gene-b2123	2.054849042	2.42E-05	*yehR*
gene-b0385	2.054437115	4.02E-13	*dgcC*
gene-b2677	-2.052684038	8.06E-26	*proV*
gene-b1040	2.050099267	2.47E-26	*csgD*
gene-b1678	2.049653342	5.23E-37	*ldtE*
gene-b2679	-2.047398831	1.57E-33	*proX*
gene-b1220	-2.041358703	3.33E-39	*ychO*
gene-b2240	2.041144591	7.09E-28	*glpT*
gene-b4215	2.039191877	5.30E-08	*ytfI*
gene-b1100	2.037004578	7.76E-43	*ycfH*
gene-b0301	2.035244434	5.90E-12	*rclC*
gene-b2387	2.034400202	0.000124147	*fryB*

gene-b4120	2.027937329	2.25E-28	*melB*
gene-b0108	-2.025672271	0.00103056	*ppdD*
gene-b1387	2.021195279	1.46E-24	*paaZ*
gene-b3711	-2.019703156	2.03E-18	*yidZ*
gene-b1508	2.019212846	9.80E-10	*hipB*
gene-b2753	-2.018597801	5.78E-20	*iap*
gene-b2924	2.008760181	7.06E-34	*mscS*
gene-b2665	2.00244124	1.14E-49	*kbp*
gene-b4149	1.999831375	6.15E-32	*blc*
gene-b2168	1.992867958	5.34E-24	*fruK*
gene-b2167	1.99039999	6.47E-43	*fruA*
gene-b2672	1.989615228	7.37E-51	*ygaM*
gene-b0265	-1.985419031	0.000197524	*insA2*
gene-b0275	-1.98541727	0.000197136	*insA3*
gene-b3524	1.98494785	1.76E-45	*yhjG*
gene-b3262	-1.974042645	0.00036359	*yhdJ*
gene-b4071	-1.972129453	2.47E-06	*nrfB*
gene-b1426	1.972098376	1.31E-26	*ydcH*
gene-b4325	1.970610468	0.000357548	*yjiC*
gene-b0403	-1.96131059	7.06E-34	*malZ*
gene-b3971	1.960955377	1.44E-11	*rrfB*
gene-b4010	1.960954049	1.45E-11	*rrfE*
gene-b3759	1.960952727	1.46E-11	*rrfC*
gene-b3274	1.960951387	1.47E-11	*rrfD*
gene-b2588	1.960949768	1.48E-11	*rrfG*
gene-b0205	1.960947888	1.50E-11	*rrfH*
gene-b4240	-1.960732863	4.72E-30	*treB*
gene-b0753	1.958202475	6.05E-32	*ybgS*
gene-b2492	-1.957498304	9.12E-07	*focB*
gene-b1300	1.950869024	5.15E-27	*puuC*
gene-b1867	-1.949823504	9.69E-46	*yecD*
gene-b0600	-1.949634514	1.93E-17	*ybdL*
gene-b1967	1.948282541	1.72E-31	*hchA*
gene-b4310	1.947041063	3.76E-27	*nanM*
gene-b0786	1.946870698	9.82E-49	*ybhL*
gene-b1296	1.945035184	6.51E-24	*puuP*
gene-b1794	-1.944317865	5.04E-46	*dgcP*
gene-b4568	1.932721767	9.40E-23	*ytjA*
gene-b2626	1.929604144	3.71E-05	*yfjJ*
gene-b3397	-1.928373834	3.85E-35	*nudE*
gene-b3522	1.926942679	1.95E-29	*yhjD*
gene-b2425	-1.922147228	4.62E-19	*cysP*
gene-b4188	1.917467475	2.39E-26	*yjfN*
gene-b1442	1.916122776	2.58E-24	*ydcU*
gene-b3365	-1.910922367	3.06E-07	*nirB*
gene-b1423	1.9099943	9.74E-24	*ydcJ*
gene-b4037	1.908372821	2.50E-19	*malM*
gene-b3480	-1.89875144	8.58E-13	*nikE*
gene-b1389	1.898105903	3.18E-07	*paaB*
gene-b1661	1.888581234	1.32E-45	*cfa*
gene-b1927	1.888501919	7.88E-43	*amyA*
gene-b1496	1.888044502	1.22E-08	*yddA*
gene-b4029	-1.886649005	1.89E-33	*yjbH*
gene-b1250	1.885897647	1.56E-34	*kch*
gene-b4013	-1.880052964	6.23E-18	*metA*
gene-b4554	1.878341163	5.68E-17	*yibT*
gene-b1051	1.877308743	6.49E-24	*msyB*
gene-b3336	1.873194197	5.18E-38	*bfr*
gene-b1197	1.872884599	3.78E-24	*treA*
gene-b4582	1.871568416	2.62E-17	*yoeA*
gene-b1159	-1.871224164	4.96E-10	*mcrA*
gene-b4459	1.870253375	3.21E-14	*ryjA*
gene-b2169	1.869537712	2.12E-06	*fruB*
gene-b1444	1.869404266	7.00E-40	*patD*
gene-b2266	1.867381833	1.04E-39	*elaB*
gene-b3074	1.866201931	1.44E-22	*ygjH*

gene-b0599	-1.864308753	4.89E-19	*hcxA*
gene-b1559	1.862679946	2.63E-16	*ydfT*
gene-b4437	1.854616198	8.39E-08	*sibB*
gene-b0138	1.854496761	1.19E-06	*yadM*
gene-b3923	1.852603971	8.14E-24	*uspD*
gene-b3588	1.842703313	3.21E-21	*aldB*
gene-b1156	1.841327481	2.12E-07	*tfaE*
gene-b4214	1.838238796	2.64E-38	*cysQ*
gene-b0546	1.836970999	1.02E-23	*ybcM*
gene-b3450	1.832827006	5.40E-23	*ugpC*
gene-b1682	1.828544198	5.94E-18	*sufC*
gene-b1849	1.828166586	3.22E-19	*purT*
gene-b1681	1.826870756	8.48E-18	*sufD*
gene-b2647	1.821695644	1.13E-30	*ypjA*
gene-b0456	1.820844512	2.90E-20	*ybaA*
gene-b0860	-1.817711168	1.20E-13	*artJ*
gene-b3940	-1.816962999	5.12E-38	*metL*
gene-b0871	1.812206557	2.60E-29	*poxB*
gene-b0985	-1.812074079	0.008447325	*gfcC*
gene-b1817	1.811485551	5.25E-48	*manX*
gene-b0787	1.810618494	9.76E-08	*ybhM*
gene-b1648	1.807275482	1.01E-21	*ydhL*
gene-b3696	-1.805446284	2.31E-10	*yidX*
gene-b3710	-1.804724	2.71E-08	*mdtL*
gene-b4072	-1.798559102	8.26E-07	*nrfC*
gene-b3546	-1.795395924	1.21E-32	*eptB*
gene-b1396	1.794656534	1.72E-08	*paaI*
gene-b1431	1.792974547	2.03E-41	*ydcL*
gene-b2467	-1.785205491	3.10E-36	*nudK*
gene-b2930	1.783698603	5.24E-11	*yggF*
gene-b3478	-1.78331437	4.80E-10	*nikC*
gene-b0841	-1.780743953	1.10E-16	*ybjG*
gene-b3674	-1.777652157	4.88E-20	*yidF*
gene-b1815	1.777015469	3.93E-32	*pdeD*
gene-b0602	1.775786936	0.000334624	*ybdN*
gene-b3448	1.774031368	8.17E-27	*yhhA*
gene-b1003	1.7732769	3.07E-20	*yccJ*
gene-b4376	1.770702716	6.76E-28	*osmY*
gene-b0098	-1.768406029	4.82E-46	*secA*
gene-b3073	1.768399675	7.00E-28	*patA*
gene-b1548	-1.768180052	0.000292193	*nohA*
gene-b3027	-1.766579328	0.000678616	*ygiZ*
gene-b4033	1.763816189	4.85E-17	*malF*
gene-b3654	1.763315466	1.60E-11	*xanP*
gene-b1724	1.763234989	1.66E-30	*ydiZ*
gene-b1868	-1.757672347	1.12E-20	*yecE*
gene-b1487	1.750017832	5.04E-14	*ddpA*
gene-b4231	1.74830026	1.14E-36	*yjfF*
gene-b1004	1.747608438	1.38E-36	*wrbA*
gene-b0762	1.747518479	2.59E-08	*acrZ*
gene-b1608	-1.745500435	1.70E-23	*rstA*
gene-b1443	1.741946971	1.16E-38	*ydcV*
gene-b4755	1.741286636	0.00012189	*yqhI*
gene-b1990	1.739714683	6.61E-27	*ldtA*
gene-b1691	1.737812148	5.35E-05	*ydiN*
gene-b4107	1.737766155	4.25E-35	*yjdN*
gene-b3452	1.737462073	7.72E-08	*ugpA*
gene-b1842	-1.736859848	2.55E-15	*holE*
gene-b4126	1.735470277	1.29E-19	*yjdI*
gene-b2935	-1.729729946	4.51E-43	*tktA*
gene-b0254	-1.727399244	1.26E-07	*perR*
gene-b0453	1.724944693	1.63E-25	*ybaY*
gene-b2187	-1.722688031	4.57E-09	*yejL*
gene-b0926	-1.720732347	5.01E-28	*mepK*
gene-b0623	1.720120297	8.55E-44	*cspE*
gene-b1739	1.719276415	1.27E-30	*osmE*

gene-b1101	1.708094425	1.76E-40	*ptsG*
gene-b3453	1.707022717	1.93E-15	*ugpB*
gene-b0906	1.703760212	2.53E-35	*ycaP*
gene-b2780	1.70362099	1.24E-42	*pyrG*
gene-b0316	-1.700809379	1.03E-16	*yahB*
gene-b0992	-1.692408629	4.54E-09	*yccM*
gene-b3488	1.68907946	5.26E-10	*yhiJ*
gene-b0461	1.688780822	6.62E-18	*tomB*
gene-b3102	1.686716189	5.68E-33	*yqjG*
gene-b0685	-1.686085808	2.51E-07	*ybfE*
gene-b4487	-1.684666049	5.14E-19	*yjdP*
gene-b3057	-1.682628365	5.74E-35	*bacA*
gene-b0347	1.682080496	1.27E-07	*mhpA*
gene-b3920	-1.67855893	7.88E-19	*yiiQ*
gene-b0527	-1.676193468	4.99E-10	*ybcI*
gene-b2302	1.675945876	1.49E-18	*yfcG*
gene-b0280	-1.674457704	1.44E-29	*yagN*
gene-b0348	1.673396754	0.000148489	*mhpB*
gene-b0697	1.672178564	3.66E-10	*kdpB*
gene-b4096	1.670971407	0.000566968	*phnL*
gene-b3459	-1.668258048	1.24E-17	*panZ*
gene-b2826	-1.667359982	2.69E-15	*ppdA*
gene-b1395	1.664190311	2.43E-24	*paaH*
gene-b1147	1.663704882	0.001896433	*ymfL*
gene-b0011	1.66313183	2.11E-14	*yaaW*
gene-b2137	1.66108937	9.81E-23	*yohF*
gene-b1923	1.659210187	6.13E-34	*fliC*
gene-b4245	-1.656180867	2.64E-13	*pyrB*
gene-b1623	-1.65600196	2.60E-29	*add*
gene-b1676	1.648727614	3.70E-32	*pykF*
gene-b3008	-1.648083193	1.95E-35	*metC*
gene-b0662	1.645458684	5.39E-29	*ubiF*
gene-b4109	1.645394975	1.42E-16	*rdcA*
gene-b1823	1.645281592	1.76E-25	*cspC*
gene-b2682	-1.644834183	8.54E-32	*ygaZ*
gene-b1819	1.643660235	1.80E-41	*manZ*
gene-b0966	1.643313345	2.11E-34	*hspQ*
gene-b0296	1.640601872	1.27E-05	*ykgM*
gene-b2408	1.640525996	3.08E-08	*yfeN*
gene-b4204	1.640108496	0.003575384	*yjfZ*
gene-b4069	1.639551953	1.88E-22	*acs*
gene-b0836	1.638116708	3.88E-29	*bssR*
gene-b1741	-1.638070959	9.19E-12	*cho*
gene-b3739	-1.637899961	9.00E-30	*atpI*
gene-b1680	1.63571133	6.97E-17	*sufS*
gene-b0241	1.634489432	3.14E-06	*phoE*
gene-b2678	-1.630956758	4.62E-20	*proW*
gene-b3291	1.630358936	3.94E-21	*mscL*
gene-b2144	-1.629862645	1.50E-10	*sanA*
gene-b1399	1.628371623	1.92E-32	*paaX*
gene-b1704	-1.627059686	1.43E-34	*aroH*
gene-b1264	-1.625924866	7.07E-21	*trpE*
gene-b4230	1.62580786	2.02E-23	*ytfT*
gene-b1486	1.625664813	1.58E-07	*ddpB*
gene-b2877	1.625596189	6.78E-10	*mocA*
gene-b1138	-1.623786431	1.92E-08	*ymfE*
gene-b0135	1.623028974	3.57E-05	*yadC*
gene-b1393	1.6225109	2.05E-14	*paaF*
gene-b4057	1.618309213	1.96E-40	*yjbR*
gene-b1489	1.618158571	3.41E-15	*dosP*
gene-b4457	1.614976093	1.00E-34	*csrC*
gene-b0287	1.613653462	2.68E-14	*yagU*
gene-b0507	1.613423968	2.55E-20	*gcl*
gene-b1846	-1.612597302	1.46E-15	*yebE*
gene-b2420	-1.610149224	3.07E-10	*yfeS*
gene-b2543	1.605522815	9.43E-25	*yphA*

gene-b1837	1.605376185	5.53E-10	*yebW*
gene-b1639	-1.60440102	1.64E-13	*mliC*
gene-b0545	1.603893316	1.21E-09	*ybcL*
gene-b0458	-1.601192341	7.17E-13	*ylaC*
gene-b2305	1.599128863	9.13E-07	*rpnB*
gene-b1295	-1.597603445	5.21E-08	*ymjA*
gene-b0637	-1.596397855	8.27E-13	*rsfS*
gene-b1172	-1.595077351	6.04E-15	*ymgG*
gene-b1012	1.593183535	0.01527956	*rutA*
gene-b3261	1.591145595	1.71E-15	*fis*
gene-b0161	-1.590811597	2.21E-23	*degP*
gene-b1014	1.588845604	0.009420114	*putA*
gene-b4465	1.587416606	7.66E-12	*yggP*
gene-b0033	1.578733236	1.98E-06	*carB*
gene-b1725	1.575441252	1.54E-37	*yniA*
gene-b3568	1.575410266	5.98E-27	*xylH*
gene-b2436	-1.573433187	7.14E-26	*hemF*
gene-b2121	1.572505869	0.005322271	*yehP*
gene-b3022	1.569778414	3.78E-15	*mqsR*
gene-b1484	1.567636026	3.23E-10	*ddpD*
gene-b3012	1.5671948	2.24E-20	*dkgA*
gene-b3415	1.566359143	5.17E-20	*gntT*
gene-b1450	1.565215967	2.91E-10	*mcbR*
gene-b3093	1.564001857	8.09E-21	*exuT*
gene-b1194	-1.55963171	0.010152801	*ycgR*
gene-b1818	1.559064345	9.08E-33	*manY*
gene-b0122	-1.55642152	3.03E-11	*yacC*
gene-b1583	-1.553850159	5.33E-21	*ynfB*
gene-b3615	-1.553549545	2.11E-09	*waaH*
gene-b0632	-1.552923073	2.35E-28	*dacA*
gene-b3638	-1.552733122	2.90E-17	*yicR*
gene-b2603	-1.552610761	1.35E-08	*yfiR*
gene-b1598	1.550372739	2.62E-32	*ydgD*
gene-b1937	-1.550032744	0.014192581	*fliE*
gene-b1394	1.545964106	1.21E-09	*paaG*
gene-b3829	-1.544981644	2.59E-10	*metE*
gene-b1377	-1.542106473	0.000227052	*ompN*
gene-b1726	1.540671035	7.10E-29	*yniB*
gene-b1891	1.537629961	3.50E-06	*flhC*
gene-b0358	1.537398797	1.88E-08	*yaiO*
gene-b0988	-1.535906715	0.032447258	*insB4*
gene-b1276	1.53533118	4.74E-24	*acnA*
gene-b2378	-1.531871477	3.98E-11	*lpxP*
gene-b4248	1.530968219	6.32E-15	*yjgH*
gene-b2454	1.529091399	0.002808209	*eutJ*
gene-b0139	1.528501518	0.005822308	*htrE*
gene-b3366	-1.525471727	0.000934381	*nirD*
gene-b4254	-1.525167292	4.29E-08	*argI*
gene-b3441	-1.52211196	1.03E-23	*yhhY*
gene-b3876	1.520876398	4.68E-05	*yihO*
gene-b0897	1.5198016	1.20E-24	*ycaC*
gene-b1271	-1.519205962	2.43E-24	*yciK*
gene-b0502	-1.516260424	1.65E-12	*ylbG*
gene-b1797	-1.511980627	0.004153521	*yeaR*
gene-b4241	-1.511591754	1.77E-24	*treR*
gene-b3484	-1.511516882	6.95E-12	*yhhI*
gene-b3161	-1.509120145	5.78E-20	*mtr*
gene-b0636	-1.507868949	2.60E-14	*rlmH*
gene-b1646	1.504871604	2.56E-19	*sodC*
gene-b4121	-1.503140193	3.80E-05	*yjdF*
gene-b3113	-1.496932023	2.88E-11	*tdcF*
gene-b2602	1.496345857	7.18E-10	*yfiL*
gene-b2740	1.493958142	2.72E-10	*ygbN*
gene-b2213	1.49212145	2.01E-18	*ada*
gene-b2253	1.491666431	1.86E-11	*arnB*
gene-b2905	-1.490872648	8.95E-18	*gcvT*

gene-b2509	-1.486933701	3.90E-30	*xseA*
gene-b2085	1.486553355	0.002792707	*yegR*
gene-b0849	1.483238467	1.45E-06	*grxA*
gene-b1385	1.477079081	1.80E-30	*feaB*
gene-b1762	-1.476392134	2.66E-05	*ynjI*
gene-b0880	1.475606493	9.51E-28	*cspD*
gene-b2212	1.474639224	4.84E-07	*alkB*
gene-b0821	1.47382574	9.55E-18	*ybiU*
gene-b1915	1.472050587	1.40E-08	*yecF*
gene-b3088	-1.470614718	4.24E-15	*alx*
gene-b1216	-1.46932182	4.57E-24	*chaA*
gene-b0544	1.467776719	0.001077693	*ybcK*
gene-b2631	1.466035719	5.51E-16	*rnlB*
gene-b1614	1.464841076	9.84E-25	*ydgA*
gene-b2291	-1.46470788	5.19E-21	*yfbR*
gene-b2419	-1.463209129	1.20E-07	*yfeK*
gene-b4326	1.459878656	0.004542464	*iraD*
gene-b4309	1.459878415	6.89E-06	*nanS*
gene-b0508	1.459014903	0.004428474	*hyi*
gene-b4034	1.458750394	2.16E-18	*malE*
gene-b2413	-1.456255142	1.24E-24	*cysZ*
gene-b0198	-1.455793346	1.35E-26	*metI*
gene-b0927	-1.453238875	3.47E-23	*gloC*
gene-b0855	1.45149272	1.47E-09	*potG*
gene-b3360	1.449776779	2.19E-10	*pabA*
gene-b1664	1.449663153	1.41E-27	*ydhQ*
gene-b3128	1.44874305	2.15E-28	*garD*
gene-b4232	-1.446330031	1.87E-23	*fbp*
gene-b1545	-1.44508531	0.005992108	*pinQ*
gene-b2275	1.441553618	0.005480507	*yfbP*
gene-b2015	1.439998711	7.77E-23	*yeeY*
gene-b3238	1.439224677	4.21E-05	*yhcN*
gene-b3348	1.439140532	3.02E-13	*slyX*
gene-b1886	-1.435825288	0.008082693	*tar*
gene-b4406	1.43439867	2.11E-20	*yaeP*
gene-b4022	-1.433481095	3.97E-19	*rluF*
gene-b1803	1.43259108	5.08E-11	*yeaX*
gene-b1285	1.427902563	7.05E-25	*pdeR*
gene-b4379	-1.425677933	6.60E-07	*yjjW*
gene-b1297	1.424772978	2.25E-10	*puuA*
gene-b2475	1.423084861	6.82E-22	*ypfJ*
gene-b2972	-1.422635259	0.000149708	*pppA*
gene-b2465	1.418521719	1.92E-21	*tktB*
gene-b4124	-1.416971756	9.81E-15	*dcuR*
gene-b3922	1.415165187	9.84E-19	*yiiS*
gene-b1482	1.414991083	5.62E-26	*osmC*
gene-b3381	1.41137865	0.00178883	*yhfX*
gene-b1943	-1.410805177	0.005688448	*fliK*
gene-b2666	1.40780139	7.75E-07	*yqaE*
gene-b0064	-1.403886296	6.37E-29	*araC*
gene-b3917	-1.403026639	9.13E-08	*sbp*
gene-b1043	1.401967039	0.00559036	*csgC*
gene-b2153	-1.400908062	2.18E-29	*folE*
gene-b0953	1.399923573	3.05E-21	*rmf*
gene-b3586	1.399573431	0.000321374	*yiaV*
gene-b2183	-1.399022117	3.86E-16	*rsuA*
gene-b1398	1.397018733	7.40E-20	*paaK*
gene-b4167	1.397016684	9.57E-21	*nnr*
gene-b0464	-1.396340611	1.07E-07	*acrR*
gene-b3089	1.39587678	2.34E-22	*sstT*
gene-b2687	1.39519354	5.67E-16	*luxS*
gene-b4127	1.393711554	7.94E-12	*yjdJ*
gene-b1751	-1.392559312	1.13E-10	*ydjY*
gene-b3483	-1.39239583	0.016042746	*yhhH*
gene-b4005	1.391967452	2.92E-17	*purD*
gene-b0274	-1.391781365	1.12E-05	*insB3*

gene-b0264	-1.391780357	1.12E-05	*insB2*
gene-b4565	1.390651041	0.000231146	*sgcB*
gene-b1241	1.390541281	4.44E-28	*adhE*
gene-b0010	1.388504723	3.81E-17	*satP*
gene-b2009	1.388430567	2.03E-22	*sbmC*
gene-b3811	-1.388036852	2.43E-23	*xerC*
gene-b0412	-1.385757609	7.34E-24	*yajI*
gene-b2369	-1.384601912	3.27E-14	*evgA*
gene-b1793	-1.384448506	1.82E-09	*yoaF*
gene-b2496	-1.383167367	2.24E-13	*hda*
gene-b2869	1.382092634	1.01E-18	*ygeV*
gene-b2237	1.381843736	4.27E-21	*inaA*
gene-b1692	1.380345499	0.00016807	*ydiB*
gene-b3626	1.376928397	7.10E-14	*waaJ*
gene-b0320	1.376501217	3.96E-07	*yahF*
gene-b0643	1.375716389	1.00E-19	*ybeL*
gene-b0494	-1.374896401	1.59E-17	*tesA*
gene-b0158	-1.373685221	5.79E-09	*btuF*
gene-b0211	-1.372400757	1.10E-23	*mltD*
gene-b2417	1.371191799	1.84E-24	*crr*
gene-b0007	1.371052037	1.91E-16	*yaaJ*
gene-b1677	1.370649517	1.35E-19	*lpp*
gene-b2774	1.369059033	9.36E-06	*ygcW*
gene-b2831	-1.368179694	1.66E-14	*mutH*
gene-b0147	-1.365508849	1.08E-07	*thpR*
gene-b3629	1.36498434	5.87E-10	*waaS*
gene-b1824	1.36490068	5.56E-10	*yobF*
gene-b3267	-1.364438269	7.13E-06	*yhdV*
gene-b2683	-1.362031302	2.44E-05	*ygaH*
gene-b3502	1.361505544	3.72E-10	*arsB*
gene-b1897	1.361370831	9.14E-22	*otsB*
gene-b3859	-1.359840609	8.58E-13	*srkA*
gene-b0788	1.359333623	7.66E-15	*ybhN*
gene-b1737	1.357175393	8.44E-11	*chbC*
gene-b3024	1.355841342	8.31E-23	*ygiW*
gene-b0790	1.3557823	2.61E-14	*ybhP*
gene-b3572	-1.355291894	2.20E-22	*avtA*
gene-b3870	-1.352184947	1.94E-22	*glnA*
gene-b1820	1.349975526	5.81E-05	*yobD*
gene-b2127	1.348927684	1.73E-19	*mlrA*
gene-b0866	1.348531058	1.14E-24	*ybjQ*
gene-b0272	-1.3479298	1.49E-12	*xynR*
gene-b3597	1.346230155	3.82E-19	*yibH*
gene-b1376	1.345896194	7.72E-17	*uspF*
gene-b3687	-1.343945063	8.44E-06	*ibpA*
gene-b0696	1.343760097	5.23E-09	*kdpC*
gene-b1320	-1.342694238	1.27E-10	*ycjW*
gene-b4052	-1.342628039	1.79E-22	*dnaB*
gene-b0899	1.342046779	1.18E-10	*ycaM*
gene-b3154	-1.34189014	1.59E-07	*yhbP*
gene-b0622	1.33881643	9.62E-14	*pagP*
gene-b4208	1.338533555	3.54E-14	*cycA*
gene-b1615	1.336036911	1.26E-05	*uidC*
gene-b2330	-1.335352349	4.10E-24	*prmB*
gene-b4269	1.333129377	3.07E-17	*ahr*
gene-b4485	1.332935001	2.10E-17	*ytfR*
gene-b0317	1.330263462	0.004086617	*yahC*
gene-b2107	-1.329733689	4.88E-17	*rcnB*
gene-b4051	1.329611577	1.93E-24	*qorA*
gene-b3800	-1.329495247	1.76E-06	*aslB*
gene-b0791	1.328300225	1.03E-20	*ybhQ*
gene-b1237	1.327975662	1.25E-22	*hns*
gene-b0504	-1.327221895	0.005912615	*allS*
gene-b4001	-1.326056974	1.97E-14	*yjaH*
gene-b0721	-1.324196491	6.96E-24	*sdhC*
gene-b3472	-1.322863928	4.49E-24	*dcrB*

gene-b2975	1.322645972	3.48E-08	*glcA*
gene-b3097	1.322604299	1.88E-19	*yqjC*
gene-b1973	1.322211038	5.99E-12	*zinT*
gene-b4336	1.321665935	7.38E-17	*yjiN*
gene-b2798	-1.32103399	2.15E-15	*ygdG*
gene-b3664	1.318425207	4.68E-05	*adeQ*
gene-b1270	-1.318406869	1.34E-20	*btuR*
gene-b2779	1.317985804	8.69E-23	*eno*
gene-b4002	-1.317762536	0.022520132	*zraP*
gene-b0136	1.317001977	0.005005853	*yadK*
gene-b1335	-1.315495376	1.08E-16	*ogt*
gene-b4805	1.315055786	6.76E-09	*raiZ*
gene-b1971	1.314836394	3.64E-22	*msrP*
gene-b0925	1.309514177	1.14E-18	*ldtD*
gene-b2312	1.308611949	2.72E-10	*purF*
gene-b0493	-1.308400891	1.42E-20	*ybbO*
gene-b2392	1.306636022	1.90E-20	*mntH*
gene-b0707	1.30649343	9.76E-09	*ybgA*
gene-b3554	1.306344036	3.02E-23	*yiaF*
gene-b0938	1.30435487	3.67E-05	*elfA*
gene-b4304	1.302744431	4.46E-10	*sgcC*
gene-b1164	1.302335755	0.002675771	*ycgZ*
gene-b2754	1.298242769	0.025861896	*cas2*
gene-b1584	-1.29505014	1.31E-17	*speG*
gene-b2749	-1.294083869	1.55E-08	*ygbE*
gene-b2987	1.292767232	0.000623015	*pitB*
gene-b0250	1.292066634	5.02E-17	*ykfB*
gene-b0751	1.290307051	5.41E-18	*pnuC*
gene-b3474	1.287200774	3.67E-18	*yhhT*
gene-b3809	-1.284659501	1.58E-13	*dapF*
gene-b3083	-1.282510107	0.0127461	*higB*
gene-b1920	1.277966163	2.94E-21	*tcyJ*
gene-b0146	1.277789705	1.77E-15	*sfsA*
gene-b2506	1.277692413	9.67E-07	*yfgI*
gene-b4311	1.277300978	0.000764965	*nanC*
gene-b2761	1.276420079	4.48E-12	*cas3*
gene-b4314	1.276315299	5.18E-11	*fimA*
gene-b1619	1.274524353	4.14E-10	*hdhA*
gene-b0976	1.274470373	0.000358425	*hyaE*
gene-b3153	1.272356043	7.40E-11	*yhbO*
gene-b1847	1.272175621	4.53E-18	*yebF*
gene-b0854	1.271753272	1.10E-11	*potF*
gene-b2533	-1.270693544	3.98E-17	*suhB*
gene-b2889	1.269188247	3.06E-14	*idi*
gene-b3136	1.268646441	0.014855375	*agaS*
gene-b1345	1.26733684	4.25E-06	*intR*
gene-b0343	1.266188792	0.01507059	*lacY*
gene-b1750	-1.265773674	3.21E-06	*ydjX*
gene-b1841	-1.264703282	7.34E-17	*yobA*
gene-b2073	-1.264416968	7.79E-07	*yegL*
gene-b1476	-1.263173187	8.77E-05	*fdnI*
gene-b3271	1.261817072	1.83E-05	*yhdZ*
gene-b0099	-1.261150504	2.17E-05	*mutT*
gene-b1196	1.259566216	0.008225523	*ycgY*
gene-b4512	1.259245233	7.15E-11	*ybdD*
gene-b2350	-1.258201739	2.98E-07	*yfdG*
gene-b2731	1.256796193	3.92E-16	*fhlA*
gene-b1536	1.256576047	3.27E-15	*ydeI*
gene-b3633	-1.254828529	2.51E-20	*waaA*
gene-b3449	1.253659358	1.38E-14	*ugpQ*
gene-b1235	1.25237468	8.02E-20	*rssB*
gene-b0195	-1.25222756	8.70E-09	*trmO*
gene-b3527	-1.250207387	6.43E-21	*yhjJ*
gene-b1210	-1.248812757	3.97E-20	*hemA*
gene-b4119	1.248714727	1.25E-10	*melA*
gene-b2207	-1.248100256	0.010082885	*napD*

gene-b0717	1.246406589	0.043574741	*ybgP*
gene-b1702	-1.246014193	1.27E-23	*ppsA*
gene-b2949	-1.24315806	7.03E-10	*yqgF*
gene-b4227	1.243033426	2.26E-24	*ytfQ*
gene-b2535	1.242919946	5.74E-17	*csiE*
gene-b1543	1.242241728	0.001001067	*ydfJ*
gene-b3627	1.242007361	8.40E-11	*waaO*
gene-b1187	-1.241562452	2.28E-20	*fadR*
gene-b1712	1.24143308	2.30E-20	*ihfA*
gene-b3821	-1.239877855	3.06E-17	*pldA*
gene-b1529	1.238213706	2.88E-19	*marC*
gene-b2435	-1.237611934	2.05E-19	*amiA*
gene-b2736	1.236184753	1.51E-07	*ygbJ*
gene-b3479	-1.23576936	2.61E-06	*nikD*
gene-b0876	1.234871042	1.89E-18	*ybjD*
gene-b2254	1.234733763	0.000488299	*arnC*
gene-b3496	1.232505083	4.31E-21	*dtpB*
gene-b3548	1.231712482	1.34E-17	*yhjY*
gene-b2451	1.23161845	0.000871322	*eutA*
gene-b0767	1.231245248	6.07E-18	*pgl*
gene-b0581	1.230245733	1.10E-16	*ybdK*
gene-b4390	1.23003456	1.22E-18	*nadR*
gene-b2459	1.229476453	0.000775376	*eutT*
gene-b0759	1.229464201	4.99E-18	*galE*
gene-b1844	-1.228933289	3.27E-13	*exoX*
gene-b3418	1.228776944	4.19E-22	*malT*
gene-b0186	1.227560906	2.92E-14	*ldcC*
gene-b2752	-1.227128883	6.20E-06	*cysD*
gene-b3394	-1.226892535	0.001976339	*hofN*
gene-b0678	1.226645949	4.05E-19	*nagB*
gene-b4346	-1.22600747	4.79E-09	*mcrB*
gene-b2976	1.225928614	8.15E-25	*glcB*
gene-b3041	1.22344315	1.67E-15	*ribB*
gene-b1523	-1.222774984	0.009163241	*yneG*
gene-b2446	1.22271497	0.000969715	*yffO*
gene-b3954	-1.222033877	2.37E-09	*yijO*
gene-b3812	-1.221161507	5.26E-13	*yigB*
gene-b1652	-1.22030765	7.37E-07	*rnt*
gene-b2739	1.219810576	9.60E-05	*ygbM*
gene-b0965	1.218172546	5.68E-20	*yccU*
gene-b1896	1.217270588	3.76E-16	*otsA*
gene-b1956	1.216722901	1.71E-19	*dgcQ*
gene-b0475	1.216678309	1.83E-18	*hemH*
gene-b3290	1.216632187	3.93E-21	*trkA*
gene-b1759	-1.216158272	6.81E-05	*nudG*
gene-b2134	-1.213636595	5.48E-15	*pbpG*
gene-b3021	1.212790406	9.19E-11	*mqsA*
gene-b1607	-1.212639545	1.15E-06	*ydgC*
gene-b3101	1.210842141	0.000130382	*yqjF*
gene-b2077	1.210489092	1.35E-05	*mdtD*
gene-b3748	1.210445367	5.63E-14	*rbsD*
gene-b0511	1.209918419	0.017820227	*ybbW*
gene-b1181	1.209302236	7.02E-13	*ycgN*
gene-b3937	-1.208892555	2.22E-07	*yiiX*
gene-b4049	-1.206059115	6.55E-19	*dusA*
gene-b4699	-1.2048244	0.000135815	*fnrS*
gene-b4012	1.204635703	3.24E-12	*yjaB*
gene-b0162	-1.203466035	8.43E-12	*cdaR*
gene-b1921	1.202690515	0.002028131	*fliZ*
gene-b4263	1.20126703	1.86E-17	*yjgR*
gene-b0164	-1.200880542	0.001480103	*yaeI*
gene-b0977	1.200194383	1.28E-05	*hyaF*
gene-b1535	-1.200117345	5.04E-11	*dgcZ*
gene-b1840	-1.199388627	3.09E-15	*yebZ*
gene-b4256	-1.197957606	1.35E-11	*yjgM*
gene-b4056	1.197943017	1.13E-19	*yjbQ*

gene-b3077	1.197454394	0.020902321	*ebgC*
gene-b1821	-1.197403641	0.000765887	*mntP*
gene-b1601	-1.196980412	9.46E-15	*tqsA*
gene-b1730	1.195342971	0.023009328	*ydjO*
gene-b2578	1.192977871	4.62E-11	*eamB*
gene-b1963	1.192728435	0.003477687	*drpB*
gene-b0119	1.192060167	3.16E-15	*yacL*
gene-b3520	1.191708781	8.76E-10	*yhjB*
gene-b3280	1.190627803	8.05E-11	*yrdB*
gene-b3728	-1.188861592	1.24E-09	*pstS*
gene-b0789	1.1849609	2.57E-10	*clsB*
gene-b2019	-1.184897753	7.65E-19	*hisG*
gene-b3946	-1.184763461	7.26E-10	*fsaB*
gene-b4609	1.184550536	7.70E-07	*ryfD*
gene-b3476	-1.182093787	4.09E-08	*nikA*
gene-b0571	-1.18195247	7.61E-12	*cusR*
gene-b0968	1.181482976	2.60E-07	*yccX*
gene-b1537	1.18107649	1.75E-11	*ydeJ*
gene-b1801	1.180485485	0.005204382	*yeaV*
gene-b0781	-1.179276678	4.48E-16	*moaA*
gene-b3287	1.177433267	1.74E-19	*def*
gene-b3454	1.176527884	0.000192282	*livF*
gene-b1380	1.175406443	1.86E-19	*ldhA*
gene-b3808	1.174263716	0.003390698	*yzcX*
gene-b1469	1.173409878	5.05E-12	*narU*
gene-b3677	-1.173370487	3.64E-09	*yidI*
gene-b0912	1.172696806	7.70E-17	*ihfB*
gene-b4382	1.168937489	2.02E-14	*deoA*
gene-b1890	-1.168237433	0.0574543	*motA*
gene-b0856	1.167838278	0.003670347	*potH*
gene-b1047	-1.165965457	1.47E-05	*opgC*
gene-b4255	1.165876319	8.18E-17	*rraB*
gene-b1959	-1.165684758	1.87E-09	*yedA*
gene-b2156	1.164524108	3.14E-21	*lysP*
gene-b3628	1.163928563	4.73E-10	*waaB*
gene-b2074	1.163067561	1.49E-10	*mdtA*
gene-b3810	-1.16289129	1.08E-16	*yigA*
gene-b1816	-1.162534736	3.73E-20	*yoaE*
gene-b1094	1.161277159	5.54E-18	*acpP*
gene-b0157	-1.160599722	4.82E-07	*yadS*
gene-b4383	1.159116654	1.01E-15	*deoB*
gene-b2310	1.158653567	2.02E-07	*argT*
gene-b4331	-1.157154866	5.90E-14	*kptA*
gene-b1917	1.154914117	1.54E-19	*tcyN*
gene-b0142	-1.153288897	0.000698028	*folK*
gene-b3966	-1.152863405	6.20E-21	*btuB*
gene-b3099	1.151448525	1.59E-18	*yqjE*
gene-b4135	1.151445899	3.09E-18	*yjdC*
gene-b3085	-1.151390195	8.90E-05	*ygjP*
gene-b1358	1.150642801	0.008447955	*ydaT*
gene-b2321	-1.149735521	5.32E-10	*flk*
gene-b2571	-1.149104164	3.67E-16	*rseB*
gene-b0394	1.148352697	9.69E-18	*mak*
gene-b4070	-1.145748068	8.46E-05	*nrfA*
gene-b2737	1.145741433	1.41E-06	*ygbK*
gene-b1045	1.14574067	7.66E-18	*ymdB*
gene-b4133	1.144773719	2.10E-08	*cadC*
gene-b4064	1.143601137	2.04E-08	*ghxP*
gene-b3918	-1.143078148	0.000135685	*cdh*
gene-b0993	-1.143017687	1.17E-09	*torS*
gene-b2768	-1.142186015	0.000215352	*ygcP*
gene-b0484	1.14166665	6.47E-15	*copA*
gene-b0405	-1.140622318	3.83E-13	*queA*
gene-b0425	1.140434403	2.08E-10	*panE*
gene-b2792	-1.140269968	3.93E-05	*yqcC*
gene-b1449	1.139888735	8.54E-19	*curA*

gene-b4358	1.139544292	0.001554599	*lgoD*
gene-b1931	1.138928833	3.79E-08	*yedK*
gene-b3175	-1.138317821	5.78E-12	*secG*
gene-b3680	-1.138154426	2.58E-06	*yidL*
gene-b2075	1.137736504	1.09E-12	*mdtB*
gene-b1987	-1.137576084	5.07E-06	*cbl*
gene-b1960	-1.13681161	7.09E-06	*vsr*
gene-b0097	-1.136610317	2.87E-11	*secM*
gene-b1524	-1.13576021	1.39E-09	*glsB*
gene-b0867	1.135597025	7.10E-13	*amiD*
gene-b0273	-1.134859987	6.39E-11	*argF*
gene-b4194	1.132926883	0.040119293	*ulaB*
gene-b0920	-1.132662212	1.67E-10	*elyC*
gene-b3130	1.131161012	6.26E-11	*yhaV*
gene-b2944	-1.128873539	0.001700578	*yggI*
gene-b2250	-1.127727435	4.15E-06	*yfaZ*
gene-b2209	-1.126879283	4.65E-18	*eco*
gene-b2106	-1.124916394	0.000246751	*rcnA*
gene-b2458	1.123285108	8.92E-06	*eutD*
gene-b4278	-1.122756077	1.90E-07	*insG*
gene-b0857	1.121011879	2.01E-06	*potI*
gene-b1112	1.120406491	2.38E-06	*bhsA*
gene-b3098	1.118526337	2.75E-19	*yqjD*
gene-b2817	-1.118422235	2.89E-13	*amiC*
gene-b4234	1.118289771	5.31E-18	*yjgA*
gene-b0457	1.118017846	2.05E-14	*pdeB*
gene-b1540	-1.117940892	2.14E-11	*rspR*
gene-b3746	1.117253526	1.07E-19	*ravA*
gene-b3755	1.115937196	2.58E-16	*yieP*
gene-b4031	1.115612057	2.97E-08	*xylE*
gene-b0630	-1.114846031	2.45E-07	*lipB*
gene-b4110	1.114072026	0.009745445	*rdcB*
gene-b2177	1.114039296	8.19E-15	*yejA*
gene-b1400	1.113846148	4.12E-16	*paaY*
gene-b0695	1.11352083	8.37E-14	*kdpD*
gene-b3613	-1.113238621	1.04E-16	*envC*
gene-b0522	1.11308429	2.58E-08	*purK*
gene-b3421	1.113027817	2.29E-09	*rtcB*
gene-b1640	-1.112901491	5.45E-19	*anmK*
gene-b4118	1.111368977	5.32E-15	*melR*
gene-b3014	1.11134053	0.095702888	*yqhH*
gene-b4305	1.110024104	4.99E-09	*sgcX*
gene-b3845	1.109400473	4.70E-13	*fadA*
gene-b2480	-1.108117672	2.19E-16	*bcp*
gene-b0279	-1.107487456	0.054850808	*yagM*
gene-b3140	1.106325798	0.084460436	*agaD*
gene-b1018	1.103599296	5.03E-10	*efeO*
gene-b3947	-1.102316436	3.97E-10	*ptsA*
gene-b4577	1.102063563	2.72E-10	*sgrS*
gene-b1281	-1.101092652	4.20E-11	*pyrF*
gene-b0189	1.100029255	1.13E-07	*rof*
gene-b2495	-1.098799603	8.45E-15	*yfgD*
gene-b1360	1.097807995	0.000918852	*ydaV*
gene-b1787	-1.097307458	8.18E-09	*yeaK*
gene-b0295	1.096961806	0.028390327	*ykgL*
gene-b2732	-1.09681583	5.15E-05	*ygbA*
gene-b2570	-1.095575364	5.40E-11	*rseC*
gene-b0842	1.095182263	1.19E-13	*mdfA*
gene-b3960	1.094748674	2.06E-17	*argH*
gene-b0780	-1.094080112	3.34E-12	*ybhK*
gene-b1212	-1.093536046	3.71E-09	*prmC*
gene-b0422	-1.093367069	8.89E-05	*xseB*
gene-b1177	1.092761974	2.82E-08	*ycgJ*
gene-b1059	-1.091510067	8.16E-14	*solA*
gene-b1610	1.091442744	3.07E-08	*tus*
gene-b2991	-1.09120122	0.00593346	*hybF*

gene-b3477	-1.09056825	3.47E-05	*nikB*
gene-b0398	-1.090128033	9.18E-11	*sbcD*
gene-b0946	-1.090098505	2.06E-11	*zapC*
gene-b0390	1.089979475	2.42E-09	*aroM*
gene-b3690	-1.088640679	0.008621172	*cbrA*
gene-b4441	-1.088523676	3.77E-14	*glmY*
gene-b0543	1.086757094	0.015794288	*emrE*
gene-b1192	-1.086741794	4.42E-09	*ldcA*
gene-b3697	-1.086289799	1.42E-08	*yidA*
gene-b3598	1.085542081	6.54E-05	*yibI*
gene-b2532	-1.084922543	1.80E-14	*trmJ*
gene-b4279	1.082741424	5.23E-07	*nanX*
gene-b3958	-1.08235088	1.99E-07	*argC*
gene-b0806	1.08077086	1.10E-10	*mcbA*
gene-b2619	-1.080505062	9.62E-15	*ratA*
gene-b0557	-1.079548557	0.000166419	*borD*
gene-b3816	-1.078809588	9.60E-12	*corA*
gene-b3100	1.076984269	3.65E-15	*yqjK*
gene-b2597	1.076095442	1.56E-14	*raiA*
gene-b1082	1.076005499	1.48E-07	*flgK*
gene-b0225	-1.075977036	0.000620693	*yafQ*
gene-b1633	-1.075148343	7.13E-05	*nth*
gene-b3594	1.073093618	2.80E-09	*yibA*
gene-b4357	1.072259877	1.97E-12	*lgoR*
gene-b0875	1.071707571	3.64E-10	*aqpZ*
gene-b2620	-1.071607697	3.92E-09	*smpB*
gene-b4244	-1.071461717	1.17E-05	*pyrI*
gene-b3823	-1.071224751	5.44E-11	*rhtC*
gene-b2452	1.07102404	0.000369117	*eutH*
gene-b3127	-1.069283027	0.000503949	*garP*
gene-b0660	1.068341802	4.51E-12	*ybeZ*
gene-b0754	-1.067743272	8.94E-18	*aroG*
gene-b0874	-1.063766186	1.62E-08	*lysO*
gene-b2464	1.063162303	5.77E-14	*talA*
gene-b4829	1.063128984	0.000127296	*uhpU*
gene-b4596	-1.062982741	0.000793672	*yciZ*
gene-b2813	-1.062859252	2.80E-15	*mltA*
gene-b3630	1.062034993	5.93E-08	*waaP*
gene-b4463	1.061848102	1.86E-06	*ygcU*
gene-b0921	-1.059647069	2.54E-10	*cmoM*
gene-b3363	-1.057666789	1.39E-13	*ppiA*
gene-b1368	-1.05760843	0.017314205	*ynaA*
gene-b3963	-1.056091694	6.80E-15	*fabR*
gene-b1582	-1.055975995	0.000564605	*ynfA*
gene-b0285	1.0555618	1.35E-06	*paoB*
gene-b0193	-1.054734538	0.041754783	*yaeF*
gene-b0509	1.054016159	0.000528414	*glxR*
gene-b4397	-1.053451152	1.40E-10	*creA*
gene-b4157	-1.05325434	0.025249119	*yjeN*
gene-b1209	-1.052199747	1.79E-14	*lolB*
gene-b0682	1.051662108	0.000600975	*chiQ*
gene-b2076	1.051245549	4.83E-11	*mdtC*
gene-b1961	-1.050806544	3.22E-14	*dcm*
gene-b4394	-1.050204345	5.56E-10	*yjjX*
gene-b2788	-1.050013282	6.10E-07	*gudX*
gene-b1945	-1.049796409	0.045731559	*fliM*
gene-b3344	-1.049630812	2.20E-06	*tusC*
gene-b4114	1.049065683	0.000113471	*eptA*
gene-b3281	1.048829477	1.11E-15	*aroE*
gene-b2874	1.048532702	5.63E-09	*yqeA*
gene-b0156	1.048427996	8.90E-11	*erpA*
gene-b2913	-1.04826289	3.10E-13	*serA*
gene-b2582	1.0472508	7.26E-07	*trxC*
gene-b0008	-1.046802492	1.12E-19	*talB*
gene-b1679	1.046077093	1.44E-09	*sufE*
gene-b1113	-1.045617162	3.89E-13	*ldtC*

gene-b3643	-1.044007572	9.19E-15	*rph*
gene-b2136	1.041319065	2.99E-13	*yohD*
gene-b1586	1.040730414	2.58E-15	*ynfD*
gene-b1083	1.040187777	4.75E-10	*flgL*
gene-b1859	-1.03863034	1.14E-09	*znuB*
gene-b1284	-1.037730006	1.74E-09	*yciT*
gene-b3447	1.037666052	1.39E-13	*ggt*
gene-b1179	-1.037598449	2.61E-09	*ycgL*
gene-b3207	1.037404636	8.35E-16	*yrbL*
gene-b2225	1.036675349	0.002277382	*yfaP*
gene-b2257	1.0366215	3.49E-06	*arnT*
gene-b2176	-1.036484372	1.06E-09	*pdeN*
gene-b1397	1.035155764	7.42E-14	*paaJ*
gene-b1234	1.034312783	1.64E-11	*rssA*
gene-b1541	1.034171075	0.004234724	*ydfZ*
gene-b1627	-1.033519827	4.53E-06	*rsxA*
gene-b3536	1.033497987	8.23E-13	*bcsE*
gene-b4436	-1.031751639	6.68E-06	*sibA*
gene-b1418	-1.031477867	1.39E-14	*cybB*
gene-b2954	-1.031328249	2.61E-10	*rdgB*
gene-b2575	-1.030619578	1.61E-09	*yfiC*
gene-b4555	1.030270998	0.008986994	*yicS*
gene-b0474	-1.029281679	8.11E-16	*adk*
gene-b3159	-1.029244451	0.000430509	*ubiV*
gene-b1406	1.028958138	4.86E-15	*pdxI*
gene-b0163	1.027495376	2.61E-13	*yaeH*
gene-b2037	1.026023513	1.73E-07	*rfbX*
gene-b3924	-1.024834618	9.92E-14	*fpr*
gene-b0798	-1.024588819	7.20E-05	*ybiA*
gene-b4401	1.024428631	1.13E-13	*arcA*
gene-b0795	-1.024335608	1.30E-10	*ybhG*
gene-b2938	1.023573368	4.94E-14	*speA*
gene-b3663	-1.023482557	0.000341577	*yicN*
gene-b2255	1.021848443	6.42E-07	*arnA*
gene-b2109	-1.021668592	0.002069191	*yehB*
gene-b0227	1.02056926	1.52E-06	*yafL*
gene-b3599	1.019594193	2.20E-15	*mtlA*
gene-b3624	1.019411691	8.07E-05	*waaZ*
gene-b2082	1.016510003	0.083241757	*ogrK*
gene-b4834	1.016343271	0.001090191	*xylZ*
gene-b0047	1.015862358	2.99E-10	*kefC*
gene-b0305	1.014015055	4.93E-05	*rclR*
gene-b1873	-1.014001547	0.003762581	*torY*
gene-b0266	-1.01393682	1.78E-06	*yagB*
gene-b1436	-1.01299159	8.19E-05	*yncJ*
gene-b2389	1.012206587	1.74E-11	*yfeO*
gene-b2066	-1.012062022	1.41E-06	*udk*
gene-b0415	-1.011869856	4.83E-10	*ribE*
gene-b0036	1.010508749	0.035476895	*caiD*
gene-b0043	1.008700122	0.002818129	*fixC*
gene-b0388	-1.008453887	2.78E-08	*aroL*
gene-b4377	1.007112625	2.30E-15	*yjjU*
gene-b2959	1.006773897	3.11E-09	*yggL*
gene-b0480	1.006619576	2.07E-15	*ushA*
gene-b2601	1.005150867	3.49E-12	*aroF*
gene-b2328	-1.005082313	1.60E-10	*mepA*
gene-b1856	-1.004683258	4.72E-12	*mepM*
gene-b4810	1.004266025	1.95E-10	*ftsO*
gene-b2638	1.002734209	0.059506803	*yfjU*
gene-b0526	-1.001731805	4.17E-16	*cysS*
gene-b3269	1.001318622	0.029222532	*yhdX*
gene-b3596	-1.000312738	0.047998273	*yibG*
gene-b2818	-0.998256485	1.18E-08	*argA*
gene-b1972	0.998246189	4.52E-07	*msrQ*
gene-b1327	0.996890175	1.31E-08	*ycjY*
gene-b3916	0.996780308	1.03E-16	*pfkA*

gene-b4498	-0.995690109	2.90E-11	*gatR*
gene-b4132	-0.995231925	6.74E-05	*cadB*
gene-b2759	0.994815332	0.002058018	*casB*
gene-b4580	0.994340088	6.12E-08	*yaiT*
gene-b3084	-0.993928215	4.23E-07	*rlmG*
gene-b0034	0.993175212	0.004351709	*caiF*
gene-b3872	-0.992204215	2.14E-05	*yihL*
gene-b4077	-0.992076464	1.22E-14	*gltP*
gene-b1919	0.991346792	7.09E-14	*dcyD*
gene-b1275	-0.99134457	8.55E-12	*cysB*
gene-b2290	-0.989616274	2.98E-12	*alaA*
gene-b4018	-0.989315069	1.35E-10	*iclR*
gene-b1086	-0.989175054	1.01E-11	*rluC*
gene-b1116	-0.989036502	3.90E-14	*lolC*
gene-b2714	-0.987811818	3.53E-12	*ascG*
gene-b0477	-0.987726638	2.90E-12	*gsk*
gene-b0898	-0.986721848	1.90E-08	*ycaD*
gene-b0952	0.985941166	3.17E-13	*pqiC*
gene-b3149	-0.98567456	5.90E-14	*diaA*
gene-b2171	-0.985135591	2.22E-15	*yeiP*
gene-b1308	-0.984977772	8.45E-09	*pspE*
gene-b0865	0.984903887	3.41E-12	*ybjP*
gene-b2309	0.984877578	2.78E-07	*hisJ*
gene-b0489	0.984558607	5.22E-10	*qmcA*
gene-b3221	0.982819083	1.39E-10	*nanQ*
gene-b0284	0.982479387	8.13E-09	*paoC*
gene-b0226	0.981451786	8.07E-10	*dinJ*
gene-b1021	0.98142833	0.016683898	*pgaD*
gene-b2461	0.980507386	0.018177971	*eutP*
gene-b1015	0.980245094	0.002705019	*putP*
gene-b0983	-0.980180326	0.001020198	*gfcE*
gene-b4356	0.978964107	3.48E-05	*lgoT*
gene-b4340	-0.97853004	4.64E-07	*yjiR*
gene-b3603	-0.976211051	2.39E-07	*lldP*
gene-b4360	-0.975863787	1.60E-07	*yjjA*
gene-b3830	0.975218188	2.27E-13	*ysgA*
gene-b1647	0.975200536	1.10E-15	*ydhF*
gene-b2449	0.974678148	5.44E-07	*yffR*
gene-b3428	0.973701527	2.25E-16	*glgP*
gene-b3190	-0.973616038	4.96E-06	*ibaG*
gene-b3553	-0.972927783	3.97E-14	*ghrB*
gene-b2269	0.972061956	0.008100736	*elaD*
gene-b3105	-0.971746148	4.54E-10	*yhaJ*
gene-b0376	-0.971316145	7.67E-11	*ampH*
gene-b4307	-0.968134535	0.001870696	*yjhQ*
gene-b0980	0.967149171	9.39E-08	*appA*
gene-b1507	0.966945574	9.64E-07	*hipA*
gene-b1178	0.966779604	1.50E-06	*pliG*
gene-b3023	0.96518296	3.55E-09	*ygiV*
gene-b4212	0.964600054	2.83E-06	*ytfH*
gene-b3114	-0.962475977	5.94E-05	*tdcE*
gene-b3964	-0.962309571	5.50E-11	*yijD*
gene-b2091	-0.962067774	3.93E-14	*gatD*
gene-b0659	0.961129081	2.11E-10	*ybeY*
gene-b1117	-0.959494677	5.88E-11	*lolD*
gene-b2002	-0.958623271	0.187466884	*yeeS*
gene-b2943	0.958314037	1.19E-14	*galP*
gene-b2042	-0.957392969	1.30E-13	*galF*
gene-b3974	-0.957297527	1.09E-10	*coaA*
gene-b1280	-0.955202582	2.27E-09	*lapB*
gene-b1636	-0.955116811	2.29E-13	*pdxY*
gene-b2251	-0.955067332	0.000214924	*nudI*
gene-b2152	-0.954745479	1.52E-07	*yeiB*
gene-b1211	-0.954691127	7.44E-10	*prfA*
gene-b2486	0.954058712	0.000134947	*hyfF*
gene-b3229	-0.953943019	9.72E-13	*sspA*

gene-b0887	-0.953663473	4.31E-10	*cydD*
gene-b3961	-0.953369096	1.32E-11	*oxyR*
gene-b3965	0.953023624	3.49E-14	*trmA*
gene-b3899	-0.952962763	0.0164674	*frvB*
gene-b0940	-0.952930901	0.002515721	*elfC*
gene-b3824	-0.950863824	0.001452883	*rhtB*
gene-b1448	-0.950501346	1.14E-11	*mnaT*
gene-b1221	-0.949820986	7.67E-11	*narL*
gene-b2882	0.948742775	0.007647451	*xanQ*
gene-b4398	-0.947059673	5.99E-11	*creB*
gene-b4292	-0.946583407	3.46E-06	*fecR*
gene-b3092	0.946516497	1.69E-09	*uxaC*
gene-b3155	-0.946408564	0.089487869	*yhbQ*
gene-b0524	-0.94564572	5.90E-09	*lpxH*
gene-b3347	-0.944920983	8.09E-12	*fkpA*
gene-b1171	-0.944906022	1.75E-08	*ymgD*
gene-b3716	-0.944891655	0.078403885	*cbrB*
gene-b4073	-0.944550918	0.004725238	*nrfD*
gene-b1994	-0.943839269	0.000770409	*insH6*
gene-b1772	0.943483523	0.0122067	*ydjH*
gene-b1521	0.943262831	2.33E-06	*uxaB*
gene-b4137	-0.94234474	4.53E-05	*cutA*
gene-b1378	0.941610302	4.53E-09	*ydbK*
gene-b3091	0.940801671	2.18E-08	*uxaA*
gene-b4061	0.940707613	3.47E-11	*pdeC*
gene-b4251	-0.937011001	1.15E-05	*bdcR*
gene-b1287	-0.936275888	0.000461179	*yciW*
gene-b4546	0.935873693	4.54E-05	*ypeB*
gene-b1446	0.935441716	2.80E-08	*ydcY*
gene-b1294	0.935231571	2.73E-11	*sapA*
gene-b2344	0.935147027	6.09E-06	*fadL*
gene-b3873	-0.93495955	1.37E-06	*yihM*
gene-b1298	0.934572558	9.10E-05	*puuD*
gene-b2611	-0.934342933	3.61E-08	*ypjD*
gene-b4353	-0.934090605	0.11772042	*yjiX*
gene-b1845	0.933591765	4.51E-13	*ptrB*
gene-b3410	-0.933468575	0.000203295	*feoC*
gene-b1048	-0.932929673	1.31E-13	*opgG*
gene-b1698	0.932654717	7.10E-05	*ydiR*
gene-b3996	0.932084639	5.71E-11	*nudC*
gene-b1781	-0.932056004	1.55E-12	*yeaE*
gene-b3433	-0.932028313	2.01E-14	*asd*
gene-b1861	-0.930921647	1.23E-07	*ruvA*
gene-b0166	-0.930840851	1.36E-12	*dapD*
gene-b2829	-0.929215336	4.67E-10	*ptsP*
gene-b0419	0.928794347	2.15E-10	*yajO*
gene-b1918	0.92849506	3.18E-11	*tcyL*
gene-b0058	0.927086709	7.77E-12	*rluA*
gene-b2175	-0.926668763	2.66E-06	*mepS*
gene-b3541	0.926389354	5.05E-10	*dppD*
gene-b3600	0.926191834	1.06E-13	*mtlD*
gene-b2830	-0.925948572	1.66E-09	*rppH*
gene-b0694	0.925812612	7.99E-06	*kdpE*
gene-b1261	-0.925744133	1.33E-12	*trpB*
gene-b4122	-0.925586514	4.16E-05	*fumB*
gene-b0775	-0.925147968	2.01E-08	*bioB*
gene-b1447	-0.923814702	0.000277448	*ydcZ*
gene-b0224	-0.923354136	6.58E-09	*dpaA*
gene-b0699	0.922963024	2.73E-05	*ybfA*
gene-b4393	-0.922761352	1.03E-07	*trpR*
gene-b2151	-0.920352388	5.59E-10	*galS*
gene-b2846	0.920304876	0.000115611	*yqeH*
gene-b3150	-0.920125555	3.43E-12	*dolP*
gene-b0421	-0.91971118	3.69E-09	*ispA*
gene-b1616	0.919658096	2.22E-08	*uidB*
gene-b2416	0.91928705	2.95E-14	*ptsI*

gene-b0366	-0.919033218	0.149017175	*tauB*
gene-b1139	0.918027815	5.94E-05	*lit*
gene-b3270	0.917841582	0.041705709	*yhdY*
gene-b2948	-0.9167402	4.59E-10	*yqgE*
gene-b1922	0.915082499	0.06394528	*fliA*
gene-b0537	-0.914849248	2.04E-10	*intD*
gene-b4299	0.914305966	0.000709539	*yjhI*
gene-b2981	-0.911872782	0.027907242	*yghO*
gene-b3211	-0.911547472	0.00021966	*yhcC*
gene-b1333	0.910550304	3.51E-12	*uspE*
gene-b1596	0.908982736	5.72E-07	*ynfM*
gene-b4053	-0.907827325	9.30E-10	*alr*
gene-b4264	-0.905865614	2.67E-07	*idnR*
gene-b0492	-0.904722349	2.03E-13	*cnoX*
gene-b0077	-0.904341599	1.61E-11	*ilvI*
gene-b4003	-0.904300397	7.66E-06	*zraS*
gene-b0807	-0.904293795	2.28E-06	*rlmF*
gene-b3364	0.904145213	0.00351851	*tsgA*
gene-b2550	-0.904129646	1.20E-07	*yphH*
gene-b0469	-0.902787622	1.62E-10	*apt*
gene-b2706	-0.89950897	0.094847357	*gutM*
gene-b3031	-0.899059632	1.99E-06	*yqiA*
gene-b2668	-0.898341885	1.07E-05	*ygaP*
gene-b1093	0.897317827	4.02E-13	*fabG*
gene-b3542	0.895788802	2.47E-09	*dppC*
gene-b1133	-0.894165878	1.57E-08	*mnmA*
gene-b2919	-0.89319956	0.018520628	*scpB*
gene-b0387	-0.893025239	1.00E-07	*yaiI*
gene-b1695	0.891863989	0.018019873	*ydiO*
gene-b0881	0.891790616	1.83E-06	*clpS*
gene-b2181	0.891349894	0.000628037	*yejG*
gene-b2896	-0.890383371	2.31E-10	*ygfX*
gene-b2370	-0.890379237	7.25E-08	*evgS*
gene-b2671	-0.889628653	1.28E-10	*ygaC*
gene-b3416	0.888934296	6.36E-12	*malQ*
gene-b3226	-0.888658237	3.47E-11	*nanR*
gene-b2536	-0.888622546	1.14E-07	*hcaT*
gene-b3417	0.887973564	2.45E-10	*malP*
gene-b0217	-0.887814757	0.103768966	*yafT*
gene-b1790	-0.886936751	0.000334142	*nimR*
gene-b0380	-0.886170681	1.02E-05	*yaiZ*
gene-b4384	0.88528729	3.14E-14	*deoD*
gene-b2484	0.884367326	0.043120288	*hyfD*
gene-b1485	0.883289706	0.00127797	*ddpC*
gene-b1644	-0.882251036	2.56E-05	*ydhJ*
gene-b1357	0.882079433	0.049613371	*ydaS*
gene-b0426	0.881770939	5.91E-10	*yajQ*
gene-b2469	-0.881679591	3.69E-08	*narQ*
gene-b1834	0.880617738	4.18E-13	*letB*
gene-b1765	-0.880294317	2.02E-10	*ydjA*
gene-b4378	0.87842288	1.32E-09	*yjjV*
gene-b0188	-0.877858777	2.79E-09	*tilS*
gene-b0503	-0.877079628	2.04E-06	*selU*
gene-b3359	-0.876908016	1.00E-06	*argD*
gene-b0700	-0.876144257	2.37E-06	*rhsC*
gene-b2094	-0.875552591	9.49E-10	*gatA*
gene-b4084	0.87497549	0.001484965	*alsK*
gene-b3212	-0.874817539	2.36E-08	*gltB*
gene-b1176	-0.873140105	1.88E-10	*minC*
gene-b4483	-0.87278205	3.53E-08	*tatD*
gene-b2316	-0.872780938	5.56E-11	*accD*
gene-b4233	-0.871849994	1.44E-11	*mpl*
gene-b2347	0.871452205	6.43E-06	*yfdC*
gene-b0777	-0.870716393	0.003050704	*bioC*
gene-b0891	-0.870618832	1.50E-12	*lolA*
gene-b1660	-0.870121462	5.35E-07	*punC*

gene-b3473	-0.869175508	8.16E-07	*yhhS*
gene-b1339	-0.86712991	1.71E-08	*abgR*
gene-b3253	0.866860996	1.41E-11	*yhdH*
gene-b1667	0.866601419	1.17E-08	*ydhR*
gene-b2893	-0.866219178	3.89E-08	*dsbC*
gene-b0950	0.866147178	2.17E-10	*pqiA*
gene-b4743	0.866060202	0.053590461	*ynaL*
gene-b1838	0.865955384	0.000186536	*pphA*
gene-b0868	-0.865666763	5.68E-07	*ybjS*
gene-b1690	0.865585666	0.0978056	*ydiM*
gene-b1356	-0.865520312	2.60E-06	*racR*
gene-b1461	0.865511081	0.006229329	*pptA*
gene-b0884	0.865289278	4.73E-06	*infA*
gene-b2494	-0.865214108	6.74E-12	*bepA*
gene-b1262	-0.86428387	1.02E-08	*trpC*
gene-b3741	-0.863884012	3.60E-09	*mnmG*
gene-b2304	0.86181742	2.32E-10	*yfcH*
gene-b2953	-0.859659023	0.005783745	*yggU*
gene-b0588	-0.859520139	2.62E-06	*fepC*
gene-b0904	0.859410704	6.20E-11	*focA*
gene-b2669	0.859382369	0.032702486	*stpA*
gene-b1519	0.858474034	2.73E-09	*tam*
gene-b0517	-0.858439365	0.029095094	*allD*
gene-b4395	-0.857487463	1.28E-08	*ytjC*
gene-b3499	-0.855210635	1.36E-05	*rlmJ*
gene-b1711	-0.854629889	8.94E-07	*btuC*
gene-b0315	0.85461153	0.001069651	*pdeL*
gene-b0056	0.854318987	2.75E-06	*yabP*
gene-b0758	0.851813415	7.18E-08	*galT*
gene-b3152	0.850178726	1.63E-05	*yraR*
gene-b1293	0.849831481	7.68E-06	*sapB*
gene-b4380	-0.847802093	3.49E-06	*yjjI*
gene-b4352	-0.847759795	5.47E-10	*yjiA*
gene-b0608	0.847656914	1.25E-05	*ybdR*
gene-b1727	-0.847499971	5.04E-09	*hxpB*
gene-b0459	-0.847484139	1.35E-06	*maa*
gene-b2537	-0.84715524	0.000171711	*hcaR*
gene-b3425	-0.846026405	0.000776367	*glpE*
gene-b1466	0.844819606	0.000262329	*narW*
gene-b2349	-0.843134039	0.001075684	*intS*
gene-b0261	-0.842816643	1.09E-07	*mmuM*
gene-b4388	-0.841949561	9.53E-11	*serB*
gene-b0560	-0.841617465	0.061025652	*nohD*
gene-b2336	-0.840077499	0.246278046	*yfcS*
gene-b2332	0.83986972	1.48E-06	*yfcO*
gene-b2510	0.838956322	0.108337014	*yfgJ*
gene-b3068	0.837787948	6.70E-09	*mug*
gene-b0651	-0.8376852	7.11E-11	*rihA*
gene-b4544	0.837116433	0.136844799	*arnE*
gene-b4115	0.836583124	0.067061826	*adiC*
gene-b2359	0.834832324	0.03409127	*yfdP*
gene-b3582	0.833833118	0.047602232	*sgbU*
gene-b3538	0.833376432	3.48E-07	*bcsG*
gene-b1958	0.832830812	2.72E-09	*yedI*
gene-b1591	-0.832761146	0.000106703	*dmsD*
gene-b0059	0.832437596	1.78E-09	*rapA*
gene-b3640	-0.832369775	9.84E-08	*dut*
gene-b1808	-0.832201775	5.17E-08	*yoaA*
gene-b2688	0.831893996	5.15E-11	*gshA*
gene-b1562	0.831804576	0.000617132	*hokD*
gene-b0895	-0.830292964	1.74E-05	*dmsB*
gene-b0755	0.829805582	1.96E-07	*gpmA*
gene-b1344	-0.829691587	9.80E-09	*ttcA*
gene-b0808	0.828883194	8.27E-10	*ybiO*
gene-b0442	-0.828186434	8.37E-06	*ybaV*
gene-b3919	0.827899851	5.93E-08	*tpiA*

gene-b4301	0.82764868	0.000293126	*sgcE*
gene-b3429	0.826777222	3.09E-09	*glgA*
gene-b3605	0.826481652	5.15E-07	*lldD*
gene-b1465	0.826351819	3.68E-05	*narV*
gene-b2790	-0.826207725	3.79E-10	*yqcA*
gene-b1127	0.825471587	2.22E-10	*pepT*
gene-b1168	0.82525094	0.003275315	*pdeG*
gene-b0048	0.824958435	2.00E-09	*folA*
gene-b1477	-0.823506478	4.42E-06	*yddM*
gene-b4804	0.823342066	5.22E-11	*rbsZ*
gene-b1273	0.823240046	1.44E-08	*yciN*
gene-b3967	-0.823146334	4.65E-08	*murI*
gene-b2453	0.823073734	0.022852918	*eutG*
gene-b2583	-0.822855055	0.002028409	*tapT*
gene-b2295	0.822703466	2.88E-10	*yfbV*
gene-b4178	0.821716453	7.64E-10	*nsrR*
gene-b2154	-0.820435148	9.98E-12	*yeiG*
gene-b2702	-0.82027542	2.83E-05	*srlA*
gene-b3614	-0.819938086	1.75E-05	*yibQ*
gene-b3890	-0.818419391	0.018704527	*yiiF*
gene-b3689	-0.81801211	1.11E-07	*yidR*
gene-b0955	0.817575097	1.66E-09	*ycbZ*
gene-b3059	-0.817219587	4.13E-07	*plsY*
gene-b3530	0.81703753	4.34E-10	*bcsC*
gene-b1024	-0.816966228	1.68E-05	*pgaA*
gene-b2791	-0.816667079	3.36E-06	*truC*
gene-b3543	0.816132505	1.62E-06	*dppB*
gene-b4306	-0.81592412	0.000456944	*yjhP*
gene-b0704	0.815750899	0.007864556	*ybfC*
gene-b3874	0.815500403	0.001585323	*yihN*
gene-b2442	-0.814184595	0.000124119	*intZ*
gene-b0197	-0.813494753	9.17E-09	*metQ*
gene-b0196	-0.813429569	6.63E-07	*rcsF*
gene-b3934	-0.812719898	2.20E-10	*cytR*
gene-b0598	0.812504586	1.24E-09	*cstA*
gene-b4146	-0.812175295	2.89E-08	*epmB*
gene-b3860	-0.811407183	1.10E-07	*dsbA*
gene-b3928	0.811340849	4.33E-10	*zapB*
gene-b3740	-0.810791725	4.14E-05	*rsmG*
gene-b0495	-0.810202877	8.70E-07	*ybbA*
gene-b0174	-0.809156674	8.90E-07	*ispU*
gene-b4280	0.808179772	0.000312391	*nanY*
gene-b4257	0.8079461	0.070419357	*yjgN*
gene-b3567	0.806173193	1.39E-06	*xylG*
gene-b0070	-0.804756636	3.46E-08	*setA*
gene-b3165	0.804610015	1.30E-07	*rpsO*
gene-b3936	0.804511704	5.04E-08	*rpmE*
gene-b0214	-0.804176802	8.18E-05	*rnhA*
gene-b0709	-0.803405204	3.96E-08	*dtpD*
gene-b3087	0.802612607	4.55E-11	*ygjR*
gene-b3925	0.802426848	9.21E-10	*glpX*
gene-b0861	0.802414491	7.83E-08	*artM*
gene-b2945	0.801966055	0.011714807	*endA*
gene-b0862	0.801546161	1.07E-08	*artQ*
gene-b0143	-0.800785825	4.78E-08	*pcnB*
gene-b1483	0.800697089	0.00114253	*ddpF*
gene-b0894	-0.799754735	2.00E-05	*dmsA*
gene-b3558	0.799526573	0.031406305	*insK*
gene-b1657	0.798743993	3.60E-07	*ydhP*
gene-b3018	-0.798536913	5.27E-06	*plsC*
gene-b0708	0.7984665	3.36E-06	*phr*
gene-b2738	0.798259502	0.005264449	*ygbL*
gene-b3839	-0.798203467	5.37E-08	*tatC*
gene-b1464	-0.797764092	1.00E-07	*yddE*
gene-b0892	-0.797210905	1.54E-09	*rarA*
gene-b3878	0.796959042	0.000617586	*yihQ*

gene-b4529	0.796770634	1.76E-07	*ydbJ*
gene-b0837	0.795426383	1.34E-06	*yliI*
gene-b0995	-0.794717505	3.31E-05	*torR*
gene-b3390	-0.794576117	1.60E-09	*aroK*
gene-b2311	-0.793261705	6.00E-06	*ubiX*
gene-b3251	-0.792209441	2.15E-10	*mreB*
gene-b1542	0.791809822	0.001779352	*ydfI*
gene-b0885	-0.790517749	3.18E-06	*aat*
gene-b1035	-0.790448583	8.43E-08	*ycdY*
gene-b3528	-0.790244245	4.40E-10	*dctA*
gene-b1342	0.790116819	6.94E-09	*zntB*
gene-b0498	-0.788918804	0.251431563	*ybbC*
gene-b1786	-0.78808411	2.45E-05	*dgcJ*
gene-b4139	0.787856249	3.00E-09	*aspA*
gene-b2148	0.787775887	1.07E-06	*mglC*
gene-b3866	0.787550386	1.29E-07	*yihI*
gene-b0928	-0.787182353	1.65E-09	*aspC*
gene-b4557	-0.786684734	0.015286725	*yidD*
gene-b2957	-0.786479753	0.00042002	*ansB*
gene-b3003	0.786474484	1.70E-05	*yghA*
gene-b3972	-0.786263486	1.83E-08	*murB*
gene-b3560	-0.784632942	1.37E-08	*glyQ*
gene-b0080	-0.784287828	3.80E-08	*cra*
gene-b1940	-0.78420751	0.085252089	*fliH*
gene-b1256	0.783664251	0.001491821	*ompW*
gene-b4023	-0.782852209	4.62E-06	*yjbD*
gene-b2912	-0.782666202	0.001072828	*fau*
gene-b3455	0.782469857	0.072147613	*livG*
gene-b1137	-0.781800381	0.016700387	*ymfD*
gene-b1183	0.781549748	0.001471991	*umuD*
gene-b2098	0.779648002	5.87E-07	*yegT*
gene-b1804	-0.778745083	4.01E-06	*rnd*
gene-b2342	-0.778267056	2.32E-07	*fadI*
gene-b4095	0.778200109	0.137055669	*phnM*
gene-b1222	-0.777582149	1.41E-05	*narX*
gene-b4476	0.77744414	0.002257825	*gntU*
gene-b2528	0.777314945	1.30E-08	*iscA*
gene-b1044	0.776700309	0.02704302	*ymdA*
gene-b0587	0.775730383	0.089005561	*fepE*
gene-b3172	0.775442695	5.37E-10	*argG*
gene-b3470	-0.774817213	0.003834995	*tusA*
gene-b2101	-0.77463403	8.90E-07	*yegW*
gene-b3232	-0.773962379	3.55E-08	*zapE*
gene-b0529	-0.773486761	3.32E-09	*folD*
gene-b1025	-0.773311753	2.06E-06	*dgcT*
gene-b0107	-0.773137715	0.001348176	*hofB*
gene-b3029	0.77210812	7.24E-08	*ygiN*
gene-b4093	0.771672376	0.011291559	*phnO*
gene-b2161	0.771506956	0.001997633	*nupX*
gene-b2095	-0.771079207	3.95E-08	*gatZ*
gene-b0863	0.770703067	5.18E-10	*artI*
gene-b0438	0.770422742	4.65E-11	*clpX*
gene-b3574	-0.7691455	4.60E-08	*plaR*
gene-b3213	-0.769113013	1.00E-07	*gltD*
gene-b3564	0.768671486	2.13E-05	*xylB*
gene-b3838	-0.768342912	2.64E-06	*tatB*
gene-b4087	0.768120727	0.005598977	*alsA*
gene-b1311	0.767414574	0.111905359	*ycjO*
gene-b1424	-0.76717476	1.16E-09	*opgD*
gene-b1136	-0.767107957	1.11E-09	*icd*
gene-b0675	-0.766685298	1.85E-09	*umpH*
gene-b1738	0.766522484	0.000390815	*chbB*
gene-b1827	0.766470366	1.07E-08	*kdgR*
gene-b2468	-0.765820339	9.30E-06	*aegA*
gene-b0823	0.76534988	2.23E-06	*ybiW*
gene-b4471	-0.764882917	1.07E-05	*tdcG*

gene-b3540	0.76447878	5.47E-09	*dppF*
gene-b2360	0.763731443	0.015605132	*yfdQ*
gene-b2206	-0.763717536	0.000538768	*napA*
gene-b1564	0.763555844	2.79E-06	*relB*
gene-b2326	-0.763532888	1.76E-06	*epmC*
gene-b2093	-0.762942558	1.52E-09	*gatB*
gene-b4125	-0.761511402	1.04E-08	*dcuS*
gene-b4239	-0.760612184	0.000436468	*treC*
gene-b4032	0.760588185	1.96E-05	*malG*
gene-b1611	0.759997841	2.02E-09	*fumC*
gene-b3058	-0.759117547	0.001694885	*folB*
gene-b3625	0.758615913	0.001228484	*waaY*
gene-b3160	0.758240254	1.30E-05	*yhbW*
gene-b1260	-0.757675716	4.40E-09	*trpA*
gene-b3822	-0.757330236	5.78E-05	*recQ*
gene-b2011	-0.757306522	1.98E-08	*sbcB*
gene-b0583	-0.756008583	0.054902994	*entD*
gene-b0879	0.755981411	4.35E-10	*macB*
gene-b3906	-0.753953737	0.010367292	*rhaR*
gene-b3891	-0.75335892	1.95E-08	*fdhE*
gene-b3612	0.753059314	1.21E-09	*gpmM*
gene-b1914	-0.752520735	8.13E-08	*uvrY*
gene-b3571	0.752371793	0.000606247	*malS*
gene-b3584	0.752348523	0.011615391	*yiaT*
gene-b1602	0.751767253	1.07E-09	*pntB*
gene-b1561	0.751450506	0.176459689	*rem*
gene-b2977	0.750564737	2.40E-07	*glcG*
gene-b2447	0.749570769	0.009358664	*yffP*
gene-b0579	-0.749166352	0.001064659	*ybdF*
gene-b1087	0.749074187	1.26E-09	*yceF*
gene-b0445	0.748501282	3.17E-07	*ybaE*
gene-b2579	0.748394308	1.55E-06	*grcA*
gene-b2868	0.748203778	0.002439069	*xdhC*
gene-b2721	0.747079739	0.019731158	*hycE*
gene-b4374	0.746948758	1.96E-06	*yjjG*
gene-b0321	0.746887882	0.004943337	*yahG*
gene-b1688	0.746411548	1.68E-07	*ydiK*
gene-b1473	-0.746247546	0.00963562	*yddG*
gene-b0956	0.745978284	7.30E-06	*matP*
gene-b0414	-0.745739765	3.88E-06	*ribD*
gene-b0018	0.745484186	0.011788242	*mokC*
gene-b3430	0.745318345	2.52E-08	*glgC*
gene-b2113	-0.745302513	3.62E-08	*mrp*
gene-b0182	-0.745135969	2.29E-06	*lpxB*
gene-b1046	0.745134423	2.80E-07	*clsC*
gene-b0520	0.745065617	0.110499976	*ylbF*
gene-b1760	0.745016014	0.006412171	*ynjH*
gene-b1563	0.743120429	2.79E-07	*relE*
gene-b3182	-0.743040555	8.83E-06	*dacB*
gene-b3589	0.742743272	0.217514749	*yiaY*
gene-b1064	0.742459891	1.71E-08	*grxB*
gene-b0357	0.741673289	0.026287617	*frmR*
gene-b1628	-0.741026037	0.001525989	*rsxB*
gene-b1547	-0.740633304	0.167771423	*stfQ*
gene-b3593	-0.738616395	3.74E-05	*rhsA*
gene-b1468	0.738547067	0.000201351	*narZ*
gene-b2955	-0.738312028	4.23E-07	*hemW*
gene-b1135	-0.737763876	0.000832522	*rluE*
gene-b4386	-0.737497051	3.17E-05	*lplA*
gene-b1467	0.737156772	0.000227689	*narY*
gene-b4373	0.737123285	1.29E-05	*rimI*
gene-b2605	-0.736771838	0.000685252	*yfiB*
gene-b3544	0.736533143	6.33E-06	*dppA*
gene-b3129	0.735965445	0.000126614	*prlF*
gene-b2335	-0.734918607	0.189789999	*yfcR*
gene-b1217	0.734415246	8.71E-05	*chaB*

gene-b2685	-0.734247058	4.21E-05	*emrA*
gene-b2522	-0.73421291	8.13E-06	*sseB*
gene-b4432	-0.734208253	1.39E-07	*ryeA*
gene-b4160	-0.733196151	5.77E-09	*psd*
gene-b4225	-0.733009922	8.69E-05	*chpB*
gene-b0160	-0.732683132	1.59E-07	*dgt*
gene-b2538	-0.731421388	9.99E-07	*hcaE*
gene-b0514	0.731368767	0.001414543	*glxK*
gene-b4090	-0.730954763	3.39E-06	*rpiB*
gene-b3431	0.730054788	2.57E-08	*glgX*
gene-b0951	0.729871164	2.51E-09	*pqiB*
gene-b0128	0.729012506	1.16E-06	*yadH*
gene-b2412	0.728250472	3.17E-09	*zipA*
gene-b2783	0.727754973	0.002396008	*mazE*
gene-b0416	-0.727061364	1.44E-07	*nusB*
gene-b2594	0.726781157	3.13E-08	*rluD*
gene-b4558	-0.726083074	0.000127865	*yifL*
gene-b1299	0.726050754	0.000881156	*puuR*
gene-b2585	0.726011736	2.58E-09	*pssA*
gene-b3123	-0.725048091	1.16E-08	*rnpB*
gene-b4537	0.72484348	0.001028556	*yecJ*
gene-b4400	0.723895486	0.000145714	*creD*
gene-b4333	-0.723165218	0.103827151	*yjiK*
gene-b1642	0.722595093	4.93E-08	*slyA*
gene-b1363	-0.720906158	0.000417044	*trkG*
gene-b2329	-0.720012279	3.07E-08	*aroC*
gene-b0714	-0.719970583	0.000490974	*nei*
gene-b1123	0.718885871	4.47E-07	*potD*
gene-b0513	0.717624659	0.004012869	*ybbY*
gene-b2443	0.716998487	0.003730827	*yffL*
gene-b2670	0.716340037	0.000137286	*alaE*
gene-b0910	0.71632339	2.77E-08	*cmk*
gene-b3962	-0.715238731	1.68E-06	*sthA*
gene-b0970	-0.714974737	2.44E-07	*yccA*
gene-b0277	0.713783839	0.046043533	*yagK*
gene-b0396	-0.713556585	3.66E-07	*araJ*
gene-b1723	0.712942266	2.96E-07	*pfkB*
gene-b1034	-0.712734978	3.54E-07	*ycdX*
gene-b1874	0.712328341	9.51E-08	*cutC*
gene-b0060	0.712267942	5.87E-06	*polB*
gene-b4186	0.711227308	0.042557559	*yjfC*
gene-b1609	-0.711187957	9.63E-06	*rstB*
gene-b0909	0.710560129	0.000909041	*ycaL*
gene-b3395	-0.70936631	0.000187055	*hofM*
gene-b2777	0.708856474	4.55E-07	*queE*
gene-b3005	-0.708620181	6.28E-05	*exbD*
gene-b2301	0.708114145	1.74E-06	*yfcF*
gene-b2887	-0.70800164	0.019934746	*ygfT*
gene-b1124	0.707937675	1.48E-05	*potC*
gene-b4572	0.706294006	0.09597399	*ylbE*
gene-b0109	-0.706081772	2.76E-07	*nadC*
gene-b2963	-0.705854305	1.74E-06	*mltC*
gene-b1955	0.705764177	0.000429591	*yedP*
gene-b2551	-0.705426488	5.48E-09	*glyA*
gene-b3001	0.705082421	1.39E-08	*gpr*
gene-b1002	0.704282515	1.07E-07	*agp*
gene-b2523	-0.703491759	3.04E-06	*pepB*
gene-b3407	-0.702140755	7.54E-07	*yhgF*
gene-b2499	0.701890068	0.001052564	*purM*
gene-b0779	0.70131037	6.45E-09	*uvrB*
gene-b0420	-0.700707871	5.91E-07	*dxs*
gene-b4039	-0.700365996	4.50E-05	*ubiC*
gene-b3610	0.700126407	3.15E-06	*grxC*
gene-b3725	-0.700041047	1.01E-05	*pstB*
gene-b0190	-0.699945694	8.55E-05	*yaeQ*
gene-b3466	-0.699819805	0.005716455	*yhhL*

gene-b2200	-0.699466969	0.009438375	*ccmB*
gene-b1590	0.699409704	7.26E-05	*ynfH*
gene-b1962	-0.699118603	1.76E-06	*yedJ*
gene-b0844	-0.698464479	9.65E-05	*ybjI*
gene-b4054	-0.696390449	4.94E-08	*tyrB*
gene-b2128	0.696239448	0.000315548	*yehW*
gene-b0065	-0.695114389	1.02E-06	*yabI*
gene-b0719	0.694931653	0.161259497	*ybgD*
gene-b1498	0.69489563	0.010671569	*ydeN*
gene-b2875	0.694767047	0.008842651	*yqeB*
gene-b2587	0.694217503	3.24E-05	*kgtP*
gene-b3879	-0.693143357	0.153855961	*yihR*
gene-b0839	0.693039715	2.71E-08	*dacC*
gene-b3437	0.692677212	0.000432584	*gntK*
gene-b1733	-0.69259995	0.00049773	*chbG*
gene-b3898	-0.692015537	0.02033669	*frvX*
gene-b3858	-0.691438673	0.001440828	*yihD*
gene-b2039	0.691415045	6.67E-05	*rfbA*
gene-b3412	-0.690814318	3.24E-06	*bioH*
gene-b0846	-0.690606954	0.000901295	*rcdA*
gene-b1732	0.690225553	3.65E-05	*katE*
gene-b1208	-0.689744928	1.37E-06	*ispE*
gene-b0145	0.689441392	1.87E-08	*dksA*
gene-b0386	0.689297215	1.22E-05	*proC*
gene-b2980	-0.68844261	2.01E-05	*glcC*
gene-b3639	-0.687606057	5.19E-07	*dfp*
gene-b2035	0.68662416	0.00015955	*wbbH*
gene-b0130	-0.686590228	0.001260865	*yadE*
gene-b2960	0.68616143	4.55E-08	*trmB*
gene-b1613	-0.685831997	1.61E-07	*manA*
gene-b1860	-0.685289563	1.71E-07	*ruvB*
gene-b0338	-0.684704625	0.027381012	*cynR*
gene-b3780	-0.683520272	3.18E-08	*rhlB*
gene-b2871	-0.68144791	0.112971366	*ygeX*
gene-b3704	-0.681042847	0.00549877	*rnpA*
gene-b1621	-0.680820829	2.76E-05	*malX*
gene-b1404	-0.680140071	2.37E-05	*insI2*
gene-b4284	-0.680139663	2.38E-05	*insI3*
gene-b0797	-0.68009932	0.004393006	*rhlE*
gene-b1263	-0.67989771	0.00011168	*trpD*
gene-b0223	-0.679483772	1.95E-05	*yafJ*
gene-b2010	-0.679072528	0.000437623	*dacD*
gene-b4623	-0.678717871	0.032936231	*insO*
gene-b0172	0.677236098	9.22E-07	*frr*
gene-b2933	0.676586458	0.030849115	*cmtA*
gene-b3779	-0.675990668	1.62E-06	*gpp*
gene-b4024	-0.674774918	0.000147628	*lysC*
gene-b4131	0.674745303	0.031081161	*cadA*
gene-b0267	0.67285411	0.166326285	*yagA*
gene-b0957	0.672444881	2.81E-08	*ompA*
gene-b2307	0.672427797	0.001281603	*hisM*
gene-b2448	0.671979615	0.206251011	*yffQ*
gene-b1410	-0.671861794	0.002139665	*ynbC*
gene-b2742	-0.67145614	3.80E-08	*nlpD*
gene-b3339	-0.671177827	3.17E-08	*tufA*
gene-b0283	0.670948446	0.001346566	*paoD*
gene-b0020	-0.670907335	1.76E-06	*nhaR*
gene-b4464	0.669880213	0.000250386	*ghxQ*
gene-b2926	0.66981652	4.15E-08	*pgk*
gene-b2089	-0.668844456	0.007656619	*insF5*
gene-b1026	-0.668843946	0.007664398	*insF4*
gene-b0541	-0.668843512	0.00767102	*insF3*
gene-b0372	-0.66884302	0.007678539	*insF2*
gene-b0299	-0.66884255	0.007685724	*insF1*
gene-b3038	-0.668789176	2.24E-05	*ygiC*
gene-b0487	0.668737121	1.24E-05	*cueR*

gene-b0152	-0.668208146	0.000156442	*fhuD*
gene-b0712	0.667609491	3.69E-06	*pxpC*
gene-b3955	-0.667584467	9.98E-06	*eptC*
gene-b0042	0.667262297	0.117918458	*fixB*
gene-b1069	-0.666672168	6.67E-05	*murJ*
gene-b4168	0.665548246	1.90E-05	*tsaE*
gene-b2118	-0.66510534	0.003799865	*yehI*
gene-b2040	0.664735344	8.49E-06	*rfbD*
gene-b2244	-0.664552588	0.035447245	*rpnE*
gene-b3650	-0.663417686	4.37E-07	*spoT*
gene-b0761	-0.663125423	0.000112241	*modE*
gene-b0680	-0.662525634	5.10E-08	*glnS*
gene-b4302	0.661739226	0.054838711	*sgcA*
gene-b0766	-0.66170412	6.38E-06	*ybhA*
gene-b3142	0.661365216	0.146994098	*yraH*
gene-b1802	0.661355111	0.022506727	*yeaW*
gene-b2278	0.661253153	1.61E-06	*nuoL*
gene-b1970	0.660810117	0.017717288	*hiuH*
gene-b3945	-0.658382359	2.46E-07	*gldA*
gene-b2865	-0.658358631	0.001333172	*actS*
gene-b3805	-0.658293578	1.70E-07	*hemC*
gene-b0478	0.656470371	1.52E-07	*ybaL*
gene-b1065	-0.65630227	0.032207475	*mdtH*
gene-b2615	-0.656285029	5.43E-06	*nadK*
gene-b0658	0.656220891	5.13E-06	*ybeX*
gene-b1321	-0.655116024	3.65E-05	*ycjX*
gene-b3793	0.65453151	0.000765361	*wzyE*
gene-b2188	-0.653402836	2.58E-05	*lapC*
gene-b3345	-0.653392686	3.74E-05	*tusD*
gene-b4364	-0.652761796	0.007949172	*yjjP*
gene-b2520	0.651093231	1.11E-06	*yfhM*
gene-b2382	-0.651061702	0.000158844	*ypdC*
gene-b4337	0.650997754	2.54E-05	*mdtM*
gene-b0068	-0.649567184	0.003685097	*thiB*
gene-b1103	0.648699761	9.13E-05	*hinT*
gene-b1279	-0.648533143	0.000212898	*lapA*
gene-b3745	0.648466522	2.83E-06	*viaA*
gene-b0518	0.648311042	0.245550682	*fdrA*
gene-b1163	-0.648083366	0.000478277	*bluF*
gene-b0687	-0.648052679	1.48E-06	*seqA*
gene-b1063	-0.647257195	2.74E-06	*yceB*
gene-b2022	-0.646718765	3.88E-06	*hisB*
gene-b1879	-0.646017496	0.003335354	*flhA*
gene-b4571	0.645636997	3.46E-05	*wbbL*
gene-b3409	-0.644798995	6.22E-05	*feoB*
gene-b0886	-0.64459486	3.83E-06	*cydC*
gene-b0635	-0.643242141	0.000151659	*mrdA*
gene-b3825	-0.643024027	0.000578981	*pldB*
gene-b3685	-0.64293898	1.41E-05	*yidE*
gene-b2477	-0.641764452	1.40E-07	*bamC*
gene-b1324	-0.641674825	2.94E-06	*tpx*
gene-b3990	0.641575539	0.025174281	*thiH*
gene-b3635	-0.641533698	0.00230474	*mutM*
gene-b3326	-0.641438525	0.273822427	*gspE*
gene-b2838	0.64139929	0.007533078	*lysA*
gene-b0859	-0.64134225	0.00027182	*rlmC*
gene-b0286	0.640323141	0.001854867	*paoA*
gene-b1764	-0.640214411	1.71E-05	*selD*
gene-b1714	-0.639797175	5.07E-07	*pheS*
gene-b3147	-0.63911259	2.00E-07	*lpoA*
gene-b4089	-0.638442984	1.18E-05	*alsR*
gene-b3158	-0.638223647	0.117630285	*ubiU*
gene-b2758	0.638120436	0.014726458	*casC*
gene-b2411	0.637454268	6.30E-07	*ligA*
gene-b4166	-0.637257956	0.000483175	*queG*
gene-b0584	-0.636779033	0.000380051	*fepA*

gene-b2925	0.636268547	5.84E-07	*fbaA*
gene-b3356	-0.636173317	1.65E-06	*yhfA*
gene-b2325	-0.636125083	0.000158831	*yfcL*
gene-b0187	-0.636092076	7.73E-05	*yaeR*
gene-b0958	0.636050261	6.67E-06	*sulA*
gene-b1062	-0.635780728	0.000659624	*pyrC*
gene-b3011	-0.635710061	0.00035348	*yqhD*
gene-b2600	0.635399919	2.75E-06	*tyrA*
gene-b2071	0.634911852	0.212862072	*yegJ*
gene-b0818	-0.634382897	0.000286482	*ybiR*
gene-b4312	-0.634186648	0.015908181	*fimB*
gene-b1534	-0.634053875	7.61E-05	*ydeE*
gene-b4025	0.633386212	1.51E-07	*pgi*
gene-b3124	0.632376919	7.67E-05	*garK*
gene-b0774	-0.63159197	0.000738467	*bioA*
gene-b2140	-0.631309742	0.002033042	*dusC*
gene-b3539	-0.630699619	0.05518687	*yhjV*
gene-b3973	-0.630231677	4.74E-06	*birA*
gene-b3387	-0.630133433	5.28E-05	*dam*
gene-b3497	0.630085587	0.000166957	*rsmJ*
gene-b0323	0.629856532	0.217398502	*yahI*
gene-b0773	0.62952807	2.09E-06	*ybhB*
gene-b1622	-0.627994752	1.61E-06	*malY*
gene-b0577	-0.627949776	2.48E-05	*ybdG*
gene-b0778	-0.62738769	0.005831192	*bioD*
gene-b2786	-0.626981443	4.03E-06	*barA*
gene-b0851	-0.626957204	0.000101574	*nfsA*
gene-b3125	0.626444885	0.000155834	*garR*
gene-b2593	0.626336257	1.94E-05	*yfiH*
gene-b3933	-0.626272457	1.16E-06	*ftsN*
gene-b3179	0.625017703	6.89E-07	*rlmE*
gene-b3118	-0.624640516	0.001025786	*tdcA*
gene-b3601	0.6243909	3.45E-06	*mtlR*
gene-b3250	-0.624381613	0.00021438	*mreC*
gene-b3335	0.624201473	0.086806123	*gspO*
gene-b1708	0.624174106	1.01E-05	*nlpC*
gene-b2063	0.624121326	4.65E-06	*yegH*
gene-b3322	0.623825248	0.062016062	*gspB*
gene-b1282	-0.623644882	0.015230475	*yciH*
gene-b0134	-0.623489032	5.99E-07	*panB*
gene-b1855	-0.62323379	7.64E-07	*lpxM*
gene-b3765	-0.622868311	0.008097854	*yifB*
gene-b2173	-0.622788504	8.78E-06	*yeiR*
gene-b0183	-0.621651741	0.00179531	*rnhB*
gene-b0569	0.621399074	0.000212969	*nfrB*
gene-b1774	0.621354135	0.035468747	*ydjJ*
gene-b1427	-0.620473066	0.000664395	*rimL*
gene-b0845	-0.620206967	7.12E-05	*ybjJ*
gene-b3750	0.619686909	9.35E-06	*rbsC*
gene-b0369	-0.618693663	1.81E-06	*hemB*
gene-b0460	-0.618522774	0.00318659	*hha*
gene-b0589	-0.618114041	0.000249232	*fepG*
gene-b0826	-0.618070597	4.75E-06	*moeB*
gene-b1770	0.618058933	0.0002646	*ydjF*
gene-b0002	-0.617733131	1.03E-06	*thrA*
gene-b2832	0.617639682	0.00249625	*ygdQ*
gene-b1029	0.617453678	0.274306416	*ycdU*
gene-b2920	-0.617296236	0.046624048	*scpC*
gene-b1825	0.617115928	0.022071837	*yebO*
gene-b2432	-0.616755169	6.71E-05	*yfeY*
gene-b4396	0.616701573	1.63E-06	*rob*
gene-b1638	-0.616661183	2.53E-06	*pdxH*
gene-b2038	0.616659863	0.000279413	*rfbC*
gene-b2395	-0.616610296	0.000159789	*pdeA*
gene-b3115	-0.61653438	0.039485822	*tdcD*
gene-b0006	-0.616307729	7.21E-06	*yaaA*

gene-b4019	-0.615859751	1.56E-05	*metH*
gene-b3408	-0.615615487	0.00962455	*feoA*
gene-b2886	0.615188793	0.211273953	*ygfS*
gene-b3042	-0.613887343	6.38E-05	*ubiK*
gene-b2131	0.613280887	8.01E-06	*osmF*
gene-b2373	-0.612980919	0.11914772	*oxc*
gene-b1452	-0.612733409	0.00207392	*yncE*
gene-b4381	0.612641556	4.42E-05	*deoC*
gene-b2223	0.612383461	0.220428002	*atoE*
gene-b0961	-0.612288832	0.003828485	*yccF*
gene-b0532	-0.612142928	0.13859689	*sfmD*
gene-b2617	-0.612082377	3.34E-05	*bamE*
gene-b1245	0.611976472	8.04E-07	*oppC*
gene-b4207	-0.611127899	2.74E-05	*fklB*
gene-b1061	0.610979956	0.011844492	*dinI*
gene-b2517	0.610612898	2.26E-06	*rlmN*
gene-b0443	0.608927	1.40E-05	*fadM*
gene-b3623	0.608492253	0.003105032	*waaU*
gene-b0827	-0.607938185	0.000137082	*moeA*
gene-b1728	0.607700029	0.060763545	*ydjM*
gene-b3163	-0.607175673	1.11E-05	*nlpI*
gene-b3591	-0.607071507	0.000525412	*selA*
gene-b2769	-0.606912355	0.037240895	*ygcQ*
gene-b1532	0.606891171	0.362388206	*marB*
gene-b3547	0.60663443	0.001438673	*yhjX*
gene-b2276	0.606101529	1.43E-06	*nuoN*
gene-b2282	0.605858472	6.00E-06	*nuoH*
gene-b2727	-0.605811023	0.012848247	*hypB*
gene-b4150	-0.60567738	0.001115112	*ampC*
gene-b3228	-0.604437266	7.82E-06	*sspB*
gene-b2964	0.604185633	2.73E-05	*nupG*
gene-b4111	0.603982094	4.32E-06	*proP*
gene-b1755	0.603228966	0.006134494	*ynjC*
gene-b0566	-0.60298283	0.146261836	*envY*
gene-b3351	-0.602737639	0.000561182	*kefG*
gene-b3010	0.602337464	0.001202427	*yqhC*
gene-b2891	-0.602061862	4.28E-05	*prfB*
gene-b0132	-0.601250963	0.056437819	*rpnC*
gene-b4016	0.601028455	1.57E-06	*aceK*
gene-b1763	-0.600520287	3.47E-05	*topB*
gene-b2565	0.600426423	0.000619105	*recO*
gene-b3148	-0.599997767	0.001048894	*yraN*
gene-b4323	0.599560278	5.93E-05	*uxuB*
gene-b1291	0.59929021	2.29E-05	*sapD*
gene-b3992	0.598819988	0.080520011	*thiF*
gene-b3082	0.598776591	0.022118013	*higA*
gene-b3214	-0.598768897	0.141436696	*gltF*
gene-b1105	0.598219563	3.66E-06	*lpoB*
gene-b2478	-0.5980574	2.88E-06	*dapA*
gene-b3137	0.597584165	0.292679525	*kbaY*
gene-b2008	0.596604553	0.002476137	*yeeA*
gene-b0025	-0.596241036	7.22E-05	*ribF*
gene-b0052	-0.595195256	0.00010969	*pdxA*
gene-b0692	0.594763324	5.09E-05	*potE*
gene-b3189	-0.594698111	3.35E-07	*murA*
gene-b1104	0.594320499	3.37E-05	*ycfL*
gene-b1361	0.594044286	0.011175179	*ydaW*
gene-b0452	-0.593829812	4.61E-05	*tesB*
gene-b1839	-0.593826184	0.000784358	*yebY*
gene-b2837	-0.593445335	4.36E-05	*galR*
gene-b0167	-0.593305514	1.60E-05	*glnD*
gene-b2384	0.592863187	0.103136125	*ypdE*
gene-b1520	-0.592391641	0.190137634	*yneE*
gene-b2733	-0.591587307	1.39E-05	*mutS*
gene-b3781	-0.591454454	2.95E-05	*trxA*
gene-b2580	-0.591109493	9.93E-06	*ung*

gene-b3456	0.590954574	0.025658486	*livM*
gene-b2910	0.590667443	0.000228166	*zapA*
gene-b1807	-0.590496914	0.000258076	*tsaB*
gene-b1414	-0.590119426	9.44E-05	*ydcF*
gene-b0231	0.589729276	5.32E-05	*dinB*
gene-b2604	-0.589039549	0.027484714	*dgcN*
gene-b0794	-0.588627741	9.42E-05	*ybhF*
gene-b4443	-0.58825999	0.000581086	*gcvB*
gene-b3110	-0.587859655	0.114962694	*cyuP*
gene-b2020	-0.587635717	1.00E-04	*hisD*
gene-b0838	-0.58712807	1.12E-06	*gstB*
gene-b1641	0.587008163	6.27E-06	*slyB*
gene-b4030	-0.586715292	0.220950027	*psiE*
gene-b0401	0.586639295	2.96E-06	*brnQ*
gene-b1313	0.585968543	0.010551827	*ycjQ*
gene-b0269	0.58579042	8.74E-06	*yagF*
gene-b3950	0.585477725	0.084711007	*frwB*
gene-b3432	0.585037396	5.96E-05	*glgB*
gene-b2236	0.584366117	0.06068727	*yfaE*
gene-b4350	-0.584340113	0.00051282	*hsdR*
gene-b0101	0.58373406	0.032098789	*yacG*
gene-b1976	-0.583207718	4.61E-06	*mtfA*
gene-b1835	-0.582969294	0.000497324	*rsmF*
gene-b0349	-0.582812975	0.002588244	*mhpC*
gene-b3145	0.582715485	0.028444496	*yraK*
gene-b2470	-0.582201858	7.51E-05	*acrD*
gene-b3025	-0.582019874	7.45E-05	*qseB*
gene-b3467	-0.581076936	0.003196612	*yhhM*
gene-b4224	-0.581014496	0.022618581	*chpS*
gene-b3778	-0.579754399	4.33E-05	*rep*
gene-b2525	-0.579516624	0.001474056	*fdx*
gene-b0576	-0.577786246	0.000371028	*pheP*
gene-b3078	0.577399317	0.032002435	*ygjI*
gene-b4327	-0.577341371	0.002038276	*hypT*
gene-b1131	0.576341538	0.000793692	*purB*
gene-b1126	-0.576044171	4.75E-05	*potA*
gene-b2323	-0.576038968	7.87E-06	*fabB*
gene-b0383	0.575548626	0.000288968	*phoA*
gene-b2996	-0.575084507	0.071738756	*hybA*
gene-b3151	-0.575078732	0.001579708	*yraQ*
gene-b1098	-0.575039119	0.000150289	*tmk*
gene-b2929	-0.574869932	0.002228662	*fumE*
gene-b3724	-0.574599117	7.20E-05	*phoU*
gene-b0030	-0.57452776	0.000170761	*rihC*
gene-b3943	-0.57452208	0.004820492	*yijE*
gene-b3354	0.573628272	0.243849907	*yheU*
gene-b2810	-0.573597374	0.000299456	*csdA*
gene-b2318	-0.573594699	1.87E-05	*truA*
gene-b2125	-0.573406508	8.70E-05	*btsR*
gene-b1303	-0.572745759	0.000409632	*pspF*
gene-b1853	0.572241046	2.08E-05	*yebK*
gene-b0796	-0.572034479	0.002781435	*cecR*
gene-b3669	-0.570759696	0.004525259	*uhpA*
gene-b2490	-0.570561541	0.423922047	*hyfJ*
gene-b2024	-0.570066038	0.000390752	*hisA*
gene-b3668	-0.56985372	0.003434154	*uhpB*
gene-b2398	-0.567632955	0.028230746	*yfeC*
gene-b2120	-0.567574086	0.1377291	*yehM*
gene-b4219	0.567458863	4.18E-06	*msrA*
gene-b2219	0.566410821	0.000379465	*atoS*
gene-b2950	-0.566298207	0.006877775	*yggR*
gene-b2184	-0.565706396	0.002809045	*radD*
gene-b0817	-0.565683777	0.002145648	*mntR*
gene-b0809	-0.565412796	8.01E-06	*glnQ*
gene-b1735	-0.565400746	0.003011219	*chbR*
gene-b3813	-0.565377244	0.000128011	*uvrD*

gene-b0633	-0.565340044	1.14E-05	*rlpA*
gene-b1370	-0.564988107	0.004800018	*insH5*
gene-b0771	0.56413285	0.013615953	*ybhJ*
gene-b2804	0.563671853	0.039601991	*fucU*
gene-b3631	0.563541942	4.24E-05	*waaG*
gene-b3208	-0.563504983	0.001164753	*mtgA*
gene-b1433	0.563318043	0.010324357	*ydcO*
gene-b3959	0.562914012	3.26E-05	*argB*
gene-b0840	-0.561514884	0.000179235	*deoR*
gene-b2122	0.56071732	0.090861578	*yehQ*
gene-b2690	-0.557897999	0.000414407	*yqaB*
gene-b0933	0.557601531	0.042925887	*ssuB*
gene-b4243	-0.55715588	4.81E-05	*ridA*
gene-b3346	-0.557132869	1.86E-05	*yheO*
gene-b2966	0.557058584	0.003207387	*yqgA*
gene-b0736	-0.557031643	0.001864018	*ybgC*
gene-b2149	0.556739854	0.001088508	*mglA*
gene-b1982	-0.556671988	1.30E-05	*amn*
gene-b3897	-0.555544294	0.173284757	*frvR*
gene-b3620	-0.555541554	4.33E-05	*waaF*
gene-b3333	-0.555322941	0.190891705	*gspL*
gene-b0929	-0.555187341	0.005126065	*ompF*
gene-b0423	-0.554942239	0.000468816	*thiI*
gene-b3671	0.554683966	6.11E-05	*ilvB*
gene-b4467	0.552925907	0.000590077	*glcF*
gene-b2558	-0.552860338	0.026906268	*mltF*
gene-b2025	-0.552829296	0.000667498	*hisF*
gene-b0896	-0.552725983	0.018726625	*dmsC*
gene-b0391	0.552497528	0.000332318	*ppnP*
gene-b3468	0.552350547	1.67E-05	*yhhN*
gene-b4313	-0.552318606	0.161669595	*fimE*
gene-b2105	-0.552309715	0.046132131	*rcnR*
gene-b1858	-0.552092564	0.000116637	*znuC*
gene-b3383	0.551372522	0.000684441	*yhfZ*
gene-b3173	0.550648612	0.099893734	*yhbX*
gene-b4249	-0.550496413	0.086867884	*bdcA*
gene-b0345	-0.550421934	0.000745174	*lacI*
gene-b3227	-0.549817559	0.207706684	*dcuD*
gene-b2567	0.548936385	7.07E-05	*rnc*
gene-b2473	0.548745759	0.000139971	*ypfH*
gene-b0947	-0.548453133	3.29E-05	*ycbX*
gene-b3386	-0.548433306	7.54E-06	*rpe*
gene-b4347	0.54793999	0.440187266	*symE*
gene-b1099	-0.547938108	0.000839925	*holB*
gene-b3665	0.547701498	0.001922805	*adeD*
gene-b0639	-0.547556275	0.02217667	*nadD*
gene-b3703	0.547413953	0.050097428	*rpmH*
gene-b0192	0.547402781	9.79E-06	*nlpE*
gene-b3235	-0.547190117	0.000844966	*degS*
gene-b1254	0.547081195	9.21E-05	*yciB*
gene-b1836	0.546718967	6.46E-05	*yebV*
gene-b1775	0.546349888	0.068377717	*ydjK*
gene-b2916	-0.546075447	9.66E-05	*argP*
gene-b2065	-0.54585604	4.09E-05	*dcd*
gene-b4293	-0.545696299	0.00370179	*fecI*
gene-b3180	0.545006034	0.000502772	*yhbY*
gene-b4161	-0.544868725	3.76E-05	*rsgA*
gene-b2835	0.544583016	0.000407407	*lplT*
gene-b0917	-0.543651611	0.0499439	*ycaR*
gene-b3905	-0.543450164	0.012629848	*rhaS*
gene-b0722	-0.54233805	0.000154838	*sdhD*
gene-b3877	0.542258868	0.09753304	*yihP*
gene-b0129	-0.541799823	0.000720904	*yadI*
gene-b1077	0.541223858	0.226066226	*flgF*
gene-b2367	0.540717683	0.166446151	*emrY*
gene-b1908	-0.540051108	0.000120738	*yecA*

gene-b0318	0.540033143	0.101062249	*yahD*
gene-b3529	0.538997713	2.62E-05	*pdeK*
gene-b3634	-0.538448702	0.00052539	*coaD*
gene-b2026	-0.538393599	0.000289619	*hisI*
gene-b2627	0.537850571	5.66E-05	*abpB*
gene-b3188	0.537065507	0.066144883	*sfsB*
gene-b2993	-0.536583786	0.014644748	*hybD*
gene-b1669	-0.536503551	0.132933582	*ydhT*
gene-b4173	-0.536472897	2.36E-05	*hflX*
gene-b0336	0.536122675	0.141883956	*codB*
gene-b1023	-0.535799757	0.029901866	*pgaB*
gene-b1006	0.535427205	0.004460091	*rutG*
gene-b3288	0.535352496	8.29E-05	*fmt*
gene-b1078	-0.535214553	0.141586711	*flgG*
gene-b1710	0.534953258	3.45E-05	*btuE*
gene-b0848	0.534550491	0.107006317	*ybjM*
gene-b0200	-0.533854581	5.48E-05	*gmhB*
gene-b0291	0.53377856	0.022540323	*ecpC*
gene-b3884	-0.533350493	0.000289749	*csqR*
gene-b3338	0.533348006	0.000133664	*chiA*
gene-b2164	0.532814725	0.003769457	*psuT*
gene-b3355	-0.532709046	2.54E-05	*prkB*
gene-b2203	-0.532518173	0.098893463	*napB*
gene-b2159	0.53243062	0.000117068	*nfo*
gene-b0888	-0.532364155	0.000314196	*trxB*
gene-b2560	0.532348156	9.69E-05	*pgpC*
gene-b3075	-0.532105355	0.003651799	*ebgR*
gene-b1141	0.531163091	0.323813105	*xisE*
gene-b0772	-0.530969347	0.00013171	*ybhC*
gene-b1617	0.530925274	0.000101347	*uidA*
gene-b0962	0.530867967	5.44E-05	*helD*
gene-b3667	-0.53078641	0.061666341	*uhpC*
gene-b3699	-0.530298566	8.95E-05	*gyrB*
gene-b0210	0.530277822	0.001784416	*yafE*
gene-b0404	-0.53024833	0.006818103	*acpH*
gene-b1033	-0.529530369	1.35E-05	*ghrA*
gene-b0606	0.529498429	1.68E-05	*ahpF*
gene-b1248	0.529497947	0.001683432	*yciU*
gene-b0106	-0.529483639	0.128743693	*hofC*
gene-b4267	0.529221366	0.004715239	*idnD*
gene-b3747	0.528694096	3.23E-05	*kup*
gene-b1238	0.528523682	0.013343822	*tdk*
gene-b3738	-0.528349809	1.84E-05	*atpB*
gene-b3249	0.527908319	0.007214385	*mreD*
gene-b2414	-0.527628281	0.017978117	*cysK*
gene-b3222	0.527474688	0.000135205	*nanK*
gene-b0270	0.527468231	0.001575362	*yagG*
gene-b1277	-0.527320366	0.000485	*ribA*
gene-b1693	-0.527265235	0.000156367	*aroD*
gene-b1384	-0.526868595	0.004247182	*feaR*
gene-b4478	-0.526803399	0.043419985	*dgoD*
gene-b2104	-0.526563249	0.023167905	*thiM*
gene-b1843	-0.526469812	0.003111129	*yobB*
gene-b2280	0.526392583	0.00013993	*nuoJ*
gene-b0400	0.526276363	0.000292365	*phoR*
gene-b1694	0.526146777	0.285537739	*ydiF*
gene-b0505	-0.525474416	0.104258235	*allA*
gene-b3465	-0.525426377	0.000239027	*rsmD*
gene-b0810	-0.52510887	9.73E-05	*glnP*
gene-b1991	-0.524986485	0.000636195	*cobT*
gene-b4006	0.523842408	0.001470957	*purH*
gene-b4210	-0.523786001	0.050938352	*ytfF*
gene-b4566	-0.523234866	0.517483756	*topAI*
gene-b1932	0.522911708	0.07693117	*yedL*
gene-b2267	0.522797352	0.00011003	*elaA*
gene-b1826	0.522352853	0.190311679	*mgrB*

gene-b1233	-0.521413232	0.014790276	*ychJ*
gene-b3187	-0.521407575	6.12E-05	*ispB*
gene-b2897	-0.521264535	0.000257925	*sdhE*
gene-b3608	0.5209253	3.75E-05	*gpsA*
gene-b2308	0.520288726	0.014017565	*hisQ*
gene-b2618	-0.520138643	0.043088419	*yfjF*
gene-b2277	0.520108726	3.99E-05	*nuoM*
gene-b0578	-0.519874183	0.000305307	*nfsB*
gene-b1429	0.519778977	0.000435147	*tehA*
gene-b2415	0.519313253	2.55E-05	*ptsH*
gene-b1887	0.519208137	0.168615357	*cheW*
gene-b1882	0.518487935	0.243285088	*cheY*
gene-b3199	0.51807025	7.36E-05	*lptC*
gene-b2190	0.517630773	0.000945411	*yejO*
gene-b1055	-0.517430734	0.001834327	*trhO*
gene-b1149	0.517139383	0.09369844	*ymfN*
gene-b4046	-0.516908112	0.000823519	*zur*
gene-b3076	0.516874602	0.004235722	*ebgA*
gene-b4330	-0.516872696	0.165344873	*yjiH*
gene-b2023	-0.515616405	0.002440149	*hisH*
gene-b4142	-0.515164335	0.000112686	*groS*
gene-b0306	-0.515148323	0.016683132	*ykgE*
gene-b1413	0.515107637	3.39E-05	*hrpA*
gene-b2873	-0.515096065	0.210498138	*hyuA*
gene-b2995	-0.514802582	0.031073081	*hybB*
gene-b2306	0.514074075	0.015603098	*hisP*
gene-b3026	-0.513336191	0.000380533	*qseC*
gene-b2781	0.513332511	0.000496933	*mazG*
gene-b0413	-0.51320968	0.00118555	*nrdR*
gene-b0815	0.51223645	0.007539854	*opgE*
gene-b0741	0.512124401	0.000372351	*pal*
gene-b3070	-0.511978472	0.012882498	*nfeF*
gene-b2686	0.511863973	0.002334286	*emrB*
gene-b3865	-0.51153215	0.000292995	*yihA*
gene-b3886	0.511458246	0.000285569	*yihY*
gene-b4180	-0.511437115	0.000414257	*rlmB*
gene-b0314	0.511271119	0.001797578	*betT*
gene-b1778	-0.511149132	6.87E-05	*msrB*
gene-b1432	-0.511068485	0.000844123	*insQ*
gene-b2078	0.510896702	0.070224208	*baeS*
gene-b0368	0.510846039	0.077321801	*tauD*
gene-b3637	0.509691108	0.000899128	*rpmB*
gene-b3980	-0.509649466	0.000483246	*tufB*
gene-b2872	-0.509634239	0.119631316	*ygeY*
gene-b3786	-0.509601871	0.000453651	*wecB*
gene-b0055	-0.509279224	0.000300729	*djlA*
gene-b4460	0.508084292	3.26E-05	*araH*
gene-b0757	0.507413601	0.002822461	*galK*
gene-b2460	0.505242151	0.065803463	*eutQ*
gene-b1791	-0.505119023	0.071981694	*nimT*
gene-b1769	0.504840798	0.251111427	*ydjE*
gene-b2729	-0.504790964	0.026062975	*hypD*
gene-b0679	0.504624186	6.45E-05	*nagE*
gene-b3717	0.504607907	0.053636816	*cbrC*
gene-b3396	-0.50387171	7.17E-05	*mrcA*
gene-b2205	-0.503856771	0.072770969	*napG*
gene-b0981	0.503746007	0.000170731	*etk*
gene-b3869	-0.503585519	0.002195096	*glnL*
gene-b2512	0.503522653	8.37E-05	*bamB*
gene-b2256	0.503065195	0.079692298	*arnD*
gene-b3611	0.502945135	0.000335264	*yibN*
gene-b4169	0.502142974	0.000592742	*amiB*
gene-b1902	0.501966044	0.00024018	*ftnB*
gene-b1686	0.501860781	0.004825351	*menI*
gene-b2807	0.501554632	0.022929997	*ygdD*
gene-b1252	-0.501468971	0.001134157	*tonB*

gene-b0406	-0.501287929	0.000272248	*tgt*
gene-b4349	-0.501228091	0.001470381	*hsdM*
gene-b2541	0.500789553	0.028561401	*hcaB*
gene-b3156	-0.500780852	0.000314404	*yhbS*
gene-b0356	0.500027999	0.000645493	*frmA*
gene-b2086	0.499945592	0.000403979	*yegS*
gene-b0711	0.499106003	0.000291503	*pxpB*
gene-b1939	-0.498981013	0.154479066	*fliG*
gene-b4387	-0.498721591	0.004185589	*ytjB*
gene-b2111	-0.498567817	0.039419398	*yehD*
gene-b1255	-0.498507115	0.005525377	*yciC*
gene-b2132	-0.498160085	7.44E-05	*bglX*
gene-b0133	-0.498022516	5.88E-05	*panC*
gene-b0367	0.497217304	0.365273577	*tauC*
gene-b1274	0.496847955	0.000166745	*topA*
gene-b3342	-0.496550452	8.57E-05	*rpsL*
gene-b1289	0.495719915	0.051263643	*ycjD*
gene-b2805	-0.495304326	0.000115482	*fucR*
gene-b2764	-0.495060544	0.072413465	*cysJ*
gene-b0900	-0.494783365	0.049679626	*ycaN*
gene-b3651	-0.494687765	0.005548778	*trmH*
gene-b1674	-0.494434915	0.448892755	*ydhY*
gene-b3709	0.493201153	0.000167428	*tnaB*
gene-b3842	-0.492825005	0.014820519	*rfaH*
gene-b1180	-0.492776366	0.001052638	*ycgM*
gene-b4428	0.492687484	0.128028121	*hokB*
gene-b1884	-0.492664185	0.373801574	*cheR*
gene-b1191	-0.491844296	0.00043498	*cvrA*
gene-b2366	-0.489405178	0.001237831	*dsdA*
gene-b2625	0.488659309	0.062525185	*yfjI*
gene-b2497	0.488390988	0.016519649	*uraA*
gene-b2839	-0.488095534	0.055164622	*lysR*
gene-b1697	0.487710349	0.294892589	*ydiQ*
gene-b2751	0.487497283	0.066207812	*cysN*
gene-b3590	-0.487425419	0.000593888	*selB*
gene-b4372	-0.486762874	0.020777753	*holD*
gene-b4175	-0.486658909	8.23E-05	*hflC*
gene-b1323	0.486318583	8.17E-05	*tyrR*
gene-b4475	0.486271431	0.038821205	*rtcA*
gene-b1312	0.485720221	0.323291598	*ycjP*
gene-b0131	-0.485193048	0.002503206	*panD*
gene-b0490	-0.484929555	0.022841007	*fetA*
gene-b0931	-0.484825454	0.00019147	*pncB*
gene-b2457	0.483964756	0.042409211	*eutM*
gene-b2150	0.483911092	0.005303931	*mglB*
gene-b0686	-0.48329404	0.002853588	*ybfF*
gene-b3080	0.482791422	0.072694996	*ygjK*
gene-b2521	0.482724492	0.001004112	*sseA*
gene-b4222	0.481616952	0.000404962	*ytfP*
gene-b0252	0.481566155	0.423792501	*yafZ*
gene-b1924	-0.48139396	0.044144506	*fliD*
gene-b4297	0.481182077	0.00388489	*yjhG*
gene-b1742	-0.480981419	0.249058775	*ves*
gene-b0213	-0.480861807	0.006711109	*yafS*
gene-b1292	0.480682788	0.014126226	*sapC*
gene-b2674	0.479876015	0.030128513	*nrdI*
gene-b3549	0.479463202	0.000581027	*tag*
gene-b2474	0.47914722	0.000841609	*tmcA*
gene-b1857	-0.477824331	0.001362214	*znuA*
gene-b2279	0.477538716	0.006608571	*nuoK*
gene-b2230	-0.477436003	0.049224259	*yfaA*
gene-b0949	0.476498316	0.000297704	*uup*
gene-b0521	0.476094779	0.180251494	*ybcF*
gene-b2563	-0.475793322	0.025985007	*acpS*
gene-b2027	-0.474308246	0.000243179	*wzzB*
gene-b1604	-0.474053383	0.000155065	*ydgH*

gene-b2989	0.473617881	0.000149754	*yghU*
gene-b3240	-0.473510331	0.038302839	*aaeB*
gene-b0081	-0.472318009	0.001818768	*mraZ*
gene-b4492	0.471725816	0.000314599	*ydbA*
gene-b3133	0.471630142	0.096925794	*agaV*
gene-b2232	-0.471152076	0.00274836	*ubiG*
gene-b0516	-0.470345576	0.103906221	*allC*
gene-b4354	0.470116588	0.042176897	*btsT*
gene-b1589	0.470114359	0.086951776	*ynfG*
gene-b1557	-0.47011283	0.176656585	*cspB*
gene-b3184	0.469985444	0.005566698	*yhbE*
gene-b1407	0.469974319	0.119192178	*ydbD*
gene-b2021	-0.469855142	0.000658576	*hisC*
gene-b2317	-0.469204177	0.000722844	*dedA*
gene-b2281	0.468692489	0.000874216	*nuoI*
gene-b3439	0.468509262	0.000144761	*yhhW*
gene-b3831	0.467992357	7.33E-05	*udp*
gene-b2711	0.467873578	0.13984283	*norW*
gene-b1587	0.467319014	0.051823134	*ynfE*
gene-b0969	-0.467112186	0.04289278	*tusE*
gene-b0121	-0.466323923	0.000321391	*speE*
gene-b2616	0.466089868	0.00082413	*recN*
gene-b0363	0.465731004	0.219019451	*yaiP*
gene-b2381	-0.465725475	0.004516696	*pyrR*
gene-b1605	-0.465069396	0.017940269	*ydgI*
gene-b3072	0.464872737	0.007168109	*aer*
gene-b3177	-0.464522023	0.001251379	*folP*
gene-b0582	-0.464427994	0.003294603	*insL2*
gene-b2394	-0.464427858	0.003296948	*insL3*
gene-b0016	-0.464427672	0.003300183	*insL1*
gene-b2345	0.463670496	0.057595498	*yfdF*
gene-b4065	-0.463327612	0.000677227	*yjcE*
gene-b3562	-0.463264609	0.286335568	*yiaA*
gene-b2491	0.463178358	0.069922214	*hyfR*
gene-b1928	-0.463037051	0.000429765	*yedD*
gene-b4092	0.462673629	0.047186639	*phnP*
gene-b0432	-0.461695199	0.001528742	*cyoA*
gene-b0603	-0.46150434	0.212902696	*ybdO*
gene-b2825	-0.461167151	0.025643664	*ppdB*
gene-b0061	-0.460780119	0.000289525	*araD*
gene-b2511	0.460691273	0.000174846	*der*
gene-b3398	-0.459997118	0.000408622	*igaA*
gene-b2942	-0.459980886	0.003264359	*metK*
gene-b4174	-0.459818232	0.000135369	*hflK*
gene-b3037	-0.459770998	0.001792699	*ygiB*
gene-b3753	-0.459764464	0.015808823	*rbsR*
gene-b0967	-0.459743316	0.002850328	*rlmI*
gene-b4238	0.459414296	0.015579392	*nrdD*
gene-b2114	-0.458890832	0.000752423	*metG*
gene-b0029	-0.458646525	0.000682821	*ispH*
gene-b0994	-0.458448568	0.026216297	*torT*
gene-b1206	-0.458259982	0.018287907	*dauA*
gene-b4551	0.457831581	0.158991201	*yheV*
gene-b4129	0.45760593	0.001637604	*lysU*
gene-b1243	0.457035472	0.00039018	*oppA*
gene-b3090	-0.456736353	0.122320797	*ygjV*
gene-b2424	-0.456416649	0.071270636	*cysU*
gene-b0232	0.456297397	0.160650176	*yafN*
gene-b3183	0.456264744	0.001568982	*obgE*
gene-b0427	-0.455829202	0.013913255	*yajR*
gene-b2259	-0.455719674	0.011098632	*pmrD*
gene-b2270	0.455467241	0.271819133	*yfbK*
gene-b2186	-0.455043406	0.000336853	*yejK*
gene-b3867	-0.454970143	0.000313803	*hemN*
gene-b2529	0.454502732	0.002461941	*iscU*
gene-b1462	0.454253481	0.004733135	*yddH*

gene-b2870	-0.45420273	0.169264797	*ygeW*
gene-b0093	0.454174223	0.000155378	*ftsQ*
gene-b0556	0.454154694	0.326364405	*rzpD*
gene-b3653	0.454135164	0.001765498	*gltS*
gene-b4260	0.452360695	0.000295663	*pepA*
gene-b0877	-0.451161652	0.00330628	*ybjX*
gene-b2900	0.450686671	0.005236261	*yqfB*
gene-b0437	0.450213956	0.001105848	*clpP*
gene-b1143	0.449866813	0.100039263	*ymfI*
gene-b4078	-0.449799174	0.001001179	*yjcO*
gene-b2429	0.449695452	0.01431094	*murP*
gene-b0268	0.448572405	0.001309901	*yagE*
gene-b4192	0.448312343	0.016373652	*ulaG*
gene-b4295	-0.448225296	0.000423251	*yjhU*
gene-b4351	-0.448194123	0.003593331	*mrr*
gene-b2921	-0.448052727	0.322107072	*ygfI*
gene-b0825	0.44799692	0.009256822	*fsaA*
gene-b1246	0.447487543	0.000190638	*oppD*
gene-b3438	0.447353301	0.002837118	*gntR*
gene-b1253	0.447216857	0.118089404	*yciA*
gene-b1322	-0.446895457	0.001793635	*ycjF*
gene-b1743	-0.446831227	0.000902467	*spy*
gene-b4385	0.446374305	0.00416342	*yjjJ*
gene-b0650	-0.445877678	0.151438154	*hscC*
gene-b2898	-0.445487831	0.000201142	*ygfZ*
gene-b0350	-0.44535685	0.015781254	*mhpD*
gene-b1663	0.444913603	0.000357073	*mdtK*
gene-b3908	-0.444287045	0.002977989	*sodA*
gene-b3462	0.44385151	0.000412246	*ftsX*
gene-b1822	-0.443389984	0.026038325	*rlmA*
gene-b2584	0.443297319	0.008183978	*patZ*
gene-b3621	-0.44307818	0.004403356	*waaC*
gene-b1832	-0.44297172	0.00268737	*msrC*
gene-b0448	-0.44295548	0.015125861	*mdlA*
gene-b1314	0.442906183	0.21211942	*ycjR*
gene-b2296	0.442831558	0.001156593	*ackA*
gene-b2407	-0.442500473	0.20014175	*xapA*
gene-b0071	0.442271013	0.000580511	*leuD*
gene-b1213	-0.441737086	0.141735714	*ychQ*
gene-b4195	0.441569849	0.1749221	*ulaC*
gene-b3375	-0.441191215	0.013541363	*frlR*
gene-b3402	0.440674714	0.000670273	*yhgE*
gene-b0289	-0.440400185	0.093549341	*ecpE*
gene-b2409	0.440153328	0.051854815	*yfeR*
gene-b1175	-0.440145329	0.000540739	*minD*
gene-b1454	0.439978186	0.016786869	*yncG*
gene-b1869	0.439823727	0.005704158	*yecN*
gene-b0776	-0.439763398	0.035531677	*bioF*
gene-b1632	-0.43965649	0.034999046	*rsxE*
gene-b4156	-0.439586313	0.007356274	*yjeM*
gene-b3107	-0.439330838	0.27914632	*yhaL*
gene-b0752	0.439284077	0.001465551	*zitB*
gene-b0271	0.439209452	0.003669506	*yagH*
gene-b2707	0.439143718	0.015410948	*srlR*
gene-b4367	-0.439022615	0.00706166	*fhuF*
gene-b0353	0.439000407	0.015977253	*mhpT*
gene-b0038	0.438651765	0.181612786	*caiB*
gene-b2327	-0.438531891	0.002486819	*yfcA*
gene-b3684	-0.437897939	0.404005627	*yidP*
gene-b0228	-0.437617972	0.005778286	*rayT*
gene-b0713	0.437391446	0.001251811	*pxpA*
gene-b0720	-0.437378371	0.000726499	*gltA*
gene-b0424	0.436914267	0.014415867	*yajL*
gene-b4004	-0.436647497	0.003109385	*zraR*
gene-b0102	-0.436479976	0.003863048	*zapD*
gene-b0734	0.4362371	0.025868387	*cydB*

gene-b3912	-0.435874682	0.000599773	*cpxR*
gene-b0154	-0.435319194	0.00071706	*hemL*
gene-b3993	0.433356005	0.353193016	*thiE*
gene-b0923	-0.433070001	0.002076612	*mukE*
gene-b2471	-0.432997684	0.026505975	*yffB*
gene-b1659	0.432529126	0.007920081	*punR*
gene-b2965	-0.432018835	0.004739578	*speC*
gene-b2343	0.431955635	0.012598223	*yfcZ*
gene-b0893	-0.431860401	0.000566635	*serS*
gene-b2828	-0.431815843	0.010938474	*lgt*
gene-b4080	-0.431692442	0.311220418	*mdtP*
gene-b0433	-0.431186312	0.007413358	*ampG*
gene-b3790	-0.430610377	0.067915596	*rffC*
gene-b1766	-0.430504732	0.000445064	*sppA*
gene-b2676	-0.430042665	0.025593234	*nrdF*
gene-b0570	-0.429417663	0.039043689	*cusS*
gene-b0835	-0.428373639	0.00601166	*rimO*
gene-b3929	0.426900951	0.003027074	*rraA*
gene-b3951	-0.426868886	0.01018715	*pflD*
gene-b3469	-0.426694705	0.001270263	*zntA*
gene-b2437	0.426422756	0.097385479	*eutR*
gene-b3666	-0.426186088	0.071993404	*uhpT*
gene-b2514	-0.426014553	0.001002993	*hisS*
gene-b2421	-0.425403859	0.057251386	*cysM*
gene-b3531	0.425043737	0.098023626	*bcsZ*
gene-b3882	0.42491097	0.175396564	*yihU*
gene-b0151	-0.42473292	0.005789493	*fhuC*
gene-b0276	0.424516132	0.041885436	*yagJ*
gene-b3370	0.424413374	0.502612721	*frlA*
gene-b4298	0.424283111	0.058504606	*yjhH*
gene-b0742	0.423887041	0.002159411	*cpoB*
gene-b2667	-0.423884227	0.48290762	*ygaV*
gene-b2610	-0.423838307	0.001282318	*ffh*
gene-b2577	-0.423696565	0.021507052	*yfiE*
gene-b1174	-0.423172713	0.012966951	*minE*
gene-b2775	0.423087305	0.147307514	*yqcE*
gene-b3676	0.422892466	0.043943759	*yidH*
gene-b4262	0.422659309	0.000555947	*lptG*
gene-b1631	-0.422581094	0.039611838	*rsxG*
gene-b0191	0.422341851	0.031473118	*arfB*
gene-b1190	0.422179897	0.010429788	*dadX*
gene-b2878	-0.42202211	0.336659918	*ygfK*
gene-b4332	0.421783836	0.1160387	*yjiJ*
gene-b0936	-0.421476309	0.378236241	*ssuA*
gene-b0483	0.421398324	0.012023041	*ybaQ*
gene-b1875	-0.421395463	0.040869938	*yecM*
gene-b0066	-0.421232552	0.201066524	*thiQ*
gene-b2572	-0.420774644	0.000370337	*rseA*
gene-b4751	-0.420064569	0.624253498	*yoaL*
gene-b3219	0.420049833	0.256406964	*yhcF*
gene-b0628	-0.419779259	0.001177986	*lipA*
gene-b3176	-0.419624389	0.000804944	*glmM*
gene-b0496	-0.4194564	0.002270359	*ybbP*
gene-b2527	-0.419158271	0.038952581	*hscB*
gene-b1379	-0.418822434	0.006717066	*hslJ*
gene-b0674	-0.418517962	0.001951805	*asnB*
gene-b2487	0.41850243	0.058460389	*hyfG*
gene-b1267	0.418266474	0.001105001	*yciO*
gene-b0454	-0.418208523	0.117667245	*ybaZ*
gene-b2728	-0.418163407	0.335232474	*hypC*
gene-b3585	-0.417861251	0.023910885	*yiaU*
gene-b1142	0.416950735	0.042380034	*ymfH*
gene-b0506	-0.41602214	0.00115269	*allR*
gene-b0756	0.415638513	0.006154576	*galM*
gene-b0155	0.414645681	0.01173785	*clcA*
gene-b0117	0.413747764	0.202403073	*yacH*

gene-b0738	-0.413656511	0.029441108	*tolR*
gene-b3770	-0.413584916	0.000959653	*ilvE*
gene-b0568	0.413079671	0.011932352	*nfrA*
gene-b1162	-0.412964627	0.031363413	*bluR*
gene-b3949	-0.412721471	0.088381847	*frwC*
gene-b1337	-0.412576597	0.062173844	*abgB*
gene-b0028	-0.412049867	0.008989763	*fkpB*
gene-b4088	-0.412028805	0.004077764	*alsB*
gene-b3846	-0.411836376	0.040282525	*fadB*
gene-b3788	-0.411368855	0.00800885	*rffG*
gene-b2283	0.411253666	0.00148835	*nuoG*
gene-b1925	0.4108723	0.266736577	*fliS*
gene-b1286	-0.410848098	0.00654048	*rnb*
gene-b1799	-0.410753609	0.007187106	*dmlR*
gene-b4041	-0.410743144	0.001354171	*plsB*
gene-b2842	0.410485514	0.002780023	*kduD*
gene-b4595	0.410299339	0.118819412	*yciY*
gene-b2632	0.410253029	0.169098867	*yfjP*
gene-b2716	0.410179912	0.006867367	*ascB*
gene-b0222	-0.410136773	0.006805125	*gmhA*
gene-b0253	0.409466449	0.040780413	*ykfA*
gene-b0585	-0.409292962	0.078271345	*fes*
gene-b4569	0.409131093	0.405410877	*yhcE*
gene-b4144	-0.408472388	0.001317291	*yjeI*
gene-b3743	0.40846881	0.065181269	*asnC*
gene-b4300	0.408158856	0.069815643	*sgcR*
gene-b4539	-0.408124841	0.149213461	*yoeB*
gene-b0607	0.407874633	0.002819605	*uspG*
gene-b4136	-0.407528354	0.00711051	*dsbD*
gene-b4371	-0.406781735	0.00204058	*rsmC*
gene-b2559	-0.406573203	0.033618616	*tadA*
gene-b1096	-0.406454952	0.008351784	*pabC*
gene-b2880	-0.406198512	0.372267726	*ygfM*
gene-b0027	-0.406162428	0.014007177	*lspA*
gene-b3754	0.405412715	0.003020641	*hsrA*
gene-b1145	0.405372532	0.011238753	*ymfK*
gene-b1076	-0.404266352	0.255369603	*flgE*
gene-b0393	-0.404202397	0.017598661	*rdgC*
gene-b1283	-0.404199717	0.002387385	*osmB*
gene-b4258	0.403905639	0.000795613	*valS*
gene-b2549	0.403669034	0.032553084	*yphG*
gene-b1097	-0.403424744	0.010213582	*mltG*
gene-b0733	0.403051515	0.015945664	*cydA*
gene-b3991	0.402761935	0.125622579	*thiG*
gene-b0238	-0.401369578	0.010864213	*gpt*
gene-b0125	-0.400029255	0.003521912	*hpt*
gene-b2488	0.399918141	0.500805567	*hyfH*
gene-b0470	-0.399038011	0.020778394	*dnaX*
gene-b0828	-0.399033756	0.003013798	*iaaA*
gene-b2561	0.39838082	0.036048468	*yfhH*
gene-b3132	0.398342504	0.119290316	*kbaZ*
gene-b0737	-0.398181688	0.015964591	*tolQ*
gene-b2951	-0.398166944	0.032936445	*yggS*
gene-b1603	0.397781645	0.000829697	*pntA*
gene-b2902	-0.39759719	0.026508869	*ygfF*
gene-b3673	0.397292979	0.008612626	*emrD*
gene-b3942	-0.39725577	0.003290057	*katG*
gene-b1969	-0.397066501	0.04943304	*hprR*
gene-b3561	-0.395968021	0.098776742	*wecH*
gene-b1316	-0.395803204	0.056198243	*ycjT*
gene-b1703	-0.394982038	0.008554777	*ppsR*
gene-b2701	0.394912462	0.013119802	*mltB*
gene-b4076	0.394129053	0.408641667	*nrfG*
gene-b3636	0.393536704	0.010511264	*rpmG*
gene-b2438	0.393504667	0.047024225	*eutK*
gene-b3579	0.393413689	0.245226772	*yiaO*

gene-b0447	-0.393327364	0.057993479	*decR*
gene-b2879	-0.392917008	0.480314268	*ssnA*
gene-b3051	-0.392249987	0.055893082	*yqiK*
gene-b1805	0.392136126	0.06138068	*fadD*
gene-b1593	-0.392064489	0.019165756	*bidA*
gene-b2351	-0.3920494	0.013589004	*yfdH*
gene-b4361	-0.391925764	0.015359391	*dnaC*
gene-b0112	0.391652243	0.003409029	*aroP*
gene-b2595	-0.391558724	0.003272188	*bamD*
gene-b2157	0.391146502	0.002707384	*yeiE*
gene-b3956	-0.390813516	0.002450969	*ppc*
gene-b4266	0.390676436	0.045360699	*idnO*
gene-b3783	-0.390630877	0.002520138	*rho*
gene-b0233	-0.390342745	0.361667146	*yafO*
gene-b2067	0.390161412	0.003122478	*dgcE*
gene-b3883	0.389903154	0.128157716	*yihV*
gene-b1236	-0.389699591	0.005574023	*galU*
gene-b2070	0.389605157	0.065510324	*yegI*
gene-b3388	0.389141536	0.001284489	*damX*
gene-b2569	-0.389049381	0.001431751	*lepA*
gene-b4473	-0.388926776	0.020717118	*smf*
gene-b4296	0.388327437	0.2941627	*yjhF*
gene-b2199	-0.387838322	0.044449123	*ccmC*
gene-b0944	-0.3876542	0.170747112	*ycbF*
gene-b4055	-0.386978216	0.004008418	*aphA*
gene-b1776	0.386783265	0.095259298	*ydjL*
gene-b4108	0.386453826	0.020264372	*yjdM*
gene-b2936	0.386068197	0.002065836	*loiP*
gene-b2918	0.385980139	0.405579788	*argK*
gene-b3985	-0.385920416	0.00187835	*rplJ*
gene-b0354	-0.385353763	0.011537396	*yaiL*
gene-b0676	-0.385298259	0.007129658	*nagC*
gene-b2756	0.385244554	0.097494656	*casE*
gene-b3379	0.385022895	0.475482558	*php*
gene-b0243	0.384976266	0.003483224	*proA*
gene-b1247	0.384535047	0.001562568	*oppF*
gene-b4691	0.384244527	0.337972876	*sroH*
gene-b0681	-0.383818419	0.389805227	*chiP*
gene-b3844	-0.383803507	0.003991065	*fre*
gene-b3385	-0.382917871	0.002770607	*gph*
gene-b4198	0.382732083	0.143981658	*ulaF*
gene-b3826	-0.38226786	0.010060181	*yigL*
gene-b2158	0.382175207	0.004363787	*yeiH*
gene-b3302	-0.381686755	0.024380029	*rpmD*
gene-b2836	-0.380097512	0.002595322	*aas*
gene-b2901	-0.379535546	0.001661445	*bglA*
gene-b1585	0.3791344	0.022580996	*ynfC*
gene-b0032	0.37904493	0.206124648	*carA*
gene-b2385	0.378475373	0.230902	*ypdF*
gene-b0440	0.378203532	0.004844398	*hupB*
gene-b0922	-0.377582025	0.003506418	*mukF*
gene-b0804	0.377316611	0.229451567	*ybiX*
gene-b3787	-0.377003167	0.004625872	*wecC*
gene-b0462	0.376380233	0.00107584	*acrB*
gene-b0488	0.376218764	0.02062856	*ybbJ*
gene-b1900	0.376001898	0.006831798	*araG*
gene-b1242	0.375763938	0.108355751	*ychE*
gene-b1315	-0.375636898	0.257964514	*ycjS*
gene-b0799	-0.375603455	0.042262381	*dinG*
gene-b1716	0.375479939	0.005326279	*rplT*
gene-b3819	-0.375093188	0.260473724	*rarD*
gene-b3921	0.374328671	0.125335804	*yiiR*
gene-b3146	-0.373599899	0.02759731	*rsmI*
gene-b0831	-0.373039867	0.053182235	*gsiC*
gene-b0411	-0.372670341	0.008463868	*tsx*
gene-b4414	-0.37191285	0.323972217	*tff*

gene-b0820	0.371629998	0.008637266	*ybiT*
gene-b3341	-0.3715128	0.002476863	*rpsG*
gene-b1749	-0.371422593	0.008058157	*xthA*
gene-b3834	-0.370538809	0.003994387	*ubiJ*
gene-b2284	0.370450869	0.007625039	*nuoF*
gene-b0293	0.370187605	0.14538801	*ecpA*
gene-b2503	-0.368710181	0.206531466	*pdeF*
gene-b2126	-0.368678985	0.009217777	*btsS*
gene-b0240	0.36836145	0.009228663	*crl*
gene-b1050	0.368225518	0.539406422	*yceK*
gene-b1729	0.367427359	0.094722455	*tcyP*
gene-b0473	-0.366434576	0.002027375	*htpG*
gene-b0800	0.36575951	0.013486999	*ybiB*
gene-b3751	0.364960363	0.003328741	*rbsB*
gene-b2720	0.363781464	0.513755135	*hycF*
gene-b3784	-0.363637648	0.005727257	*rfe*
gene-b3498	0.363608615	0.004254699	*prlC*
gene-b1415	0.362856821	0.044339103	*aldA*
gene-b3371	0.362015044	0.096450882	*frlB*
gene-b3609	0.361969021	0.011316884	*secB*
gene-b0590	-0.361860392	0.065835834	*fepD*
gene-b1833	0.361730347	0.012818815	*letA*
gene-b1888	0.361290084	0.25040119	*cheA*
gene-b1985	0.361271484	0.01530584	*yeeO*
gene-b1829	-0.360497862	0.003956292	*htpX*
gene-b3785	-0.360434452	0.012547447	*wzzE*
gene-b1525	-0.360188976	0.015531996	*sad*
gene-b2375	0.359352647	0.458261554	*yfdX*
gene-b1757	-0.359068261	0.023001135	*ynjE*
gene-b0074	-0.358386983	0.005993602	*leuA*
gene-b4145	0.358383861	0.271667079	*yjeJ*
gene-b2014	0.358097665	0.077522716	*plaP*
gene-b0023	0.35775321	0.009709008	*rpsT*
gene-b3648	-0.356928944	0.010589027	*gmk*
gene-b3583	0.355658673	0.230755707	*sgbE*
gene-b0677	0.355212313	0.007341864	*nagA*
gene-b1848	0.354411034	0.011507752	*yebG*
gene-b1488	0.354305159	0.274155097	*ddpX*
gene-b3735	-0.35426397	0.004208536	*atpH*
gene-b3440	0.354017306	0.004192558	*yhhX*
gene-b4328	-0.353942236	0.010206184	*iadA*
gene-b3935	-0.35263963	0.01679596	*priA*
gene-b0525	-0.351867326	0.036169578	*ppiB*
gene-b0410	-0.35170597	0.013203142	*yajD*
gene-b2431	-0.351634939	0.003231509	*yfeX*
gene-b1734	-0.35119475	0.130825075	*chbF*
gene-b2096	-0.351033627	0.00290907	*gatY*
gene-b2081	0.350967086	0.018319823	*trhP*
gene-b3298	-0.350954946	0.011224119	*rpsM*
gene-b0409	0.35035169	0.006495526	*secF*
gene-b0943	-0.3503269	0.395739837	*ycbV*
gene-b1872	-0.35028171	0.0558753	*torZ*
gene-b3343	0.350216446	0.169544204	*tusB*
gene-b2100	0.350154771	0.066003655	*yegV*
gene-b0239	-0.350019641	0.003483212	*frsA*
gene-b3094	0.349605638	0.013733377	*exuR*
gene-b0935	-0.349149391	0.227134971	*ssuD*
gene-b3505	-0.348953685	0.005957365	*insH11*
gene-b4711	-0.348953673	0.005953715	*insH21*
gene-b0552	-0.348953325	0.005844811	*insH2*
gene-b0259	-0.348953316	0.005842021	*insH1*
gene-b2030	-0.348953291	0.005834355	*insH7*
gene-b0656	-0.348953285	0.005832368	*insH3*
gene-b1331	-0.348953275	0.005829442	*insH4*
gene-b2192	-0.348953271	0.005828248	*insH8*
gene-b2982	-0.34895327	0.005827762	*insH9*

gene-b3218	-0.348953267	0.005826813	*insH10*
gene-b1114	-0.348167977	0.003704289	*mfd*
gene-b4259	0.347915782	0.028557592	*holC*
gene-b2261	0.347893119	0.012526654	*menC*
gene-b3020	-0.347821506	0.00568703	*ygiS*
gene-b3660	0.347522494	0.079319309	*yicL*
gene-b0351	-0.347485191	0.024045042	*mhpF*
gene-b4440	0.347448741	0.020057551	*ryfA*
gene-b2808	0.347080366	0.03449122	*gcvA*
gene-b4086	0.347065082	0.241548671	*alsC*
gene-b0207	0.346968836	0.039787247	*dkgB*
gene-b0110	-0.346764389	0.030889545	*ampD*
gene-b1434	-0.345793646	0.329410878	*sutR*
gene-b2508	-0.345715225	0.071954003	*guaB*
gene-b0782	-0.345586291	0.019393867	*moaB*
gene-b3210	-0.344833151	0.005275043	*arcB*
gene-b2962	-0.344585269	0.051570593	*yggX*
gene-b0731	0.344226101	0.351069903	*mngA*
gene-b3500	-0.343667635	0.005040715	*gor*
gene-b0954	0.34324592	0.006612194	*fabA*
gene-b4780	0.342974429	0.320117047	*yodE*
gene-b1785	-0.342510729	0.377856896	*cdgI*
gene-b2129	0.342460593	0.019763091	*yehX*
gene-b1761	-0.341337623	0.011186087	*gdhA*
gene-b2710	0.340112518	0.106825344	*norV*
gene-b1421	0.33922332	0.087744004	*trg*
gene-b1386	-0.339179227	0.103699165	*tynA*
gene-b2937	0.339096073	0.005723771	*speB*
gene-b2708	0.338557885	0.045671429	*gutQ*
gene-b3733	-0.338314775	0.010529832	*atpG*
gene-b0377	-0.337612782	0.028785167	*sbmA*
gene-b2722	0.337536228	0.527271035	*hycD*
gene-b3843	-0.337389124	0.00558844	*ubiD*
gene-b2542	0.337226175	0.120885322	*hcaD*
gene-b1381	-0.337122629	0.008667476	*ydbH*
gene-b4218	0.337069922	0.026923034	*paeA*
gene-b3712	-0.337042603	0.028758949	*yieE*
gene-b4265	-0.336803714	0.090439735	*idnT*
gene-b2285	0.336757774	0.006191361	*nuoE*
gene-b0126	-0.336752513	0.052209626	*can*
gene-b2684	-0.336088074	0.083097893	*mprA*
gene-b1036	-0.33605139	0.091339123	*ycdZ*
gene-b4391	0.335259153	0.023577333	*ettA*
gene-b0123	0.334966236	0.026329839	*cueO*
gene-b4456	0.334816509	0.06043333	*glmZ*
gene-b3223	0.333684543	0.032264994	*nanE*
gene-b2299	0.33365639	0.022812622	*yfcD*
gene-b2700	-0.332988711	0.041794789	*pncC*
gene-b3279	-0.332900247	0.02002694	*yrdA*
gene-b0792	0.332608812	0.077349412	*ybhR*
gene-b3413	-0.332178654	0.032810259	*yhgH*
gene-b0234	-0.331281659	0.545514506	*yafP*
gene-b2493	-0.331135998	0.021914488	*yfgO*
gene-b4140	-0.33112007	0.024041948	*fxsA*
gene-b0832	-0.331084697	0.162976252	*gsiD*
gene-b2297	0.330914262	0.005587685	*pta*
gene-b3028	-0.330842293	0.010483113	*mdaB*
gene-b2713	-0.330585885	0.699158723	*hydN*
gene-b0153	-0.330024636	0.036985183	*fhuB*
gene-b2841	0.329778894	0.005167695	*araE*
gene-b0852	-0.329556095	0.018127647	*rimK*
gene-b2410	-0.328573136	0.02410488	*yfeH*
gene-b1600	0.328394693	0.22456252	*mdtJ*
gene-b2623	-0.328384465	0.100923918	*yfjH*
gene-b0209	-0.328240515	0.011175628	*yafD*
gene-b1214	-0.32814802	0.029279333	*ychA*

gene-b2211	-0.328036291	0.040286647	*yojI*
gene-b2427	-0.327832135	0.290659822	*murR*
gene-b3106	-0.327700952	0.182252458	*yhaK*
gene-b1215	-0.327693297	0.014198479	*kdsA*
gene-b0523	0.327579912	0.23939945	*purE*
gene-b2214	-0.32726958	0.091794185	*ftp*
gene-b4021	-0.327211136	0.025315071	*pepE*
gene-b4287	-0.326520405	0.207476266	*fecE*
gene-b1881	0.326358618	0.281287294	*cheZ*
gene-b3201	0.326223339	0.026988713	*lptB*
gene-b2726	0.326099011	0.501815075	*hypA*
gene-b3566	0.325709317	0.036948653	*xylF*
gene-b2293	0.325026924	0.007490213	*hxpA*
gene-b0346	0.324729529	0.104507627	*mhpR*
gene-b0046	0.324648325	0.237213815	*kefF*
gene-b3849	0.324353851	0.023685732	*trkH*
gene-b3647	-0.324231499	0.125414931	*ligB*
gene-b1186	-0.324117213	0.009658287	*nhaB*
gene-b2007	0.322810932	0.06682165	*tmaR*
gene-b1420	0.322685686	0.14616887	*mokB*
gene-b1699	-0.322295425	0.356499222	*ydiS*
gene-b2519	0.322239124	0.135719236	*pbpC*
gene-b2130	0.321546614	0.030758157	*yehY*
gene-b3857	-0.320910005	0.068696601	*mobA*
gene-b3340	-0.320830763	0.008263775	*fusA*
gene-b3642	-0.320558604	0.198232379	*pyrE*
gene-b3052	0.320314321	0.016094486	*hldE*
gene-b1290	0.320077803	0.045042766	*sapF*
gene-b3526	-0.320024202	0.025093813	*kdgK*
gene-b2262	0.319964901	0.020740225	*menB*
gene-b3715	-0.319544841	0.225275066	*yieH*
gene-b0716	0.319282149	0.569017304	*ybgO*
gene-b2482	-0.319115981	0.458024466	*hyfB*
gene-b2735	-0.318739263	0.020973878	*ygbI*
gene-b0103	-0.318630279	0.082168533	*coaE*
gene-b3565	0.318376996	0.029107206	*xylA*
gene-b3035	0.318352975	0.008001526	*tolC*
gene-b3422	0.318116619	0.257895745	*rtcR*
gene-b1866	-0.317974874	0.006477315	*aspS*
gene-b3607	0.317919262	0.016371157	*cysE*
gene-b2827	-0.317583924	0.016857411	*thyA*
gene-b2029	0.317330976	0.064023714	*gnd*
gene-b0181	-0.317315897	0.013613504	*lpxA*
gene-b2894	0.317187783	0.014686072	*xerD*
gene-b2952	-0.317051521	0.060864591	*yggT*
gene-b1411	0.316529213	0.039897224	*ynbD*
gene-b0768	0.315983702	0.323148645	*ybhD*
gene-b0149	0.315771604	0.013924501	*mrcB*
gene-b2992	-0.315485552	0.128135244	*hybE*
gene-b2313	0.315458868	0.095811643	*cvpA*
gene-b3168	0.31518522	0.015916409	*infB*
gene-b1530	-0.315126342	0.419547401	*marR*
gene-b2741	0.314819752	0.020496713	*rpoS*
gene-b1653	0.314783168	0.042361865	*lhr*
gene-b1580	0.314469744	0.071484998	*rspB*
gene-b1374	-0.314253014	0.464452985	*pinR*
gene-b2911	-0.314071849	0.056072814	*ssrS*
gene-b2422	-0.313417425	0.237503953	*cysA*
gene-b1756	0.313315551	0.183484074	*ynjD*
gene-b4294	-0.311194089	0.426146911	*insA7*
gene-b2876	0.311149538	0.294220543	*yqeC*
gene-b1438	-0.310813918	0.17730332	*hicB*
gene-b2770	0.310766975	0.302970035	*ygcR*
gene-b1132	-0.310152681	0.031172211	*hflD*
gene-b3061	0.310129233	0.616645725	*ttdA*
gene-b1809	-0.309902612	0.05996154	*yoaB*

gene-b1831	-0.309720866	0.013238242	*proQ*
gene-b4059	0.309232343	0.01322941	*ssb*
gene-b1597	0.308898699	0.416690443	*asr*
gene-b1419	0.308328829	0.385099229	*ydcA*
gene-b0889	-0.307958144	0.018727188	*lrp*
gene-b0813	0.307815744	0.151122697	*rhtA*
gene-b1129	-0.307321809	0.033664885	*phoQ*
gene-b2012	0.307202992	0.266089547	*tsuB*
gene-b3701	0.306774642	0.011408751	*dnaN*
gene-b2794	-0.306655881	0.02238072	*queF*
gene-b4291	-0.306528251	0.062285697	*fecA*
gene-b3384	-0.305878743	0.016659318	*trpS*
gene-b1662	0.305389962	0.058103831	*ribC*
gene-b3389	-0.305324041	0.00999214	*aroB*
gene-b0903	0.30504214	0.017190594	*pflB*
gene-b1402	-0.304889999	0.08551883	*insD2*
gene-b0361	-0.304889631	0.085555166	*insD1*
gene-b2860	-0.304888094	0.085706966	*insD4*
gene-b1996	-0.304887915	0.085724635	*insD3*
gene-b3045	-0.304886772	0.085837715	*insD5*
gene-b4273	-0.304886558	0.085858862	*insD6*
gene-b3056	0.304717355	0.059103954	*cca*
gene-b4000	0.304570411	0.052722451	*hupA*
gene-b0565	-0.304127028	0.234879032	*ompT*
gene-b1753	-0.303892771	0.104099957	*ynjA*
gene-b2215	0.30349476	0.012818411	*ompC*
gene-b0739	0.302947763	0.018036565	*tolA*
gene-b0014	-0.302496845	0.015524749	*dnaK*
gene-b3054	-0.302449345	0.018305053	*ygiF*
gene-b1453	-0.301045449	0.266444632	*ansP*
gene-b0037	0.300911827	0.37391398	*caiC*
gene-b3983	-0.300761511	0.018949336	*rplK*
gene-b3915	0.300252415	0.02791824	*fieF*
gene-b4289	0.299894516	0.145760012	*fecC*
gene-b4399	-0.299841489	0.037329004	*creC*
gene-b1649	0.299685526	0.135577881	*nemR*
gene-b2268	-0.299401514	0.057846527	*rbn*
gene-b1672	-0.299364612	0.213591748	*ydhW*
gene-b2888	-0.298874843	0.63860957	*uacT*
gene-b3424	-0.298811406	0.058519233	*glpG*
gene-b3457	-0.298708723	0.460637095	*livH*
gene-b1993	-0.298399841	0.117754397	*cobU*
gene-b0649	-0.297516522	0.138931667	*djlC*
gene-b1244	0.297137589	0.027094917	*oppB*
gene-b1550	-0.296707526	0.781581464	*gnsB*
gene-b0430	0.296408501	0.037929321	*cyoC*
gene-b2498	-0.296279028	0.082159269	*upp*
gene-b3064	-0.296033313	0.022292279	*tsaD*
gene-b2556	-0.295912102	0.055313311	*glrK*
gene-b1981	-0.295707431	0.040574118	*shiA*
gene-b3358	-0.295579882	0.058274967	*yhfK*
gene-b3237	0.294735289	0.029015259	*argR*
gene-b1706	-0.294343178	0.06233261	*selO*
gene-b2041	0.294335974	0.045273716	*rfbB*
gene-b4472	0.294237108	0.022567049	*yhdP*
gene-b0169	-0.293626921	0.019338452	*rpsB*
gene-b3775	-0.293549996	0.038241028	*ppiC*
gene-b3244	0.293505436	0.023604404	*tldD*
gene-b2927	0.29273016	0.055895998	*epd*
gene-b1767	-0.292598122	0.037268525	*ansA*
gene-b3700	-0.292559182	0.082706959	*recF*
gene-b2179	-0.292472927	0.151812552	*yejE*
gene-b3521	-0.292269564	0.123926517	*rcdB*
gene-b0997	-0.292096809	0.156307822	*torA*
gene-b2997	-0.291901149	0.267200119	*hybO*
gene-b1412	-0.291867159	0.092567227	*azoR*

gene-b2300	0.290741882	0.037120993	*yfcE*
gene-b2133	-0.290232433	0.01350737	*dld*
gene-b0339	0.290067453	0.629841799	*cynT*
gene-b4359	-0.289908073	0.034523095	*opgB*
gene-b0621	0.28959711	0.161224452	*dcuC*
gene-b1522	-0.289296402	0.148981783	*dgcF*
gene-b4827	-0.289268769	0.150994963	*fliX*
gene-b0208	-0.289202514	0.056236247	*yafC*
gene-b2630	-0.289097222	0.029845122	*rnlA*
gene-b0735	0.289019396	0.092805148	*ybgE*
gene-b1219	0.288651574	0.049125969	*ychN*
gene-b3243	-0.288590163	0.104012658	*aaeR*
gene-b0878	0.288530917	0.021997254	*macA*
gene-b0638	-0.287894102	0.544121436	*cobC*
gene-b3224	0.287583668	0.035828807	*nanT*
gene-b1067	-0.286842498	0.049814969	*yceH*
gene-b0352	-0.286588707	0.070205528	*mhpE*
gene-b1125	0.286314532	0.106572076	*potB*
gene-b0902	-0.285676922	0.030829255	*pflA*
gene-b2504	0.283769516	0.246597116	*yfgG*
gene-b0706	-0.283086422	0.447084563	*ybfD*
gene-b3987	0.283077787	0.017822143	*rpoB*
gene-b3392	-0.282371263	0.590999109	*hofP*
gene-b1592	-0.281633669	0.37687032	*clcB*
gene-b2824	-0.281613189	0.204821014	*ygdB*
gene-b1798	0.281068351	0.354098162	*leuE*
gene-b2785	-0.280943381	0.052101391	*rlmD*
gene-b1072	0.280661281	0.374677408	*flgA*
gene-b1792	-0.28025754	0.130913358	*yeaO*
gene-b4211	-0.279725073	0.093272524	*qorB*
gene-b3382	-0.279294037	0.171837571	*yhfY*
gene-b4474	0.27901515	0.473655665	*frlC*
gene-b4553	0.278820778	0.231053758	*ysaB*
gene-b2795	0.278467154	0.098458609	*ppnN*
gene-b0104	-0.277837769	0.10879503	*guaC*
gene-b3403	-0.277808046	0.023612143	*pck*
gene-b2272	0.277188764	0.280189078	*yfbM*
gene-b4466	0.276965464	0.052420056	*yghJ*
gene-b3380	0.276917757	0.600311739	*yhfW*
gene-b3046	-0.276676862	0.663717239	*yqiG*
gene-b0399	-0.276426393	0.05371645	*phoB*
gene-b2833	0.275037248	0.037196102	*ygdR*
gene-b1207	-0.274629456	0.02117694	*prs*
gene-b1088	0.273788101	0.037349403	*yceD*
gene-b2322	-0.273754591	0.498883318	*yfcJ*
gene-b1983	-0.273306153	0.171025702	*yeeN*
gene-b2032	0.272631401	0.092013961	*wbbK*
gene-b1383	-0.272430633	0.357398242	*ydbL*
gene-b2380	-0.272312937	0.05079701	*pyrS*
gene-b1202	0.271869434	0.17542948	*ycgV*
gene-b2730	0.271776192	0.092914245	*hypE*
gene-b0481	-0.271678094	0.111950719	*ybaK*
gene-b4221	0.271225255	0.024820435	*tamB*
gene-b0467	0.271106438	0.114824532	*priC*
gene-b1689	0.27073625	0.661781989	*ydiL*
gene-b2406	-0.270221771	0.265040126	*xapB*
gene-b0175	-0.269366496	0.039946684	*cdsA*
gene-b3645	-0.268896302	0.203944029	*dinD*
gene-b1685	0.268837967	0.153694592	*ydiH*
gene-b0378	-0.268700718	0.042441696	*yaiW*
gene-b3986	-0.268681573	0.041619945	*rplL*
gene-b0418	-0.267990454	0.050185285	*pgpA*
gene-b2621	0.267882737	0.025557266	*ssrA*
gene-b2033	0.267783272	0.079794189	*wbbJ*
gene-b0930	-0.267500674	0.022937102	*asnS*
gene-b3053	0.267468397	0.042161869	*glnE*

gene-b1789	-0.267139913	0.509573313	*yeaL*
gene-b0040	0.267113505	0.638409217	*caiT*
gene-b3894	-0.266671347	0.066335887	*fdoG*
gene-b2102	-0.266546844	0.445123864	*yegX*
gene-b4342	0.266222204	0.169820264	*yjiT*
gene-b0049	-0.265950668	0.091656095	*apaH*
gene-b3487	0.26594214	0.065803241	*yhiI*
gene-b1588	0.265415173	0.154906329	*ynfF*
gene-b2220	-0.264976201	0.140794589	*atoC*
gene-b2374	-0.264903443	0.579528305	*frc*
gene-b1091	0.264642002	0.031297161	*fabH*
gene-b3727	-0.264463391	0.244990138	*pstC*
gene-b2903	-0.264137072	0.062836153	*gcvP*
gene-b3368	-0.263704083	0.046690149	*cysG*
gene-b4158	-0.263688631	0.618099695	*yjeO*
gene-b1782	-0.263574088	0.10078268	*mipA*
gene-b3399	0.263326177	0.057373677	*yrfG*
gene-b2185	0.261974909	0.043118045	*rplY*
gene-b0760	-0.261942865	0.028832768	*modF*
gene-b3850	-0.261732009	0.054651451	*hemG*
gene-b4063	-0.261584761	0.362396548	*soxR*
gene-b1054	-0.261547951	0.050051647	*lpxL*
gene-b0819	-0.261539994	0.055487507	*ldtB*
gene-b1626	-0.260201728	0.309271282	*ydgK*
gene-b3327	0.260170393	0.750602688	*gspF*
gene-b4190	-0.259421535	0.07461728	*yjfP*
gene-b0159	0.259168334	0.056671623	*mtn*
gene-b0575	0.259057035	0.179606515	*cusA*
gene-b4020	0.258868406	0.05520237	*yjbB*
gene-b2697	-0.258397008	0.038438922	*alaS*
gene-b1201	0.257581175	0.148388748	*dhaR*
gene-b0179	0.257054062	0.035434257	*lpxD*
gene-b1812	-0.257008933	0.125429572	*pabB*
gene-b3909	0.256949968	0.29381223	*kdgT*
gene-b1629	-0.256948778	0.084979432	*rsxC*
gene-b2566	0.255486256	0.049095854	*era*
gene-b0653	0.254869329	0.101647382	*gltK*
gene-b4193	-0.2547031	0.290039677	*ulaA*
gene-b2439	0.254511231	0.270356323	*eutL*
gene-b3806	-0.254364361	0.032656853	*cyaA*
gene-b0083	0.253808929	0.070526116	*ftsL*
gene-b1134	-0.253006774	0.234762606	*nudJ*
gene-b3827	-0.252981942	0.37987576	*bioP*
gene-b0087	0.252690449	0.064729616	*mraY*
gene-b3652	-0.251078299	0.106340683	*recG*
gene-b3164	-0.250448135	0.033655396	*pnp*
gene-b3791	-0.250076178	0.068407947	*wecE*
gene-b2637	-0.249855371	0.689536772	*yfjT*
gene-b0446	-0.24980688	0.157388775	*cof*
gene-b2248	0.249780338	0.374788598	*yfaX*
gene-b1539	0.249190137	0.112718987	*ydfG*
gene-b1332	0.248879901	0.112784606	*ynaJ*
gene-b0178	0.248774615	0.040750872	*skp*
gene-b2607	0.248749025	0.071629135	*trmD*
gene-b1144	0.248674142	0.5727995	*ymfJ*
gene-b0054	0.248611559	0.03682093	*lptD*
gene-b3999	-0.247951882	0.11932546	*yjaG*
gene-b3319	0.247246447	0.071647354	*rplD*
gene-b2928	-0.247170775	0.193623323	*yggC*
gene-b3792	-0.246884372	0.146844945	*wzxE*
gene-b2958	0.246874779	0.054139396	*yggN*
gene-b3393	-0.246717533	0.575766814	*hofO*
gene-b2994	-0.246539635	0.135611697	*hybC*
gene-b2017	-0.246502914	0.260313027	*yefM*
gene-b1701	0.246394289	0.618487419	*fadK*
gene-b3734	-0.246149311	0.056680991	*atpA*

gene-b0784	0.246099437	0.286531541	*moaD*
gene-b0256	-0.245939303	0.15851121	*insI1*
gene-b3225	-0.244790102	0.051704043	*nanA*
gene-b2218	-0.24395374	0.077511178	*rcsC*
gene-b1929	-0.243451622	0.060105413	*yedE*
gene-b1203	-0.243366145	0.054360945	*ychF*
gene-b1780	-0.243342938	0.08792348	*yeaD*
gene-b0870	-0.243141559	0.073633027	*ltaE*
gene-b2673	0.243131214	0.403085065	*nrdH*
gene-b1334	-0.243121996	0.063385439	*fnr*
gene-b0901	-0.242073112	0.502307623	*ycaK*
gene-b0069	0.242016104	0.127113951	*sgrR*
gene-b0592	-0.241854457	0.081630634	*fepB*
gene-b3191	0.241215337	0.083136383	*mlaB*
gene-b2170	-0.241080678	0.38453768	*setB*
gene-b0610	-0.241060554	0.125052973	*rnk*
gene-b4481	0.240765112	0.12901443	*wecF*
gene-b0194	-0.240622236	0.048584553	*proS*
gene-b2606	0.24059523	0.083777157	*rplS*
gene-b3167	0.240436517	0.123048673	*rbfA*
gene-b2233	-0.240278109	0.080143961	*yfaL*
gene-b4375	-0.239986731	0.105327858	*prfC*
gene-b3678	0.239920712	0.469515625	*yidJ*
gene-b0654	-0.239816247	0.176863323	*gltJ*
gene-b2204	-0.23944612	0.359902015	*napH*
gene-b1811	-0.238997851	0.560157293	*yoaH*
gene-b2968	0.237889977	0.51341641	*yghD*
gene-b4362	-0.237651814	0.204718359	*dnaT*
gene-b4143	-0.237643333	0.050253587	*groL*
gene-b0919	0.237599904	0.208824832	*ycbJ*
gene-b1013	-0.237598855	0.211003036	*rutR*
gene-b3033	-0.237473855	0.098685449	*yqiB*
gene-b0724	0.237116142	0.116586473	*sdhB*
gene-b2245	-0.236605198	0.276691123	*yfaU*
gene-b2479	-0.236306952	0.053517775	*gcvR*
gene-b3034	-0.235153872	0.144648016	*nudF*
gene-b1106	0.234950883	0.128891037	*thiK*
gene-b0641	0.234923935	0.062073658	*lptE*
gene-b2890	-0.234919712	0.06409893	*lysS*
gene-b1108	-0.234784111	0.090241169	*ycfP*
gene-b3802	0.234417112	0.056084189	*hemY*
gene-b2743	-0.23422084	0.079945553	*pcm*
gene-b1092	0.234150369	0.052184149	*fabD*
gene-b4408	0.233891026	0.260910123	*csrB*
gene-b3006	-0.233540091	0.093361781	*exbB*
gene-b1796	-0.233095815	0.873870144	*yoaG*
gene-b2755	0.233045107	0.255336352	*cas1*
gene-b3887	0.232793536	0.119957546	*dtd*
gene-b3292	-0.232779291	0.094425652	*zntR*
gene-b3670	0.232693796	0.224254398	*ilvN*
gene-b3910	0.232643878	0.094509975	*yiiM*
gene-b2440	0.232595929	0.183466914	*eutC*
gene-b3662	-0.232471241	0.446516878	*nepI*
gene-b4153	-0.232372851	0.146106366	*frdB*
gene-b2988	0.232251254	0.05350501	*gss*
gene-b0078	0.232232998	0.193319114	*ilvH*
gene-b3307	-0.232040748	0.157164559	*rpsN*
gene-b3209	-0.231894435	0.083679302	*elbB*
gene-b3040	0.231848433	0.076902299	*zupT*
gene-b1111	-0.231359375	0.220083274	*comR*
gene-b4532	0.231263495	0.661600621	*hicA*
gene-b1718	0.231170337	0.092316687	*infC*
gene-b2518	-0.231088543	0.301980075	*ndk*
gene-b1671	-0.230632356	0.443978006	*ydhX*
gene-b1620	0.230521878	0.173219855	*malI*
gene-b3353	0.23049776	0.100739059	*yheT*

gene-b3186	-0.230124546	0.106675072	*rplU*
gene-b3337	-0.229921001	0.361500408	*bfd*
gene-b2513	-0.22982158	0.062692929	*yfgM*
gene-b2249	-0.229432858	0.101073094	*yfaY*
gene-b3202	0.229402478	0.061157276	*rpoN*
gene-b4480	-0.229373207	0.08587134	*hdfR*
gene-b2433	0.228886094	0.377586162	*yfeZ*
gene-b0553	-0.228846812	0.241170704	*nmpC*
gene-b0655	0.228762531	0.174258548	*gltI*
gene-b0644	0.228354756	0.486518921	*ybeQ*
gene-b3708	0.228308363	0.08907906	*tnaA*
gene-b3533	0.228259856	0.398473347	*bcsA*
gene-b4317	0.227811515	0.712443266	*fimD*
gene-b4470	-0.227586581	0.250627296	*cyuA*
gene-b1185	-0.227070491	0.169996233	*dsbB*
gene-b3771	-0.226135411	0.137746526	*ilvD*
gene-b1049	-0.226094329	0.069844419	*opgH*
gene-b3930	0.226084566	0.181093118	*menA*
gene-b2709	0.225678521	0.240428856	*norR*
gene-b2909	-0.225632624	0.13336977	*ygfB*
gene-b2524	-0.224816153	0.309784237	*iscX*
gene-b0619	-0.224711401	0.239466326	*dpiB*
gene-b0185	-0.224445305	0.068185334	*accA*
gene-b0911	0.223911466	0.0572798	*rpsA*
gene-b2784	-0.223516811	0.079575355	*relA*
gene-b0120	0.223466602	0.124792531	*speD*
gene-b0431	0.223125258	0.111075698	*cyoB*
gene-b1027	-0.222852064	0.429575912	*insE4*
gene-b2088	-0.22285188	0.429568595	*insE5*
gene-b0540	-0.222850008	0.429494335	*insE3*
gene-b0373	-0.222831746	0.428770804	*insE2*
gene-b0298	-0.222830231	0.42871088	*insE1*
gene-b3804	-0.222275776	0.149655864	*hemD*
gene-b0144	-0.222157056	0.183004028	*gluQ*
gene-b1916	0.222081748	0.113697315	*sdiA*
gene-b3252	-0.222063714	0.089062184	*csrD*
gene-b3248	-0.22205831	0.183856428	*yhdE*
gene-b0830	-0.221988212	0.146760721	*gsiB*
gene-b3732	-0.221913248	0.137962391	*atpD*
gene-b2548	-0.221867689	0.340948183	*yphF*
gene-b3632	0.221589198	0.136848772	*waaQ*
gene-b4151	0.221559906	0.181551838	*frdD*
gene-b3039	-0.221345153	0.284459499	*ygiD*
gene-b3095	-0.221195245	0.113848701	*yqjA*
gene-b3657	-0.220992914	0.3967628	*yicJ*
gene-b4191	-0.2206946	0.231091161	*ulaR*
gene-b1118	-0.220571865	0.078843582	*lolE*
gene-b0355	0.219600132	0.416284046	*frmB*
gene-b4074	-0.218376197	0.443236536	*nrfE*
gene-b3773	0.217758724	0.196538307	*ilvY*
gene-b4154	-0.217398693	0.135589777	*frdA*
gene-b3060	-0.217106233	0.369009795	*ttdR*
gene-b0180	-0.216653161	0.118772432	*fabZ*
gene-b2455	0.216571361	0.612722846	*eutE*
gene-b0330	0.216506932	0.305630999	*prpR*
gene-b3236	-0.216026743	0.073435183	*mdh*
gene-b3911	-0.215692723	0.080966458	*cpxA*
gene-b3764	0.215122738	0.267699492	*maoP*
gene-b3131	-0.214816269	0.122128812	*agaR*
gene-b0960	0.214736426	0.177845774	*yccS*
gene-b0089	0.214496573	0.074117103	*ftsW*
gene-b3698	0.21403068	0.299423224	*yidB*
gene-b2099	0.214015378	0.173620815	*yegU*
gene-b3032	-0.213680591	0.131303059	*cpdA*
gene-b3616	0.21364402	0.107480819	*tdh*
gene-b0869	-0.213388266	0.135130609	*ybjT*

gene-b1800	-0.213077445	0.143967052	*dmlA*
gene-b2172	0.212911864	0.089487757	*yeiQ*
gene-b4079	-0.21270677	0.245741059	*fdhF*
gene-b2428	-0.212523227	0.294875774	*murQ*
gene-b3200	0.212455041	0.085333179	*lptA*
gene-b4482	0.21238137	0.744617146	*yigE*
gene-b4484	-0.212239155	0.196219796	*cpxP*
gene-b4389	-0.212236868	0.131422325	*radA*
gene-b0482	-0.211796293	0.284817205	*ybaP*
gene-b2564	-0.211144562	0.086850053	*pdxJ*
gene-b0429	0.211047873	0.139421791	*cyoD*
gene-b1268	0.210522048	0.255286676	*yciQ*
gene-b2586	0.210438707	0.434966122	*yfiM*
gene-b1326	-0.210367143	0.142810739	*mpaA*
gene-b3162	0.210359942	0.354742999	*deaD*
gene-b0984	-0.210336908	0.607436217	*gfcD*
gene-b1658	0.209850725	0.210240678	*purR*
gene-b1325	0.209819149	0.098778784	*ycjG*
gene-b0340	-0.20969694	0.653057485	*cynS*
gene-b1343	0.20947156	0.139611655	*dbpA*
gene-b0472	-0.209263168	0.158595397	*recR*
gene-b0853	0.20909904	0.200902741	*ybjN*
gene-b2515	-0.208791212	0.121105788	*ispG*
gene-b3683	0.208063143	0.74712962	*glvC*
gene-b3801	0.207950174	0.348229818	*aslA*
gene-b1095	0.20790464	0.094755127	*fabF*
gene-b4197	0.207577163	0.459644695	*ulaE*
gene-b2806	0.207388892	0.124538543	*rlmM*
gene-b0793	-0.206056656	0.311326581	*ybhS*
gene-b2160	0.205705737	0.131404661	*yeiI*
gene-b0114	-0.205672916	0.092833508	*aceE*
gene-b2892	-0.205439751	0.115773798	*recJ*
gene-b0591	-0.20538925	0.264904259	*entS*
gene-b3984	-0.205110528	0.076362362	*rplA*
gene-b0262	-0.205002003	0.615274638	*afuC*
gene-b0148	-0.204969642	0.176242458	*hrpB*
gene-b0242	-0.204790711	0.159275907	*proB*
gene-b1266	0.204732106	0.158031179	*rnm*
gene-b4303	0.204214598	0.292898684	*sgcQ*
gene-b0814	0.204073571	0.115932589	*ompX*
gene-b0035	-0.203989871	0.780530641	*caiE*
gene-b0479	0.203909019	0.121608159	*fsr*
gene-b3019	-0.203839694	0.110446283	*parC*
gene-b3197	-0.203736057	0.17055503	*kdsD*
gene-b3308	-0.203657743	0.113600165	*rplE*
gene-b1570	-0.203536261	0.356806968	*dicA*
gene-b0168	0.203238014	0.121305137	*map*
gene-b3405	-0.202672527	0.118815412	*ompR*
gene-b3297	-0.202592282	0.1385641	*rpsK*
gene-b2324	-0.20231074	0.140009307	*mnmC*
gene-b0497	0.201562153	0.239678023	*rhsD*
gene-b2797	0.20139806	0.137034574	*sdaB*
gene-b3349	0.201317735	0.143524715	*slyD*
gene-b3310	-0.201099415	0.131161239	*rplN*
gene-b1102	0.20101696	0.393168545	*fhuE*
gene-b1673	0.200883057	0.423877271	*ydhV*
gene-b0220	-0.19992886	0.117361816	*ivy*
gene-b2260	0.199797176	0.149791119	*menE*
gene-b2914	-0.199792629	0.140991368	*rpiA*
gene-b0688	-0.199559292	0.092211554	*pgm*
gene-b1876	-0.199540014	0.105765576	*argS*
gene-b2265	0.19904874	0.147328958	*menF*
gene-b3604	0.199027646	0.312144555	*lldR*
gene-b1852	0.198224692	0.10911573	*zwf*
gene-b1198	0.198068026	0.177838643	*dhaM*
gene-b2145	0.197908325	0.730293784	*yeiS*

gene-b2194	0.197868253	0.282617641	*ccmH*
gene-b0574	0.197196106	0.651029209	*cusB*
gene-b3706	0.196853543	0.120450513	*mnmE*
gene-b4117	0.196702138	0.581140618	*adiA*
gene-b0237	-0.196498933	0.10644743	*pepD*
gene-b3895	0.196085641	0.152590161	*fdhD*
gene-b2719	-0.195693246	0.64425667	*hycG*
gene-b2287	-0.195141525	0.139970447	*nuoB*
gene-b1193	-0.195067564	0.269249103	*emtA*
gene-b3116	-0.194896932	0.363215127	*tdcC*
gene-b1901	0.194760462	0.10793903	*araF*
gene-b2820	-0.194602658	0.165784098	*recB*
gene-b1119	0.194315262	0.110800795	*nagK*
gene-b2609	-0.194007723	0.169154807	*rpsP*
gene-b2760	-0.193938086	0.475655209	*casA*
gene-b4290	-0.193713685	0.214972766	*fecB*
gene-b3313	0.193322343	0.110088042	*rplP*
gene-b3774	-0.192871888	0.14559337	*ilvC*
gene-b1895	0.192853106	0.291871068	*uspC*
gene-b3618	-0.192762971	0.279071189	*htrL*
gene-b0612	-0.19268182	0.674881449	*citT*
gene-b3833	-0.192507282	0.195147589	*ubiE*
gene-b0770	-0.191660454	0.787072257	*ybhI*
gene-b1251	-0.191615613	0.227036594	*yciI*
gene-b4172	0.191596308	0.249987973	*hfq*
gene-b1650	-0.191587859	0.201321161	*nemA*
gene-b2354	-0.191557736	0.698791781	*yfdK*
gene-b3066	-0.191154698	0.180817061	*dnaG*
gene-b1089	-0.190716094	0.254552848	*rpmF*
gene-b4517	-0.190681672	0.811455763	*gnsA*
gene-b4200	-0.189867234	0.137532024	*rpsF*
gene-b1090	0.189699652	0.145215841	*plsX*
gene-b2298	-0.189593555	0.518162392	*yfcC*
gene-b3305	-0.189426718	0.117080265	*rplF*
gene-b3196	0.18933886	0.200285081	*yrbG*
gene-b1968	-0.189200255	0.479783797	*hprS*
gene-b1722	-0.18917708	0.449364735	*ydiY*
gene-b1184	-0.189148578	0.278906034	*umuC*
gene-b4315	0.188926293	0.738582789	*fimI*
gene-b2162	0.188796997	0.591006413	*rihB*
gene-b0417	0.188591712	0.358448628	*thiL*
gene-b2782	-0.188378224	0.386665149	*mazF*
gene-b3989	-0.187820255	0.691164634	*yjaZ*
gene-b0397	-0.187759476	0.137851186	*sbcC*
gene-b2843	0.187655575	0.209032225	*kduI*
gene-b0594	0.187565718	0.27818674	*entE*
gene-b2699	-0.187487559	0.110789785	*recA*
gene-b2034	0.187038	0.328514189	*wbbI*
gene-b4329	-0.186032785	0.704666769	*yjiG*
gene-b3259	-0.185686354	0.193154842	*prmA*
gene-b2675	-0.185634818	0.287665142	*nrdE*
gene-b4477	-0.185536746	0.544129316	*dgoA*
gene-b4141	-0.185001403	0.171944344	*yjeH*
gene-b3198	0.184796765	0.137460196	*kdsC*
gene-b3203	0.184749788	0.311386676	*hpf*
gene-b0428	0.184692186	0.158546654	*cyoE*
gene-b2379	-0.183111665	0.20075843	*alaC*
gene-b2573	0.182711503	0.155702557	*rpoE*
gene-b1777	0.182675803	0.231254198	*yeaC*
gene-b4177	-0.182508425	0.203630865	*purA*
gene-b2845	0.181847043	0.272189088	*yqeG*
gene-b3357	-0.181027402	0.175733525	*crp*
gene-b2399	-0.18089287	0.384945758	*yfeD*
gene-b0402	-0.180415236	0.286787805	*proY*
gene-b1978	-0.180291688	0.225304061	*yeeJ*
gene-b3096	-0.180125919	0.501292169	*mzrA*

gene-b2744	-0.179825909	0.21975343	*umpG*
gene-b0580	0.179375951	0.535245363	*ybdJ*
gene-b4182	-0.179180498	0.706352773	*yjfJ*
gene-b0620	-0.179051403	0.459969904	*dpiA*
gene-b3231	-0.178987387	0.215716352	*rplM*
gene-b2217	-0.178968717	0.185186396	*rcsB*
gene-b1463	-0.178582867	0.337167365	*nhoA*
gene-b3312	0.178517384	0.254286113	*rpmC*
gene-b0801	-0.178026298	0.19649568	*hcxB*
gene-b0740	0.177919329	0.190661578	*tolB*
gene-b0365	-0.177386428	0.801228882	*tauA*
gene-b0642	0.177381062	0.150265917	*leuS*
gene-b3655	0.176721532	0.180398622	*yicH*
gene-b0084	0.176463159	0.139303805	*ftsI*
gene-b0915	0.176261111	0.398829705	*lpxK*
gene-b4348	-0.176193843	0.453952266	*hsdS*
gene-b2867	0.17554621	0.575601332	*xdhB*
gene-b1481	-0.175389894	0.697083008	*bdm*
gene-b4040	0.175162403	0.286424279	*ubiA*
gene-b0882	0.174981537	0.136316077	*clpA*
gene-b2802	-0.174874589	0.298991114	*fucI*
gene-b3893	-0.174675947	0.196180268	*fdoH*
gene-b3736	-0.17455857	0.203620801	*atpF*
gene-b1885	0.174174364	0.744112347	*tap*
gene-b3067	0.173949371	0.164484115	*rpoD*
gene-b3050	0.173880274	0.636941791	*yqiJ*
gene-b0491	-0.173267977	0.382270566	*fetB*
gene-b4220	0.173007978	0.216585284	*tamA*
gene-b1606	0.172776432	0.327349999	*folM*
gene-b2165	0.171553861	0.395146119	*psuG*
gene-b0730	0.171062407	0.320893582	*mngR*
gene-b3320	0.170812745	0.209742665	*rplC*
gene-b1373	-0.170660688	0.721718287	*tfaR*
gene-b3569	-0.170624478	0.324349849	*xylR*
gene-b2908	-0.170417727	0.233268318	*pepP*
gene-b1773	0.170397849	0.538337393	*ydjI*
gene-b3550	-0.170370203	0.407785536	*yiaC*
gene-b2748	-0.170354804	0.290470096	*ftsB*
gene-b0026	-0.17023864	0.187415069	*ileS*
gene-b2320	-0.170068095	0.189488814	*pdxB*
gene-b3532	0.169846	0.494649221	*bcsB*
gene-b0381	-0.169790608	0.199359503	*ddlA*
gene-b0476	-0.169716038	0.369650635	*aes*
gene-b4237	-0.169524192	0.572233908	*nrdG*
gene-b2979	-0.169063013	0.347175537	*glcD*
gene-b1526	-0.16886768	0.397465955	*yneJ*
gene-b4469	-0.168575411	0.319036493	*ygiQ*
gene-b0088	-0.168211242	0.187725157	*murD*
gene-b1336	0.168060713	0.43065318	*abgT*
gene-b2540	0.167957374	0.886500212	*hcaC*
gene-b3009	0.16769105	0.232325416	*yghB*
gene-b0727	-0.167623042	0.246939346	*sucB*
gene-b0631	-0.167035499	0.298660348	*ybeD*
gene-b1768	0.167024801	0.332671188	*pncA*
gene-b3693	0.166503407	0.331934741	*dgoK*
gene-b0586	-0.166268427	0.198980326	*entF*
gene-b0764	-0.166133682	0.349509189	*modB*
gene-b3749	-0.166025056	0.222067175	*rbsA*
gene-b3283	-0.165870322	0.287041545	*yrdD*
gene-b2501	0.165236236	0.163819195	*ppk*
gene-b3157	-0.165152561	0.222256342	*ubiT*
gene-b1200	0.16508698	0.33374833	*dhaK*
gene-b4085	0.165050897	0.652464612	*alsE*
gene-b1717	0.164737763	0.304887192	*rpmI*
gene-b0019	0.164619148	0.28296115	*nhaA*
gene-b2821	-0.164395182	0.228429719	*ptrA*

gene-b3230	0.164284416	0.17755852	*rpsI*
gene-b2500	-0.164109471	0.407981302	*purN*
gene-b2234	0.163975785	0.360698436	*nrdA*
gene-b1199	0.163752896	0.359885135	*dhaL*
gene-b0596	-0.163577366	0.283633646	*entA*
gene-b1430	0.16353511	0.312745919	*tehB*
gene-b3622	0.163217529	0.453940358	*waaL*
gene-b2793	0.163215999	0.306354389	*syd*
gene-b0308	0.163164897	0.478106672	*ykgG*
gene-b1813	-0.162600322	0.370575493	*nudL*
gene-b3570	-0.162568814	0.296891261	*bax*
gene-b0184	0.162251267	0.179210072	*dnaE*
gene-b4138	0.162134818	0.313821255	*dcuA*
gene-b1907	0.161699547	0.308653172	*tyrP*
gene-b3713	0.161169081	0.221029106	*yieF*
gene-b3309	-0.161072619	0.253876267	*rplX*
gene-b2434	0.160855306	0.338373275	*ypeA*
gene-b4324	0.160787907	0.236911851	*uxuR*
gene-b2226	-0.160000126	0.76290603	*yfaQ*
gene-b4587	0.159984392	0.48958822	*insN*
gene-b0171	0.159733563	0.192185186	*pyrH*
gene-b0710	0.159366527	0.183496298	*ybgI*
gene-b0924	0.158655963	0.180006708	*mukB*
gene-b3988	0.158355218	0.211724059	*rpoC*
gene-b2393	0.157918434	0.305335541	*nupC*
gene-b0468	-0.157845013	0.6040766	*ybaN*
gene-b0290	0.157594619	0.467121789	*ecpD*
gene-b0444	-0.15738083	0.295438188	*queC*
gene-b0941	-0.157332032	0.612196756	*elfG*
gene-b3856	-0.157283792	0.38458051	*mobB*
gene-b0683	0.156883503	0.247187438	*fur*
gene-b4334	-0.156794027	0.555037566	*yjiL*
gene-b1731	-0.15676455	0.753278742	*cedA*
gene-b1828	-0.156042487	0.48482701	*yebQ*
gene-b2539	0.155947669	0.788773463	*hcaF*
gene-b2526	-0.15563353	0.266352041	*hscA*
gene-b3675	0.155558478	0.737665448	*yidG*
gene-b1651	0.155182291	0.271136769	*gloA*
gene-b0072	0.154879225	0.265228457	*leuC*
gene-b0150	0.154405614	0.262742807	*fhuA*
gene-b3301	-0.154356808	0.209493428	*rplO*
gene-b1656	-0.154303258	0.283408419	*sodB*
gene-b3434	0.153541135	0.41811489	*yhgN*
gene-b4043	0.153458646	0.250772726	*lexA*
gene-b0281	-0.153312787	0.459725017	*intF*
gene-b3458	-0.153096053	0.616540644	*livK*
gene-b0082	0.152757031	0.233142354	*rsmH*
gene-b2633	-0.152603256	0.721817324	*yfjQ*
gene-b0963	-0.152156343	0.264968872	*mgsA*
gene-b3318	0.152131997	0.250012752	*rplW*
gene-b0215	0.151537137	0.306197289	*dnaQ*
gene-b1232	-0.151313536	0.298427203	*purU*
gene-b3729	0.150960164	0.22562421	*glmS*
gene-b2801	0.15091138	0.618106913	*fucP*
gene-b2235	0.150688275	0.324158446	*nrdB*
gene-b0916	0.150467111	0.465644258	*ycaQ*
gene-b2990	0.15011949	0.679568418	*hybG*
gene-b0031	-0.149938723	0.303237562	*dapB*
gene-b3266	0.1498736	0.435879332	*acrF*
gene-b3117	-0.149822036	0.471463125	*tdcB*
gene-b1912	-0.149246566	0.294627433	*pgsA*
gene-b2983	-0.149246234	1	*yghQ*
gene-b0324	0.149132666	0.275689533	*yahJ*
gene-b4202	-0.148856464	0.347668123	*rpsR*
gene-b1880	-0.148789601	1	*flhB*
gene-b1249	0.148687641	0.232115641	*clsA*

gene-b4363	0.148677995	0.89980607	*yjjB*
gene-b4042	-0.14865961	0.330195638	*dgkA*
gene-b2340	0.148625094	0.23727286	*sixA*
gene-b0170	-0.148442808	0.259007943	*tsf*
gene-b2555	-0.148400305	0.458588266	*qseG*
gene-b3195	0.148383416	0.25313638	*mlaF*
gene-b0750	0.148015492	0.395970513	*nadA*
gene-b3367	-0.147807728	0.715549762	*nirC*
gene-b1460	0.147625647	0.831648348	*ydcC*
gene-b1594	-0.147527108	0.272817639	*mlc*
gene-b3234	0.147439722	0.243769113	*degQ*
gene-b0572	0.147128886	0.884060165	*cusC*
gene-b2472	-0.146968602	0.318129834	*dapE*
gene-b1070	0.146055917	0.73180077	*flgN*
gene-b3871	-0.145924985	0.256868699	*bipA*
gene-b1913	-0.145696714	0.225221848	*uvrC*
gene-b1624	-0.145562847	0.257039638	*ydgJ*
gene-b0907	-0.145367482	0.285832076	*serC*
gene-b2776	0.144786388	0.559584687	*ygcE*
gene-b0783	-0.144574193	0.305563515	*moaC*
gene-b3501	0.144276915	0.819001815	*arsR*
gene-b2703	-0.143732919	0.436683413	*srlE*
gene-b1864	-0.143451164	0.261372108	*yebC*
gene-b0091	-0.143346391	0.254387799	*murC*
gene-b1491	-0.143272587	0.374155016	*digH*
gene-b0436	-0.143019346	0.264499773	*tig*
gene-b2747	-0.142876407	0.417456416	*ispD*
gene-b3907	0.142231223	0.556054856	*rhaT*
gene-b3705	-0.14210382	0.248468744	*yidC*
gene-b0053	-0.141648917	0.238465955	*surA*
gene-b2956	-0.141354417	1	*yggM*
gene-b3017	-0.140516784	0.293475591	*ftsP*
gene-b2103	-0.140382422	0.512913348	*thiD*
gene-b0127	-0.13998274	0.273938586	*yadG*
gene-b3953	0.139142257	0.76376751	*frwD*
gene-b3323	0.139058377	0.754860464	*gspA*
gene-b2405	0.138461448	0.415633951	*xapR*
gene-b3304	-0.138287988	0.278347372	*rplR*
gene-b2763	0.138184325	0.651987129	*cysI*
gene-b1329	0.138036915	0.31943839	*mppA*
gene-b2216	0.137861452	0.305019333	*rcsD*
gene-b2718	0.137484574	0.766152364	*hycH*
gene-b2895	0.137483279	0.356677087	*fldB*
gene-b2704	-0.13735769	0.64676518	*srlB*
gene-b2286	0.137005674	0.275665128	*nuoC*
gene-b0805	0.136912797	0.616166312	*fiu*
gene-b3803	-0.136716971	0.293876003	*hemX*
gene-b3495	0.13660359	0.256737568	*uspA*
gene-b3737	-0.136327468	0.397865503	*atpE*
gene-b0015	-0.136163971	0.373575179	*dnaJ*
gene-b2426	0.13563887	0.353495873	*ucpA*
gene-b1645	-0.135621076	0.57768079	*ydhK*
gene-b1637	-0.135109792	0.30918701	*tyrS*
gene-b1107	0.134189612	0.271121454	*nagZ*
gene-b2463	-0.134098603	0.286578658	*maeB*
gene-b3169	0.134012526	0.294729513	*nusA*
gene-b3178	0.133815228	0.294457951	*ftsH*
gene-b0864	0.133731669	0.286652512	*artP*
gene-b3205	-0.133635146	0.292480355	*rapZ*
gene-b3414	-0.133015659	0.295496411	*nfuA*
gene-b1814	-0.132724967	0.252077957	*sdaA*
gene-b2315	-0.132418522	0.315716619	*folC*
gene-b0811	0.132400809	0.430266837	*glnH*
gene-b0684	-0.132247103	0.397220201	*fldA*
gene-b3602	-0.132123555	0.396462482	*yibL*
gene-b3938	0.132086709	0.480102941	*metJ*

gene-b2292	-0.131597975	0.421438473	*yfbS*
gene-b0605	-0.131277786	0.399793072	*ahpC*
gene-b0113	-0.131159399	0.411743555	*pdhR*
gene-b2840	0.130420961	0.353125264	*ygeA*
gene-b2289	-0.130211814	0.397930707	*lrhA*
gene-b1022	-0.129983931	0.686816399	*pgaC*
gene-b2016	-0.129744318	0.274497307	*yeeZ*
gene-b2489	0.12947895	0.715705199	*hyfI*
gene-b3406	-0.129422851	0.483519447	*greB*
gene-b2822	0.129388295	0.332914327	*recC*
gene-b2507	-0.129041852	0.390070883	*guaA*
gene-b4515	-0.128879805	0.810697434	*cydX*
gene-b3300	-0.128850355	0.281729878	*secY*
gene-b4261	-0.128460114	0.290507868	*lptF*
gene-b2922	-0.128086976	0.374665955	*yggE*
gene-b0932	0.128059096	0.30394834	*pepN*
gene-b3492	0.128030641	0.368902593	*yhiN*
gene-b1719	-0.127950804	0.32705624	*thrS*
gene-b1992	-0.127919501	0.514715591	*cobS*
gene-b0041	0.12785205	0.779503573	*fixA*
gene-b0212	0.127492003	0.480286674	*gloB*
gene-b3289	0.127475006	0.411865844	*rsmB*
gene-b1349	-0.127270395	0.68460464	*recT*
gene-b2530	0.127261067	0.446199269	*iscS*
gene-b0451	0.127253402	0.536420287	*amtB*
gene-b2717	0.126551059	0.440669467	*hycI*
gene-b3644	0.126393999	0.374007749	*yicC*
gene-b4112	-0.125665428	0.459220201	*basS*
gene-b2789	-0.125095694	0.677545277	*gudP*
gene-b3863	-0.124779117	0.341789637	*polA*
gene-b2800	-0.124300801	0.610583195	*fucA*
gene-b1079	0.123964372	0.788483477	*flgH*
gene-b1865	0.123917521	0.368448754	*nudB*
gene-b4015	0.123856298	0.358895785	*aceA*
gene-b0595	-0.123845193	0.395130919	*entB*
gene-b0085	-0.123332372	0.335502791	*murE*
gene-b3002	0.123311132	0.4908982	*yqhA*
gene-b4152	-0.123179665	0.500600774	*frdC*
gene-b0177	-0.122871536	0.28929805	*bamA*
gene-b3641	-0.121950878	0.451772816	*slmA*
gene-b3166	0.121923341	0.387760849	*truB*
gene-b1905	-0.121914803	0.486318006	*ftnA*
gene-b3994	-0.121689604	0.599873176	*thiC*
gene-b4044	-0.121414977	0.515275833	*dinF*
gene-b2899	-0.121333972	0.38630464	*yqfA*
gene-b3317	0.12117156	0.340367319	*rplB*
gene-b0307	-0.120798171	0.43330969	*ykgF*
gene-b0328	0.120617909	0.730360903	*yahN*
gene-b0661	0.120110533	0.360892004	*miaB*
gene-b3220	0.120039645	0.784515487	*yhcG*
gene-b3401	-0.11978225	0.377430847	*hslO*
gene-b2036	0.119459345	0.389674704	*glf*
gene-b3744	0.119445321	0.350679856	*asnA*
gene-b2642	0.118995573	0.461433031	*yfjW*
gene-b1140	-0.118394664	0.580281415	*intE*
gene-b2547	0.118335762	0.629071174	*yphE*
gene-b3192	-0.11821273	0.314734842	*mlaC*
gene-b3464	-0.117720412	0.323039757	*ftsY*
gene-b3126	0.11722658	0.638996546	*garL*
gene-b2450	0.116732925	0.473420459	*yffS*
gene-b3233	-0.116658651	0.397686312	*zapG*
gene-b0441	-0.116463311	0.359979195	*ppiD*
gene-b4147	0.116422189	0.375954691	*efp*
gene-b2331	-0.115680169	0.428337426	*smrB*
gene-b2712	-0.115141512	0.510224724	*hypF*
gene-b4155	-0.115087237	0.444200106	*epmA*

gene-b2799	-0.114984208	0.47106462	*fucO*
gene-b0515	-0.114972314	0.596685106	*allE*
gene-b1068	-0.114818199	0.353527911	*yceM*
gene-b1634	0.114815584	0.447036956	*dtpA*
gene-b0905	0.11459189	0.397048719	*ycaO*
gene-b2400	-0.114573437	0.348977293	*gltX*
gene-b2803	-0.114364166	0.517314349	*fucK*
gene-b0039	-0.113554117	0.639888459	*caiA*
gene-b1707	0.113438883	0.780705612	*rflP*
gene-b4403	-0.113203383	0.452585598	*yjtD*
gene-b2196	0.112822214	0.483306203	*ccmF*
gene-b3581	0.112685175	0.788520702	*sgbH*
gene-b1439	-0.112496981	0.414639007	*ydcR*
gene-b3932	0.11215964	0.417779951	*hslV*
gene-b3617	-0.111994693	0.367097241	*kbl*
gene-b0094	0.111825782	0.337069839	*ftsA*
gene-b3374	0.110715679	0.728024503	*frlD*
gene-b3752	-0.110603301	0.364915711	*rbsK*
gene-b0313	-0.110068503	0.546563348	*betI*
gene-b0528	-0.109507109	0.75569852	*ybcJ*
gene-b1501	-0.109418876	0.66159252	*ydeP*
gene-b1071	-0.10905101	0.833824378	*flgM*
gene-b1350	0.108678116	0.745845114	*recE*
gene-b2750	0.108675476	0.697000974	*cysC*
gene-b4320	0.108544154	0.829575364	*fimH*
gene-b0312	0.108439146	0.516545592	*betB*
gene-b1618	0.108267853	0.481037858	*uidR*
gene-b3294	-0.108200982	0.370193438	*rplQ*
gene-b0913	0.107966746	0.435981653	*ycaI*
gene-b1871	0.107029401	0.430319566	*cmoB*
gene-b4468	0.106976961	0.661442131	*glcE*
gene-b1340	-0.106240421	0.59282381	*smrA*
gene-b1771	-0.106051576	0.627147095	*ydjG*
gene-b2745	-0.105699251	0.387777127	*truD*
gene-b3311	0.105580507	0.501302788	*rpsQ*
gene-b3316	0.105428797	0.416970811	*rpsS*
gene-b2599	-0.105230394	0.506118274	*pheA*
gene-b2883	-0.104872724	0.64696408	*guaD*
gene-b3193	0.104686219	0.405858242	*mlaD*
gene-b3981	0.104360442	0.498050103	*secE*
gene-b2224	0.104256354	0.782455574	*atoB*
gene-b2202	-0.104107208	0.663923564	*napC*
gene-b0567	0.103996932	0.617919973	*ybcH*
gene-b3559	0.10382221	0.400626526	*glyS*
gene-b0601	0.103157461	0.746641196	*ybdM*
gene-b4159	-0.102767026	0.418875637	*mscM*
gene-b2812	-0.102624641	0.498264422	*tcdA*
gene-b2069	-0.102312298	0.549210736	*yegD*
gene-b3957	-0.102098441	0.399927005	*argE*
gene-b1533	0.10205039	0.53032823	*eamA*
gene-b2195	0.101776467	0.663009207	*ccmG*
gene-b4235	0.101764161	0.461599526	*tldE*
gene-b0463	-0.101432896	0.414082774	*acrA*
gene-b0657	-0.101381343	0.536897902	*lnt*
gene-b0732	-0.101335425	0.54649843	*mngB*
gene-b0604	0.099935784	0.541263851	*dsbG*
gene-b0843	-0.099601809	0.81371547	*ybjH*
gene-b2746	-0.099487791	0.580761689	*ispF*
gene-b2166	0.099404495	0.658325027	*psuK*
gene-b0050	0.099286325	0.635434387	*apaG*
gene-b2906	-0.099240697	0.517461888	*ubiI*
gene-b0095	-0.098751336	0.402665729	*ftsZ*
gene-b4213	-0.098710392	0.425696394	*cpdB*
gene-b3284	-0.09868049	0.439957732	*smg*
gene-b2844	-0.098668854	0.456936428	*yqeF*
gene-b2013	0.098524867	0.729014927	*tsuA*

gene-b0959	0.098266396	0.494191555	*sxy*
gene-b0934	0.098161123	0.917299449	*ssuC*
gene-b4242	-0.097912555	0.553080688	*mgtA*
gene-b3580	-0.097869723	0.868640069	*lyxK*
gene-b1696	-0.097264242	0.627769211	*ydiP*
gene-b2787	0.09615895	0.562229033	*gudD*
gene-b3995	-0.096050029	0.500257194	*rsd*
gene-b4335	-0.095789046	0.589123886	*yjiM*
gene-b0847	0.095600612	0.501246079	*ybjL*
gene-b4162	-0.095202344	0.546059152	*orn*
gene-b1372	0.095124662	0.722947656	*stfR*
gene-b0115	-0.095099976	0.478481124	*aceF*
gene-b2689	0.09480132	0.650777639	*yqaA*
gene-b3247	-0.094535285	0.456343447	*rng*
gene-b3352	-0.09439763	0.471901544	*yheS*
gene-b1269	0.094056021	0.492619918	*rluB*
gene-b1938	0.093577311	0.909130434	*fliF*
gene-b4201	-0.093291078	0.43570381	*priB*
gene-b3997	-0.09295506	0.486712345	*hemE*
gene-b2614	-0.092907933	0.513858009	*grpE*
gene-b0439	0.092403623	0.433417911	*lon*
gene-b2834	0.092313512	0.459397119	*tas*
gene-b4288	-0.092287746	0.803147043	*fecD*
gene-b2628	0.091957856	0.48409425	*abpA*
gene-b3350	0.091622633	0.485044032	*kefB*
gene-b1066	-0.091470533	0.498130321	*rimJ*
gene-b2182	-0.091381464	0.588439402	*bcr*
gene-b3486	-0.091260374	0.613445369	*rbbA*
gene-b2866	0.091126025	0.666691514	*xdhA*
gene-b3885	-0.090973581	0.507498272	*yihX*
gene-b1806	-0.090679972	0.49905752	*yeaY*
gene-b0833	0.090568969	0.817109215	*pdeI*
gene-b2319	-0.09046309	0.551283292	*usg*
gene-b2418	-0.089736651	0.562115428	*pdxK*
gene-b3377	0.089707504	0.828199928	*yhfT*
gene-b0634	-0.089599155	0.574097909	*mrdB*
gene-b4321	0.089452124	0.728934994	*gntP*
gene-b2847	0.088990889	0.812063635	*yqeI*
gene-b1272	0.088374354	0.466798439	*sohB*
gene-b0802	-0.088362992	0.794611177	*ybiJ*
gene-b2210	0.088311069	0.659857443	*mqo*
gene-b1863	-0.088019994	0.506536088	*ruvC*
gene-b2723	0.08800407	0.865197415	*hycC*
gene-b3888	0.087962011	0.504508879	*fabY*
gene-b4014	-0.087825309	0.516754369	*aceB*
gene-b3679	0.087131678	0.812403364	*yidK*
gene-b1081	0.086902491	1	*flgJ*
gene-b2923	0.08650353	0.742959048	*argO*
gene-b2544	-0.086296403	0.735731579	*yphB*
gene-b3619	-0.085938048	0.497474931	*rfaD*
gene-b1056	-0.085874841	0.644592304	*yceI*
gene-b2383	-0.085258119	0.735852114	*fryA*
gene-b1850	0.084956914	0.525339458	*eda*
gene-b3303	-0.08480345	0.470001362	*rpsE*
gene-b2771	0.084742847	0.915252587	*ygcS*
gene-b2368	-0.084387876	0.913409637	*emrK*
gene-b2180	-0.083773624	0.568535329	*yejF*
gene-b0693	-0.083741446	0.842823587	*speF*
gene-b3794	0.08365292	0.648911359	*rffM*
gene-b4170	-0.08340958	0.537063212	*mutL*
gene-b3306	-0.083259453	0.521214251	*rpsH*
gene-b2946	0.082945237	0.559166765	*rsmE*
gene-b2197	0.082408078	0.767876779	*ccmE*
gene-b3795	-0.082293248	0.575121841	*yifK*
gene-b1736	0.082250747	0.871070124	*chbA*
gene-b1189	0.081751816	0.616521897	*dadA*

gene-b2947	-0.081640643	0.513506726	*gshB*
gene-b2441	0.081392877	0.623255472	*eutB*
gene-b3868	-0.080954796	0.605859174	*glnG*
gene-b0408	-0.080917488	0.534064258	*secD*
gene-b3376	0.080670887	0.782827097	*yhfS*
gene-b1120	-0.080254114	0.559543253	*cobB*
gene-b3194	0.079263532	0.544961104	*mlaE*
gene-b1130	0.078209289	0.561584954	*phoP*
gene-b0765	0.078080915	0.584201949	*modC*
gene-b1328	0.077749096	0.666974928	*pgrR*
gene-b3661	0.077373013	0.728728978	*nlpA*
gene-b3065	0.077340268	0.560375628	*rpsU*
gene-b2001	-0.077260164	0.732834938	*yeeR*
gene-b0051	-0.077241519	0.613727226	*rsmA*
gene-b1218	-0.077038354	0.640488428	*chaC*
gene-b1019	0.076995326	0.670384712	*efeB*
gene-b2231	-0.076721377	0.535217724	*gyrA*
gene-b0118	0.075942783	0.585898747	*acnB*
gene-b3730	-0.075134434	0.521587406	*glmU*
gene-b0278	0.074975797	1	*yagL*
gene-b3807	-0.074496079	0.666894795	*cyaY*
gene-b2823	-0.074452378	0.829509191	*ppdC*
gene-b2545	-0.074233962	0.749220364	*yphC*
gene-b0726	0.074214788	0.625168102	*sucA*
gene-b2554	0.074128487	0.689868023	*glrR*
gene-b0116	-0.073055544	0.554085395	*lpd*
gene-b2201	-0.07285574	0.718271178	*ccmA*
gene-b2581	-0.072466347	0.564267187	*yfiF*
gene-b4081	-0.072138609	1	*mdtO*
gene-b2364	-0.072078818	0.775800634	*dsdC*
gene-b0341	-0.071646408	0.938058566	*cynX*
gene-b3835	-0.071472653	0.615941763	*ubiB*
gene-b4226	0.071372822	0.589118338	*ppa*
gene-b3714	0.071340733	0.710522535	*adeP*
gene-b4171	-0.071245254	0.578218187	*miaA*
gene-b0471	0.071028339	0.59264437	*ybaB*
gene-b1713	-0.071013229	0.561830079	*pheT*
gene-b1779	-0.070795801	0.557173554	*gapA*
gene-b2193	0.070685447	0.663359872	*narP*
gene-b2622	-0.07066279	0.615316744	*intA*
gene-b4322	0.070631439	0.621667928	*uxuA*
gene-b0455	-0.070381074	0.866186766	*ffs*
gene-b3325	0.070330716	0.85482752	*gspD*
gene-b3258	-0.070257503	0.726104614	*panF*
gene-b1670	-0.070138329	0.790250521	*ydhU*
gene-b0728	-0.070031926	0.60658092	*sucC*
gene-b1309	-0.069930034	0.91443192	*ycjM*
gene-b1435	-0.069791445	0.589909865	*rlhA*
gene-b4252	0.069471711	0.596794665	*tabA*
gene-b0073	-0.069469535	0.605376049	*leuB*
gene-b3861	-0.069291328	0.903354447	*yihF*
gene-b0258	0.069285302	0.741245203	*ykfC*
gene-b2505	-0.068672705	1	*yfgH*
gene-b4536	0.06767548	0.825526055	*yobH*
gene-b3892	-0.06756251	0.675171996	*fdoI*
gene-b2314	-0.067552782	0.639502516	*dedD*
gene-b2819	-0.067147434	0.613041217	*recD*
gene-b4206	-0.066955162	0.600732581	*ytfB*
gene-b0822	-0.066801273	0.724384507	*ybiV*
gene-b1612	-0.066639858	0.624788721	*fumA*
gene-b2352	-0.066370599	0.838012722	*yfdI*
gene-b3400	0.066085847	0.734729482	*hslR*
gene-b1752	-0.066002132	0.75387536	*ydjZ*
gene-b1075	-0.065893478	1	*flgD*
gene-b3063	0.065816167	1	*ttdT*
gene-b1338	-0.065658036	0.713510666	*abgA*

gene-b2174	0.064736106	0.786195816	*lpxT*
gene-b2064	-0.064336683	0.590783689	*asmA*
gene-b1974	-0.064119825	0.918685935	*yodB*
gene-b3471	0.063637727	0.799361288	*yhhQ*
gene-b3848	-0.063490093	0.677582748	*yigZ*
gene-b1288	0.063410809	0.64333403	*fabI*
gene-b3523	0.062970261	0.749735819	*yhjE*
gene-b0723	0.062874089	0.662652905	*sdhA*
gene-b3282	0.062753924	0.6784109	*tsaC*
gene-b2303	-0.062504096	0.775672274	*folX*
gene-b2247	0.062271361	0.666072081	*rhmD*
gene-b3789	-0.062220797	0.693991638	*rffH*
gene-b0057	-0.062219015	1	*yabQ*
gene-b3931	0.061774344	0.645896122	*hslU*
gene-b0834	0.061343595	0.926586898	*dgcI*
gene-b0850	0.061296509	0.869781082	*ybjC*
gene-b0640	-0.060721584	0.682240631	*holA*
gene-b2766	0.060384888	0.830994957	*ygcN*
gene-b3204	0.060301449	0.655435842	*ptsN*
gene-b0096	-0.060230922	0.598898767	*lpxC*
gene-b3551	0.060169632	0.636685843	*bisC*
gene-b1109	-0.060151263	0.668863899	*ndh*
gene-b1630	-0.05996297	0.76541398	*rsxD*
gene-b4123	-0.059506362	0.828084668	*dcuB*
gene-b2502	0.058667955	0.649116903	*ppx*
gene-b1595	-0.058582866	0.821098181	*ynfL*
gene-b4203	0.058533489	0.637214482	*rplI*
gene-b1451	0.058397902	0.784702511	*yncD*
gene-b2917	0.057239938	0.872341836	*scpA*
gene-b0914	0.057076833	0.671364832	*msbA*
gene-b2811	0.056172936	0.68827547	*csdE*
gene-b3503	-0.055965286	0.908885632	*arsC*
gene-b0003	-0.055701504	0.672042052	*thrB*
gene-b3170	0.055593525	0.721219367	*rimP*
gene-b1528	-0.05552318	0.822460628	*ydeA*
gene-b2178	-0.055181755	0.804172037	*yejB*
gene-b4479	0.055098353	0.716282653	*dgoR*
gene-b3862	-0.054811049	0.908439754	*yihG*
gene-b3847	0.054556767	0.649917238	*pepQ*
gene-b0176	-0.054536332	0.655710556	*rseP*
gene-b4355	-0.054265735	0.808174875	*tsr*
gene-b2124	-0.054049195	0.777073659	*yehS*
gene-b3081	-0.052587793	0.739132363	*fadH*
gene-b2796	0.052288744	0.674443012	*sdaC*
gene-b0942	0.051763763	1	*ycbU*
gene-b1204	-0.051478428	0.716788345	*pth*
gene-b0219	0.050791423	0.705609823	*yafV*
gene-b3299	0.050646934	0.894037323	*rpmJ*
gene-b3772	0.050549045	0.743440009	*ilvA*
gene-b1084	0.050387478	0.732156654	*rne*
gene-b3216	0.050108118	0.90232841	*yhcD*
gene-b3485	0.050055985	0.774990177	*yhhJ*
gene-b3575	0.049678487	0.826871667	*yiaK*
gene-b4538	0.049526511	1	*yoeF*
gene-b0092	0.049495178	0.690321311	*ddlB*
gene-b2531	0.049079386	0.793855705	*iscR*
gene-b2155	0.048760489	0.826264645	*cirA*
gene-b2553	0.048637651	0.789943299	*glnB*
gene-b1870	-0.048301729	0.771181969	*cmoA*
gene-b1128	-0.04829621	0.717826914	*roxA*
gene-b3649	0.048263102	0.715818287	*rpoZ*
gene-b0908	-0.047404226	0.746941416	*aroA*
gene-b3427	0.047182074	1	*yzgL*
gene-b2163	-0.046306474	0.899056444	*yeiL*
gene-b2516	0.046277932	0.746510297	*rodZ*
gene-b1851	-0.046060739	0.748101804	*edd*

gene-b3731	-0.045468965	0.756157415	*atpC*
gene-b3573	-0.044770411	0.845829427	*ysaA*
gene-b3315	0.044675375	0.720036507	*rplV*
gene-b2294	-0.044516762	0.728776649	*yfbU*
gene-b3557	0.043976787	0.948065226	*insJ*
gene-b3461	0.043914837	0.727385298	*rpoH*
gene-b0251	0.043883749	1	*yafY*
gene-b3820	0.043543885	0.92202761	*yigI*
gene-b1422	0.043531583	0.829666162	*ydcI*
gene-b3030	-0.04341061	0.738565678	*parE*
gene-b0611	-0.042831629	0.779418952	*rna*
gene-b2705	-0.042373609	0.784532375	*srlD*
gene-b4392	-0.04212813	0.737783032	*slt*
gene-b2961	-0.041220767	0.775021414	*mutY*
gene-b1655	0.041179597	0.792654571	*mepH*
gene-b1053	-0.040838029	0.898094909	*mdtG*
gene-b0465	-0.040784917	0.757867428	*mscK*
gene-b2000	0.040533983	0.808712668	*flu*
gene-b3460	0.040243022	0.843533079	*livJ*
gene-b2568	0.040234347	0.762445418	*lepB*
gene-b2907	-0.039796934	0.809281577	*ubiH*
gene-b1531	0.038487984	0.916523386	*marA*
gene-b1330	-0.038185538	0.898237038	*ynaI*
gene-b1754	0.037773151	0.810564514	*ynjB*
gene-b3181	0.037494335	0.802065846	*greA*
gene-b3836	-0.03734874	0.804609899	*tatA*
gene-b0763	-0.036504047	0.82903679	*modA*
gene-b3880	0.03643572	0.874191575	*yihS*
gene-b4179	-0.036236147	0.794056508	*rnr*
gene-b3726	-0.036199472	0.88042624	*pstA*
gene-b3832	-0.036113302	0.800626072	*rmuC*
gene-b4461	0.035419782	0.803575195	*yfjD*
gene-b3071	-0.034674948	0.876123748	*nfeR*
gene-b2079	0.034308871	0.835924725	*baeR*
gene-b3493	0.032777022	0.806476636	*pitA*
gene-b2097	0.032507555	0.828132513	*fbaB*
gene-b0858	-0.032313785	0.919236974	*ybjO*
gene-b3592	0.031941391	0.881702456	*yibF*
gene-b2430	0.031568856	0.905138518	*yfeW*
gene-b1599	0.031294834	1	*mdtI*
gene-b3656	0.031267117	0.875294791	*yicI*
gene-b0998	-0.031222982	0.948361535	*torD*
gene-b0829	0.031158173	0.841090684	*gsiA*
gene-b0111	-0.031134446	0.816064885	*ampE*
gene-b0337	-0.030942433	0.884172427	*codA*
gene-b2341	0.03090356	0.818476207	*fadJ*
gene-b2388	0.030548663	0.810093944	*glk*
gene-b3982	-0.030121288	0.819730214	*nusG*
gene-b0948	0.029811422	0.83540565	*rlmL*
gene-b0918	-0.029218778	0.829172544	*kdsB*
gene-b3404	-0.028988667	0.845084713	*envZ*
gene-b0597	0.028584475	0.881424128	*entH*
gene-b0311	0.02824722	0.875678138	*betA*
gene-b0512	0.028023118	0.891845074	*allB*
gene-b2546	0.027850192	1	*yphD*
gene-b0090	0.027313367	0.835966793	*murG*
gene-b3718	0.027162754	1	*yieK*
gene-b2288	0.026314874	0.850544724	*nuoA*
gene-b4216	-0.026009062	0.854380376	*ytfJ*
gene-b1057	-0.025750625	0.903500649	*yceJ*
gene-b1538	0.025475748	0.85710123	*dcp*
gene-b1359	0.025466783	0.942111647	*ydaU*
gene-b3185	-0.025337531	0.871713186	*rpmA*
gene-b0045	0.025222417	1	*yaaU*
gene-b0009	0.025207764	0.853961637	*mog*
gene-b0086	0.025109065	0.850778334	*murF*

gene-b1581	-0.024672699	0.886462252	*rspA*
gene-b3256	0.024183959	0.883973869	*accC*
gene-b4272	-0.023709794	0.917579588	*insC6*
gene-b1997	-0.023709745	0.91757663	*insC3*
gene-b3044	-0.023709592	0.917567452	*insC5*
gene-b2861	-0.023709564	0.917565804	*insC4*
gene-b0360	-0.023707852	0.91746348	*insC1*
gene-b1403	-0.023707735	0.91745652	*insC2*
gene-b3255	0.023532846	0.908855966	*accB*
gene-b2576	-0.02336447	0.862128473	*srmB*
gene-b1654	-0.022729606	0.89516439	*grxD*
gene-b1479	-0.022569162	0.859691284	*maeA*
gene-b2574	0.022102071	0.926280052	*nadB*
gene-b0004	-0.02172946	0.858942104	*thrC*
gene-b1988	0.021387477	1	*nac*
gene-b2762	0.021267918	0.940944053	*cysH*
gene-b1080	0.021204158	1	*flgI*
gene-b4130	-0.021057809	0.936383051	*dtpC*
gene-b0282	0.020937659	1	*yagP*
gene-b2143	-0.020608994	0.893627164	*cdd*
gene-b2264	-0.020582249	0.916142304	*menD*
gene-b3144	-0.020248459	0.958992587	*yraJ*
gene-b1546	0.019888618	1	*tfaQ*
gene-b0785	0.019886793	0.914785618	*moaE*
gene-b4795	-0.019815101	1	*yibX*
gene-b0945	0.019526097	0.917792176	*pyrD*
gene-b0221	0.018447196	0.897482023	*fadE*
gene-b0593	-0.018218941	0.925314065	*entC*
gene-b2592	-0.018035367	0.883699164	*clpB*
gene-b3475	0.018029714	0.948024201	*acpT*
gene-b2246	-0.017777274	0.968222719	*yfaV*
gene-b3691	0.017773262	1	*dgoT*
gene-b2068	-0.017725156	0.926796324	*alkA*
gene-b3321	0.017563179	0.909384307	*rpsJ*
gene-b2263	-0.0172839	0.977177302	*menH*
gene-b1687	0.016974719	0.913276699	*ydiJ*
gene-b0627	0.015998341	0.953741696	*tatE*
gene-b4148	-0.01562452	1	*gdx*
gene-b3260	-0.015304161	0.939262901	*dusB*
gene-b2423	0.015084971	0.943181135	*cysW*
gene-b1740	-0.014682767	0.912797111	*nadE*
gene-b1758	-0.014553319	0.941449375	*ynjF*
gene-b3881	0.01426661	0.974576721	*yihT*
gene-b3719	-0.013864267	1	*yieL*
gene-b0715	-0.012618503	1	*abrB*
gene-b0173	-0.012391121	0.931041737	*dxr*
gene-b4058	0.011586446	0.939888156	*uvrA*
gene-b2715	0.011570286	0.967237218	*ascF*
gene-b2608	0.010778201	0.935197115	*rimM*
gene-b2904	0.010653692	0.949042937	*gcvH*
gene-b3241	-0.010286776	1	*aaeA*
gene-b0407	-0.010228388	0.950808764	*yajC*
gene-b1635	0.009927377	0.937793974	*gstA*
gene-b3314	0.009049032	0.940164276	*rpsC*
gene-b4763	0.008889532	0.967669466	*sroC*
gene-b2208	0.008660106	1	*napF*
gene-b3013	-0.008653031	1	*yqhG*
gene-b4113	-0.008451546	0.966085936	*basR*
gene-b0890	-0.008350651	0.944637899	*ftsK*
gene-b3463	0.008008106	0.956211759	*ftsE*
gene-b2365	-0.007950892	1	*dsdX*
gene-b2881	0.006940969	0.990595148	*xdhD*
gene-b2698	0.006725456	1	*recX*
gene-b3391	-0.0066351	1	*hofQ*
gene-b2346	-0.006424423	0.960885812	*mlaA*
gene-b3952	0.006403985	1	*pflC*

gene-b3295	0.005946696	0.960356222	*rpoA*
gene-b2757	0.005945834	1	*casD*
gene-b3296	-0.005759496	0.962346428	*rpsD*
gene-b1830	0.005539884	0.966656075	*prc*
gene-b1709	0.005075016	0.989269845	*btuD*
gene-b0652	-0.004847225	0.97649854	*gltL*
gene-b4253	0.003782488	0.984400138	*yjgL*
gene-b1930	0.003685716	1	*yedF*
gene-b1278	0.003479851	0.990124898	*pgpB*
gene-b3702	-0.003380973	0.985858038	*dnaA*
gene-b1854	0.002919565	0.980849723	*pykA*
gene-b4268	0.002915625	1	*idnK*
gene-b3896	0.002307812	1	*yiiG*
gene-b0982	-0.002162012	1	*etp*
gene-b0449	-0.001987472	1	*mdlB*
gene-b3293	0.001843181	1	*yhdN*
gene-b0729	-0.001818241	0.989436338	*sucD*
gene-b0076	0.001269975	0.995456685	*leuO*
gene-b3998	-0.001118355	1	*nfi*
gene-b0067	-0.001038329	1	*thiP*
gene-b3411	0.000883192	1	*rpnA*
gene-b3688	-0.000115391	1	*yidQ*
gene-b4196	1.88E-05	1	*ulaD*

The genes related to the colanic acid (CA) synthesis and glutamate-GABA metabolism are highlighted in blue and yellow, respectively. *rcsA* gene is colored in red.
