# Supplementary figures and images for: Fitness costs of mobilised colistin resistance gene 3 (mcr-3): systematic review, epidemiological study, and functional analysis
Source: eBioMedicine. 2025 Sep 12;120:105923. doi: 10.1016/j.ebiom.2025.105923 (PMC12571581; doi:10.1016/j.ebiom.2025.105923)

**Fig 3e**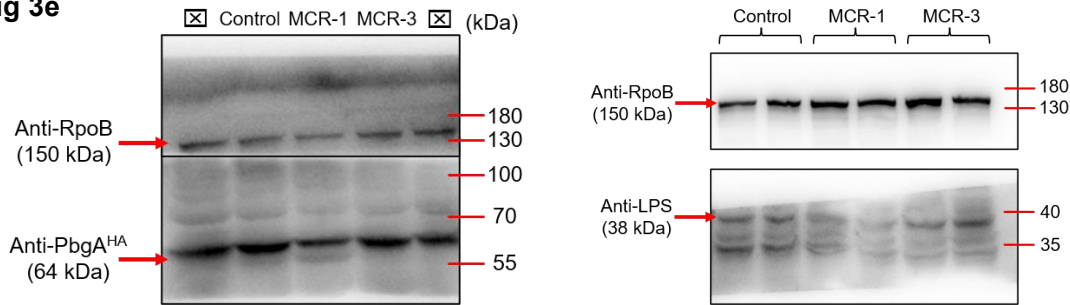**Fig 4b**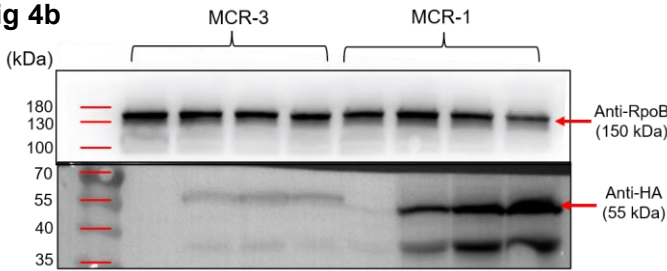**Fig 4d**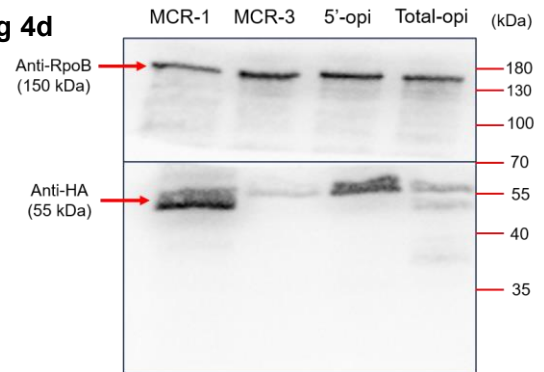**Fig 5b**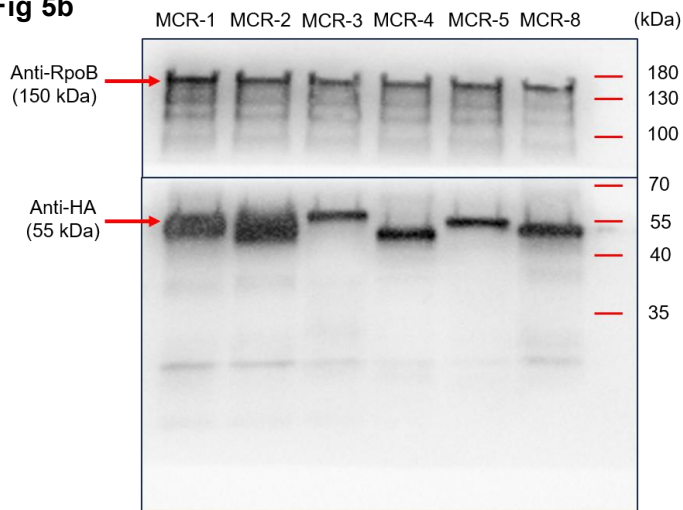**Fig S14a**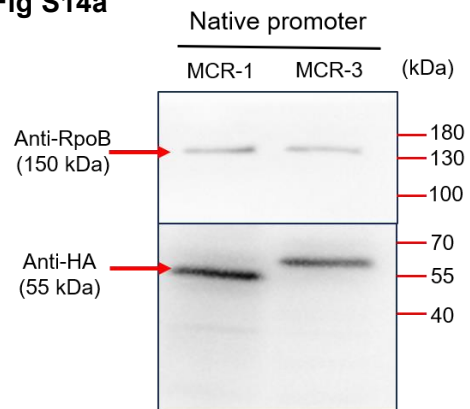**Fig S17b**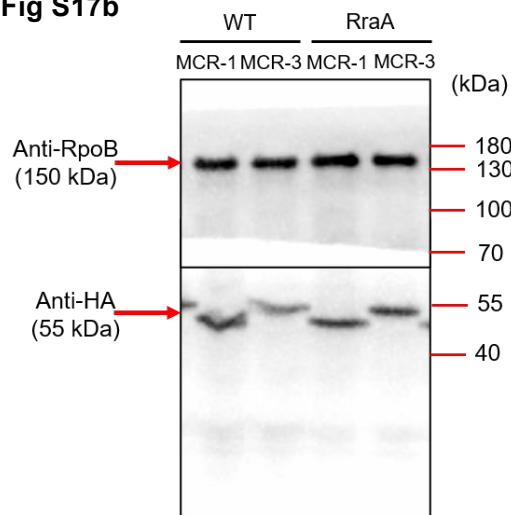

Supplement: Western blot [file mmc7.pdf]
